# Attention/Working Memory and Executive Function in Parkinson's Disease: Review, Critique, and Recommendations

**DOI:** 10.1002/mds.30293

**Published:** 2025-07-18

**Authors:** Roberta Biundo, Ondrej Bezdicek, Davide Maria Cammisuli, Brenna Cholerton, John C. Dalrymple‐Alford, Nicola Edelstyn, Eleonora Fiorenzato, Erin Holker, Saul Martinez‐Horta, Alice Martini, Gabriella Santangelo, Barbara Segura, Chiara Siri, Alexander Tröster, Tiago A. Mestre, Álvaro Sánchez Ferro, Michelle Hyczy de Siqueira Tosin, Matej Skorvanek, Daniel Weintraub, Gert J. Geurtsen, the members of the MDS Clinical Outcome Assessment Scientific Evaluation Committee

**Affiliations:** ^1^ Department of General Psychology University of Padua Padua Italy; ^2^ IRCCS, San Camillo Hospital Venice Italy; ^3^ Department of Neurology and Centre of Clinical Neuroscience First Faculty of Medicine and General University Hospital in Prague, Charles University Prague Czech Republic; ^4^ Department of Psychology Catholic University of the Sacred Heart Milan Italy; ^5^ Department of Pathology Stanford University School of Medicine Stanford California USA; ^6^ Te Kura Mahi ā‐Hirikapo School of Psychology, Speech and Hearing University of Canterbury Christchurch New Zealand; ^7^ New Zealand Brain Research Institute Christchurch New Zealand; ^8^ Department of Medicine University of Otago Dunedin New Zealand; ^9^ Department of Psychology Bath Spa University Bath UK; ^10^ Neurodegenerative Disease Unit, Department of Neuroscience University of Padua Padua Italy; ^11^ Department of Rehabilitation Medicine University of Minnesota Minneapolis Minnesota USA; ^12^ Movement Disorders Unit, Department of Neurology Hospital de la Santa Creu i Sant Pau, Research Institute Sant Pau Barcelona Spain; ^13^ Centro de Investigagión Biomédica en Red‐Enfermedades Neurodegenerativas (CIBERNED) Madrid Spain; ^14^ Addiction and Mental Health Department Azienda Sanitaria Friuli Occidentale Pordenone Italy; ^15^ Department of Psychology University of Campania, “Luigi Vanvitelli” Caserta Italy; ^16^ Medical Psychology Unit, Department of Medicine Institute of Neurosciences, University of Barcelona Barcelona Spain; ^17^ Institut d'Investigacions Biomèdiques August Pi i Sunyer (IDIBAPS) Barcelona Spain; ^18^ Centro de Investigación Biomédica en Red sobre Enfermedades Neurodegenerativas Barcelona Spain; ^19^ Parkinson's Disease and Movement Disorders Neurorehabilitation Unit Moriggia Pelascini Hospital Como Italy; ^20^ Department of Clinical Neuropsychology and Center for Neuromodulation Barrow Neurological Institute Phoenix Arizona USA; ^21^ Parkinson's Disease and Movement Disorders Center, Division of Neurology, Department of Medicine The Ottawa Hospital Research Institute, University of Ottawa Brain and Mind Institute Ottawa Ontario Canada; ^22^ Movement Disorders Unit, Neurology Department Hospital Universitario 12 de Octubre Madrid Spain; ^23^ Department of Neurological Sciences Rush University Medical Center Chicago Illinois USA; ^24^ Department of Neurology Safarik University Kosice Slovakia; ^25^ Department of Neurology University Hospital of Louis Pasteur Kosice Slovakia; ^26^ Department of Psychiatry University of Pennsylvania School of Medicine; Parkinson's Disease Research, Education and Clinical Center (PADRECC), Philadelphia Veterans Affairs Medical Center Philadelphia Pennsylvania USA; ^27^ Department of Medical Psychology Amsterdam UMC Location, University of Amsterdam, Amsterdam Neuroscience Amsterdam The Netherlands

**Keywords:** attention‐executive functions, cognition, Parkinson's disease, psychometric properties, rating test

## Abstract

**Background:**

Cognitive impairment in Parkinson's disease (PD) is a well‐established non‐motor complication that significantly affects the quality of life and well‐being of both patients and care partners. To optimally detect mild cognitive impairment or dementia, extensive neuropsychological assessment is essential. A wide range of cognitive tests and clinical outcome assessments have been used in clinical settings, often without regard to their clinimetric quality.

**Methods:**

We performed a literature review of tests assessing attention/working memory and executive domains in PD (tests on other domains are included in an accompanying review). The selected tests were evaluated for their clinimetric properties and categorized by a panel of experts as “recommended,” “recommended with caveats,” “suggested,” or “listed” according to the International Parkinson and Movement Disorder Society Clinical Outcome Assessment Scientific Evaluation Committee guidelines.

**Results:**

A total of 30 tests were reviewed. Eight tests were “recommended,” including four tests assessing attention/working memory abilities (WAIS‐IV Digit Span, Coding and Symbol Search subtests, and Trail Making Test) and four tests assessing executive abilities (WAIS‐IV Similarities, Wisconsin Card Sorting Test, Fluency Tests, and Stroop Color‐Word Test). These tests demonstrated good to excellent levels of reliability and validity, have normative datasets, and are sensitive to change. Eight other tests were “recommended with caveats”, eleven were “suggested,” and three were “listed.”

**Conclusions:**

The recommended tests for attention/working memory and executive functioning in PD can guide PD cognitive assessment. Other tests were identified as potentially useful; however, caution is advised due to their clinimetric limitations. Further validation studies are required for these tests. © 2025 The Author(s). *Movement Disorders* published by Wiley Periodicals LLC on behalf of International Parkinson and Movement Disorder Society.

The diagnosis of Parkinson's disease (PD) relies on the presence of specific motor symptoms, but cognitive decline is one of the most frequent non‐motor symptoms (up to six times more common than in healthy controls), often occurring in the early or even prodromal stage of the disease and significantly impacting the quality of life and increasing caregiver burden.^1^, ^2^, ^3^

The fronto‐striatal network‐based dysexecutive alterations represent the most predominant cognitive symptoms. The dorsolateral and medial prefrontal cortex provides top‐down regulation of attention, inhibition, and cognitive control through connections with the posterior cortex and subcortical structures which include the striatum (caudate, putamen, or ventral striatum), as well as the globus pallidus, substantia nigra, and thalamic nuclei.^4^, ^5^

Given the trajectory of the pathophysiological process that characterize PD and their early effects on fronto‐striatal circuits, it is predictable that patients with PD will develop attention/executive impairments that are dependent on these systems.

Studies show that PD patients experience a range of cognitive issues. These include non‐executive cognitive deficits, and the cognitive profile of PD varies in quality and severity. This spectrum of impairment can range from subjective cognitive decline (SCD) and mild cognitive impairment (PD‐MCI) to dementia (PDD). The progression of cognitive deficits and time to onset of dementia are also variable.^6^

To support clinicians, the International Parkinson and Movement Disorder Society (MDS) provides guidelines for assessing cognitive statuses, including recommendations for clinical outcome assessments (COAs).^7^, ^8^

Since these guidelines were published, several studies have focused on early identification of the PD‐MCI phenotype, with the primary objective of identifying specific cognitive profiles that are most indicative of progression to PDD.^9^, ^10^ However, the heterogeneous criteria used to define PD‐MCI, the unclear methodological parameters, and the lack of robust discussion of the clinimetric properties of the heterogenous tests/scales proposed (and the even wider range of tests used in studies), may have contributed to a poorly characterized PD‐MCI profile.^1^, ^6^

From a clinimetric perspective, a selected COA should possess good reliability, validity, and, for the neuropsychological tests, robust normative data. Furthermore, it should be sensitive to early, subtle alterations, capable of tracking change over time, and able to evaluate the effects of cognition‐enhancing treatments. It is important to recognize that the most suitable instrument(s) may vary depending on the purpose of the assessment. For instance, the tests best suited for detecting subtle deficits may not be the same as those most effective in measuring deficit progression, or the outcome of treatments.^11^

In this MDS‐commissioned review, the psychometric properties of the attention/working memory and executive tests (see an accompanying review on language, memory, and visuospatial functions) were investigated, following similar procedures employed in the “global scales” review for PD cognitive screening.^12^ These assessments have the potential to aid in the identification of PD‐MCI or PDD in clinical settings.

## 1 Methods

### 1.1 Organization and Review Process

An international group of experts on neuropsychological assessment in PD was selected by the MDS COA Program Scientific Evaluation Committee (SEC). The group focused on reviewing attention/working memory and executive COAs and was chaired by R.B.

A panel of 14 experts (O.B., D.M.C., B.C., J.C.D.‐A., N.E., E.F., E.H., S.M.‐H, A.M., G.S., B.S., C.S., A.T., G.J.G.) conducted a thorough review and evaluation of the measures assessing attention/working memory and executive functions in PD. Each assessment was carried out using a systematic procedure, with all evaluations documented in a template provided by the MDS COA program SEC, specifically tailored for the review of neuropsychological assessments.

Each review included a detailed description of the COAs, along with their properties, contemporary applications, psychometric properties, and an overall evaluation of the suitability and applicability within a clinical setting for PD patients. Initially, each scale or test was evaluated independently by two neuropsychologists, followed by an additional review by the chair of the group (R.B.). If there was disagreement, group discussion was convened to facilitate a consensus. The final decision was based on agreement of all expert panel members. The recommendation criteria were adopted from a previous review^12^ to include the categories “recommended,” “recommended with caveats,” “suggested,” and “listed.” Oversight of the entire project was provided by two liaisons (M.S., D.W.) who also reviewed the project. Finally, the manuscript was reviewed and approved by the MDS COA program SEC chairs (M.S., M.H.S.T.), COA program directors (T.A.M., A.S.F.), and the members of the MDS COA program SEC.

### 1.2 Literature Search

A literature search was conducted using PubMed, Web of Science, Medline, and Scopus for all publications from 1975 to December 2022. Keywords used in the search contained “Parkinson*” and the terms “cognit*” OR “test” OR “neuropsych*” OR “cognition” OR “cognitive deficits” OR “neuropsychological assessment” OR “cognitive testing” OR “neurocognitive” OR “neurocognitive assessment” OR “screening” OR “evaluation.” Tests accepted for the review were those included in published or in‐press peer‐reviewed articles with full text in English available to the expert members.

### 1.3 Selection of COAs

The review examined COAs that are part of the diagnostic criteria for PD‐MCI^8^ or that have been used at least once in PD research. It specifically addressed assessments of attention/working memory and executive functions. Individual measures were considered for inclusion if they are part of a multi‐test battery but have been independently used in PD for the cognitive functions pertinent to this review. Assessments undergoing re‐standardization, unstandardized tests, or those that are not commercially available were excluded from this review. Furthermore, computerized neuropsychological tests and assessments without an English version or with copyright issues were also excluded, as these may not be widely accessible in clinical settings.

### 1.4 Recommendation Levels

Each COA was categorized as follows: a test was “recommended” if (1) it has been applied to PD populations, (2) there are data on its use in studies beyond the group that developed the test, and (3) it has been studied clinimetrically in PD and found to be valid, reliable, and sensitive to change. “Recommended with caveats” indicates that the test's properties were generally found to be adequate, but some of the measurement properties were not evaluated specifically at different stages of cognitive impairment in PD. A COA was “suggested” if it had been applied to PD, but only one of the other criteria was met. A test is “listed” if it has been used in PD but does not meet the other two criteria defined for recommended tests.

## 2 Results

### 2.1 Identified COAs and their Use in Clinical Research

A total of 30 assessments of attention/working memory and executive function were identified, 16 of which are recommended for use in the MDS Task Force guidelines for PD‐MCI diagnostic criteria. After reaching a consensus, the expert panel recommended eight COAs: four assessing attention/working memory abilities and four evaluating executive functions. These COAs have shown good to excellent levels of reliability and validity, supported by normative datasets that span a wide age range and demonstrate sensitivity to change.

Moreover, eight COAs were designated as “recommended with caveats,” primarily due to inadequate psychometric quality or lack of sensitivity to change. Eleven additional COAs were classified as “suggested” level, and three were classified as “listed” (see Table 1).

**TABLE 1:** Overview of the clinical outcome assessments for each domain investigated

Attention/working memory domain	Executive domain
Recommended	
WAIS‐IV Digit Span WAIS‐IV Coding WAIS‐IV Symbol Search Trail Making Test	WAIS‐IV Similarities Wisconsin Card Sorting Test Verbal Fluency Tests Stroop Color‐Word Test
Recommended with caveat	
WAIS‐IV LNS SDMT Modified Levin's PASAT Corsi Block Tapping	Tower of London 10‐Point Clock Drawing Test Frontal Assessment Battery WAIS‐IV‐Matrix Reasoning Iowa Gambling Task
Suggested	
Digit Ordering Test Visual Search Test Brief Test of Attention TEA: Map Search/Visual Elevator Serial Reaction Time Task WMS‐III‐Digit Span	Behavioral Assessment of the Dysexecutive Syndrome Hayling Sentence Completion Test Brixton Spatial Anticipation Test Design Fluency Test
Listed	
Odd‐Man Out Test WSM‐III Mental Control	

Abbreviations: LNS, Letter Number Sequencing; PASAT, Paced Auditory Serial Addition Test; SDMT, Symbol Digit Modality Test; TEA, Test of Everyday Attention; WAIS‐IV, Wechsler Adult Intelligence Scale 4th edition; WMS‐III, Wechsler Memory Scale 3rd edition.

Comprehensive and detailed clinimetric properties regarding each COA are provided in Table 2 (only the recommended COAs) and in the Supplementary Materials (comprising a table detailing all the other COAs reviewed followed by all COA grids provided by reviewers). Namely, reliability (internal consistency, intra‐rater, inter‐rater and/or test–retest reliability); validity (including construct and empirical validity indices); sensitivity to change (from longitudinal studies or clinical trials); strengths and weaknesses; and level of recommendation and justification are provided.^12^

**TABLE 2:** Recommended neuropsychological tests including their psychometric properties

Scale/test	Reliability	Validity	Sensitive to change	Strengths	Clinimetric limitations	Recommendation level
Attention/working memory						
1. WAIS‐IV Coding	Good to excellent	Good	Yes	Strong normative data, based on a large sample Sensitive to subtle cognitive deficits in the early PD stages It is feasible and easy to administer: 5–10 min Translated into several languages Applicable to PD normal cognition, MCI, and early dementia Suitable for screening	Copyrighted Severe motor deficits (such as tremor, bradykinesia, or dyskinesia) can hamper its administrations as well as score interpretations No parallel forms available	Recommended
2. WAIS‐IV Digit Span	Good to excellent	Good	Yes	Strong normative data, based on a large sample Parallel form available It is feasible and easy to administer: 5–10 min Verbal administration suitable in the context of marked motor deficits Sensitive to subtle cognitive deficits in the early PD stages Applicable to PD normal cognition, MCI, and early to moderate dementia Translated into several languages Suitable for screening	Copyrighted Hearing deficits must be taken into account	Recommended
3. WAIS‐IV Symbol Search	Good	Good	Yes	Strong normative data, based on a large sample It is feasible and easy to administer: 3–4 min Applicable to PD normal cognition, MCI, and early dementia Translated into several languages	No parallel forms available Hearing‐visual and motor deficits must be taken into account Copyrighted Small literature in PD	Recommended
4. Trail Making Test (TMT)	Adequate to good	Good	Yes	It is feasible and easy to administer: 5–10 min Comparable forms are available Used a lot in many studies including lesion and MRI studies Many international groups use the TMT and have developed norm groups for determination of severity of impairment It is extensively used in PD Suitable for screening	Copyrighted Mostly not applicable for H&Y score: 4 (several disabilities or confined to bed) Floor effects in severe cognitive impaired patients Standard deviation is broad Determining change in individual cases is difficult due to broad RCIs Partially loading in different domains	Recommended
Executive domain						
1. WAIS‐IV‐Similarities	Good to excellent	Good	Yes	Strong normative data, based on a large sample Verbal administration suitable in the context of marked motor deficits It is feasible and easy to administer: 10–15 min Sensitive to subtle cognitive deficits in the early PD stages Applicable to PD normal cognition, MCI, and early dementia	Copyrighted Does not provide impairment‐only scaled scores Lack of validity studies specific for PD population Risk of possible floor/ceiling effects should be better investigated Hearing and language deficits must be taken into account No alternate forms available Scoring can be ambiguous Not suitable for screening	Recommended
2. SCWT‐VST version	Good test–retest reliability	Good criterion and convergent validity	Yes	Several comparable versions exist Public domains Translated and validated in several languages It is feasible and easy to administer: 5 min Good measure of executive functions Useful in PD population since a poor performance is a good predictor of conversion from PD‐normal cognition to PD‐MCI and to PDD	Not suitable for color blind people or patients with dyslexia, aphasia, hemianopsia, neglect Floor effect for severe cognitive deficits It may be difficult for patients with important dyskinesia, and it should not be administered in OFF condition Dopamine intake and motor severity affect performance Too many versions and too many scoring methods could make it difficult to choose which one to use	Recommended
3. WCST	Good to excellent	Good to excellent. More sensitive than specific. Good divergent validity also in PD	Yes	Several forms available, also PC based Can be used in PD with MMSE>19	Copyrighted It takes 20–30 min to administer for long version or 10–15 min for short version Dopamine intake and motor severity affect performance	Recommended
4. VFTs/COWAT	Good	Good content and criterion validity. Mixed construct validity: good within subtest, poor between subtest validity	Yes	Parallel/alternates forms available Translated in different languages Some versions are free on website It is easy and quick to administer (4–5 min) Good psychometric properties Applicable to PD normal cognition, MCI, and early dementia It is extensively used in PD	Standard deviation is broad Determining change in individual cases is difficult due to broad RCIs Loading partially on different domains	Recommended

Abbreviations: COWAT, Controlled Oral Word Association Test; H&Y, Hoehn and Yahr Scale; MCI, mild cognitive impairment; MMSE, Mini‐Mental State Examination; MRI, magnetic resonance imaging; PD, Parkinson's disease; PDD, Parkinson with dementia; RCI, Reliable Change Index; SCWT, Stroop Color‐Word Test; TMT, Trail Making Test; VFT, Verbal Fluency Test; VST, Victoria Stroop Test; WAIS‐IV, Wechsler Adult Intelligence Scale 4th edition; WCST, Wisconsin Card Sorting Test.

The following section provides a summary (test description, strengths, and weaknesses) of the recommended COAs.

### 2.2 Attention/Working Memory Domain

The Wechsler Adult Intelligence Scale, 4th edition (WAIS‐IV),^13^ was developed to evaluate intelligence and cognition in adolescents and adults aged 16–90 years, encompassing both normal and clinical populations, and has been translated into multiple languages. The subtests of the WAIS‐IV recommended in this review (Digit Span, Coding, and Symbol Search), have been used in the evaluation of PD, showing good to excellent reliability and validity. These subtests are supported by strong normative data based on large samples. Each subtest provides a scaled score, by converting the raw score into an age‐corrected scaled score, with a mean of 10 and a standard deviation of 3. These scaled scores can subsequently be converted into z‐scores and percentile ranks, assuming near normal score distributions. This allows for meaningful comparisons between the performance of the patient and the normative population, as well as among other subtests. Moreover, these subtests have demonstrated sensitivity to changes associated with PD and have been employed in research protocols within both PD and non‐PD populations. However, it is important to note that the WAIS‐IV is copyrighted (https://pearsonassessments.com/), and access to the full battery must be purchased to administer the subtests. In 2024, after the systematic review, the WAIS‐IV was superseded by the WAIS‐5,^14^ and was not yet independently used in PD. The WAIS‐5 incorporates several different working memory tests and has a modified factor structure compared with its predecessor.

#### 2.2.1 Digit Span Subtest

##### 2.2.1.1 Test description

The Digit Span Subtest is a core component of the WAIS‐IV Working Memory Index and is divided into three parts: Digit Span Forward (DSF), Digit Span Backward (DSB), and Digit Span Sequencing (DSS). It assesses auditory processing, attention, and encoding (mainly DSF), and auditory working memory and mental manipulation (DSB and DSS). DSB and DSS demand additional working memory load. In the DSF, the patient is asked to repeat digits in the order provided, whereas in the DSB subtest the digits must be recited in the reverse order. Finally, with DSS, patients are tasked with repeating the digits in ascending order. Each subtest starts with a relatively short string of digits, which is progressively extended with each successful trial until errors in recall are encountered. Each item is scored as either correct or incorrect, with higher scores indicating better cognitive performance. Estimates of working memory capacity may be derived from the total score across all subtests, reported as Working Memory Index, or the maximum span achieved within each subtest. Administration time is about 5–10 min.

##### 2.2.1.2 Strengths and Weaknesses

This subtest, particularly the DSB, has been used in PD research.^15^, ^16^, ^17^ It is demonstrated to be sensitive to change with ageing and PD‐MCI.^18^, ^19^ Outside of the PD field, it has been included in research related to MCI and dementia due to Alzheimer's disease (AD), and several other neurological disease.^20^, ^21^, ^22^, ^23^, ^24^, ^25^, ^26^ Research‐based alternate forms exist,^27^ and it is quick and easy to administer. Moreover, as it requires a verbal response, there is minimal motor component and it can be used across PD cognitive stages,^15^, ^28^ with this version reducing floor effects so it can be administered into the advanced stages of the disease. It is suitable for screening as it is sensitive to subtle cognitive deficits in the early PD stages. However, as subjects receive different numbers of trials the variance of the total correct score is high relative to the mean and it is highly skewed. Such high variance may reduce sensitivity to clinical abnormalities.^29^ Hearing deficits (for test instructions) and language skills must be taken into account during administration.

#### 2.2.2 Coding Subtest

##### 2.2.2.1 Test Description

The Coding Subtest is a core subtest of the WAIS‐IV Processing Speed Index.^10^, ^13^ In this subtest, individuals are asked to record associations between different symbols and numbers within time limits. It evaluates attention and executive domains, processing speed, visual‐motor coordination, and visual working memory. The administration time is 5–10 min, and the test is scored on a continuum based on the number of correct responses (maximum number of items = 135). Higher scores reflect better cognitive performance.

##### 2.2.2.2 Strengths and Weaknesses

It is easy and quick to administer and can be used across PD cognitive stages of disease.^30^, ^31^, ^32^ It is suitable for screening and is also demonstrated to be sensitive to change (either over time or due to treatment)^33^, ^34^, ^35^, ^36^ and is predictive of cognitive decline in PD and non‐PD.^36^, ^37^ Research‐based alternate forms exist.^38^ However, adequate vision, motor abilities, and language skills (sufficient to understand test instructions) must be present. As this subtest is timed and requires fine hand movements, severe motor disabilities associated with PD (such as tremor, bradykinesia, or dyskinesia) can hamper its administration and interpretation.

#### 2.2.3 Symbol Search Subtest

##### 2.2.3.1 Test Description

Symbol Search is also a core subtest of the WAIS‐IV Processing Speed Index.^13^ This subtest evaluates visual information processing speed as well as short‐term visual memory, visual‐motor coordination, cognitive flexibility, visual discrimination, attention, and concentration. During the subtest, the examinee is allowed 2 min to scan a group of items (ambiguous, geometric shapes) presented sequentially as a row, and indicate whether one of the symbols in the target group matches with those items. The overall administration time is about 3–4 min. The subtest is scored on a continuum based on the number of correct responses (maximum number of items 60) with higher scores indicating better cognitive performance.

##### 2.2.3.2 Strengths and Weaknesses

Symbol Search is easy to administer and can be used across PD cognitive stages. It has been shown to be sensitive to changes due to treatment,^33^ although there are no parallel forms available. There have been relatively few studies specific to the PD population. It is not suitable for screening. The potential floor/ceiling effects should be further investigated. Additionally, individuals must have adequate hearing (for test instructions), vision, motor abilities, and language skills (to understand test instructions). Severe motor disabilities, such as tremors, bradykinesia, or dyskinesia, can hinder the administration and interpretation of this subtest, as it is time‐based and requires precise hand movements.

#### 2.2.4 Trail Making Test (TMT)

##### 2.2.4.1 Test Description

The TMT was developed in 1938 by Partington and Leiter as a “distributed attention” test and published in 1949 as part of the Army Individual Test Battery.^39^ The test provides estimates of visual search, visual attention, speed of processing, and mental flexibility.^40^ TMT consists of two parts, A and B. In both parts, the examinee is required to connect spatially distributed target circles. In Part A, the target circles each contain a number, and the task is to link them in ascending order, from 1 to 25, as quickly as possible. In Part B the participant is asked to alternate between numbers and letters in ascending order and alphabetical sequence as quickly as possible. Part B is often regarded as an executive function measure due to rapid set shifting between numbers and letters, monitoring for speed of visual search, and elements of working memory. Performance indicators include the difference in time to completion and error scores between Parts A and B. Scores (ie, time in seconds, with lower scores representing better performance) can be compared with normative groups for determination of severity of impairment.^24^ Some authors include a time limit of 5 min on Part B. Administration time is about 5–10 min.

##### 2.2.4.2 Strengths and Weaknesses

The TMT is a widely recognized standard test in neuropsychological test batteries. It is relatively quick to administer. Poor performance is a good predictor of conversion to PD‐MCI and to PDD^41^ and it is suitable for screening. Normative data are available across several countries/languages and age and education groups.^40^, ^42^ However, it has no parallel versions but only comparable forms.^43^, ^44^ It is in the public domain, although copyrighted versions (https://www.neuropsych.com/) can be found. The risk of possible floor effects in more advanced dementia patients should be investigated. Moreover, as this test is timed and requires fine hand movements, severe motor disabilities (such as tremor, bradykinesia, or dyskinesia) can hamper its administration and interpretation in PD.

### 2.3 Executive Domain

#### 2.3.1 WAIS‐IV Similarities Subtest

##### 2.3.1.1 Test Description

The Similarities Subtest is included as a core test of the WAIS‐IV Verbal Comprehension Index. It evaluates both language abilities and executive functions, particularly verbal abstract reasoning and conceptualization. There is ongoing discussion about whether language or executive functions are more prominent in this assessment. For our classification, we have opted to categorize this test under executive functions. Similarities consists of 18 pairs of words. The examinee is asked to identify the qualitative relationship between the two words. Higher scores reflect better cognitive performance; each item is scored on an ordinal scale (0, 1, 2 points) based on the correctness of the response, and the instrument has a maximum score of 36. The test takes 10–15 min (including the instructions, the example, and items) and requires less time if the discontinuation criterion is met (ie, three consecutive failures).

##### 2.3.1.2 Strengths and Weaknesses

It can be administered across cognitive stages of the disease. It is commonly included in research related to AD, MCI, multiple sclerosis (MS), Huntington's disease, and others.^45^, ^46^, ^47^ It is suitable for screening as it is sensitive to subtle cognitive deficits in the early PD stages and to MCI status.^9^, ^11^ Additionally, it is sensitive to changes due to dopaminergic treatment.^33^, ^48^, ^49^ As it is verbally administered, and there are no motor or timed components, it is suitable for more advanced PD stages. It is relatively quick to administer. However, it has no parallel forms and there is a lack of validity studies in PD. It is not suitable for screening. Scoring can be ambiguous and thus potentially time‐consuming. The risk of possible floor effects in advanced dementia patients should be investigated. For more detailed clinimetric properties see the earlier WAIS‐IV Subtests section.

#### 2.3.2 Stroop Color‐Word Test

##### 2.3.2.1 Test Description

The Stroop Color‐Word Test was developed by John Ridley Stroop in 1935 and is used to measure interference effects in sequential verbal reactions.^50^ It evaluates mainly executive functions, in particular working memory, cognitive inhibition, and flexibility, and speed of visual search. The Stroop Test exists in several comparable versions.^43^, ^51^ The Victoria version, called the Victoria Stroop Test (VST), is a shorter version of the original test (30 items for each condition compared with 100 items) and a psychometrically‐sound version of Stroop's original task.^52^, ^53^ VST is the most used in PD patients and thus is assessed in this review. It takes approximately 3–5 min to administer. The VST includes three cards presented in a fixed order: color dot naming (D), word reading (W), both as control tasks, and incongruent color‐naming of color words (C) (such as the word red printed in green ink), as interference task. The test assesses response inhibition using two measures: Interference Effect (IE) and Error Score (ES). IE is a reaction time score whilst ES is the number of errors. These measures in each card are recorded, and the time difference and error between cards C and D is calculated. In some versions each score is obtained by subtracting the mean score of the two “control” tasks (reading neutral words and colored dots naming) from the interference task score, which is predicted by both conflict monitoring and working memory; one can also calculate the ratio score (color‐word divided by color‐naming) (predicted by conflict monitoring alone).^51^, ^54^

##### 2.3.2.2 Strengths and Weaknesses

The Stroop Test is widely utilized in diagnosing and researching executive functions due to its ease of administration and diagnostic importance.^50^ The Stroop Test has been translated and validated in numerous languages with normative data available for a wide age range group (<20 to >94 years)^52^, ^53^, ^55^, ^56^, ^57^, ^58^ and is highly sensitive in differentiating several neurological diseases from the normal population.^59^, ^60^, ^61^, ^62^ Poor performance is a good predictor of conversion to PD‐MCI and PDD.^8^, ^28^, ^45^, ^58^, ^63^, ^64^ It is also suitable for screening and is sensitive to change due to treatment in PD.^49^, ^65^, ^66^ The VST is in the public domain, and users may make their stimuli (eg, Prague Stroop test)^58^ or purchase them from the University of Victoria, although at least one version has been copyrighted (https://www.parinc.com). In addition, adequate psychometric data, including reliability and validity, have been obtained for the VST.^40^ There are no parallel versions and many not completely comparable versions and scoring methods. Gaze palsy can hamper its administration. It may be difficult for patients with troublesome dyskinesia. Moreover, it is not suitable for people with certain types of color blindness or patients with dyslexia, aphasia, hemianopsia, severe hypokinetic dysarthria, and neglect. Finally, floor effects in advanced dementia patients are observed.

#### 2.3.3 Wisconsin Card Sorting Test (WCST)

##### 2.3.3.1 Test Description

The WCST was developed in 1948 to assess perseveration, abstract reasoning, and set‐shifting in normal adult populations.^67^ It is now generally used to assess clinical populations.^68^ This test is often viewed as the “gold standard” for examining executive function in terms of cognitive flexibility and attention/task switching, especially through the number of perseverative errors—that is, category repetitions in response to negative feedback.^69^ The test also gauges strategic planning, working memory, response inhibition, and impulsive responses.^70^ There is evidence that the test reflects both automatic stimulus–response learning as well as higher‐order concept/category formation/learning.^71^ During the test, participants are presented with four multidimensional stimulus cards featuring different colors, shapes, and numbers, and are required to sort the cards based on an undisclosed rule. There are two versions of the tests: a longer (20–30 min) version (WCST‐128) and a short version (WCST‐64).^72^ Scores on the two versions are generally similar but may not be identical.^73^ A modified version^74^ with 48 deck cards also exists aiming to minimize participant frustration; at least 10 scores can be generated by the standard WCST, but a measure of perseveration (errors) and total categories achieved are generally the primary measures used.^75^ Normative data are utilized to assess impairment and severity.

##### 2.3.3.2 Strengths and Weaknesses

The WCST is widely used to assess executive function.^76^ It has been translated into numerous languages with normative data available for a wide age range group (6–89 years).^77^, ^78^, ^79^, ^80^, ^81^, ^82^ The WCST has been utilized in various neurological patient groups and neurodevelopmental disorders.^75^, ^83^ The WCST has been applied in a range of clinical and research applications for PD,^75^ including those with PD‐MCI, mild Lewy body dementia patients (based on the MMSE >19).^84^ While evidence in PD is limited, the WCST has shown sensitivity to treatment‐induced changes.^85^, ^86^ It is suitable for screening. Multiple forms of the test are available, including a computer‐based version. However, it is copyrighted. Additionally, the WCST‐128 requires long administration and can be impacted by PD medication use and motor severity.

#### 2.3.4 Verbal Fluency Test (VFT)

##### 2.3.4.1 Test Description

The VFT, also known as the Controlled Oral Word Association Test (COWAT), the Controlled Oral Word Association (COWA), the Word Fluency, the Letter Fluency, the FAS‐Test, the Category Fluency, the Phonemic Fluency, the Semantic Fluency, Thurstone Word Fluency Test, and so on, is a widely‐used neuropsychological tool developed by Thurstone (1938).^87^ It is considered a classical tool for neuropsychological assessment,^88^ and explores various domains such as word production, verbal fluency, word search, semantic memory, mental lexicon, mental flexibility, and retrieval from semantic memory. The test evaluates an individual's ability to generate as many words as possible within a specified time frame, either from a given letter of the alphabet (eg, English FAS; Dutch DAT, KOM, or PGR; Spanish PMR) or from a semantic category (eg, animal, fruit, color, vegetables, supermarket items) within a 1‐min period. Less frequently used fluency tests involve action verb generation, writing, or design tasks. Number of correct words is calculated. A more detailed scoring of the individual's performance such as perseverations, stuck in a set, intrusions, paraphasias, spelling errors, clustering, and switching have been proposed as valuable sources of information.^40^, ^52^ Normative data are used to measure impairment and severity.

##### 2.3.4.2 Strengths and Weaknesses

It is easy to administer. It is useful in PD populations since it shows high sensitivity across all PD cognitive statuses.^9^, ^88^ There are several versions of the tests. It has been translated into numerous languages, and normative data are provided for a wide age range (6–89 years).^40^ The VFT is useful for screening, and it is very sensitive to change over time due to the progression of the disease and due to interventions, such as deep brain stimulation (DBS).^89^, ^90^, ^91^ However, depending on which version is used, it can be free of charge, or be part of copyrighted batteries,^92^, ^93^ or require purchase from PAR (https://www.parinc.com/). Moreover, increasing evidence has made clear that cultural, linguistic, and sociodemographic factors influence performance on verbal fluency.^40^, ^94^, ^95^, ^96^, ^97^ Finally, hypokinetic dysarthria severity should be considered when interpreting VFT performance in PD.^98^

## 3 Discussion and Recommendations

This review provides critique and recommendations of COAs that assess attention/working memory and executive measures in PD. The MDS COA Program SEC commissioned a subcommittee comprising a panel of 14 expert neuropsychologists to investigate the attention/working memory and executive measures used in PD, namely the COAs whose psychometric properties could better contribute to cognitive diagnostic accuracy. Namely, from a plethora of 30 COAs, 8 tests were recommended according to the guidelines adopted in the review,^12^ including 4 tests assessing mainly attention/working memory abilities and 4 tests assessing mainly executive functions (see Table 1).

### 3.1 Attention/Working Memory Domain

Overall, three of four recommended attention/working memory tests (Digit Span, Coding, and Symbol Search), are part of the WAIS‐IV Processing Speed and Attention/Working Memory indices, a scale with excellent clinimetric properties including normative data with an upper age limit to the 90s and several language translations. In PD, the WAIS‐IV verbal span tasks, including Digit Span and Letter Number Sequences WAIS‐IV Subtest (which reached the “recommended with caveats” level), offer a potentially straightforward and quick assessment of baseline attention/working memory abilities. Evidence^28^ shows that Digit Span Backward successfully distinguishes PD cognitive statuses (regardless of whether ON or OFF medications). Moreover, the Digit Spans involve minimal training, and do not necessitate good motor abilities, whilst the other three recommended tests (Coding, Symbol Search, and TMT) require additional cognitive functions (eg, visual–motor coordination, cognitive flexibility, visual discrimination, attention, and concentration), are time‐based, and require fine hand movements possibly hampering administration and making interpretation difficult if motor dysfunction is present. In general, these issues are quite common in several tests assessing attention/working memory domain in PD (eg, the Symbol Digit Modality Test [SDMT], which reached the “recommended with caveats” level) despite otherwise adequate clinimetric properties. Future research should be directed at developing more attention/working memory tests that minimize the role of the aforementioned limitations.

### 3.2 Executive Domain

Executive dysfunction (EF) is perhaps one of the most frequently present cognitive impairment in PD.^99^ Significant impairment in the VFT (semantic, phonemic, and alternating), various measures of the WCST and the Stroop Test (all recommended tests in this review), are confirmed, by meta‐analysis and reviews, being very sensitive in detecting impairment in PD, relative to age‐matched HC.^100^, ^101^ However, in movement disorders research, a major limitation in the accurate assessment of those abilities is the severity of motor impairment. For example, although altered WCST performance can be considered a well‐established neuropsychological symptom in patients with PD, as it is present in non‐demented, non‐medicated, and non‐depressed PD, it may also be linked to the severity of patients' motor symptoms.^75^ Similarly, Stroop task performances change as a function of two markers of disease severity (ie, disease duration and levodopa medications), supporting the concept that declining test performance may be associated with more severe motor symptoms. Of note, the version used (pen and paper vs. computerized), the influence of multiple domains involved, and the wider brain areas implicated, which may be only partially sensitive to PD medications,^102^ may contribute to contradictory findings.^103^, ^104^

Interestingly, it has been proposed that motor severity constitutes a confounding variable during neuropsychological testing, likely affecting performance, rather than resulting entirely from underlying neuropathological changes to dopaminergic systems.^105^, ^106^ Further, understanding the complex impact of motor symptom therapies in PD is highly relevant, as it may differentially affect performance on cognition.^102^ Future research should be considered in this regard.

Conversely, the verbally administered fluency tests and the Similarities subtest of the WAIS‐IV should be considered when exploring executive abilities in advanced PD stages or when motor complications are present. Specifically, the fluency tests, despite their simple and quick administration, provide the clinician with valuable information on PD cognitive status,^107^, ^108^ as they are among the earliest cognitive changes in the disease and have low floor effects in advanced cognitive stages.^8^ Moreover, reduced verbal fluency is a potential risk factor for the development of PDD.^9^, ^109^, ^110^ Of note, unlike verbal fluency, Similarities subtest scoring may be highly variable due to the examiner judgement required for scoring. Furthermore, floor effects can be observed in advanced dementia patients.

Finally, because of the influential role of sociodemographic factors (eg, previous occupation, socioeconomic level, education, and premorbid intelligence quotient [IQ]) on attentional‐executive functions test scores,^111^, ^112^ cognitive measures with normative data correcting for demographic factors may be preferred as part of a valid cognitive battery.

This review has some limitations. First, we did not include the domain of social cognition. Although evidence recognizes the role of social cognition as a possible early marker of cognitive decline,^113^ the lack of standardized instruments and the relatively recent focus on this area have prevented their inclusion in our current review. A future review is needed once the conceptual framework and measurement methods are more developed. Second, although digital versions have been developed for some of the included scales/tests, which may appear similar to the original paper‐and‐pencil formats, we decided to exclude them *a priori*, as they need thorough validation before they can be used appropriately (ie, reaching the appropriate level for critique and recommendations). Third, journal readers should consider that many of the recommended tests (eg, TMT, Stroop Color‐Word Test‐) require involvement of multiple cognitive domains with lack of expert consensus on how best to categorize them. For this review, we decided to list the main domain assessed by the test/scale. A fourth limitation in neuropsychology is the lack of culturally specific and culturally sensitive assessments for PD and more broadly. Most tests are designed for educated populations in Western countries, which can significantly affect performance due to variations in culture, language, education, and literacy. Individuals from non‐Western cultures are often at a disadvantage, leading to misidentification of cognitive abilities. This can result in impairment being overlooked and preserved functions being mistakenly regarded as deficits. Consequently, these individuals may not receive personalized and effective treatment plans that cater to their unique needs, emphasizing the need for culturally appropriate neuropsychological assessments.^114^

## 4 Conclusions

Understanding the nature and extent of executive/attentive dysfunctions in PD is of critical importance, as its presence in PD is related to reduced patient and care partner quality of life^115^ and predicts progression to PDD.^64^, ^116^, ^117^, ^118^, ^119^ Moreover, evidence indicates that all forms of PD interventions (ie, pharmacotherapy, exercise/physical therapy, and DBS) appear to impact fronto‐striatal functioning.^120^ As such, clinimetric investigation of the neuropsychological tools most commonly proposed for their assessment is crucial for a broader understanding of the role of EF in PD.

Similar to the previous MDS review on global scales for cognitive screening,^12^ in this review an expert international panel selected the recommended attention/working memory and executive tests to guide the assessment of fronto‐striatal functioning in PD at different stages (including PD‐MCI or PDD). These recommended neuropsychological tests with high psychometric qualities will enable clinicians to increase PD cognitive diagnostic accuracy and facilitate research to fill the gaps still present in this topic.

## Author Roles

(1) Research Project: A. Conception, B. Design, C. Execution, D. Analysis; (2) Statistical Analysis: A. Design, B. Execution, C. Review and Critique; (3) Manuscript Preparation: A. Writing of the First Draft, B. Review and Revision.

R.B.: 1A, 1B, 1C, 1D, 3A, 3B.

O.B.: 1A, 1B, 1C, 1D, 3A.

D.M.C.: 1B, 1C, 1D, 3B.

B.C.: 1B, 1C, 1D, 3B.

J.C.D.‐A.: 1B, 1C, 1D, 3B.

N.E.: 1B, 1C, 1D, 3B.

E.F.: 1B, 1C, 1D, 3B.

E.H.: 1B, 1C, 1D, 3B.

S.M.‐H.: 1B, 1C, 1D, 3B.

A.M.: 1B, 1C, 1D, 3B.

G.S.: 1B, 1C, 1D, 3B.

B.S.: 1B, 1C, 1D, 3B.

C.S.: 1B, 1C, 1D, 3B.

A.T.: 1B, 1C, 1D, 3B.

T.A.M.: 1C, 3B.

Á.S.F.: 1C, 3B.

M.H.S.T.: 1C, 3B.

M.S.: 1A, 1B, 1C, 1D, 3A, 3B.

D.W.: 1A, 1B, 1C, 1D, 3A, 3B.

G.J.G.: 1A, 1B, 1C, 1D, 3A, 3B.

## Financial Disclosures of All Authors (for the Previous 12 Months)

None.

## Supporting information


Data S1.

## Data Availability

The data that supports the findings of this study are available in the supplementary material of this article.
