# Supplementary material for: Attention/Working Memory and Executive Function in Parkinson's Disease: Review, Critique, and Recommendations
Source: Mov Disord. 2025 Jul 18;40(9):1791–804. doi: 10.1002/mds.30293 (PMC12485586; doi:10.1002/mds.30293)
Supplement: Supplementary file 1 — Data S1. [file MDS-40-1791-s001.docx]

**Supplementary Table**. Recommended with caveats, Suggested and Listed neuropsychological tests including their psychometric properties.

| **Scales/tests** | Reliability | Validity | Sensitive to change | Strengths | Clinimetric limitations | Recommendation level |
| --- | --- | --- | --- | --- | --- | --- |
| **Attention/working memory** |  |  |  |  |  |  |
| WAIS-IV-LNS | Good to Excellent | Good | Mixed results | - Strong normative data, based on a large sample (for ages up to 69 ys). - Sensitive to subtle cognitive deficits in early PD-stages. - Verbal administration suitable in the context of marked motor deficits. - It is feasible and easy to administer: 5-10 minutes. - Translated in several languages. - Applicable to PD normal cognition, MCI and early dementia | - Copyrighted. - The risk of possible floor/ceiling effects should be better investigated. - Hearing deficits must be taken into account. - No alternate forms available. - No reliability/validity/norms data for patients over age 69 provided in the WAIS-IV manual. - Not suitable for screening. - For severe cognitive deficits, floor effect and frustration. | Recommended with caveats. |
| SDMT | Good | Good | Mixed results | - Parallel forms available. - Translated and validated in several languages. - Does not require literacy. - Quick and easy to administer (90 seconds) - Normative data for written and oral versions are available. - Well recognized amongst neurodegenerative diseases and extensively used in PD. - Applicable to PD normal cognition, MCI | - Copyrighted. - Number of errors not used as an outcome. - Hearing-visual / visual scanning and motor deficits must be taken into account. - Written and oral substitution tasks require the integration of several complex cognitive abilities involved in visual, motor, speech and mental functions. | Recommended with caveats. |
| The modified Levin’s PASAT | Reliability is high, but stability (test-retest consistency) is low due to practice effects | Good. | Mixed results | - Public domain - Highly sensitive test in early-stage PD. - Parallel forms available - Translated in Italian - Applicable to PD normal cognition, MCI | - Practice effect. - Chunking strategies may ameliorate performance. - For severe cognitive deficits, floor effect and frustration. - Normative data not suitable for PD. - It takes 15-20 minutes to complete. | Recommended with caveats. |
| Corsi block tapping | Acceptable/good | Good | Mixed results | - Parallel forms available, also PC based. - Translated in several language. - It scores impairment. - Public domain. - Easy and quick to administer (5-10 minutes). - Relatively simple, widely use, suitable for remote (web-based) testing. | - Methodological inconsistencies across versions have been identified. - A widely accepted version remains to be adopted. - More work is needed to determine whether a Corsi test would usefully identify prediction of conversion to dementia in PD. | Recommended with caveats. |
| Digit ordering test | Good general internal consistency. Internal consistency and split half reliability are questionable in patients with PD. | Good. The component of dimensionality, variability and dependency structure has not been analysed. | N/A | - The DOT and DOT-A appear to discriminate between PD and healthy controls, as well mild PD vs. severe PD. - The measure is easy to administer, similar to Digit Span. - It is more sensitive than Digit Span backwards to impairment in verbal executive functions in PD. - It was developed specifically to understand verbal working memory in PD. - Public domain. | - Not suitable for hearing problem. - Sample sizes in psychometric studies have been relatively small. - Lack of references about the version used in the paper. - There are questions about test-retest reliability. | Suggested. |
| Visual Search Test/Digit cancellation Task | Poor | Poor | N/A | - Public domain. - Each task has digits only, therefore it is theoretically suitable for any language where Arabic number system is in use. - Easy and quick to administer: 3-4 minutes. - Sensitive to dementia also in PD | - Do not provide impairment or severity. - Good visual abilities needed. - Not widely used. - few studies in PD. - Not really studied from a psychometric point of view. | Suggested. |
| Brief Test of Attention | Test-retest reliability: low to adequate”. Internal consistency: Good | Good. | Mixed results, limited info from literature | - Bedside, short screening test. - It is suitable for individuals with visual and/or motor impairments. - Easy and quick to administer: 8 minutes | - Literacy is needed. - Limited to auditory divided attention. - Only screen for deficits. - No normal function evaluation. - More research is needed on test stability over time. - Translations and validations only English, Spanish and Portuguese. - Cut-off score in relation to the diagnostic unit (MCI/dementia) was not found. | Suggested. |
| TEA: Map search | Good for the original version. Mixed for the other forms. Limited info for PD | Good | Not good. | - Three parallel forms. - It is feasible and easy to administer: 1-2 minutes. - It measures visual selective attention without any motor component (although there is a speeded component). - TEA are based on everyday materials. - Together with TMT-B and CVLT-II SF total immediate recall, Map Search first minute predicts conversion to PDD from PD-MCI | - Copyrighted. - Sensory problems play a role in not completing tests. - Reported ceiling effect (<50ys). - It takes 45-60 minutes to complete the TEA. | Suggested. |
| TEA: Visual elevator | Good for the original version. Mixed for the other forms. Limited info for PD | Good | Not good. | - Three parallel forms. - It can be used in pathology and normal subjects. - Sensitive enough to show normal age effects in the normal population. - Scaled scores are provided. - Practice effects can be controlled. - TEA are based on everyday materials. | - Floor effect (>80ys). - Sensory problems play a role in not completing tests. - It takes 45-60 minutes to complete the TEA. | Suggested. |
| Serial reaction time task | Poor | Good | Mix results | - Public domain - It is a visuospatial task. No translation is needed | - It is an experimental paradigm and no cut-off score, or severity index have been found. - Lack of psychometric info. | Suggested. |
| WMS-III-Digit Span | Adequate to good | Good | N/A | - Parallel forms available. - It assesses both impairment and severity. - Age-education corrected normative data available in several languages. - It is a well-recognized instrument. - It is feasible and easy to administer (5 minutes). | - Copyrighted. - Hearing deficits must be taken into account. - Does not appear as the best to be used in the context of PD for the purpose of measurement of attention. | Suggested. |
| Odd-Man Out test | N/A | N/A | N/A | - Two parallel versions. - Less challenging and more pure measure of abstraction and shifting than the WCST. - Public domain. | - It takes around 10-15 minutes. - The test needs to be developed by researchers. - Lack of a standardized test with access to instructions, test material, administration norms. | Listed. |
| WMS-III-Mental Control (optional subtest) | Good (for ages 55-89) | N/A | Yes | - It is feasible and easy to administer: 3-4 minutes. - Reported good reliability (although not assessed as carefully as the primary WMS-III subtests). - Translated into multiple languages. | - May be difficult to purchase or obtain permission to use due to being out of print and lack of inclusion in the current WMS-IV. - Not included in the WMS-III validity studies. - Rarely used in PD research, validity and sensitivity to change in this population is largely unknown. | Listed. |
| **Executive** |  |  |  |  |  |  |
| Tower of London (the original manual version) | Mixed results according to the version. Poor for the original one. Good for the TOL-F version. | Mixed results. Good for TOLDX | Yes | - Parallel forms available, also PC based. - The original version is free.   It is sensitive to planning deficit in PD patients. | - Not suitable for colour blinded people, patients with severe apraxia or paraplegia or severe motor deficits (i.e., dyskinesias). - It takes 15-20 minutes for administration. - For severe cognitive deficits, floor effect and frustration.   Do not provide impairment or severity. | Recommended with caveats. |
| 10-point Clock drawing | Good | Moderate | Yes | - Parallel forms available. - Translated in different languages. - Public domain. - Easy and quick to administer (2-4 mis). - Used in PD population and included among the best cognitive test to diagnose PD-MCI (Goldman et al., 2015). - Suitable for differential diagnosis (PDD/DLB). | - It assesses multiple domains. - Not suitable for patients with severe apraxia, paraplegia or severe movement disorders (dyskinesias). - Mostly not applicable for H&Y score: 4 (several disabilities or confined to bed). - Does not provide severity score. | Recommended with caveats. |
| WAIS-IV-Matrix Reasoning | Good to excellent | Good | Not good | - Strong normative data, based on a large sample (for ages up to 69 ys). - Translated in several languages. - It is easy to administer. - Applicable to PD normal cognition, MCI and early dementia | - Copyrighted. - No alternate forms available. - Small literature in PD. | Recommended with caveats. |
| FAB | Overall Good. Good internal consistency in PD. | Good in PD | N/A | - Public domain. - Easy and quick to administer (5-10 minutes). - Translated in different languages. | - Not parallel versions. - Good motor functions are needed for some subtests. | Recommended with caveats. |
| Iowa Gambling Task | Questionable | Good but only construct validity had been investigated in PD | Good | - Easy and quick to administer (5-10 minutes). - It is not affected by dopaminergic level, disease duration, severity of motor symptoms. - It scores impairment. - Some versions are in public domain. | - Practice effect.ect - Performance could be influenced by the frequency of losses rather than the long-term outcomes resulting in poor performance and complicating the interpretation of the data. - Good and visual abilities needed. | Recommended with caveats. |
| Behavioural Assessment of the Dysexecutive Syndrome | Only partially assessed in PD: good inter-rater reliability, moderate test-retest reliability | Overall good to moderate. In PD good concurrent validity, Moderate convergent validity | N/A | - The battery focuses on revealing dysexecutive syndromes which are part of PD. - The measure has been used in PD and in a variety of other disorders affecting frontal systems. - Translated in different language. | - Copyrighted. - No parallel versions. - It takes 40 minutes to complete. - The clinicometric properties of the battery are poorly evaluated and deserve to be investigated. | Suggested. |
| Hayling Sentence Completion  Test | Low to good | Moderate | N/A | - Easy and quick to administer (5 minutes). - Entirely spoken therefore suitable for patients with reading and movement impairment. - Well recognized. - Translated in different languages. - It assesses severity. - Applicable to PD normal cognition, MCI. | - Copyrighted. - No evidence for inter-rater reliability despite significant judgment required for scoring. - Variable concurrent validity. - Modest normative sample or lack of validation studies in other languages. - No parallel forms. - Risk of ceiling effect and floor effect. | Suggested. |
| Brixton Spatial Anticipation Test | Questionable | Mixed Criterion validity.  Overall mixed construct validity-not good in PD | N/A | - Easy and quick to administer (5 minutes). - It can be administered also in bed. - It does not require a verbal or complex motor response by patients, making suitable also for being used with PD patients H&Y > 4. | - It has not been studied clinimetrically in PD. - Clinimetric data not generally good. | Suggested. |
| Design Fluency Test | Good | Good | N/A | - Parallel forms available - It scores impairment and severity. - It allows to account the motor component during task execution. - Translated in different languages. | - Copyrighted. - Good motor and visual abilities needed. - Absence of reliable normative data in PD and subgroups. - No specific validation studies in PD.   Extremely expensive. | Suggested. |

*Note*. WAIS-IV: Wechsler Adult Intelligence Scale 4° edition; LNS: Letter number sequencing task; SDMT: Symbol Digit Modality test; TOL-F: Tower of London-Freiburg version; TOLDX; T Tower of London -Drexel PASAT: Paced Auditory Serial Addition Test; FAB: Frontal Assessment Battery; WMS-III: Wechsler Memory Scale 3° edition; TEA: Test of Everyday Attention; TMT-B: Trail Making Test part B; CVLT-II SF: California Verbal Learning Test; SCWT: Stroop Colour and Word test. PD: Parkinson’s Disease; MCI: Mild Cognitive Impairment; PDD: Parkinson with dementia; H&Y: Hoehn and Yahr Scale; N/A = evidence not available.

**Supplementary grids with all COAs’ details per domains.**

Attention/working memory domain: Recommended Tests/subtests.

**Rating test evaluation**

| **Test name: Coding subtest from WAIS-IV** | Responses |
| --- | --- |
| **Version**  Are there several versions (alternate or parallel) of the test? If so, which one has been assessed? | Coding is a subtest of the Wechsler Adult Intelligence Scale, 4^th^ edition (WAIS-IV) (1, 2), with previous versions included in the WAIS-III and WAIS-R (Digit Symbol). These prior versions are still frequently used in research. There are no alternate/parallel versions available for the WAIS-IV, although research-based forms exist (3). |

| **Description of test**   - What population the test was originally developed for? - Which domains/components of domains do the test assess? - Are there multiple domains or a predominant domain assessed? - Does it score impaired/unimpaired? - Does it score severity   (subtle/mild/moderate/severe)? | - Subtest from the WAIS-IV, which was developed to evaluate intelligence and cognition in adolescents and adults (16 to 90 years) in both cognitively normal and clinical populations. - The Coding subtest evaluates primarily information processing speed (visual-motor coordination, motor and mental speed, visual working memory). - Does not explicitly score impaired/unimpaired. - Score is based on severity since higher scores reflect better cognitive performance. The manual provides scaled scores, which can be converted into *z* scores and percentile ranks, allowing to compare the performance with the normative population. Regarding the ‘severity qualitative descriptors’, there are some controversies based on the applied validation/ standardization.[[1]](#footnote-1) |
| --- | --- |
| **Scaling Metric**: Type of response format? (e.g., Likert, Binary, multiple-choice, continuum, reaction time, etc.) | The test is scored on a continuum based on number of correct responses. Raw score (sum of correct responses), which can be converted to an age-corrected scaled score (*M* = 10; *SD* = 3) |
| **Respondent: Patient:** any specific requirements for the target study population (e.g. non-demented, motor, auditory, visual abilities, etc)? | Adequate hearing (for test instructions), vision, motor abilities and language skills (sufficient to understand test instructions). Given this subtest is time-based and requires fine hand-movements, patients with severe motor disabilities can appear more impaired due to motor deficits rather than cognitive abilities. Clinical judgment of the rater is required. |
| **Access:** How can the test be obtained (mail address or website)? | Pearson serves most of the international service orders and products ([pearsonassessments.com](file:///Users/bchol/Library/Containers/com.apple.mail/Data/Library/Mail%20Downloads/D768BDAD-F31A-4632-A8FB-D6439D83DF7B/pearsonassessments.com)). However, some countries have to refer to different publishers (i.e., Italy: [giuntipsy.it](file:///Users/bchol/Library/Containers/com.apple.mail/Data/Library/Mail%20Downloads/D768BDAD-F31A-4632-A8FB-D6439D83DF7B/giuntipsy.it)) |
| Copyrighted or in public domain?  Conditions for use? Fee? | Copyrighted; the full WAIS-IV must be purchased. The fee is quite expensive ($ 1,500/ € 2,000). |
| **Translations:** Has the test been translated and validated in other languages besides English? | Yes. The WAIS-IV has been translated in several languages[[2]](#footnote-2): English (US), English (Australia and New Zealand), English (Canada), English (UK), Chinese, Danish, Dutch, Finnish, French (Canada), French (France), German, Greek, Hebrew, Hungarian, Icelandic, Italian, Japanese, Korean, Lithuanian, Mandarin (Taiwan), Norwegian, Polish, Portuguese (Brazil), Spanish (Argentina), Spanish (Chile), Spanish (Mexico), Spanish (Spain), Swedish as well as Indonesian.  Some versions are translated, language adapted, and/or have local normative data. |

| **Test properties**  (Please provide references for all statements or note if this is your judgment) | |
| --- | --- |
| Which cognitive domain (or component/specific aspect of a cognitive domain) does this test *primarily* measure? | Attention and executive function domains, with the involvement of processing speed processes. This is a core subtest of the WAIS-IV Processing Speed Index. |
| Which aspects of the domain you are assessing are not covered? | Planning, abstraction, and conceptualization. |

| **Use** |  |
| --- | --- |
| Was it *designed* to measure severity, screen or diagnosis of the domain? | Designed to measure severity of processing speed deficits rather than for screening or diagnosis. If the raw score is converted into the scaled score, this allows comparison of the individual performance to the normative population (in terms of *z-*scores/percentile ranks). |
| Is there a cut-off score? Are there measures of screening/diagnostic performance of the test? (Receiver characteristic curve analyses; sensitivity/specificity; Positive Predictive Value/Negative Predictive Value), True Positive Rate, False Positive Rate etc.) | The test manual (2) does not provide cut-off scores; rather, age-corrected scaled scores can be converted to *z-*scores/percentiles. |
| Is this appropriate for the PD population? If not, why (low sensitivity and/or specificity values)? | Yes.  However, if *severe motor deficits* are observed, the rater’s clinical judgement will be necessary to assess whether they interfere with the task performance. |
| **Clinimetric/psychometric properties in the target population** | |
| **Feasibility /Acceptability** |  |
| Length | Number of items: maximum 135  Maximum time permitted in minutes: 2 min  Plus 2-4 minutes for the instructions and the example items. |
| Ambiguities in instructions to patient | No |
| Ambiguities in rating anchors | No |
| Appropriateness of questions for population | Yes |
| Applicability across disease cognitive stages | Normal cognition, MCI and early dementia (4-6) |
| Applicability across disease motor severity? | H&Y 1-3, depending on severity of tremor or other motor deficits |
| Clear instructions to raters | Standard instructions for administration and scoring are included in the test manual. |
| Has the test been used by researcher other than developers?  By other groups  Not applicable? | Yes, widely used in PD and non-PD research protocols |
| Are there instructions for dealing with missing data?  Not applicable? | Correct administration of the test requires completion of test items in a specific order without skipping any, thus avoiding missing answers. Items not completed within the time limit are scored as 0 rather than missing data. |
| Floor and ceiling effects, skewed score distributions? | Normalized distribution |

| **Have the component of dimensionality, variability and dependency structures been analyzed?**  (Exploratory and confirmatory factor analysis (EFA and CFA)? | Yes.[[3]](#footnote-3) CFA confirms loading on processing speed index, with **Coding** loading 0.97 on the Processing Speed factor (2, 7) |
| --- | --- |
|  |  |
| **Reliability: assessed – good; not good. Not assessed**  Are alternative versions developed?  If so, were they validated with reliability measures? | Assessed – good, reported in the WAIS-IV Technical and Interpretive Manual (2)  No alternate forms available |
| Internal consistency (A rule of thumb) (8)  0.9 ≤ Cronbach’s alpha (α) Excellent  0.8 ≤ α < 0.9 Good  0.7 ≤ α < 0.8 Acceptable  0.6 ≤ α < 0.7 Questionable  0.5 ≤ α < 0.6 Poor  α < 0.5 Unacceptable | Not calculated for Coding, as split-half and Cronbach are not appropriate indexes for timed tests. Reliability is based on test-retest (see below). |
| “Corrected item-to-total correlation” | No |
| Test/retest | Yes, short-term test-retest stability was investigated for 298 individuals from four age groups with retest intervals ranging from 8–82 days (mean retest interval of 22 days)  Average across age-ranges: 0.83 (0.86 after correcting for variability of the normative sample). .89 for ages 55-69, .86 for ages 70-90 (corrected)(2) |
| Inter-rater | Excellent. All WAIS-IV subtests are .98 to .99 (2) |
| Intra-rater | No |
| Other (e.g. Standard Error of Measurement) | Yes, average across age-ranges: 1.05 – 1.13 (2, 7) |
| **Validity: assessed –good; not good. Not assessed (reference)** |  |
| Face and Content validity (does test appears to be measuring what it is intended to measure and do test’s items comprehensively represent the domain of interest?) | Good: WAIS-IV Technical and Interpretive Manual provides a discussion of content validity as determined by comprehensive literature and expert reviews (2). |
| Criterion validity (compared to gold-standard) or other criteria for  diagnosis) (e.g. other comparable tests or lesion or MRI research) | Compared to previous Wechsler Adult Intelligence Scales and Intelligence Scale for Children. |
| Construct validity (correlations with other convergent tests and divergent tests; known-groups comparisons) | Convergent validity: r=0.61 with Symbol Search, r=0.90 with Processing Speed Index. Further, Coding displayed highest correlations with the D-KEFS Trail-Making completion time scores and reasonable convergent validity was also shown with RBANS index scores assessing Attention (2).  Divergent validity: lower correlations with working memory, perceptual reasoning, and verbal Indexes of the WAIS-IV: r=0.32, r=0.41 and 0.31 respectively (2). |
| Reference group used to develop this test:   1. If available, please define the characteristic of the norm group adopted (demographic variables used for stratification: age, education, sex, etc). 2. What kind of standardized score generated (T score, Z score, derived IQ score, percentile ranks, etc)? 3. If translated/validated in other languages, are reference norms created? If so, please specify demographic variables used for stratification (age, education, gender etc). | Group characteristics: 2,200 examinees highly matching in age/sex/education/ US regions and ethnicity.  Age in years: Age range from 16 to 90 years; Analyzed while considering 13 age-groups (16-17, 18-19, 20-24, 25-29, 30-34, 35-44, 45-54, 55-64, 65-69, 70-74, 75-79, 80-84,85-90). Approximately 200 examinees per group, except in the oldest groups where the sample was about 100 individuals.  Education in years: Five groups: ≤8, 9-11, 12, 13-15, and ≥16 years.  Sex (% women): equal numbers of men and women in each age-group, except the five oldest groups, which were represented by more women than men (consistently with the 2005 US Census proportions).  Country: USA  Type of standardized score: scaled scores, z-scores and percentile rank.  WAIS-IV has been translated in many languages. Some versions have local normative data. |

| **Responsiveness: assessed –good; not good. Not assessed (reference)** |  |
| --- | --- |
| Demonstrated to be sensitive to change (change over time or due to treatment)*  * [determined by the strength of the relationship between change in the test scores and the criterion or anchor scores] | - Coding subtest of WAIS-III (together with Symbol Search subtest) declined one year after STN DBS in PD (9). - Small gains were seen in tests measuring speed of processing with z-score changes of 0.21 in WAIS-IV Coding, following a computerized working memory updating training program (3 times/week x 5 weeks) (10). - WAIS-IV coding declined over time in nursing home residents not enrolled in a physical exercise program (11). - Predictive of cognitive decline. In PD and non-PD aging populations (12, 13). |
| Has the minimal clinically important change and minimal clinically relevant incremental difference been assessed?  (besides a statistically significant improvement) | No |
| Has this test been assessed or used in patients with other condition than that of interest? | Yes, several conditions including AD and MCI |
| Other | Increased relative theta band power has been associated with lower scores on WAIS-III Coding, indicating mild cognitive impairment. Suggesting that Coding subtest might be used as a screening method for cognitive decline in patients with PD after STN-DBS (9).  Digit Symbol (WAIS-R) associated with progression to PDD; better performance associated with maintaining normal cognition over time (14, 15).  Reduced performance on Digit Symbol (WAIS-R) seen in non-cognitively impaired participants with PD compared to healthy controls (16). |
| **Overall impression** |  |
| Strengths | This subtest from the WAIS-IV has strong normative data, based on a large sample, and strong clinimetric validation. Further, instructions and example items are quick and easy to administer. It is available in many languages. It is sensitive to change and predictive of cognitive decline in PD and non-PD. |
| Weaknesses | Severe motor deficits (such as tremor, bradykinesia or dyskinesia) can hamper its administration and interpretation. |

| **Level of Recommendation** |  |
| --- | --- |
| “Recommended” – The test was applied to the target population, AND was studied clinimetrically and found to be valid, reliable and sensitive to change, AND was used in studies by researchers, others than the developers  “Recommended with caveats” – Test’s properties were generally found to be adequate, but some of the measurement properties were not evaluated or not evaluated specifically in a PD cohort."  ‘Suggested’ – The test was applied to the target population, but only one of the other criteria applies  ‘Listed” – The test was applied to the target population, but none of the other criteria applies | Please choose one of these options:  “Recommended” |
| Which type of study is this test suitable for and which type of study is it not suitable for (screening, prevalence, etiological (e.g. case-control or genetic), treatment trial, correlation with biological markers or other scales, e.g. of parkinsonism, clinical practice for diagnosis/ screening). List all. | Suitable for:  Case-control study  Treatment trial  Screening  Prevalence  Correlation with biological markers. Etc.  Clinical practice |

**REFERENCES**

1. Wechsler D**.** Wechsler Adult Intelligence Scale-Fourth Edition (WAIS-IV). Bloomington, MN USA: NCS Pearson; 2008.

2. Wechsler D**.** Wechsler Adult Intelligence Scale - Fourth Edition: Technical and Interpretive Manual. Bloomington, MN: NCS Pearson; 2008.

3. Williamson M, Maruff P, Schembri A, Cummins H, Bird L, Rosenich E, et al. Validation of a digit symbol substitution test for use in supervised and unsupervised assessment in mild Alzheimer's disease. J Clin Exp Neuropsychol. 2022;44(10):768-79.

4. Burdick DJ, Cholerton B, Watson GS, Siderowf A, Trojanowski JQ, Weintraub D, et al. People with Parkinson's disease and normal MMSE score have a broad range of cognitive performance. Mov Disord. 2014;29(10):1258-64.

5. Liozidou A, Potagas C, Papageorgiou SG, Zalonis I**.** The role of working memory and information processing speed on wisconsin card sorting test performance in Parkinson disease without dementia. J Geriatr Psychiatry Neurol. 2012;25(4):215-21.

6. Oda H, Yamamoto Y, Maeda K**.** Neuropsychological profile of dementia with Lewy bodies. Psychogeriatrics. 2009;9(2):85-90.

7. Orsini A, Pezzuti L**.** WAIS-4: Contributo alla taratura italiana (16-69 anni): Giunti OS; 2013.

8. Streiner DL**.** Starting at the beginning: an introduction to coefficient alpha and internal consistency. J Pers Assess. 2003;80(1):99-103.

9. Yakufujiang M, Higuchi Y, Aoyagi K, Yamamoto T, Sakurai T, Abe M, et al. Predicting Neurocognitive Change after Bilateral Deep Brain Stimulation of Subthalamic Nucleus for Parkinson's Disease. World Neurosurg. 2021;147:e428-e36.

10. Walton L, Domellof ME, Boraxbekk CJ, Domellof E, Ronnqvist L, Backstrom D, et al. The Effects of Working Memory Updating Training in Parkinson's Disease: A Feasibility and Single-Subject Study on Cognition, Movement and Functional Brain Response. Front Psychol. 2020;11:587925.

11. Arrieta H, Rezola-Pardo C, Kortajarena M, Hervas G, Gil J, Yanguas JJ, et al. The impact of physical exercise on cognitive and affective functions and serum levels of brain-derived neurotrophic factor in nursing home residents: A randomized controlled trial. Maturitas. 2020;131:72-7.

12. Farina M, Breno Costa D, Webber de Oliveira JA, Polidoro Lima M, Machado WL, Moret-Tatay C, et al. Cognitive function of Brazilian elderly persons: longitudinal study with non-clinical community sample. Aging Ment Health. 2020;24(11):1807-14.

13. Young CB, Cholerton B, Smith AM, Shahid-Besanti M, Abdelnour C, Mormino EC, et al. The Parkinson's Disease Composite of Executive Functioning: A Measure for Detecting Cognitive Decline in Clinical Trials. Neurology. 2024;103(2):e209609.

14. Cholerton B, Johnson CO, Fish B, Quinn JF, Chung KA, Peterson-Hiller AL, et al. Sex differences in progression to mild cognitive impairment and dementia in Parkinson's disease. Parkinsonism Relat Disord. 2018;50:29-36.

15. Phongpreecha T, Cholerton B, Mata IF, Zabetian CP, Poston KL, Aghaeepour N, et al. Multivariate prediction of dementia in Parkinson's disease. NPJ Parkinsons Dis. 2020;6:20.

16. Cholerton BA, Poston KL, Yang L, Rosenthal LS, Dawson TM, Pantelyat A, et al. Semantic fluency and processing speed are reduced in non-cognitively impaired participants with Parkinson's disease. J Clin Exp Neuropsychol. 2021;43(5):469-80.

**Rating test evaluation**

| **Test name: Digit Span subtest from WAIS-IV** | Responses |
| --- | --- |
| **Version**  Are there several versions (alternate or parallel) of the test? If so, which one has been assessed? | Digit Span is a subtest of the Wechsler Adult Intelligence Scale, 4^th^ edition (WAIS-IV) (1, 2), with previous versions included in the WAIS-III and WAIS-R (these prior versions are still frequently used in research). The WAIS-IV adds Digit Span Sequencing (DSS) in addition to Digit Span Forward (DSF) and Digit Span Backward (DSB), which to date is less commonly used in research. Alternate versions of Digit Span, e.g., Number Span (3) or other number sequences created by generating random number spans are also used for research. |

| **Description of test**   - What population the test was originally developed for? - Which domains/components of domains do the test assess? - Are there multiple domains or a predominant domain assessed? - Does it score impaired/unimpaired? - Does it score severity   (subtle/mild/moderate/severe)? | - Subtest from the WAIS-IV, which was developed to evaluate intelligence and cognition in adolescents and adults (16 to 90 years) in both cognitively normal and clinical populations. - Assesses auditory processing, attention, encoding (DSF) and auditory working memory and mental manipulation (DSB & DSS). DSS was added to increase the working memory load of the total Digit Span test score. - If examined separately, DSF and DSB/DSS measure multiple components under the “fronto-striatal” umbrella: (1) auditory attention and (2) auditory working memory. - Does not explicitly score impaired/ unimpaired. - Score is based on severity since higher scores reflect better cognitive performance. The manual provides scaled scores, which can be converted into *z* scores and percentile ranks, allowing to compare the performance with the normative population. Regarding the ‘severity qualitative descriptors’, there are some controversies based on the applied validation/ standardization.[[4]](#footnote-4) |
| --- | --- |
| **Scaling Metric**: Type of response format? (e.g., Likert, Binary, multiple-choice, continuum, reaction time, etc.) | Each item requires a binary response (0-1 points) based on the correctness of the response. The raw score (= sum of correct responses) can be converted to an age-corrected scaled score (*M* = 10; *SD* = 3).  Total score across all subtests or total score for each subtest individually (scaled scores available) or maximum span achieved scores from each subtest (raw scores only) can be used. Span scores, while useful for clinical evaluation of patients, have less desirable test performance metrics (described below) |
| **Respondent: Patient:** any specific requirements for the target study population (e.g. non-demented, motor, auditory, visual abilities, etc)? | This test is an auditory measure and thus requires hearing sufficient to understand the examiner, and language skills sufficient to comprehend the test instructions and to repeat number strings. From the WAIS-IV Technical and Interpretive Manual (1): “To reduce auditory confounds, the use of phonetically similar digits was minimized” in the current (WAIS-IV) version. The test can also be influenced by anxiety, ADHD, and learning disabilities. |
| **Access:** How can the test be obtained (mail address or website)? | Pearson serves most of the international service orders and products ([pearsonassessments.com](file:///Users/bchol/Library/Containers/com.apple.mail/Data/Library/Mail%20Downloads/953B9DD7-D021-4E91-BF8B-1EB94F0A9B38/pearsonassessments.com)). However, some countries have to refer to different publishers (e.g., Italy: [giuntipsy.it](file:///Users/bchol/Library/Containers/com.apple.mail/Data/Library/Mail%20Downloads/953B9DD7-D021-4E91-BF8B-1EB94F0A9B38/giuntipsy.it)) |
| Copyrighted or in public domain?  Conditions for use? Fee? | Copyrighted; the full WAIS-IV must be purchased. The fee is quite expensive ($ 1,500/ € 2,000). Alternate versions are used in the public domain by generating random number spans (3). Previous versions (WMS-III/WAIS-III and earlier) are frequently used in research but may no longer be available for purchase online. |
| **Translations:** Has the test been translated and validated in other languages besides English? | Yes. The WAIS-IV has been translated in several languages[[5]](#footnote-5): English (US), English (Australia and New Zealand), English (Canada), English (UK), Chinese, Danish, Dutch, Finnish, French (Canada), French (France), German, Greek, Hebrew, Hungarian, Icelandic, Italian, Japanese, Korean, Lithuanian, Mandarin (Taiwan), Norwegian, Polish, Portuguese (Brazil), Spanish (Argentina), Spanish (Chile), Spanish (Mexico), Spanish (Spain), Swedish as well as Indonesian.  Some versions are translated, language adapted, and/or have local normative data. |

| **Test properties**  (Please provide references for all statements or note if this is your judgment) | |
| --- | --- |
| Which cognitive domain (or component/specific aspect of a cognitive domain) does this test *primarily* measure? | Attention and working memory domains. In the WAIS-IV, this subtest is included as a core test of the Working Memory Index. |
| Which aspects of the domain you are assessing are not covered? | Alternating attention, divided attention, visual attention, visual working memory, response inhibition, abstract reasoning and planning |

| **Use** |  |
| --- | --- |
| Was it *designed* to measure severity, screen or diagnosis of the domain? | It was designed to measure severity of auditory attention/working memory deficits. If the raw score is converted into the scaled score, this allows comparison of the individual performance to the normative population (in terms of *z-*scores/percentile ranks).  This test has subsequently been validated as a screening tool for MCI in PD (4, 5). |
| Is there a cut-off score? Are there measures of screening/diagnostic performance of the test? (Receiver characteristic curve analyses; sensitivity/specificity; Positive Predictive Value/Negative Predictive Value), True Positive Rate, False Positive Rate etc.) | The test manual does not provide cut-off scores; rather, age-corrected scaled scores can be converted to *z-*scores/percentiles.  However, cut-off scores for DSB in relation to the diagnostic unit (MCI/dementia) have been established in some populations:   - For use in discriminating cognitive impairment in PD: optimal cutoff <3, sens .85, spec .44, AUC .71 (4). - For discriminating cognitive impairment in geriatric inpatients: optimal cutoff <3 , sens .77, spec .78 for dementia (6). |
| Is this appropriate for the PD population? If not, why (low sensitivity and/or specificity values)? | Yes, particularly DSB (5, 7, 8). |
| **Clinimetric/psychometric properties in the target population** | |
| **Feasibility /Acceptability** |  |
| Length | Number of items: Digit Span is subdivided in 3 parts, which are all necessary to derive the Digit Span subtest scaled score:  1) DSF  2) DSB  3) DSS  Each part has a maximum of 16 points (2 levels for 8 items). Total maximum points: 48.  Average range: 5-10 minutes, including  the instructions and the example and practice items. The test will take less time if the discontinuation criterion is met (2 failures within the same item) |
| Ambiguities in instructions to patient | No |
| Ambiguities in rating anchors | No |
| Appropriateness of questions for population | Yes |
| Applicability across disease cognitive stages | Normal cognition, MCI, and early to moderate dementia |
| Applicability across disease motor severity? | H&Y: 1-4  Appropriate for all stages of motor disease as it does not require intact motor function for completion and is not a timed task. May be impacted if dysarthria is severe or if the participant has an excessively soft voice. |
| Clear instructions to raters | Yes, standard instructions for administration and scoring are included in the test manual. |
| Has the test been used by researcher other than developers?  By other groups  Not applicable? | Yes, widely used in both PD and non-PD research protocols (5, 7-14). |
| Are there instructions for dealing with missing data?  Not applicable? | Discontinue rule states that if participant fails two items in the same trial, test should be discontinued as it is unlikely the participant would be able to attain the next level. Thus, the following scores are treated as scores of 0 rather than missing data. |
| Floor and ceiling effects, skewed score distributions? | There may be ceiling effects particularly for span scores. Especially for DSF, maximum span is 8, which means the subject does not reach their capacity span (e.g., 7) until late in the test).  A subject who misses one trial at each length will have the same total correct score as another who is accurate at the initial lengths but fails twice earlier on. Because subjects receive different numbers of trials the variance of the total correct score is high relative to the mean, and is highly skewed. Such high variance may reduce sensitivity to clinical abnormalities (15). |

| **Have the component of dimensionality, variability and dependency structures been analyzed?**  (Exploratory and confirmatory factor analysis (EFA and CFA)? | Yes.[[6]](#footnote-6) CFA confirms loading on Working Memory Index, with Digit Span loading .81 on this index (1, 16)  Despite differences in the underlying traits measured by DSFW and DSBW, factor analyses generally place them in the same factor (17-19) |
| --- | --- |
|  |  |
| **Reliability: assessed – good; not good. Not assessed**  Are alternative versions developed?  If so, were they validated with reliability measures? | Assessed – good, reported in the WAIS-IV Technical and Interpretive Manual (1).  An alternate version (Number Span) was developed for research (3). Crosswalk data indicate Number Span and Digit Span were closely aligned (20). |
| Internal consistency (21)  0.9 ≤ Cronbach’s alpha (α) Excellent  0.8 ≤ α < 0.9 Good  0.7 ≤ α < 0.8 Acceptable  0.6 ≤ α < 0.7 Questionable  0.5 ≤ α < 0.6 Poor  α < 0.5 Unacceptable | Digit Span overall: .93  DSF = .84; DSB = .78; DSS = .89  >.9 in the older age groups and special populations, including MCI and dementia (1).  There is some suggestion that internal consistency is less for the total subscale score (.74), and the authors caution that Digit Span should be interpreted using DSF, DSB, and DSS test scores instead, which retain the higher internal consistency reported in the WAIS-IV standardization sample (22). |
| “Corrected item-to-total correlation” | No |
| Test/retest | Yes, short-term test-retest stability was investigated for 298 individuals from four age groups with retest intervals ranging from 8–82 days (mean retest interval of 22 days) (1).  Average across age-ranges:  DSF, DSB and DSS were .71 to .77  Overall test: .82 across age groups (0.83 after correcting for variability of the normative sample); .90 for ages 55-69; .85 for ages 70-90 (corrected) (1).  Also has good to excellent test-retest reliability when used in dual-task studies in PD (23). |
| Inter-rater | Excellent due to low ambiguity in scoring.  Interscorer agreement was examined by comparing two independent scorers of all WAIS-IV standardization record forms.  All WAIS-IV subtests are .98 to .99 (1). |
| Intra-rater | No |
| Other (e.g. Standard Error of Measurement) | Yes, average across age-ranges: .81;  .73 - .85 in older age groups (1). |
| **Validity: assessed –good; not good. Not assessed (reference)** | |
| Face and Content validity (does test appears to be measuring what it is intended to measure and do test’s items comprehensively represent the domain of interest?) | Good: WAIS-IV Technical and Interpretive Manual provides a discussion of content validity as determined by comprehensive literature and expert reviews (1). Several studies have shown that Digit Span measures auditory processing, attention, and working memory (best if DSF and DSB are assessed separately) (24-28). |
| Criterion validity (compared to gold-standard) or other criteria for  diagnosis) (e.g. other comparable tests or lesion or MRI research) | Compared to previous Wechsler Adult Intelligence Scales and Intelligence Scale for Children.   - DSB successfully identifies MCI/ dementia in PD both on and off meds (4, 5, 7). - Digit Span performance correlated with white matter hyperintensities in patients with PD (29). - Reduced frontal tau associated with impaired DSF in DLB on tau PET imaging (30). |
| Construct validity (correlations with other convergent tests and divergent tests; known-groups comparisons) | Convergent validity: r=.66 with Letter Number Sequencing, r=.56 with Arithmetic and overall r=.88 with the Working Memory Index. Reasonable convergent validity was also shown with RBANS index scores assessing Attention) (1).  Divergent validity: lower correlations with processing speed, verbal comprehension and perceptual reasoning Indexes of the WAIS-IV: r=0.29, r=0.45 and 0.45 respectively (1, 16) |
| Reference group used to develop this test:   1. If available, please define the characteristic of the norm group adopted (demographic variables used for stratification: age, education, sex, etc.). 2. What kind of standardized score generated (T score, Z score, derived IQ score, percentile ranks, etc.)? 3. If translated/validated in other languages, are reference norms created? If so, please specify demographic variables used for stratification (age, education, gender etc.) | Group characteristics: 2,200 examinees highly matching in age/sex/education/ US regions and ethnicity.  Age in years: Age range from 16 to 90 years; Analyzed while considering 13 age-groups (16-17, 18-19, 20-24, 25-29, 30-34, 35-44, 45-54, 55-64, 65-69, 70-74, 75-79, 80-84,85-90). Approximately 200 examinees per group, except in the oldest groups (ages 70-90) where the sample was about 100 examinees.  Education in years: Five groups: ≤8, 9-11, 12, 13-15, and ≥16 years.  Sex (% women): equal numbers of men and women in each age-group, except the five oldest groups, which were represented by more women than men (consistently with the 2005 US Census proportions).  Country: USA  Type of standardized score: scaled scores, z-scores and percentile rank.  WAIS-IV has been translated in many languages as previously reported. Some versions have local normative data. |

| **Responsiveness: assessed –good; not good. Not assessed (reference)** | |
| --- | --- |
| Demonstrated to be sensitive to change (change over time or due to treatment)*  * [determined by the strength of the relationship between change in the test scores and the criterion or anchor scores] | - Sensitive to change associated with aging (31). - PD-MCI has less change over time than AD-MCI (32). - Dopamine impairs performance on DSB, but not DSF (5). - Vitamin D improves performance in MCI (33). - Singing and painting interventions in AD improved performance in AD patients (34), and exercise interventions improved performance in MCI (35). |
| Has the minimal clinically important change and minimal clinically relevant incremental difference been assessed?  (besides a statistically significant improvement) | Some have examined in non-PD but a clear and meaningful minimal clinically important change has not been established. |
| Has this test been assessed or used in patients with other condition than that of interest? | Yes – The test is commonly used in many areas of research, including MCI and dementia due to AD, Huntington’s disease, bipolar disorder, schizophrenia, traumatic brain injury, etc. (5, 7-14). |
| Other | WAIS-III Digit Span had most robust correlation with cortical AChE activity in PD (36).  Early-PD patients vs healthy control participants were more impaired across all three WAIS-IV Digit Span components (DSF, DSB, DSS), but only showed reduced percentage accuracy on the components requiring manipulation (DSB, DSS). Dopaminergic medication did not affect performance in PD patients (7). |
| **Overall impression** | |
| Strengths | This subtest from the WAIS-IV has strong normative data, based on a large sample, and strong clinimetric validation. Available in many languages. Further, its instructions and example items are quick and easy to administer. There is no motor component and current version reduces floor effects so it can be administered into the advanced stages of disease. It is frequently used in PD research, with PD-specific validity/reliability studies. |
| Weaknesses | Most research incorporates previous versions (e.g., WAIS-R, WAIS/WMS-III). DSF may be less informative. Span is already at the average “ceiling” for healthy adults, and test characteristics may limit amenability to statistical analyses. DSB and DSS are generally considered more useful for PD research. Low cutoff scores raise questions as to the usefulness in screening. |

| **Level of Recommendation** | |
| --- | --- |
| “Recommended” – The test was applied to the target population, AND was studied clinimetrically and found to be valid, reliable and sensitive to change, AND was used in studies by researchers, others than the developers  “Recommended with caveats” – Test’s properties were generally found to be adequate, but some of the measurement properties were not evaluated or not evaluated specifically in a PD cohort."  ‘Suggested’ – The test was applied to the target population, but only one of the other criteria applies  ‘Listed” – The test was applied to the target population, but none of the other criteria applies | Recommended (based on WAIS-IV data and on published studies including WAIS-IV and older versions of Digit Span) |
| Which type of study is this test suitable for and which type of study is it not suitable for (screening, prevalence, etiological (e.g. case-control or genetic), treatment trial, correlation with biological markers or other scales, e.g. of parkinsonism, clinical practice for diagnosis/ screening). List all. | Suitable for:  Case-control study (in particular the DSB/DSS)  Treatment trial (in particular the DSB/DSS)  Screening (in particular the DSB/DSS)  Prevalence  Correlation with biological markers. Etc.  Clinical practice |

**REFERENCES**

1. Wechsler D**.** Wechsler Adult Intelligence Scale - Fourth Edition: Technical and Interpretive Manual. Bloomington, MN: NCS Pearson; 2008.

2. Wechsler D**.** Wechsler Adult Intelligence Scale-Fourth Edition (WAIS-IV). Bloomington, MN USA: NCS Pearson; 2008.

3. Weintraub S, Besser L, Dodge HH, Teylan M, Ferris S, Goldstein FC, et al. Version 3 of the Alzheimer Disease Centers' Neuropsychological Test Battery in the Uniform Data Set (UDS). Alzheimer Dis Assoc Disord. 2018;32(1):10-7.

4. Biundo R, Weis L, Pilleri M, Facchini S, Formento-Dojot P, Vallelunga A, et al. Diagnostic and screening power of neuropsychological testing in detecting mild cognitive impairment in Parkinson's disease. J Neural Transm (Vienna). 2013;120(4):627-33.

5. Warden C, Hwang J, Marshall A, Fenesy M, Poston KL**.** The effects of dopamine on digit span in Parkinson's disease. J Clin Mov Disord. 2016;3:5.

6. Leung JL, Lee GT, Lam YH, Chan RC, Wu JY**.** The use of the Digit Span Test in screening for cognitive impairment in acute medical inpatients. Int Psychogeriatr. 2011;23(10):1569-74.

7. Grogan JP, Knight LE, Smith L, Irigoras Izagirre N, Howat A, Knight BE, et al. Effects of Parkinson's disease and dopamine on digit span measures of working memory. Psychopharmacology (Berl). 2018;235(12):3443-50.

8. Ma J, Ma S, Zou H, Zhang Y, Chan P, Ye Z**.** Impaired serial ordering in nondemented patients with mild Parkinson's disease. PLoS One. 2018;13(5):e0197489.

9. Beales A, Whitworth A, Cartwright J, Panegyres PK, Kane RT**.** Profiling sentence repetition deficits in primary progressive aphasia and Alzheimer's disease: Error patterns and association with digit span. Brain Lang. 2019;194:1-11.

10. Cotrena C, Damiani Branco L, Ponsoni A, Samame C, Milman Shansis F, Paz Fonseca R**.** Executive functions and memory in bipolar disorders I and II: new insights from meta-analytic results. Acta Psychiatr Scand. 2020;141(2):110-30.

11. Jia J, Hu J, Huo X, Miao R, Zhang Y, Ma F**.** Effects of vitamin D supplementation on cognitive function and blood Abeta-related biomarkers in older adults with Alzheimer's disease: a randomised, double-blind, placebo-controlled trial. J Neurol Neurosurg Psychiatry. 2019;90(12):1347-52.

12. Lemiere J, Decruyenaere M, Evers-Kiebooms G, Vandenbussche E, Dom R**.** Cognitive changes in patients with Huntington's disease (HD) and asymptomatic carriers of the HD mutation--a longitudinal follow-up study. J Neurol. 2004;251(8):935-42.

13. Memic A, Streit F, Hasandedic L, Witt SH, Strohmaier J, Rietschel M, et al. Neurocognitive Endophenotypes of Schizophrenia and Bipolar Disorder and Possible Associations with FKBP Variant rs3800373. Med Arch. 2018;72(5):352-6.

14. Rossetti MA, Anderson KM, Hay KR, Del Bene VA, Celka AS, Piccolino A, et al. An Exploratory Pilot Study of Neuropsychological Performance in Two Huntington Disease Centers of Excellence Clinics. Arch Clin Neuropsychol. 2024;39(1):24-34.

15. Wilde NJ, Strauss E, Tulsky DS**.** Memory span on the Wechsler Scales. J Clin Exp Neuropsychol. 2004;26(4):539-49.

16. Orsini A, Pezzuti L**.** WAIS-4: Contributo alla taratura italiana (16-69 anni): Giunti OS; 2013.

17. Bowden SC, Petrauskas VM, Bardenhagen FJ, Meade CE, Simpson LC**.** Exploring the dimensionality of digit span. Assessment. 2013;20(2):188-98.

18. Trevino M, Zhu X, Lu YY, Scheuer LS, Passell E, Huang GC, et al. How do we measure attention? Using factor analysis to establish construct validity of neuropsychological tests. Cogn Res Princ Implic. 2021;6(1):51.

19. Specketer K, Zabetian CP, Edwards KL, Tian L, Quinn JF, Peterson-Hiller AL, et al. Visuospatial functioning is associated with sleep disturbance and hallucinations in nondemented patients with Parkinson's disease. J Clin Exp Neuropsychol. 2019;41(8):803-13.

20. Monsell SE, Dodge HH, Zhou XH, Bu Y, Besser LM, Mock C, et al. Results From the NACC Uniform Data Set Neuropsychological Battery Crosswalk Study. Alzheimer Dis Assoc Disord. 2016;30(2):134-9.

21. Streiner DL**.** Starting at the beginning: an introduction to coefficient alpha and internal consistency. J Pers Assess. 2003;80(1):99-103.

22. Gignac GE, Reynolds MR, Kovacs K**.** Digit Span Subscale Scores May Be Insufficiently Reliable for Clinical Interpretation: Distinguishing Between Stratified Coefficient Alpha and Omega Hierarchical. Assessment. 2019;26(8):1554-63.

23. Strouwen C, Molenaar EA, Keus SH, Munks L, Bloem BR, Nieuwboer A**.** Test-Retest Reliability of Dual-Task Outcome Measures in People With Parkinson Disease. Phys Ther. 2016;96(8):1276-86.

24. Banken JA**.** Clinical utility of considering digits forward and backward as separate components of the Wechsler Adult Intelligence Scale-Revised. J Clin Psychol. 1985;41(5):686-91.

25. Groth-Marnat**.** Handbook of Psychological Assessment (4th ed.). New York: WIley; 2003.

26. MacDonald MC, Almor A, Henderson VW, Kempler D, Andersen ES**.** Assessing working memory and language comprehension in Alzheimer's disease. Brain Lang. 2001;78(1):17-42.

27. Reynolds CR**.** Forward and backward memory span should not be combined for clinical analysis. Arch Clin Neuropsychol. 1997;12(1):29-40.

28. Werheid K, Hoppe C, Thone A, Muller U, Mungersdorf M, von Cramon DY**.** The Adaptive Digit Ordering Test: clinical application, reliability, and validity of a verbal working memory test. Arch Clin Neuropsychol. 2002;17(6):547-65.

29. Linortner P, McDaniel C, Shahid M, Levine TF, Tian L, Cholerton B, et al. White Matter Hyperintensities Related to Parkinson's Disease Executive Function. Mov Disord Clin Pract. 2020;7(6):629-38.

30. Wolters EE, van de Beek M, Ossenkoppele R, Golla SSV, Verfaillie SCJ, Coomans EM, et al. Tau PET and relative cerebral blood flow in dementia with Lewy bodies: A PET study. Neuroimage Clin. 2020;28:102504.

31. Bopp KL, Verhaeghen P**.** Aging and verbal memory span: a meta-analysis. J Gerontol B Psychol Sci Soc Sci. 2005;60(5):P223-33.

32. Besser LM, Litvan I, Monsell SE, Mock C, Weintraub S, Zhou XH, et al. Mild cognitive impairment in Parkinson's disease versus Alzheimer's disease. Parkinsonism Relat Disord. 2016;27:54-60.

33. Yang T, Wang H, Xiong Y, Chen C, Duan K, Jia J, et al. Vitamin D Supplementation Improves Cognitive Function Through Reducing Oxidative Stress Regulated by Telomere Length in Older Adults with Mild Cognitive Impairment: A 12-Month Randomized Controlled Trial. J Alzheimers Dis. 2020;78(4):1509-18.

34. Pongan E, Tillmann B, Leveque Y, Trombert B, Getenet JC, Auguste N, et al. Can Musical or Painting Interventions Improve Chronic Pain, Mood, Quality of Life, and Cognition in Patients with Mild Alzheimer's Disease? Evidence from a Randomized Controlled Trial. J Alzheimers Dis. 2017;60(2):663-77.

35. Zhou XL, Wang LN, Wang J, Zhou L, Shen XH**.** Effects of exercise interventions for specific cognitive domains in old adults with mild cognitive impairment: A meta-analysis and subgroup analysis of randomized controlled trials. Medicine (Baltimore). 2020;99(31):e20105.

36. Bohnen NI, Kaufer DI, Hendrickson R, Ivanco LS, Lopresti BJ, Constantine GM, et al. Cognitive correlates of cortical cholinergic denervation in Parkinson's disease and parkinsonian dementia. J Neurol. 2006;253(2):242-7.

**Rating test evaluation**

| **Test name: Symbol Search from WAIS-IV** | Responses (possible) |
| --- | --- |
| **Version**  Are there several versions (alternate or parallel) of the test? If so, which one has been assessed? | If yes, please add complete titles.  No |
| **Description of test**   - What population the test was originally developed for? - Which domains/components of domains do the test assess? - Are there multiple domains or a predominant domain assessed? - Does it score impaired/unimpaired? - Does it score severity   (subtle/mild/moderate/severe)? | - Subtest from Wechsler Adult Intelligence Scale, 4^th^ edition - Developed to evaluate intelligence and cognition in normal and clinical populations - This subtest (Symbol Search) evaluates primarily visual information processing speed. There are also aspects of short-term visual memory, visuomotor coordination, cognitive flexibility, visual discriminatioin, attention and concentration. - Does not provide impairment - Provides scaled score |
| **Scaling Metric**: Type of response format? (e.g., Likert, Binary, multiple-choice, continuum, reaction time, etc.) | Specify the scale of measurement:   - Raw score (total correct) is converted to age-corrected scaled score (mean 10, SD 3) |
| **Respondent: Patient:** any specific requirements for the target study population (e.g. non-demented, motor, auditory, visual abilities, etc)? | - Patient is respondent - Adequate auditory, visual and motor function |
| **Access:** How can the test be obtained (mail address or website)? | In USA: pearsonassessments.com  Available in multiple other countries from the local representative of Pearson |
| Copyrighted or in public domain?  Conditions for use? Fee? | Copyrighted; test must be purchased |
| **Translations:** Has the test been translated and validated in other languages besides English? | Y/N, add language mutations with at least one validity study in the target population   - Yes - WAIS-IV is available in Canada, Germany, France, Italy, Japan, Greece, Taiwan, South Korea, Sweden, Lithuania, Slovenia, Belgium, Netherlands, Austria, Switzerland, Belgium, Netherlands, Australia and New Zealand. Some versions are translated, language adapted, and/or have local normative data |

| **Test properties**  (Please provide references for all statements or note if this is your judgment) |  |
| --- | --- |
| Which cognitive domain (or component/specific aspect of a cognitive domain) does this test *primarily* measure? | Attention and processing speed  Executive function  Highest correlation of Symbol Search is with other processing speed tests (Wechsler et al., 2008) |
| Which aspects of the domain you are assessing are not covered? | Conceptualization, cognitive flexibility, planning |

| **Use** |  |
| --- | --- |
| Was it *designed* to measure severity, screen or diagnosis of the domain? | Measures processing speed; can set one’s own impairment cutoff |
| Is there a cut-off score? Are there measures of screening/diagnostic performance of the test? (Receiver characteristic curve analyses; sensitivity/specificity; Positive Predictive Value/Negative Predictive Value), True Positive Rate, False Positive Rate etc.) | No cutoff score: age-corrected scaled scores can be related to percentiles. No values given for diagnosis/screening |
| Is this appropriate for the PD population? If not, why (low sensitivity and/or specificity values)? | Yes |
| **Clinimetric/psychometric properties in the target population** |  |
| **Feasibility /Acceptability** |  |
| Length | Number of items/domains: maximum 60  Average range in minutes: 120 sec for test, about 2-4 minutes for instructions and sample completion |
| Ambiguities in instructions to patient | No |
| Ambiguities in rating anchors | No |
| Appropriateness of questions for population | Yes |
| Applicability across disease cognitive stages | Normal and MCI and early dementia |
| Applicability across disease motor severity? | H&Y 1-3 |
| Clear instructions to raters | Yes, standard manualized instructions to test administrator |
| Has the test been used by researcher other than developers?  By other groups  Not applicable? | Yes, widely used |
| Are there instructions for dealing with missing data?  Not applicable? | Y/N, please specify  No |
| Floor and ceiling effects, skewed score distributions? | Please specify  Normalized distribution |

| **Have the component of dimensionality, variability and dependency structures been analyzed?**  (Exploratory and confirmatory factor analysis (EFA and CFA)? | Y/N Please specify  Yes, confirmatory factor analysis confirms loading on processing speed index (Wechsler et al., 2008). Symbol Search loads 0.77 on the Processing Speed factor |
| --- | --- |
|  |  |
| **Reliability: assessed – good; not good. Not assessed**  Are alternative versions developed?  If so, were they validated with reliability measures? | Y/N Please specify  No alternate form |
| Internal consistency (A rule of thumb)  0.9 ≤ Cronbach’s alpha (α) Excellent  0.8 ≤ α < 0.9 Good  0.7 ≤ α < 0.8 Acceptable  0.6 ≤ α < 0.7 Questionable  0.5 ≤ α < 0.6 Poor  α < 0.5 Unacceptable  (Streiner, 2003 Journal of Personality Assessment, 80:1, 99-103, DOI: 10.1207/S15327752JPA8001_18) | 0.81 stability (split-half and Cronbach are not appropriate for speeded tests) |
| “Corrected item-to-total correlation” | Y/N, if Y, specify how large  N |
| Test/retest | Y/N, if Y, specify how large  0.80 across 22 days |
| Inter-rater | Y/N, if Y, specify how large  N but presumably perfect |
| Intra-rater | Y/N, if Y, specify how large  N |
| Other (e.g. Standard Error of Measurement) | Y/N, if Y, specify how large  1.32 scaled score units |
| **Validity: assessed –good; not good. Not assessed (reference)** |  |
| Face and Content validity (does test appears to be measuring what it is intended to measure and do test’s items comprehensively represent the domain of interest?) | Y: expert review for face and content validity; interview with examines re, for example, strategy use |
| Criterion validity (compared to gold-standard) or other criteria for  diagnosis) (e.g. other comparable tests or lesion or MRI research) | Compared to prior Wechsler Adult Intelligence Scales and Intelligence Scale for Children |
| Construct validity (correlations with other convergent tests and divergent tests; known-groups comparisons) | Convergent validity: r=0.65 with Coding, r=0.91 with Processing Speed Index; correlates with D-KEFS Trail making Letter-Number Switching (0.65) and Number and Letter Sequencing completion time (0.59).  Divergent validity: lower correlations with working memory, verbal and perceptual subtests of the WAIS-IV r=0.32 to 0.46  (Wechsler et al, 2008) and WMS-III working memory index (0.31) |
| Reference group used to develop this test:   1. If available, please define the characteristic of the norm group adopted (demographic variables used for stratification: age, education, sex, etc). 2. What kind of standardized score generated (T score, Z score, derived IQ score, percentile ranks, etc)? 3. If translated/validated in other languages, are reference norms created? If so, please specify demographic variables used for stratification (age, education, gender etc). | Group characteristics: 2,200 examinees closely matching in age, sex, education, geographic region and ethnicity distribution to US Census 2005  The average age in years (M, SD, range) Range 16-90 years; 13 age groups: 16-17, 18-19, 20-24, 25-29, 30-34, 35-44, 45-54, 55-64, 65-69, 70-74, 75-79, 80-84,85-90. N= 200 per group, except 4 oldest groups N=100  The average education in years (M, SD, range) 5 groups: ≤8, 9-11, 12, 13-15, and ≥16 years  Sex (% women): equal numbers of men and women in each age group, except 5 oldest groups that had more women than men to be consistent with US Census proportions. No sex difference on Symbol Search in PD (Chen et al 2021)  Country: USA  Translations of WAIS-IV for many countries |

| **Responsiveness: assessed –good; not good. Not assessed (reference)** |  |
| --- | --- |
| Demonstrated to be sensitive to change (change over time or due to treatment)*  * [determined by the strength of the relationship between change in the test scores and the criterion or anchor scores] | 1. Symbol Search declined 1 year after STN DBS in PD (Yakufujiang et al, 2019). Similar with SS from WAIS-III: Foley, J. A., Foltynie, T., Limousin, P., & Cipolotti, L. (2018) |
| Has the minimal clinically important change and minimal clinically relevant incremental difference been assessed?  (besides a statistically significant improvement) | Minimal clinically important change assessed Y/N?  N |
| Has this test been assessed or used in patients with other condition than that of interest? | Y/N, please specify if one or several condition  Y several |
| Other |  |
| **Overall impression** |  |
| Strengths | Large normative sample, strong clinimetric validation, quick and easy to administer |
| Weaknesses | small literature in PD; difficult if patient has marked bradykinesia or dyskinesia or tremor or other symptoms (Centi, J., Freeman, R., Gibbons, C. H., Neargarder, S., Canova, A. O., & Cronin-Golomb, A. (2017). |

| **Level of Recommendation** |  |
| --- | --- |
| “Recommended” – The test was applied to the target population, AND was studied clinimetrically and found to be valid, reliable and sensitive to change, AND was used in studies by researchers, others than the developers  “Recommended with caveats” – Test’s properties were generally found to be adequate, but some of the measurement properties were not evaluated or not evaluated specifically in a PD cohort."  ‘Suggested’ – The test was applied to the target population, but only one of the other criteria applies  ‘Listed” – The test was applied to the target population, but none of the other criteria applies | Please choose one of these options  Recommended |
| Which type of study is this test suitable for and which type of study is it not suitable for (screening, prevalence, etiological (e.g. case-control or genetic), treatment trial, correlation with biological markers or other scales, e.g. of parkinsonism, clinical practice for diagnosis/ screening). List all. | Suitable for:  Case-control study  Treatment trial  Unsuitable for (please add just categories for which the measure is critically unsuitable):  Screening  Prevalence  Differential reasoning (etiological) |

**Rating test evaluation**

| **Test name: Trail Making Test** | Responses (possible) |
| --- | --- |
| **Version**  Are there several versions (alternate or parallel) of the test? If so, which one has been assessed? | NO, D-KEFS is comparable but is not alternate nor parallel version of TMT.  Different versions of the Delis-Kaplan Executive Functioning System (DK-TMT): Comprehensive Trail Making Test (CTMT), and Connections Task (CT) |
| **Description of test**   - What population the test was originally developed for? - Which domains/components of domains do the test assess? - Are there multiple domains or a predominant domain assessed? - Does it score impaired/unimpaired? - Does it score severity   (subtle/mild/moderate/severe)? | The Trail Making Test (TMT) was developed in 1938 by Partington and Leiter as a “distributed attention” test and published as part of the Army Individual Test Battery (Partington & Leiter, 1949)^1^.  The TMT is a well-known almost standard test in (Parkinson) neuropsychological test battery.  Domains: psychomotor speed, visual search, visual attention and mental flexibility (Strauss, Sherman Spreen, 2006)^2^  Predominantly Attention and working memory domain.  Scores can be compared to norm groups for determination of severity of impairment. |
| **Scaling Metric**: Type of response format? (e.g., Likert, Binary, multiple-choice, continuum, reaction time, etc.) | Time in seconds is registered. |
| **Respondent: Patient:** any specific requirements for the target study population (e.g. non-demented, motor, auditory, visual abilities, etc)? | n.a. |
| **Access:** How can the test be obtained (mail address or website)? | Reitan Neuropsychological laboraty: [www.reitanlabs.com](http://www.reitanlabs.com). |
| Copyrighted or in public domain?  Conditions for use? Fee? | Free of charge after buying test material |
| **Translations:** Has the test been translated and validated in other languages besides English? | Y, Arabic, Chinese, Hebrew versions (letters/numerical system) are available (Strauss, Sherman Spreen, 2006)^2^ among suitability in many other languages. |

| **Test properties**  (Please provide references for all statements or note if this is your judgment) |  |
| --- | --- |
| Which cognitive domain (or component/specific aspect of a cognitive domain) does this test *primarily* measure? | Attention and working memory and mental flexibility |
| Which aspects of the domain you are assessing are not covered? | n.a. |

| **Use** |  |
| --- | --- |
| Was it *designed* to measure severity, screen or diagnosis of the domain? | Diagnosis and severity |
| Is there a cut-off score? Are there measures of screening/diagnostic performance of the test? (Receiver characteristic curve analyses; sensitivity/specificity; Positive Predictive Value/Negative Predictive Value), True Positive Rate, False Positive Rate etc.) | Cut-off score in relation to the diagnostic unit (MCI/dementia) is not present. Scores are compared healthy control group stratified for age and education (Strauss, Sherman Spreen, 2006)^2^. |
| Is this appropriate for the PD population? If not, why (low sensitivity and/or specificity values)? | Yes |
| **Clinimetric/psychometric properties in the target population** |  |
| **Feasibility /Acceptability** |  |
| Length | TMT A and TMT B contain 25 items. The administration time of the TMT is about 5-10 minutes. |
| Ambiguities in instructions to patient | N |
| Ambiguities in rating anchors | N |
| Appropriateness of questions for population | n.a. |
| Applicability across disease cognitive stages | Yes: MCI/Dementia |
| Applicability across disease motor severity? | H&Y score: 1-3 applicable. Mostly not applicable for H&Y score: ≥4 (several disabilities or confined to bed) |
| Clear instructions to raters | Y, see (Strauss, Sherman Spreen, 2006)^2^. |
| Has the test been used by researcher other than developers?  By other groups  Not applicable? | Y, TMT is used in almost every neuropsychological assessment |
| Are there instructions for dealing with missing data?  Not applicable? | Y, Most often not applicable. Some authors include a time limit of 5 minutes on part B. Participants who cannot complete part B within 5 minutes are assigned a time of 300 or 301 seconds. |
| Floor and ceiling effects, skewed score distributions? | No, normal distributed test scores |

| **Have the component of dimensionality, variability and dependency structures been analyzed?**  (Exploratory and confirmatory factor analysis (EFA and CFA)? | N.a. TMT A and B both have one score (time in seconds). |
| --- | --- |
|  |  |
| **Reliability: assessed – good; not good. Not assessed**  Are alternative versions developed?  If so, were they validated with reliability measures? | Y, good. Alternate forms reliability .80-.89 for TMT A and .78-.90 for TMT B (Strauss, Sherman Spreen, 2006)^2^. |
| Internal consistency (A rule of thumb)  0.9 ≤ Cronbach’s alpha (α) Excellent  0.8 ≤ α < 0.9 Good  0.7 ≤ α < 0.8 Acceptable  0.6 ≤ α < 0.7 Questionable  0.5 ≤ α < 0.6 Poor  α < 0.5 Unacceptable  (Streiner, 2003 Journal of Personality Assessment, 80:1, 99-103, DOI: 10.1207/S15327752JPA8001_18) | N.a. TMT A and B both have one score (time in seconds). |
| “Corrected item-to-total correlation” | N.a. TMT A and B both have one score (time in seconds). |
| Test/retest | Y adequate test-retest reliability varying with age range and population: TMT A .69-.94 and TMT B .66-.86 Part B in various neurological groups (Strauss, Sherman and Spreen, 2006)^2^. |
| Inter-rater | Y .94 for TMT A and .90 for TMT B (Strauss, Sherman Spreen, 2006)^2^. |
| Intra-rater | N |
| Other (e.g. Standard Error of Measurement) | Wide range using RCIs to determine significant change. See Strauss, Sherman and Spreen, 2006^2^ for adequate regression or Confidence Interval data. |
| **Validity: assessed –good; not good. Not assessed (reference)** |  |
| Face and Content validity (does test appears to be measuring what it is intended to measure and do test’s items comprehensively represent the domain of interest?) | Good.  TMT A and B correlate moderately: r = . .36-.61 . Measure similar but different concepts (Strauss, Sherman and Spreen, 2006) ^2^. |
| Criterion validity (compared to gold-standard) or other criteria for  diagnosis) (e.g. other comparable tests or lesion or MRI research) | Good  Correlation with several other test of attentional abilities (Strauss, Sherman and Spreen, 2006)^2^. |
| Construct validity (correlations with other convergent tests and divergent tests; known-groups comparisons) | Good Convergent validity: given consistency with lesion studies (Lezak, Howieson, Bigler and Tranel, 2012)^3^.  Many group-comparisons were made and groups were studied (Strauss, Sherman and Spreen, 2006)^2^. |
| Reference group used to develop this test:   1. If available, please define the characteristic of the norm group adopted (demographic variables used for stratification: age, education, sex, etc). 2. What kind of standardized score generated (T score, Z score, derived IQ score, percentile ranks, etc)? 3. If translated/validated in other languages, are reference norms created? If so, please specify demographic variables used for stratification (age, education, gender etc). | Many group norms were gathered in several countries/languages for several age and education groups. Varying from children to 80-84 group. See Strauss, Sherman and Spreen, 2006) ^2^ for details and best suitable comparison group with accompanying standardized scores.  Age and education are important to take into account.  Bezdicek et al 2017^4^, published norms for 60+ up to 85-96 years of age. |

| **Responsiveness: assessed –good; not good. Not assessed (reference)** |  |
| --- | --- |
| Demonstrated to be sensitive to change (change over time or due to treatment)*  * [determined by the strength of the relationship between change in the test scores and the criterion or anchor scores] | Yes, TMT has been used in many intervention studies, for instance in the majority of DBS studies. |
| Has the minimal clinically important change and minimal clinically relevant incremental difference been assessed?  (besides a statistically significant improvement) | Y Wide range using RCIs to determine significant change. See Strauss, Sherman and Spreen, 2006^2^ for adequate regression or Confidence Interval data. |
| Has this test been assessed or used in patients with other condition than that of interest? | Y In a wide range of neurological and other conditions. |
| Other | N.A. |
| **Overall impression** |  |
| Strengths | Adequate validity and reliability, used a lot in many studies including lesion and MRI studies. Many international groups use the TMT and have developed norm groups for determination of severity of impairment. |
| Weaknesses | SD is broad. Determining change in individual cases is difficult due to broad RCIs. |

| **Level of Recommendation** |  |
| --- | --- |
| “Recommended” – The test was applied to the target population, AND was studied clinimetrically and found to be valid, reliable and sensitive to change, AND was used in studies by researchers, others than the developers  “Recommended with caveats” – Test’s properties were generally found to be adequate, but some of the measurement properties were not evaluated or not evaluated specifically in a PD cohort."  ‘Suggested’ – The test was applied to the target population, but only one of the other criteria applies  ‘Listed” – The test was applied to the target population, but none of the other criteria applies | Recommended |
| Which type of study is this test suitable for and which type of study is it not suitable for (screening, prevalence, etiological (e.g. case-control or genetic), treatment trial, correlation with biological markers or other scales, e.g. of parkinsonism, clinical practice for diagnosis/ screening). List all. | Suitable for:  Screening  Prevalence  Differential reasoning (etiological)  Treatment trial  Biomarker study  Other  Unsuitable for (please add just categories for which the measure is critically unsuitable):  Case-control study  Treatment trial with small number of patients. |

**REFERENCES**

1. Partington JE, Leiter RG. Partington's pathway test. The Psychological Service Center Bulletin 1949; 1: 9–20.
2. Strauss E, Sherman EMS, Spreen O. A compendium of neuropsychological tests. Adminstration, norms, and commentary. Oxford University Press 2006.
3. Lezak MD, Howieson DB, Bigler ED, Tranel D. Neuropsychological assessment (5th ed.). Oxford University Press 2012.
4. Bezdicek O, Stepankova H, Aexlrod BN, et al. Clinimetric validity of the Trail Making Test Czech version in Parkinson's disease and normative data for older adults. Clin Neuropsychol 2017; 31(sup1):42-60.

Executive domain: Recommended Test/subtest.

**Rating test evaluation**

| **Test name: Similarities subtest from WAIS-IV** | Responses |
| --- | --- |
| **Version**  Are there several versions (alternate or parallel) of the test? If so, which one has been assessed? | Similarities is a subtest of the Wechsler Adult Intelligence Scale, 4^th^ edition (WAIS-IV) (1, 2), with previous versions included in the WAIS-III and WAIS-R. Some current research still uses older versions, and much of the published research is based on these previous versions. There are no alternate/parallel versions available for WAIS-IV. |
| **Description of test**   - What population the test was originally developed for? - Which domains/components of domains do the test assess? - Are there multiple domains or a predominant domain assessed? - Does it score impaired/unimpaired? - Does it score severity   (subtle/mild/moderate/severe)? | - Subtest from WAIS-IV, which was developed to evaluate intelligence and cognition in adolescents and adults (16 to 90 years) in both cognitively normal and clinical populations - The Similarities subtest evaluates primarily abstract verbal reasoning and conceptualization. - Does not explicitly score impaired/ unimpaired - Score is based on severity since higher scores reflect better cognitive performance. Raw scores can be converted to scaled scores, which can be converted to *z* scores and percentile ranks, allowing comparison of performance with the normative population. Regarding the ‘severity qualitative descriptors’, there are some controversies based on the applied validation/standardization[[7]](#footnote-7) |
| **Scaling Metric**: Type of response format? (e.g., Likert, Binary, multiple-choice, continuum, reaction time, etc.) | Each item is scored on an ordinal scale (0,1, or 2 points) based on the correctness of the response (higher score = better performance).  The total raw score (= sum of correct responses) can be converted to an age-corrected scaled score (*M* = 10; *SD* = 3). |
| **Respondent: Patient:** any specific requirements for the target study population (e.g. non-demented, motor, auditory, visual abilities, etc)? | Adequate hearing and language skills sufficient to comprehend the test instructions and provide verbal responses is required. |
| **Access:** How can the test be obtained (mail address or website)? | Pearson serves most of the international service orders and products ([pearsonassessments.com](file:///Users/bchol/Library/Containers/com.apple.mail/Data/Library/Mail%20Downloads/485149FF-6F54-4770-9553-B38ECAF80724/pearsonassessments.com)). However, some countries have to refer to different publishers (e.g., Italy: [giuntipsy.it](file:///Users/bchol/Library/Containers/com.apple.mail/Data/Library/Mail%20Downloads/485149FF-6F54-4770-9553-B38ECAF80724/giuntipsy.it)) |
| Copyrighted or in public domain?  Conditions for use? Fee? | Copyrighted; the full WAIS-IV must be purchased. The fee is quite expensive ($1,500/ € 2,000). |
| **Translations:** Has the test been translated and validated in other languages besides English? | Yes. The WAIS-IV has been translated in several languages[[8]](#footnote-8): English (US), English (Australia and New Zealand), English (Canada), English (UK), Chinese, Danish, Dutch, Finnish, French (Canada), French (France), German, Greek, Hebrew, Hungarian, Icelandic, Italian, Japanese, Korean, Lithuanian, Mandarin (Taiwan), Norwegian, Polish, Portuguese (Brazil), Spanish (Argentina), Spanish (Chile), Spanish (Mexico), Spanish (Spain), Swedish as well as Indonesian.  Some versions are translated, language adapted, and/or have local normative data. |

| **Test properties**  (Please provide references for all statements or note if this is your judgment) | |
| --- | --- |
| Which cognitive domain (or component/specific aspect of a cognitive domain) does this test *primarily* measure? | Executive function and language domains. In the WAIS-IV, this subtest is included as a core test of the Verbal Comprehension Index. |
| Which aspects of the domain you are assessing are not covered? | Planning, working memory, response inhibition, cognitive flexibility, visual reasoning |

| **Use** |  |
| --- | --- |
| Was it *designed* to measure severity, screen or diagnosis of the domain? | It was designed to measure severity of verbal abstract reasoning deficits. If the raw score is converted into the scaled score, this allows comparison of the individual performance to the normative population (in terms of *z-*scores/percentile ranks). |
| Is there a cut-off score? Are there measures of screening/diagnostic performance of the test? (Receiver characteristic curve analyses; sensitivity/specificity; Positive Predictive Value/Negative Predictive Value), True Positive Rate, False Positive Rate etc.) | The test manual does not provide cut-off scores; rather, age-corrected scaled scores can be converted to *z-*scores/percentiles. |
| Is this appropriate for the PD population? If not, why (low sensitivity and/or specificity values)? | Yes |
| **Clinimetric/psychometric properties in the target population** | |
| **Feasibility /Acceptability** |  |
| Length | Number of items: 18  Average range: 10-15 minutes, including  the instructions and the example and practical items. Less time is required if the discontinuation criterion is met (3 consecutive failures) |
| Ambiguities in instructions to patient | No |
| Ambiguities in rating anchors | Yes. Scoring can be ambiguous depending upon the participant’s provided responses, which can be highly variable. Some responses may fall in a borderline area, fitting neither a one nor two-point response clearly. Because it is impossible to list every possible answer the examinee may state, the answers provided in the manual are only a guide, thus requiring examiner judgment. The examiner must also use judgment in determining whether a response has been spoiled and whether a query is necessary. |
| Appropriateness of questions for population | Yes |
| Applicability across disease cognitive stages | Normal cognition, MCI and early dementia |
| Applicability across disease motor severity? | H&Y: 1-4  Appropriate for all stages of motor disease as it does not require intact motor function for completion and is not a timed task. May be impacted if dysarthria is severe or if the participant has an excessively soft voice. |
| Clear instructions to raters | Standard instructions for administration and scoring are included in the test manual. Scoring may be ambiguous (described above). |
| Has the test been used by researcher other than developers?  By other groups  Not applicable? | Yes, widely used |
| Are there instructions for dealing with missing data?  Not applicable? | Discontinue rule states that if the participant has three consecutive 0-point responses, the test should be discontinued as it is unlikely the participants would be able to attain the next level. Thus, the following scores are treated as scores of 0 rather than missing data. |
| Floor and ceiling effects, skewed score distributions? | Normalized distribution |

| **Have the component of dimensionality, variability and dependency structures been analyzed?**  (Exploratory and confirmatory factor analysis (EFA and CFA)? | Yes.[[9]](#footnote-9) CFA confirms loading on Verbal Comprehension Index, with Similarities loading .71-.72 on this index(1, 3, 4) |
| --- | --- |
|  |  |
| **Reliability: assessed – good; not good. Not assessed**  Are alternative versions developed?  If so, were they validated with reliability measures? | Assessed – good, reported in the WAIS-IV Technical and Interpretive Manual(1)  No alternate forms available |
| Internal consistency (5)  0.9 ≤ Cronbach’s alpha (α) Excellent  0.8 ≤ α < 0.9 Good  0.7 ≤ α < 0.8 Acceptable  0.6 ≤ α < 0.7 Questionable  0.5 ≤ α < 0.6 Poor  α < 0.5 Unacceptable | .87 average across age groups; .86 - .91 in the older age groups(1) |
| “Corrected item-to-total correlation” | No |
| Test/retest | Yes, short-term test-retest stability was investigated for 298 individuals from four age groups with retest intervals ranging from 8–82 days (mean retest interval of 22 days).  Average across age-ranges: .83 (.87 after correcting for the variability of the normative sample)  .87 for ages 55-69; .88 for ages 70-90 (corrected) (1). |
| Inter-rater | Excellent.  Interscorer agreement was examined by comparing two independent scorers of all WAIS-IV standardization record forms.  All WAIS-IV subtests are .98 to .99 (1). |
| Intra-rater | For Similarities, three raters (clinical psychology graduate students) independently scored the four subtests of verbal comprehension (including Similarities), and intraclass correlation coefficients were high, ranging from .91 to .97. |
| Other (e.g. Standard Error of Measurement) | Yes, average across age-ranges: 1.07 – 1.44  (1, 4); .90 - 1.12 in older age groups. Total = 1.07 (1). |
| **Validity: assessed –good; not good. Not assessed (reference)** | |
| Face and Content validity (does test appears to be measuring what it is intended to measure and do test’s items comprehensively represent the domain of interest?) | Yes, expert review for face and content validity (1). |
| Criterion validity (compared to gold-standard) or other criteria for  diagnosis) (e.g. other comparable tests or lesion or MRI research) | Compared to previous Wechsler Adult Intelligence Scales and Intelligence Scale for Children (1). |
| Construct validity (correlations with other convergent tests and divergent tests; known-groups comparisons) | Convergent validity: r=0.66 with Vocabulary, r=0.61 with Comprehension and overall r=0.67 with the Comprehension Verbal Index. Further, Similarities displayed highest correlations with the Letter Fluency and Category Fluency scores of the D-KEFS and reasonable convergent validity was also shown with RBANS index scores assessing Language (1).  Divergent validity: lower correlations with processing speed, working memory and perceptual reasoning Indexes of the WAIS-IV: r=0.27, r=0.42 and 0.44 respectively (1, 4). |
| Reference group used to develop this test:   1. If available, please define the characteristic of the norm group adopted (demographic variables used for stratification: age, education, sex, etc). 2. What kind of standardized score generated (T score, Z score, derived IQ score, percentile ranks, etc)? 3. If translated/validated in other languages, are reference norms created? If so, please specify demographic variables used for stratification (age, education, gender etc) | Group characteristics: 2,200 examinees highly matching in age/sex/education/ US regions and ethnicity.  Age in years: Age range from 16 to 90 years; Analyzed while considering 13 age-groups (16-17, 18-19, 20-24, 25-29, 30-34, 35-44, 45-54, 55-64, 65-69, 70-74, 75-79, 80-84,85-90). Approximately 200 examinees per group, except in the oldest groups (ages 70-90) where the sample was about 100 examinees.  Education in years: Five groups: ≤8, 9-11, 12, 13-15, and ≥16 years.  Sex (% women): equal numbers of men and women in each age-group, except the five oldest groups, which were represented by more women than men (consistently with the 2005 US Census proportions). No sex difference on Symbol Search in PD (Chen et al 2021)  Country: USA  Add the type of standardized score: scaled scores, z-scores and percentile rank.  WAIS-IV has been translated in many languages as previously reported. Some versions have local normative data. |

| **Responsiveness: assessed –good; not good. Not assessed (reference)** | |
| --- | --- |
| Demonstrated to be sensitive to change (change over time or due to treatment)*  * [determined by the strength of the relationship between change in the test scores and the criterion or anchor scores] | - Similarities subtest of WAIS-IV slightly declined 1-year after the STN DBS in PD: change score on Similarities (CI: –0.01 to 1.5) with PD-STN DBS showing greater negative change than PD-GPi DBS (6). - WAIS-III Similarities declined one year after the STN DBS in PD (7). - Improvements in WAIS-IV Similarities, after 3 months of combined GPi + NBM stimulation in a PD-MCI patient (*single case study*), with an improvement compared to the baseline (8). |
| Has the minimal clinically important change and minimal clinically relevant incremental difference been assessed?  (besides a statistically significant improvement) | No |
| Has this test been assessed or used in patients with other condition than that of interest? | Yes, several conditions, including Alzheimer’s disease, mild cognitive impairment, multiple sclerosis, and others (9-11). |
| Other | WAIS-IV Similarities good in discriminating PD-MCI from PD with normal cognition, but not PDD (12).  In newly diagnosed PD, about the 14% had a clinically significant performance in WAIS Similarities, below the average normative range (13).  Guzzetti and colleagues (2019)(14) investigated the association of cognitive reserve with cognition and motor function in patients with PD using the WAIS-IV Similarities subtest as one of the cognitive tests for executive functioning, and found that higher levels of cognitive reserve were associated with better performance on the Similarities subtest.  Yakufujiang and colleagues (2021) (7) demonstrated that the Similarities subtest is related to [MMSE](https://www.sciencedirect.com/topics/medicine-and-dentistry/mini-mental-state-examination) maintenance at 3 years and 5 years after STN-DBS. This suggests that a low score on the Similarities subtest may be a risk factor for developing visuospatial impairment, followed by further global cognitive decline.  Similarities subtest (cutoff point, 9; AUC, 0.71) was a predictive factor of visuospatial deficits following DBS.  Non-demented PD exerted greater cognitive workload (measure with Pupillary response reflects) to complete WAIS-IV Similarities despite similar behavioral performance compared to HC (15).  WAIS-IV Similarities shows a significant positive association with susceptibility-weighted imaging (SWI) intensity values in all the ROIs (putamen, globus pallidus, caudate nucleus, red nucleus, substantia nigra, dentate nucleus and frontal white matter) (16). |
| **Overall impression** | |
| Strengths | This subtest from the WAIS-IV has strong normative data, based on a large sample, and strong clinimetric validation. It is available in many languages. There is no motor or timed component so it can be administered into advanced stages of PD motor disease, provided speech/language is intact. |
| Weaknesses | Few studies in PD population specific to the WAIS-IV. Lack of validity studies specific to PD. Scoring can be ambiguous and thus potentially time-consuming.  The risk of possible floor effects in more advanced dementia patients should be investigated. |

| **Level of Recommendation** | |
| --- | --- |
| “Recommended” – The test was applied to the target population, AND was studied clinimetrically and found to be valid, reliable and sensitive to change, AND was used in studies by researchers, others than the developers  “Recommended with caveats” – Test’s properties were generally found to be adequate, but some of the measurement properties were not evaluated or not evaluated specifically in a PD cohort."  ‘Suggested’ – The test was applied to the target population, but only one of the other criteria applies  ‘Listed” – The test was applied to the target population, but none of the other criteria applies | Please choose one of these options:  “Recommended” |
| Which type of study is this test suitable for and which type of study is it not suitable for (screening, prevalence, etiological (e.g. case-control or genetic), treatment trial, correlation with biological markers or other scales, e.g. of parkinsonism, clinical practice for diagnosis/ screening). List all. | Suitable for:  Case-control study  Treatment trial  Biomarker studies  Prevalence  Correlation with biological markers. Etc.  Clinical practice  Unsuitable for (please add just categories for which the measure is critically unsuitable):  Screening |

**REFERENCES**

1. Wechsler D**.** Wechsler Adult Intelligence Scale - Fourth Edition: Technical and Interpretive Manual. Bloomington, MN: NCS Pearson; 2008.

2. Wechsler D**.** Wechsler Adult Intelligence Scale-Fourth Edition (WAIS-IV). Bloomington, MN USA: NCS Pearson; 2008.

3. Collinson R, Evans S, Wheeler M, Brechin D, Moffitt J, Hill G, et al. Confirmatory Factor Analysis of WAIS-IV in a Clinical Sample: Examining a Bi-Factor Model. J Intell. 2016;5(1).

4. Orsini A, Pezzuti L**.** WAIS-4: Contributo alla taratura italiana (16-69 anni): Giunti OS; 2013.

5. Streiner DL**.** Starting at the beginning: an introduction to coefficient alpha and internal consistency. J Pers Assess. 2003;80(1):99-103.

6. Odekerken VJ, Boel JA, Geurtsen GJ, Schmand BA, Dekker IP, de Haan RJ, et al. Neuropsychological outcome after deep brain stimulation for Parkinson disease. Neurology. 2015;84(13):1355-61.

7. Yakufujiang M, Higuchi Y, Aoyagi K, Yamamoto T, Sakurai T, Abe M, et al. Predicting Neurocognitive Change after Bilateral Deep Brain Stimulation of Subthalamic Nucleus for Parkinson's Disease. World Neurosurg. 2021;147:e428-e36.

8. Nombela C, Lozano A, Villanueva C, Barcia JA**.** Simultaneous Stimulation of the Globus Pallidus Interna and the Nucleus Basalis of Meynert in the Parkinson-Dementia Syndrome. Dement Geriatr Cogn Disord. 2019;47(1-2):19-28.

9. Fuentes-Dura I, Ruiz JC, Dasi C, Navarro M, Blasco P, Tomas P**.** WAIS-IV Performance in Patients With Schizophrenia. J Nerv Ment Dis. 2019;207(6):467-73.

10. Li X, Jiao J, Shimizu S, Jibiki I, Watanabe K, Kubota T**.** Correlations between atrophy of the entorhinal cortex and cognitive function in patients with Alzheimer's disease and mild cognitive impairment. Psychiatry Clin Neurosci. 2012;66(7):587-93.

11. Ouellet J, Scherzer PB, Rouleau I, Metras P, Bertrand-Gauvin C, Djerroud N, et al. Assessment of social cognition in patients with multiple sclerosis. J Int Neuropsychol Soc. 2010;16(2):287-96.

12. Biundo R, Weis L, Facchini S, Formento-Dojot P, Vallelunga A, Pilleri M, et al. Cognitive profiling of Parkinson disease patients with mild cognitive impairment and dementia. Parkinsonism Relat Disord. 2014;20(4):394-9.

13. Muslimovic D, Post B, Speelman JD, Schmand B**.** Cognitive profile of patients with newly diagnosed Parkinson disease. Neurology. 2005;65(8):1239-45.

14. Guzzetti S, Mancini F, Caporali A, Manfredi L, Daini R**.** The association of cognitive reserve with motor and cognitive functions for different stages of Parkinson's disease. Exp Gerontol. 2019;115:79-87.

15. Moon S, Kahya M, Lyons KE, Pahwa R, Akinwuntan AE, Devos H**.** Cognitive workload during verbal abstract reasoning in Parkinson's disease: a pilot study. Int J Neurosci. 2021;131(5):504-10.

16. Chiappiniello A, Tambasco N, Paoletti FP, Lisetti V, Chiarini P, Nigro P, et al. Susceptibility-weighted MRI values correlate with motor and cognitive dysfunction in Parkinson’s disease. . Physica Medica: European Journal of Medical Physics. 2018;56:207.

**Rating test evaluation**

| **Test name:** | **STROOP COLOR AND WORD TEST** |
| --- | --- |
| **Version**  Are there several versions (alternate or parallel) of the test? If so, which one has been assessed? | Despite the presence of numerous versions, a classical Stroop test consists of an interference task and control tasks. It consists of three tasks presented in a fixed order: word reading; color naming; and incongruent color-naming of color words, such as the word red printed in green ink.  As reported in Bezdicek et al. (2015),^1^ there are several versions such as: Comalli–Kaplan version,^2^ Golden version,^3–6^ and as  one of the tasks in D-KEFS (Delis-Kaplan  Executive Function System);^7^  and CAS (Cognitive Assessment  System).^8^  The versions vary in the number of  stimuli cards, of items, in the size and presentation of stimuli cards, the administration procedures, the method of scoring (see the table attached from Scarpina & Tagini, 2017)^9^, and the normative data calculation. For example, few standardized Stroop tests provide normative data for errors scores and not all versions correct for generalized slowing on the interference condition.  There are longer (up to 112 stimuli per condition) and shorter versions (i.e., 24 stimuli per condition: Victoria Stroop Test);^10^  Paper and pencil and computerized versions;  Children version.  The paper and pencil Victoria Stroop Test (VST) is the most used in PD patients and will be assessed as primary test. |
| **Description of test**   - What population the test was originally developed for? - Which domains/components of domains do the test assess? - Are there multiple domains or a predominant domain assessed? - Does it score impaired/unimpaired? - Does it score severity   (subtle/mild/moderate/severe)? | - The initial experiments were conducted on healthy volunteers;^11^ later, it was applied  to populations with brain damage or mental disorders^12,13^ - The test assesses **response inhibition** using two measures: Interference Effect and Error Score - Impairment and severity can be obtained using normative data |
| **Scaling Metric**: Type of response format? (e.g., Likert, Binary, multiple-choice, continuum, reaction time, etc.) | IE is a reaction time score.  ES is a continuous variable (number of errors).  In some versions each score is obtained by subtracting the mean score of the two “control” tasks (reading neutral words and colored dots naming) from the interference task score. In some versions the score is the number of corrected answers in a time span. (see Scarpina and Tagini, 2017 for a review)^9^  In the VST, four raw scores (D, W, and C in seconds and C error score = mistakes that are not spontaneously corrected) and one derived score (C/D time ratio, which tries to eliminate mental speed from the incongruent condition) are considered. |
| **Respondent: Patient:** any specific requirements for the target study population (e.g. non-demented, motor, auditory, visual abilities, etc)? | Good visual skills; not suitable for color blinded people or patients with dyslexia, aphasia; hemianopsia; neglect |
| **Access:** How can the test be obtained (mail address or website)? | It is possible to find stimuli on the web.  The Victoria Stroop Test is in the public domain, and users may make their own stimuli (as described in Spreen & Strauss 1998)^13^ or purchase them from the University of Victoria. In addition, adequate psychometric data, including reliability and validity, have been obtained for the VST (reviewed in Spreen & Strauss 1998). ^13^ |
| Copyrighted or in public domain?  Conditions for use? Fee? | Most are in public domains; there is copyright for some versions. |
| **Translations:** Has the test been translated and validated in other languages besides English? | Y, different versions of the stroop test are validated in different languages such as:  Italian; Spanish; Latin American Spanish speaking version; German; French; Czech; Sinhala; Korean |

| **Test properties**  (Please provide references for all statements or note if this is your judgment) |  |
| --- | --- |
| Which cognitive domain (or component/specific aspect of a cognitive domain) does this test *primarily* measure? | Executive functions, in particular the ability to inhibit an automatic response; selective attention, cognitive flexibility, working memory and speed processing are also involved.  Visuospatial and language functions are also involved. |
| Which aspects of the domain you are assessing are not covered? | Abstraction, planning, problem solving and strategy abilities are not measured directly. |

| **Use** |  |
| --- | --- |
| Was it *designed* to measure severity, screen or diagnosis of the domain? | It was designed to measure the interference effect.  It is used in population of patients therefore there are normative data which allow to measure severity of the impairment. |
| Is there a cut-off score? Are there measures of screening/diagnostic performance of the test? (Receiver characteristic curve analyses; sensitivity/specificity; Positive Predictive Value/Negative Predictive Value), True Positive Rate, False Positive Rate etc.) | Cut-off score in relation to the diagnostic unit (MCI/dementia)  I was not able to find cut off for PD-MCI for English version.  There is a study on the Czech version of the Praga Stroop test (derived from the Victoria version) which provides normative data and proves the clinical validity for the differentiation of PD-MCI and PD-MCI subtypes from healthy subjects.^1^  A study by Biundo and colleagues (2013) found the interference time score to be able to discriminate PDD from PD-CNT and PD-MCI (p<0.001).^14^  There are cut offs (normal/pathological) for general population for most versions. |
| Is this appropriate for the PD population? If not, why (low sensitivity and/or specificity values)? | It is widely used in PD and it has been proved that a poor performance is a good predictor of conversion from normal cognition to PD-MCI,^15^ and PDD.^16,17^ It is proposed as one of the tests to be used to detect PD-MCI.^18^ |
| **Clinimetric/psychometric properties in the target population** |  |
| **Feasibility /Acceptability** |  |
| Length | The VST has 24 items per task; it takes about 5 minutes to explain and perform the test (mean control task: 15 secs; mean interference task: 45 secs) |
| Ambiguities in instructions to patient | Y/N, if Y, specify N |
| Ambiguities in rating anchors | Y/N, if Y, specify N  The ambiguity is related to the number of different versions and corrections |
| Appropriateness of questions for population | Y/N, if Y, specify  N |
| Applicability across disease cognitive stages | MCI/Dementia Y  People with severe dementia may not be able to understand the instruction |
| Applicability across disease motor severity? | H&Y score: 1 (Unilateral involvement only)  H&Y score: 2 or 3(bilateral involvement with or without postural instability)  H&Y score: ≥4(several disabilities or confined to bed)  It may be difficult for patients with important dyskinesia and it should not be administered in off condition (valid rule for any test) for clinical purposes |
| Clear instructions to raters | Y/N, please specify Y |
| Has the test been used by researcher other than developers?  By other groups  Not applicable? | Y/N, please specify  Y  The test was developed in 1935 and since then many groups used it both for clinical and research purposes |
| Are there instructions for dealing with missing data?  Not applicable? | Y/N, please specify  NA |
| Floor and ceiling effects, skewed score distributions? | Please specify  VST has a skewed distribution with skewness values ranging from 1.5 for time to complete the Dot task to 4.5 for number of errors on the Neutral Word task.^10^ |

| **Have the component of dimensionality, variability and dependency structures been analyzed?**  (Exploratory and confirmatory factor analysis (EFA and CFA)? | Y/N Please specify  Y In an Iranian bilingual adolescent population.^19^ |
| --- | --- |
|  |  |
| **Reliability: assessed – good; not good. Not assessed**  Are alternative versions developed?  If so, were they validated with reliability measures? | Y/N Please specify  There are different versions but not alternative forms for the same version |
| Internal consistency (A rule of thumb)  0.9 ≤ Cronbach’s alpha (α) Excellent  0.8 ≤ α < 0.9 Good  0.7 ≤ α < 0.8 Acceptable  0.6 ≤ α < 0.7 Questionable  0.5 ≤ α < 0.6 Poor  α < 0.5 Unacceptable  (Streiner, 2003 Journal of Personality Assessment, 80:1, 99-103, DOI: 10.1207/S15327752JPA8001_18) | NA |
| “Corrected item-to-total correlation” | Y/N, if Y, specify how large  N |
| Test/retest | Y/N, if Y, specify how large  Y coefficients of .90, .83, and .91 for time to complete the Dot, Neutral Word, and Color Word tasks, respectively. Trial-retrial reliability for this test is above 0.75.^13^ |
| Inter-rater | Y/N, if Y, specify how large N |
| Intra-rater | Y/N, if Y, specify how large N |
| Other (e.g. Standard Error of Measurement) | Y/N, if Y, specify how large N |
| **Validity: assessed –good; not good. Not assessed (reference)** |  |
| Face and Content validity (does test appears to be measuring what it is intended to measure and do test’s items comprehensively represent the domain of interest?) | A study demonstrated a good content validity of the Golden’s Stroop version.^20^ |
| Criterion validity (compared to gold-standard) or other criteria for  diagnosis) (e.g. other comparable tests or lesion or MRI research) | Compared to other tests (Golden’s version): the Stroop Words Reading requires speed of visual search, Stroop Words Reading reflects working memory  and speed, whereas SCW reflects working memory, conflict monitoring, and speed. The results also suggest that the analyzed derived interference indexes (i.e., interference and ratio scores) minimize speed of visual search. In this regard, whereas Golden’s interference score reflected both working memory and conflict monitoring, the ratio score resulted to be a marker of conflict monitoring.^20^ |
| Construct validity (correlations with other convergent tests and divergent tests; known-groups comparisons) | Add data regarding:  Convergent validity  In the Golden’s Version, multiple regression analysis showed that Stroop Words Reading score was predicted by WAIS-IV Digit Symbol score (accounting for a 4.9% of the variance).  Stroop Color Naming score was predicted by WAIS-IV Digit Span score, and by WAIS-IV Digit Symbol score (accounting together for a 16.5% of the variance).  Stroop Color Word score suggested that working memory, conflict monitoring, and speed of visual search were the main contributing variables (as measured by Digit Span, Incongruent RT, and Digit Symbol, respectively), accounting for 21.9% of the variance.^20^ |
| Reference group used to develop this test:   1. If available, please define the characteristic of the norm group adopted (demographic variables used for stratification: age, education, sex, etc). 2. What kind of standardized score generated (T score, Z score, derived IQ score, percentile ranks, etc)? 3. If translated/validated in other languages, are reference norms created? If so, please specify demographic variables used for stratification (age, education, gender etc). | For the VST:  The original norms (Regard, 1981) were published with a wide age range but of limited sample size (N = 126; age range: 20–94 years).^21^ Additional normative data are published for elderly (aged 50+) individuals,^22^ and for a relatively small (N = 272) community sample of individuals aged 18–94.^10^  The largest normative English-speaking sample to date enrolled 3362 healthy subjects.^23^  Group characteristics:  The average age in years was 46.5 SD 8.7 (range: <35 years to > 55 years). Levels of education ≤high school, some college, college degree, graduate degree most of the participants were in “some college” or “college degree” level (1057 and 1222 respectively).  Gender 53% women  Country USA  Means, SD and percentiles by age and education are given for time (trials 1,2, and 3); interference scores (Trial3/trial1; Trial 3/trial2) and error score.  Add the language of translation and group characteristics:  French:  There is a study on a sample of 244 healthy community-dwelling French adults aged between 50 and 94 years old (France).^22^  Another study enrolled 646 adults and elderly people (383 women; 263 men), aged  between 47 and 87 (mean age 74.80 years; SD 7.09), with an education level varying between 3 and 23 years (mean education  level 13.33 years; SD 4.21).  Country: Quebec (Canada).^24^  Czech:  Sample of 539 older adults: age range 64-96 years; education level lower (8–11 years of formal education) or higher; 54.2% women; provides conversion from raw scores to scaled scores for the three times scores and error score; provides normative formulas for the four scores, means and cumulative percentages associated with raw error scores on color word task and on dot and neutral word time scores by age group.  Country: Czech Republic.^1^  Sinhala:  A sample of Six-hundred and six healthy, community-living, Sinhala-speaking adults (273 men) aged 19–83 years were enrolled  Country Sri-Lanka.^25^  Arabic adaptation:  359 participants aged from 50 to 85 years;^26^ low (below Baccalaureat) vs high (Baccalaureate qualification and higher) educational level. |

| **Responsiveness: assessed –good; not good. Not assessed (reference)** |  |
| --- | --- |
| Demonstrated to be sensitive to change (change over time or due to treatment)*  * [determined by the strength of the relationship between change in the test scores and the criterion or anchor scores] | If Yes, please specify  In PD, Stroop was able to capture a change in patients: pre- and post-DBS stimulation,^27^ following the DBS stimulation frequency,^28^ as well as in ON versus OFF DRT.^29^  In multiple Sclerosis it has been reported a change at 6-month follow-up.^30^ |
| Has the minimal clinically important change and minimal clinically relevant incremental difference been assessed?  (besides a statistically significant improvement) | Minimal clinically important change assessed Y/N?  N |
| Has this test been assessed or used in patients with other condition than that of interest? | Y it is a highly sensitive tool for differentiating elderly individuals with mild AD, mild cognitive impairment (MCI), and normal cognition.^31,32^  Multiple Sclerosis, Parkinson’s disease and atypical parkinsonisms, Dementia with Lewy bodies.^33–35^ |
| Other |  |
| **Overall impression** |  |
| Strengths | Good measure of executive functions; useful in PD population since a poor performance is a good predictor of conversion from normal cognition to PD-MCI and to PDD.^36^ |
| Weaknesses | Too many versions and too many scoring methods could make it difficult to choose which one to use. Gaze palsy can hamper its administration. |

| **Level of Recommendation** |  |
| --- | --- |
| “Recommended” – The test was applied to the target population, AND was studied clinimetrically and found to be valid, reliable and sensitive to change, AND was used in studies by researchers, others than the developers  “Recommended with caveats” – Test’s properties were generally found to be adequate, but some of the measurement properties were not evaluated or not evaluated specifically in a PD cohort."  ‘Suggested’ – The test was applied to the target population, but only one of the other criteria applies  ‘Listed” – The test was applied to the target population, but none of the other criteria applies | Recommended |
| Which type of study is this test suitable for and which type of study is it not suitable for (screening, prevalence, etiological (e.g. case-control or genetic), treatment trial, correlation with biological markers or other scales, e.g. of parkinsonism, clinical practice for diagnosis/ screening). List all. | Suitable for:  Screening  Prevalence  Differential reasoning (etiological)  Case-control study  Biomarker study  Other  Unsuitable for (please add just categories for which the measure is critically unsuitable):  Treatment trial |

Table from Scarpina & Tagini 2017: scoring methods can be really different also for the same version of the Stroop Test

**REFERENCES**

1. Bezdicek O, Lukavsky J, Stepankova H, et al. The Prague Stroop Test: Normative standards in older Czech adults and discriminative validity for mild cognitive impairment in Parkinson’s disease. Journal of Clinical and Experimental Neuropsychology [online serial]. Routledge; 2015;37:794–807.

2. Mitrushina MN, Boone KB, Razani LJ, et al. Handbook of Normative Data for Neuropsychological Assessment. Second Edition, Second Edition. Oxford, New York: Oxford University Press; 2005.

3. Golden CJ. Identification of brain disorders by the Stroop Color and Word Test. Journal of clinical psychology. 1976;32.

4. Golden C, Freshwater SM, Golden Z. Stroop color and word test. Epub 1978.

5. Graf P, Uttl B, Tuokko H. Color- and picture-word Stroop tests: performance changes in old age. J Clin Exp Neuropsychol. 1995;17:390–415.

6. Trenerry MR, Crosson BA, DeBoe J, Leber WR. Stroop neuropsychological screening test. Psychological Assessment Resources; 1989.

7. Delis DC, Kaplan E, Kramer JH. Delis-Kaplan executive function system. Assessment. Epub 2001.

8. Naglieri JA, Das JP. Das-Naglieri cognitive assessment system. Itasca, IL: Riverside. Epub 1997.

9. Scarpina F, Tagini S. The Stroop Color and Word Test. Front Psychol [online serial]. 2017;8. Accessed at: http://journal.frontiersin.org/article/10.3389/fpsyg.2017.00557/full.

10. Troyer AK, Leach L, Strauss E. Aging and response inhibition: Normative data for the Victoria Stroop Test. Neuropsychol Dev Cogn B Aging Neuropsychol Cogn. 2006;13:20–35.

11. Stroop JR. Studies of interference in serial verbal reactions. Journal of Experimental Psychology. US: Psychological Review Company; 1935;18:643–662.

12. Lansbergen MM, Kenemans JL, van Engeland H. Stroop interference and attention-deficit/hyperactivity disorder: a review and meta-analysis. Neuropsychology. 2007;21:251–262.

13. Spreen O, Strauss E. A compendium of neuropsychological tests: Administration, norms, and commentary, 2nd ed. New York, NY, US: Oxford University Press; 1998. p. xvi, 736.

14. Biundo R, Weis L, Pilleri M, et al. Diagnostic and screening power of neuropsychological testing in detecting mild cognitive impairment in Parkinson’s disease. J Neural Transm (Vienna). 2013;120:627–633.

15. Santangelo G, Vitale C, Picillo M, et al. Mild Cognitive Impairment in newly diagnosed Parkinson’s disease: A longitudinal prospective study. Parkinsonism Relat Disord. 2015;21:1219–1226.

16. Janvin CC, Aarsland D, Larsen JP. Cognitive predictors of dementia in Parkinson’s disease: a community-based, 4-year longitudinal study. J Geriatr Psychiatry Neurol. 2005;18:149–154.

17. Mahieux F, Fénelon G, Flahault A, Manifacier MJ, Michelet D, Boller F. Neuropsychological prediction of dementia in Parkinson’s disease. J Neurol Neurosurg Psychiatry. 1998;64:178–183.

18. Litvan I, Goldman JG, Tröster AI, et al. Diagnostic criteria for mild cognitive impairment in Parkinson’s disease: Movement Disorder Society Task Force guidelines. Movement Disorders. 2012;27:349–356.

19. Malek A, Hekmati I, Amiri S, Pirzadeh J, Gholizadeh H. The standardization of Victoria Stroop Color-Word Test among Iranian bilingual adolescents. Arch Iran Med. 2013;16:380–384.

20. Periáñez JA, Lubrini G, García-Gutiérrez A, Ríos-Lago M. Construct Validity of the Stroop Color-Word Test: Influence of Speed of Visual Search, Verbal Fluency, Working Memory, Cognitive Flexibility, and Conflict Monitoring. Arch Clin Neuropsychol. 2021;36:99–111.

21. Regard M. Stroop test–Victoria version. Victoria, BC: Neuropsychological Laboratory, University of Victoria. Epub 1981.

22. Bayard S, Erkes J, Moroni C, Collège des Psychologues Cliniciens spécialisés en Neuropsychologie du Languedoc Roussillon (CPCN Languedoc Roussillon). Victoria Stroop Test: normative data in a sample group of older people and the study of their clinical applications in the assessment of inhibition in Alzheimer’s disease. Arch Clin Neuropsychol. 2011;26:653–661.

23. Hankee LD, Preis SR, Piers RJ, et al. Population Normative Data for the CERAD Word List and Victoria Stroop Test in Younger- and Middle-Aged Adults: Cross-Sectional Analyses from the Framingham Heart Study. Exp Aging Res. 2016;42:315–328.

24. Tremblay MS, Carson V, Chaput J-P, et al. Canadian 24-Hour Movement Guidelines for Children and Youth: An Integration of Physical Activity, Sedentary Behaviour, and Sleep. Appl Physiol Nutr Metab. 2016;41:S311-327.

25. Dassanayake TL, Hewawasam C, Baminiwatta A, Ariyasinghe DI. Regression-based, demographically adjusted norms for Victoria Stroop Test, Digit Span, and Verbal Fluency for Sri Lankan adults. Clin Neuropsychol. 2021;35:S32–S49.

26. Kettani Z, Bichra MZ, Ahami AT. Test du Stroop Victoria – Adaptation arabophone. Matériel, consignes, procédure de cotation et données normatives. NPG Neurologie - Psychiatrie - Gériatrie [online serial]. 2020;20:6–12.

27. Odekerken VJJ, Boel JA, Geurtsen GJ, et al. Neuropsychological outcome after deep brain stimulation for Parkinson disease. Neurology [online serial]. Wolters Kluwer Health, Inc. on behalf of the American Academy of Neurology; 2015;84:1355–1361.

28. Qin G, Xie H, Shi L, et al. Unlocking potential: low frequency subthalamic nucleus stimulation enhances executive function in Parkinson’s disease patients with postural instability/gait disturbance. Front Neurosci [online serial]. 2023;17:1228711.

29. Djamshidian A, Cardoso F, Grosset D, Bowden-Jones H, Lees AJ. Pathological gambling in Parkinson’s disease--a review of the literature. Mov Disord. 2011;26:1976–1984.

30. Barker-Collo SL, Purdy SC. Determining the Presence of Reliable Change over Time in Multiple Sclerosis: Evidence from the PASAT, Adjusting-PSAT, and Stroop Test. Int J MS Care. 2013;15:170–178.

31. Bondi MW, Serody AB, Chan AS, et al. Cognitive and neuropathologic correlates of Stroop Color-Word Test performance in Alzheimer’s disease. Neuropsychology. 2002;16:335–343.

32. Kramer JH, Nelson A, Johnson JK, et al. Multiple Cognitive Deficits in Amnestic Mild Cognitive Impairment. Dement Geriatr Cogn Disord [online serial]. 2006;22:306–311.

33. Santangelo G, Bisecco A, Trojano L, et al. Cognitive performance in multiple sclerosis: the contribution of intellectual enrichment and brain MRI measures. J Neurol. 2018;265:1772–1779.

34. Fiorenzato E, Antonini A, Camparini V, Weis L, Semenza C, Biundo R. Characteristics and progression of cognitive deficits in progressive supranuclear palsy vs. multiple system atrophy and Parkinson’s disease. J Neural Transm (Vienna). 2019;126:1437–1445.

35. Park KW, Kim HS, Cheon S-M, Cha J-K, Kim S-H, Kim JW. Dementia with Lewy Bodies versus Alzheimer’s Disease and Parkinson’s Disease Dementia: A Comparison of Cognitive Profiles. J Clin Neurol. 2011;7:19–24.

36. Chung SJ, Lee HS, Kim H-R, et al. Factor analysis–derived cognitive profile predicting early dementia conversion in PD. Neurology [online serial]. Wolters Kluwer Health, Inc. on behalf of the American Academy of Neurology; 2020;95:e1650–e1659.

**Rating test evaluation**

| **Test name:** | Wisconsin Card Sorting test (WCST) |
| --- | --- |
| **Version**  Are there several versions (alternate or parallel) of the test? If so, which one has been assessed? | Two traditional versions of the WCST exist. These are the WCST-128 (long; 20-30 min) version and the WCST-64 (short) version (Greve, 2001; Heaton et al., 1993). Scores on the two versions are generally similar but may not be identical (Axelrod et al., 1996; Gardizi et al., 2019; Greve, 2001). A modified version (Nelson, 1976) with 48 deck cards also exists, in which the participant has to reach 6 consecutive trials (responses) correct (instead of 10), and ambiguous cards are not included, which helps to minimise client frustration; sometimes the category change is signalled (Schrelten, 2010; 10-15 min; but see Zubicaray and Ashton, 1996). There are also computer versions, intended to be similar (Fortuny and Heaton, 1996; Heaton and PAR Staff, 1999; Heaton and PAR Staff, 2000) or using different stimuli and procedures but intended to be variations on similar general principles (CANTAB). Some computerised WCST versions include a large number of switches (e.g. 41 switches), when the aim is to focus on different errors made and trial by trial performance (Steinke et al., 2020).  The traditional pen and paper versions are assessed here. The Milwaukee and Weigl tests were not considered. |
| **Description of test**   - What population the test was originally developed for? - Which domains/components of domains do the test assess? - Are there multiple domains or a predominant domain assessed? - Does it score impaired/unimpaired? - Does it score severity   (subtle/mild/moderate/severe)? | It was first developed in 1948 to assess perseveration, abstract reasoning and set-shifting in normal adult populations (Berg, 1948). It is now generally used to assess clinical populations (Heaton et al., 1993; Lezak et al., 2012).  Executive function. Since 1963, it has consistently been associated with frontal lobe functions, although non-frontal lesions and non-frontal activation are also associated with performance (Milner, 1963; Demakis, 2003; Dias et al, 1996; Friedman and Robbins, 2021; Heaton et al., 1993; Lezak et al., 2012; Myake et al, 2000; Nyhus and Barcelo, 2009).  The WCST is among the three most commonly used “executive function” tests (with Trail Making Test and Stroop; Rabin, Barr and Burton, 2005).  The predominant cognitive domain assessed is executive function. This test is widely regarded as the “gold standard” for assessing executive function, particularly in terms of cognitive flexibility and attention/task switching. It focuses on the number of perseverative errors, which are category repetitions that occur in response to negative feedback (Diamond, 2013). Additionally, this test has been used to validate other measures of cognitive flexibility and task switching.  However, Total Errors (e.g. used by Heaton et al., 2014 for validating the NIH toolbox) may represent a more general factor than measures of perseveration. That is, the two “primary” measures are not identical (and a larger effect size is found in PD for total errors / global performance than for perseverative errors (Lange et al., 2018).  Within the executive function domain, the test also measures strategic planning, working memory, response inhibition and impulsive responses (e.g. Ashendorf et al., 2008; Rabi et al., 2020).  There is evidence that the test reflects both automatic stimulus-response learning as well as higher order concept/category formation /learning (dual-level model of reinforcement learning; Steinke et al., 2020).  At least 10 measures can be generated by the standard WCST, but a measure of perseveration and total categories achieved are generally the primary measures used (Lange et al., 2018).  Impaired WCST performance, which is related to dopamine depletion and motor severity, is a well-established neuropsychological effect of Parkinson’s disease (PD; Lange et al., 2018; Marras et al., 2014). The dimensions typically most often impacted in PD are number of sorts completed, perseverative errors and to lesser extent trials to complete the first category. Loss of set errors are more often found later than early in the disease. Beneficial effects of dopamine are believed to be related to nigro-striatal systems, but are not always found, which may reflect the severity of changes in mesocortical and mesolimbic systems or more general pathophysiology (Steinke et al., 2020).  Heaton et al. (1993) describe a range of descriptor classifications for total errors and various “perseverative” measures are based on Standard Scores (SS, mean = 100, SD = 15) and T-Scores (mean = 50; SD = 10). For these:  SS, 70 – 76 (T, 30 - 34) = mildly to moderately impaired;  SS, 62 – 69 (T, 25 – 29) = moderately impaired;  SS, 55 – 61 (T, 20 - 24) = moderately to severely impaired;  SS, 54 or less (T, 19 or less) = severely impaired range.  Note, for Heaton et al. (1993), the number of categories completed, which is one of the primary measures, follows a “categorical” range of percentile conversions to account for skewed score distributions. For number of categories, there are only three classification levels by age 60+yrs (at 60y: 0 categories < 1%ile; 1-2 categories = 2-16^th^ %ile; 3-6 > 16^th^ %ile; at 65yrs and 70yrs, 2-6 categories = > 16^th^ %ile) until age 75 when only two levels are feasible (when 1-6 categories = > 16^th^ %ile); from 85yrs, the categories completed cannot be used. Note, research studies often use non-parametric analyses for the raw score to determine group differences. Other learning measures can be used that are also “categorical” in terms of %ile conversions.  Through the use of cut-offs generated relative to norms or controls (see above). |
| **Scaling Metric**: Type of response format? (e.g., Likert, Binary, multiple-choice, continuum, reaction time, etc.) | Binary – correct (1) or incorrect items (0). Continuous and derived scores are generated on that basis. |
| **Respondent: Patient:** any specific requirements for the target study population (e.g. non-demented, motor, auditory, visual abilities, etc)? | Generally feasible for most patients, including MCI patients, but those beyond mild dementia will struggle to understand the instructions. The WCST has been used in Alzheimer patients, but the findings are less consistent than is the case for PD (Guarino et al., 2019; Lange et al., 2018). |
| **Access:** How can the test be obtained (mail address or website)? | <https://www.parinc.com/>  https://www.wpspublish.com/wcst-wisconsin-card-sorting-test  A free computer-presented version: <https://www.psytoolkit.org/experiment-library/wcst.html> |
| Copyrighted or in public domain?  Conditions for use? Fee? | The primary WCST (and the standard version of the Modified WCST) is copyrighted by PAR and can be purchased online from various websites. |
| **Translations:** Has the test been translated and validated in other languages besides English? | Y/N, add language mutations with at least one validity study in the target population  The test has been translated into many languages and normative data provided.  For example, these include Argentinian, Italian, Lebanese, Spanish, Spanish (and African-) American, Portuguese, Taiwanese (Del Pino et al. 2016; Faustino et al., 2020; Laiacona et al., 2000; Miranda et al., 2020; Norman et al., 2011; Rammal et al., 2019; Shan et al., 2008).  Not yet found if these translations have been used in the PD population. |

| **Test properties**  (Please provide references for all statements or note if this is your judgment) |  |
| --- | --- |
| Which cognitive domain (or component/specific aspect of a cognitive domain) does this test *primarily* measure? | The primary cognitive domain of the WCST is executive function (see above for details and references). |
| Which aspects of the domain you are assessing are not covered? | The primary measures covered are perseverative scores (for cognitive flexibility) and total correct or similar measures (for concept formation). Less attention is given to other measures unless they help explain overall performance. |

| **Use** |  |
| --- | --- |
| Was it *designed* to measure severity, screen or diagnosis of the domain? | Initially designed for normal populations (as an adjunct for intelligence testing), the standard WCST is used as a clinical instrument for various conditions in adults. It has also been used to assess performance in school-aged children. |
| Is there a cut-off score? Are there measures of screening/diagnostic performance of the test? (Receiver characteristic curve analyses; sensitivity/specificity; Positive Predictive Value/Negative Predictive Value), True Positive Rate, False Positive Rate etc.) | Cut-off score in relation to the diagnostic unit (MCI/dementia)  Not found any. Most studies compare PD vs HC. The recent meta-analysis review of the WCST and PD, with over 150 studies, did not specify any effects related to PD-MCI (Lange et al., 2018). Otherwise, only differences between PD-N and PD-MCI are provided but these studies are relatively few (e.g., Galtier et al., 2016; Leroi et al., 2012; Petkus et al., 2019). |
| Is this appropriate for the PD population? If not, why (low sensitivity and/or specificity values)? | Yes. The WCST has been applied to the PD population in a range of clinical and research applications (Lange et al., 2018; Marras et al., 2014). |
| **Clinimetric/psychometric properties in the target population** |  |
| **Feasibility /Acceptability** |  |
| Length | Number of items/domains  Average range in minutes  20-30 minutes or 10-15 minutes, depending on which version of test is administered (long or short). The long version can be more time-consuming and sometimes a problem in terms of maintaining motivation. |
| Ambiguities in instructions to patient | Y/N, if Y, specify  N (within each version) |
| Ambiguities in rating anchors | Y/N, if Y, specify  N (within each version) |
| Appropriateness of questions for population | Y/N, if Y, specify  Y (executive function and the specifics of measures used in the WCST; Lange et al., 2018) |
| Applicability across disease cognitive stages | MCI/Dementia  It is applicable to PD-MCI. The tests can be used with mild PDD, and mild dementia with Lewy Bodies patients, based on a MMSE > 19 (Petrova et al., 2012; Petrova et al., 2015). |
| Applicability across disease motor severity? | H&Y score: 1 (Unilateral involvement only)  H&Y score: 2 or 3(bilateral involvement with or without postural instability)  H&Y score: ≥4(several disabilities or confined to bed)  Suitable across motor disease severity, including H&Y 1, 2, and 3 (Lange et al., 2018). A few studies have included H&Y stage 4 patients (Cropley et al., 2008). |
| Clear instructions to raters | Y/N, please specify  Clear instructions in the standardised versions. Scoring requires practice, but this is not an issue with computerized versions. |
| Has the test been used by researcher other than developers?  By other groups  Not applicable? | Y/N, please specify  Yes – used in a variety of neurological patient groups and in neurodevelopmental disorders. |
| Are there instructions for dealing with missing data?  Not applicable? | Y/N, please specify  Generally not an issue and incomplete testing can be accommodated by the scoring system. Shorter versions are preferred when the traditional full tests is seen as not feasible for some patients. |
| Floor and ceiling effects, skewed score distributions? | Please specify  The primary measures of errors and perseverative responses provide very good normative distributions. As stated above, categorical norms are favoured for the number of categories completed, trials to complete the first category, failure to maintain set and learning to learn. There is a problem with the categorical measures in terms of floor effects for older age groups. |

| **Have the component of dimensionality, variability and dependency structures been analyzed?**  (Exploratory and confirmatory factor analysis (EFA and CFA)? | Y/N Please specify  Y (Paolo et al., 1995; Greve et al., 1997; Greve et al., 1999; Greve et al., 2005).  For a summary of factor structure for the MCST, including the standardized short form, see Greve (2001). Generally, a 3-factor structure is reported, although error scores and perseverative responses may belong to the same factor, which may be related to general cognitive function in PD (Paolo et al., 1995). One study reported one factor when looking at an alcohol-dependent sample (Bowden et al., 1998). |
| --- | --- |
|  |  |
| **Reliability: assessed – good; not good. Not assessed**  Are alternative versions developed?  If so, were they validated with reliability measures? | Y/N Please specify  Good to excellent (0.85 – 0.90+ for most primary measures). |
| Internal consistency (A rule of thumb)  0.9 ≤ Cronbach’s alpha (α) Excellent  0.8 ≤ α < 0.9 Good  0.7 ≤ α < 0.8 Acceptable  0.6 ≤ α < 0.7 Questionable  0.5 ≤ α < 0.6 Poor  α < 0.5 Unacceptable  (Streiner, 2003 Journal of Personality Assessment, 80:1, 99-103, DOI: 10.1207/S15327752JPA8001_18) | Not found yet in PD.  Internal (within-test) reliability has seldom been reported (Kopp et al., 2019).  Recently, the split-half reliability for “neurological patients” was generally “good to excellent” for the Modified WCST (Kopp et al., 2021). |
| “Corrected item-to-total correlation” | Y/N, if Y, specify how large |
| Test/retest | Y/N, if Y, specify how large  Given the nature of the test, between-test consistency is poorer due to changes in participant strategies once they become familiar with the test (Bowden et al., 1998; Schretlen, 2010). This is poor (r =0.57) when the test-retest delay is a matter of days (Kopp et al., 2019). |
| Inter-rater | Y/N, if Y, specify how large  Y: Inter-rater reliability reported by Heaton et al. (1993) is generally good to excellent (0.89 – 1.00), with the exception of “Learning to Learn”. |
| Intra-rater | Y/N, if Y, specify how large  According to Heaton et al. (1993), intra-rater reliability is also good to excellent (0.83-1.0). |
| Other (e.g. Standard Error of Measurement) | Y/N, if Y, specify how large  Not located this in PD studies yet. The Heaton et al (1993) manual provides SEM values between 7.9 and 11.7, for scores with a mean of 100 and SD of 15. |
| **Validity: assessed –good; not good. Not assessed (reference)** | Good to excellent measure of executive function (Heaton et al., 1993; Paolo et al., 1995).  Sensitivity is more robust than specificity, which reflects the fact that executive function is affected in multiple disorders (Heaton et al., 1993) |
| Face and Content validity (does test appears to be measuring what it is intended to measure and do test’s items comprehensively represent the domain of interest?) | Yes, from the perspective of concept formation and cognitive flexibility. The traditional and shortened WCST, in which the correct category and category switch are not explicitly signalled, appear to perform this measurement more effectively. |
| Criterion validity (compared to gold-standard) or other criteria for  diagnosis) (e.g. other comparable tests or lesion or MRI research) | The WCST is generally regarded as the primary measure for cognitive flexibility and concept formation in terms of executive function (Lange et al., 2018). Although the WCST is deemed to be a sensitive measure of prefrontal cortex function and related cortico-subcortical networks, the standard version of the test lacks specificity (Demakis, 2003; Nyhus and Barcelo, 2009; Steinke et al., 2020). |
| Construct validity (correlations with other convergent tests and divergent tests; known-groups comparisons) | Add data regarding:  Convergent validity  Divergent validity  Group-comparisons  Plenty of studies have provided evidence for convergent validity.  In terms of divergent validity, Paolo et al. (1995) reported that the factor structure for the WCST did not load on to memory and attention measures in normal elderly and PD patients. This is perhaps one of the most robust studies in this regard.  As stated above: The WCST has consistently been associated with frontal lobe functions, although non-frontal lesions and non-frontal activation are also associated with performance Milner, 1963; Demakis, 2003; Dias et al, 1996; Friedman and Robbins, 2021; Heaton et al., 1993; Lezak et al., 2012; Myake et al, 2000; Nyhus and Barcelo, 2009).  General IQ (perhaps especially fluid IQ) and working memory, but not information processing speed, are related to WCST performance (Liozidou et al., 2012; Kopp et al., 2019). |
| Reference group used to develop this test:   1. If available, please define the characteristic of the norm group adopted (demographic variables used for stratification: age, education, sex, etc). 2. What kind of standardized score generated (T score, Z score, derived IQ score, percentile ranks, etc)? 3. If translated/validated in other languages, are reference norms created? If so, please specify demographic variables used for stratification (age, education, gender etc). | The information here is based on Heaton et al. (1993)  Group characteristics:  The average age in years (M, SD, range)  Six samples were used, with ages ranging from 6yrs 6 months through to 89 yrs. 384 adult normative subjects were 20 yrs or older (mean = 49.9 yrs, SD = 17.9).  The average education in years for adults (M, SD, range) = 14.95, 2.97, no range given. Education-corrected normative data are provided.  Sex (% women) This varied across the subsamples and the average was not given (probably about the same).  Country: USA (race was not provided for the whole sample, but this was 87% white for the school-aged children).  Add the type of standardized score.  T-score, Standard score, and %ile are provided.  Add the language of translation and group characteristics:  The average age in years (M, SD, range)  The average education in years (M, SD, range)  Sex (% women)  Country  The test has been translated into many languages and normative data provided.  For example, these include Argentinian, Italian, Lebanese, Spanish, Spanish (and African-) American, Portuguese, Taiwanese (Del Pino et al. 2016; Faustino et al., 2020; Laiacona et al., 2000; Miranda et al., 2020; Norman et al., 2011; Rammal et al., 2019; Shan et al., 2008).  Will have to get details from these papers if required.  Not yet found if these translations have been used in the PD population. |

| **Responsiveness: assessed –good; not good. Not assessed (reference)** |  |
| --- | --- |
| Demonstrated to be sensitive to change (change over time or due to treatment)*  * [determined by the strength of the relationship between change in the test scores and the criterion or anchor scores] | If Yes, please specify  Little indication that this test is sensitive to change related to general treatments. One example is Gamito et al. (2019). This study reported that cognitive training using information-based technology (apps) produced significant improvement in some elderly subjects.  In PD patients with depression, repetitive transcranial magnetic stimulation or fluoxetine was found to improve WCST over 8 weeks (Boggio et al., 2005).  In general, withdrawal of dopamine medication enhances the WCST impairment in PD, although this may be related to worsening motor symptoms per se (Lange et al., 2018).  I have not found any evidence of change over time in PD patients, other than this may be a baseline measure for future PDD. In Galtier et al (2016), the combination of fluency and WCST as an executive function domain did not predict PDD 6yrs to 8 yrs after baseline assessment. |
| Has the minimal clinically important change and minimal clinically relevant incremental difference been assessed?  (besides a statistically significant improvement) | Minimal clinically important change assessed Y/N?  N |
| Has this test been assessed or used in patients with other condition than that of interest? | Numerous clinical groups have been investigated, including Huntington’s disease, Schizophrenia, Multiple Sclerosis, seizure disorders and patients with brain damage (focal and diffuse), as well as various neurodevelopmental disorders. |
| Other |  |
| **Overall impression** |  |
| Strengths | **Reliable**- The test has been used in various clinical and research applications as a measure of executive function.  **Valid-** The WCST has been significantly utilised in the PD population as well as many other clinical groups. |
| Weaknesses | There is concern that the standard 128 card version may be too long for many patients. Various other options are available, but the similar 64-card version may be most suitable. It is possible that the loss of general cognitive function may contribute to poor scores, in addition to any specific loss of cognitive flexibility and concept formation. The number of categories completed is often favoured as a key measure, but this has poor discrimination and floor effect in older participants. |

| **Level of Recommendation** |  |
| --- | --- |
| “Recommended” – The test was applied to the target population, AND was studied clinimetrically and found to be valid, reliable and sensitive to change, AND was used in studies by researchers, others than the developers  “Recommended with caveats” – Test’s properties were generally found to be adequate, but some of the measurement properties were not evaluated or not evaluated specifically in a PD cohort."  ‘Suggested’ – The test was applied to the target population, but only one of the other criteria applies  ‘Listed” – The test was applied to the target population, but none of the other criteria applies | Please choose one of these options  “Recommended” |
| Which type of study is this test suitable for and which type of study is it not suitable for (screening, prevalence, etiological (e.g. case-control or genetic), treatment trial, correlation with biological markers or other scales, e.g. of parkinsonism, clinical practice for diagnosis/ screening). List all. | Suitable for: All of this list  Screening  Prevalence  Differential reasoning (etiological)  Case-control study  Treatment trial  Biomarker study  Other |

**REFERENCES**

1. Ashendorf L, McCaffrey RJ. Exploring age-related decline on the Wisconsin Card Sorting Test. The Clinical Neuropsychologist. 2008 Mar 3;22(2):262-72.
2. Axelrod, B.N., Goldman, R.S., Heaton, R.K., et al. (1996). Discriminability of the Wisconsin Card Sorting Test using the standardization sample. Journal of Clinical and Experimental Neuropsychology, 18, 338–342.
3. Berg EA. A simple objective technique for measuring flexibility in thinking. The Journal of general psychology. 1948 Jul 1;39(1):15-22.
4. Boggio PS, Fregni F, Bermpohl F, Mansur CG, Rosa M, Rumi DO, Barbosa ER, Odebrecht Rosa M, Pascual‐Leone A, Rigonatti SP, Marcolin MA. Effect of repetitive TMS and fluoxetine on cognitive function in patients with Parkinson's disease and concurrent depression. Movement disorders: official journal of the Movement Disorder Society. 2005 Sep;20(9):1178-84.
5. Bowden SC, Fowler KS, Bell RC, Whelan G, Clifford CC, Ritter AJ, Long CM. The reliability and internal validity of the Wisconsin Card Sorting Test. Neuropsychological Rehabilitation. 1998 May 1;8(3):243-54.
6. Cropley VL, Fujita M, Bara-Jimenez W, Brown AK, Zhang XY, Sangare J, Herscovitch P, Pike VW, Hallett M, Nathan PJ, Innis RB. Pre-and post-synaptic dopamine imaging and its relation with frontostriatal cognitive function in Parkinson disease: PET studies with [11C] NNC 112 and [18F] FDOPA. Psychiatry Research: Neuroimaging. 2008 Jul 15;163(2):171-82.
7. del Pino R, Pena J, Ibarretxe-Bilbao N, Schretlen DJ, Ojeda N. Modified Wisconsin Card Sorting Test: Standardization and norms of the test for a population sample in Spain. Revista de neurologia. 2016 Mar 1;62(5):193-202.
8. Demakis, G.J. A meta-analytic review of the sensitivity of the Wisconsin Card Sorting Test to frontal and lateralized frontal brain damage. Neuropsychology **2003**, 17, 255–264.
9. Diamond, A. Executive functions. Annu. Rev. Psychol. **64**, 135–168 (2013).
10. Dias R, Robbins TW, Roberts AC. Dissociation in prefrontal cortex of affective and
11. attentional shifts. Nature 1996;380:69–72.
12. Faustino B, Oliveira J, Lopes P. Normative scores of the Wisconsin Card Sorting Test in a sample of the adult Portuguese population. Applied Neuropsychology: Adult. 2020 Sep 4:1-8.
13. Fortuny, L. i A., & Heaton, R. K. (1996). Standard versus computerized administration of the wisconsin card sorting test. The Clinical Neuropsychologist, 10(4), 419–424.
14. Friedman NP, Robbins TW. The role of prefrontal cortex in cognitive control and executive function. Neuropsychopharmacology. 2021 Aug 18:1-8.
15. Galtier I, Nieto A, Lorenzo JN, Barroso J. Mild cognitive impairment in Parkinson’s disease: Diagnosis and progression to dementia. Journal of clinical and experimental neuropsychology. 2016 Jan 2;38(1):40-50.
16. Gamito P, Oliveira J, Morais D, Coelho C, Santos N, Alves C, Galamba A, Soeiro M, Yerra M, French H, Talmers L. Cognitive stimulation of elderly individuals with instrumental virtual reality-based activities of daily life: pre-post treatment study. Cyberpsychology, behavior, and social networking. 2019 Jan 1;22(1):69-75.
17. Gardizi E, King JP, McNeely HE, Vaz SM. Comparability of the WCST and WCST-64 in the assessment of first-episode psychosis. Psychological assessment. 2019 Feb;31(2):271.
18. Greve KW, Brooks J, Crouch JA, Williams MC, Rice WJ. Factorial structure of the Wisconsin Card Sorting Test. British Journal of Clinical Psychology. 1997 May;36(2):283-5.
19. Greve KW, Bianchini KJ, Hartley SM, Adams D. The Wisconsin Card Sorting Test in stroke rehabilitation: Factor structure and relationship to outcome. Archives of Clinical Neuropsychology. 1999 Aug 1;14(6):497-509.
20. Greve KW. The WCST-64: A standardized short-form of the Wisconsin Card Sorting Test. The Clinical Neuropsychologist. 2001 May 1;15(2):228-34.
21. Greve KW, Stickle TR, Love JM, Bianchini KJ, Stanford MS. Latent structure of the Wisconsin Card Sorting Test: a confirmatory factor analytic study. Archives of Clinical Neuropsychology. 2005 May 1;20(3):355-64.
22. Guarino A, Favieri F, Boncompagni I, Agostini F, Cantone M, Casagrande M. Executive functions in Alzheimer disease: a systematic review. Frontiers in Aging Neuroscience. 2019 Jan 15;10:437
23. Heaton, R. K., Chelune, G. J., Talley, J. L., Kay, G., & Curtiss, G. (1993). Wisconsin Card sorting Test manual: Revised and expanded. Psychological Assessment Resources.
24. Heaton, R.K., & Thompson, L.L. (1992). Wisconsin Card Sorting Test: Is one deck as good as two? Journal of Clinical and Experimental Neuropsycho-logy,14, 63.
25. Heaton, R.K., & PAR Staff. (1999). Wisconsin Card Sorting Test: Computer Version 3 for Windows: Research edition. Odessa, FL: Psychological Assessment Resources.
26. Heaton, R.K., & PAR Staff. (2000). WCST-64: Computer Version 3 for Windows-Research Edition.Odessa, FL: Psychological Assessment Resources.
27. Heaton RK, Akshoomoff N, Tulsky D, Mungas D, Weintraub S, Dikmen S, Beaumont J, Casaletto KB, Conway K, Slotkin J, Gershon R. Reliability and validity of composite scores from the NIH Toolbox Cognition Battery in adults. Journal of the International Neuropsychological Society. 2014 Jul;20(6):588-98.
28. Kopp B, Maldonado N, Scheffels JF, Hendel M, Lange F. A meta-analysis of relationships between measures of Wisconsin card sorting and intelligence. Brain sciences. 2019 Dec;9(12):349.
29. Kopp B, Steinke A, Bertram M, Skripuletz T, Lange F. Multiple levels of control processes for Wisconsin Card Sorts: An observational study. Brain sciences. 2019 Jun;9(6):141.
30. Kopp B, Lange F, Steinke A. The reliability of the Wisconsin card sorting test in clinical practice. Assessment. 2021 Jan;28(1):248-63.
31. Laiacona M, Inzaghi MG, De Tanti A, Capitani E. Wisconsin card sorting test: a new global score, with Italian norms, and its relationship with the Weigl sorting test. Neurological Sciences. 2000 Dec;21(5):279-91.
32. Lange F, Brückner C, Knebel A, Seer C, Kopp B. Executive dysfunction in Parkinson’s disease: A meta-analysis on the Wisconsin Card Sorting Test literature. Neuroscience & Biobehavioral Reviews. 2018 Oct 1;93:38-56.
33. Leroi I, Pantula H, McDonald K, Harbishettar V. Neuropsychiatric symptoms in Parkinson's disease with mild cognitive impairment and dementia. Parkinson’s Disease. 2012 May;2012.
34. Lezak, M. D., Howieson, D. B., Bigler, E. D., & Tranel, D. (2012). Neuropsychological assessment (5th ed.). New York, NY: Oxford University Press.
35. Liozidou A, Potagas C, Papageorgiou SG, Zalonis I. The role of working memory and information processing speed on Wisconsin card sorting test performance in Parkinson disease without dementia. Journal of geriatric psychiatry and neurology. 2012 Dec;25(4):215-21.
36. Marras C, Tröster AI, Kulisevsky J, Stebbins GT. The tools of the trade: a state of the art “How to Assess Cognition” in the patient with Parkinson's disease. Movement Disorders. 2014 Apr 15;29(5):584-96.
37. Milner B. Effects of different brain lesions on card sorting: the role of the frontal
38. lobes. Arch Neurol. 1963;9:90–100.
39. Miranda AR, Franchetto Sierra J, Martínez Roulet A, Rivadero L, Serra SV, Soria EA. Age, education and gender effects on Wisconsin card sorting test: standardization, reliability and validity in healthy Argentinian adults. Aging, Neuropsychology, and Cognition. 2020 Nov 1;27(6):807-25.
40. Miyake, A.; Friedman, N.P.; Emerson, M.J.; Witzki, A.H.; Howerter, A.; Wager, T.D. The Unity and Diversity of Executive Functions and Their Contributions to Complex “Frontal Lobe” Tasks: A Latent Variable Analysis. Cogn. Psychol. **2000**, 41, 49–100.
41. Norman MA, Moore DJ, Taylor M, Franklin Jr D, Cysique L, Ake C, Lazarretto D, Vaida F, Heaton RK, Hnrc Group. Demographically corrected norms for African Americans and Caucasians on the hopkins verbal learning test–revised, brief visuospatial memory test–revised, stroop color and word test, and Wisconsin card sorting test 64-card version. Journal of clinical and experimental neuropsychology. 2011 Aug 1;33(7):793-804.
42. Nyhus E, Barceló F. The Wisconsin Card Sorting Test and the cognitive assessment of prefrontal executive functions: a critical update. Brain and cognition. 2009 Dec 1;71(3):437-51.
43. Paolo, A.M., Troster, A.I., Axelrod, B.N., Koller, W.C., 1995. Construct validity of the WCST in normal elderly and persons with Parkinson's disease. Archives of Clinical Neuropsychology, 10, 463–473.
44. Petkus AJ, Filoteo JV, Schiehser DM, Gomez ME, Hui JS, Jarrahi B, McEwen S, Jakowec MW, Petzinger GM. Mild cognitive impairment, psychiatric symptoms, and executive functioning in patients with Parkinson's disease. International journal of geriatric psychiatry. 2020 Apr;35(4):396-404.
45. Petrova M, Raycheva M, Traykov L. Cognitive profile of the earliest stage of dementia in Parkinson’s disease. American Journal of Alzheimer's Disease & Other Dementias®. 2012 Dec;27(8):614-9.
46. Petrova M, Mehrabian-Spasova S, Aarsland D, Raycheva M, Traykov L. Clinical and neuropsychological differences between mild Parkinson's disease dementia and dementia with Lewy bodies. Dementia and geriatric cognitive disorders extra. 2015;5(2):212-20.
47. Rabi R, Vasquez BP, Alain C, Hasher L, Belleville S, Anderson ND. Inhibitory control deficits in individuals with amnestic mild cognitive impairment: A meta-analysis. Neuropsychology review. 2020 Mar;30(1):97-125.
48. Rabin LA, Barr WB, Burton LA. Assessment practices of clinical neuropsychologists in the United States and Canada: A survey of INS, NAN, and APA Division 40 members. Archives of Clinical Neuropsychology. 2005 Jan 1;20(1):33-65.
49. Rammal S, Abi Chahine J, Rammal M, Fares Y, Abou Abbas L. Modified Wisconsin Card Sorting Test (M-WCST): Normative data for the lebanese adult population. Developmental neuropsychology. 2019 Jul 4;44(5):397-408. – also Portuguese, Taiwanese, Argentinian etc – see within this paper.
50. Schretlen, D. J. (2010). Modified Wisconsin Card Sorting Test (M-WCST): Professional manual. Lutz, FL: Psychological Assessment Resources.
51. Shan, I., Chen, Y., Lee, Y., & Su, T. (2008). Adult normative data of the Wisconsin Card Sorting Test in Taiwan. Journal of the Chinese Medical Association, 71(10), 517–522.
52. Steinke A, Lange F, Kopp B. Parallel model-based and model-free reinforcement learning for card sorting performance. Scientific reports. 2020 Sep 22;10(1):1-8.
53. Zubicaray GD, Ashton R. Nelson's (1976) modified card sorting test: A review. The Clinical Neuropsychologist. 1996 Jul 1;10(3):245-54.

**Rating test evaluation**

| **Test name: Verbal Fluency: Letter Fluency and Category Fluency** | Verbal Fluency/Controlled Oral Word Association Test (COWAT) **Other names:** Controlled Oral Word Association (COWA), Verbal Associative Fluency Test; Controlled Word Association Test; Word Fluency, Letter Fluency, FAS-Test, Category Fluency, Phonemic Fluency, Semantic Fluency, Controlled, Verbal Fluency, and Thurstone Word Fluency Test (Benton, 1994) |
| --- | --- |
| **Version**  Are there several versions (alternate or parallel) of the test? If so, which one has been assessed? | Yes, there are different letters (versions) in the Letter fluency AND different semantic categories in the Category fluency. All versions were assessed. |
| **Description of test**   - What population the test was originally developed for? - Which domains/components of domains do the test assess? - Are there multiple domains or a predominant domain assessed? - Does it score impaired/unimpaired? - Does it score severity   (subtle/mild/moderate/severe)? | - Patients with aphasia and language disorders - Several: word production, verbal fluency, word search, semantic memory, mental lexicon, mental flexibility, and retrieval from semantic memory. - Multiple domains are assessed. - Yes, it does score impaired/unimpaired in the case cut-off values were introduced. - No, it does not score severity. |
| **Scaling Metric**: Type of response format? (e.g., Likert, Binary, multiple-choice, continuum, reaction time, etc.) | The number of reaction times (defined as spontaneous production of words under restricted conditions) in 60 seconds (or in shorter or larger time intervals) is registered. |
| **Respondent: Patient:** any specific requirements for the target study population (e.g. non-demented, motor, auditory, visual abilities, etc)? | n.a. |
| **Access:** How can the test be obtained (mail address or website)? | Phonemic fluency tasks are part of the Multilingual Aphasia Examination (CFL and PRW; (Benton, 1994); the original COWAT is from Benton and Hamsher (1989), Neurosensory Center Comprehensive Examination for Aphasia; NCCEA-FAS (Spreen & Benton, 1977), the D-KEFS, KBNA, and the NEPSY. Norms for FAS and Animal Fluency are also sold by PAR ([www.parinc.com](http://www.parinc.com)) or available by (Gladsjo, Schuman, Evans, et al., 1999; Gladsjo, Schuman, Miller, et al., 1999; Heaton et al., 2004). |
| Copyrighted or in the public domain?  Conditions for use? Fee? | Free of charge OR fees to buy test normative data or batteries in which Verbal Fluency is one of the measures |
| **Translations:** Has the test been translated and validated in other languages besides English? | Yes: in many languages besides English: Spanish, Italian, German, French, Dutch, Chinese (Cantonese), Czech, Hebrew, Greek and in several other languages. |

| **Test properties**  (Please provide references for all statements or note if this is your judgment) |  |
| --- | --- |
| Which cognitive domain (or component/specific aspect of a cognitive domain) does this test *primarily* measure? | Letter Fluency: Executive (search for words beginning with a designated letter)  Category fluency: Executive (organize clusters of meaningfully related words) |
| Which aspects of the domain you are assessing are not covered? | n.a. |

| **Use** |  |
| --- | --- |
| Was it *designed* to measure the severity, screen or diagnosis of the domain? | Severity of the domain |
| Is there a cut-off score? Are there measures of screening/diagnostic performance of the test? (Receiver characteristic curve analyses; sensitivity/specificity; Positive Predictive Value/Negative Predictive Value), True Positive Rate, False Positive Rate etc.) | The cut-off score in relation to the diagnostic unit (MCI/dementia) is not present. Scores are compared to a healthy control group stratified for age and education. |
| Is this appropriate for the PD population? If not, why (low sensitivity and/or specificity values)? | Yes |
| **Clinimetric/psychometric properties in the target population** |  |
| **Feasibility /Acceptability** |  |
| Length | Letter Fluency: contain in the COWAT three word-naming trials (“F-A-S”) or (“C-F-L” or “P-R-W” in Multilingual Aphasia Examination). In other language, the letters may differ for the reason of differing frequencies of the initial letters. Total time length 4‒5 minutes.  Category fluency: contains usually the categories of animals, fruits, and vegetables. Other categories may be used as well (e.g., shopping). Total time length 4‒5 minutes. |
| Ambiguities in instructions to patient | N |
| Ambiguities in rating anchors | N |
| Appropriateness of questions for the population | n.a. |
| Applicability across disease cognitive stages | Yes: MCI/Dementia |
| Applicability across disease motor severity? | H&Y score: 1-4 applicable. |
| Clear instructions to raters | Y, see Strauss et al. (2006). |
| Has the test been used by researchers other than developers?  By other groups  Not applicable? | Y, the COWAT is used in almost every neuropsychological assessment |
| Are there instructions for dealing with missing data?  Not applicable? | Y, most often not applicable (the test person usually produces in 60 s more than one word), however, there are rules for inadmissible words: non-words, perseverations/repetitions, proper names or for wrong words not beginning with the letter (Letter Fluency) or out of category items (Semantic Fluency). |
| Floor and ceiling effects, skewed score distributions? | No floor and ceiling effects. Test scores do not show a normal distribution. |

| **Have the components of dimensionality, variability and dependency structures been analyzed?**  (Exploratory and confirmatory factor analysis (EFA and CFA)? | Y, a two-factor solution with markedly different loadings for semantic and phonological items (Schmidt et al., 2017). |
| --- | --- |
|  |  |
| **Reliability: assessed – good; not good. Not assessed**  Are alternative versions developed?  If so, were they validated with reliability measures? | Y, good. |
| Internal consistency (A rule of thumb)  0.9 ≤ Cronbach’s alpha (α) Excellent  0.8 ≤ α < 0.9 Good  0.7 ≤ α < 0.8 Acceptable  0.6 ≤ α < 0.7 Questionable  0.5 ≤ α < 0.6 Poor  α < 0.5 Unacceptable  (Streiner, 2003 Journal of Personality Assessment, 80:1, 99-103, DOI: 10.1207/S15327752JPA8001_18) | The degree of internal consistency among “F-A-S” and “C-F-L” was .83 (Tombaugh et al., 1999; Ruff et al., 1996) |
| “Corrected item-to-total correlation” | N.a. Letter and Semantic Fluency have both in each trial a different number of items |
| Test/retest | Y high test-retest reliability varying with test-retest interval and fluency version from .70 to 74 (Strauss et al., 2006) |
| Inter-rater | Y, .99 for “C-F-L” and .96 for clustering and .99 for switching in healthy subjects (Ross, 2003). |
| Intra-rater | N.a. |
| Other (e.g., Standard Error of Measurement) | N.a. |
| **Validity: assessed –good; not good. Not assessed (reference)** |  |
| Face and Content validity (does test appears to be measuring what it is intended to measure and do test’s items comprehensively represent the domain of interest?) | High. Both Letter and Semantic fluency are classical word production tasks and measure similar but different constructs (Schmidt et al., 2017). |
| Criterion validity (compared to gold-standard) or other criteria for  diagnosis) (e.g. other comparable tests or lesion or MRI research) | Good  Correlation with several other tests of verbal abilities (Strauss et al., 2006). |
| Construct validity (correlations with other convergent tests and divergent tests; known-groups comparisons) | Good  Correlations among Letter fluency tasks are high from .85 to .94.  Correlations among Semantic fluency tasks are moderate from .66 to .71.  Correlations between Letter and Semantic fluency are moderate from .34 to .64 (Strauss et al., 2006). |
| Reference group used to develop this test:   1. If available, please define the characteristic of the norm group adopted (demographic variables used for stratification: age, education, sex, etc). 2. What kind of standardized score generated (T score, Z score, derived IQ score, percentile ranks, etc)? 3. If translated/validated in other languages, are reference norms created? If so, please specify demographic variables used for stratification (age, education, gender etc). | 1) Many group norms were gathered in several countries/languages for several age and education groups. Varying from children (aged 7 and higher) to 80-96 years of age group. See Strauss et al. (2006) for details and best suitable comparison group with accompanying standardized scores.  Age, education, gender, reading level, ethnicity and IQ are important to take into account if available.  2) The most used standardized scores are percentiles or scaled scores (M = 10, SD = 3).  3) In other languages reference norms are stratified for demographic variables (age, education, gender etc.). |

| **Responsiveness: assessed –good; not good. Not assessed (reference)** |  |
| --- | --- |
| Demonstrated to be sensitive to change (change over time or due to treatment)*  * [determined by the strength of the relationship between change in the test scores and the criterion or anchor scores] | Yes, Letter and Semantic fluency are very sensitive to change over time (e.g., by the progression of the disease). |
| Has the minimal clinically important change and minimal clinically relevant incremental difference been assessed?  (besides a statistically significant improvement) | Y, significant cognitive changes using the RCI method was used for determining statistically meaningful change, see Tröster et al. (2007) |
| Has this test been assessed or used in patients with other condition than that of interest? | Y, in a wide range of neurological and other conditions. |
| Other | N.A. |
| **Overall impression** |  |
| Strengths | Adequate validity and reliability, are used in many studies including lesion and MRI studies. Many international groups use the Letter or Semantic fluency and have developed norm groups for the determination of severity of impairment. |
| Weaknesses | SD is broad. Determining change in individual cases is difficult due to broad RCIs (see Tröster et al. (2007). |

| **Level of Recommendation** |  |
| --- | --- |
| “Recommended” – The test was applied to the target population, AND was studied clinimetrically and found to be valid, reliable and sensitive to change, AND was used in studies by researchers, other than the developers  “Recommended with caveats” – Test’s properties were generally found to be adequate, but some of the measurement properties were not evaluated or not evaluated specifically in a PD cohort."  ‘Suggested’ – The test was applied to the target population, but only one of the other criteria applies  ‘Listed” – The test was applied to the target population, but none of the other criteria applies | Recommended |
| Which type of study is this test suitable for and which type of study is it not suitable for (screening, prevalence, etiological (e.g. case-control or genetic), treatment trial, correlation with biological markers or other scales, e.g. of parkinsonism, clinical practice for diagnosis/ screening). List all. | Suitable for:  Screening  Prevalence  Differential reasoning (etiological)  Treatment trial  Biomarker study  Other  Unsuitable for (please add just categories for which the measure is critically unsuitable):  Case-control study  Treatment trial with small number of patients. |

**REFERENCES**

1. Benton AL, & Hamsher K. Multilingual Aphasia Examination. AJA Associates 1989.
2. Benton AL, Hamsher K de S, Sivan AB. Multilingual Aphasia Examination (3rd Edition ed.). The Psychological Corporation 1994.
3. Gladsjo, JA, Schuman CC, Evans JD, Peavy GM, Miller SW, Heaton RK. Norms for letter and category fluency: demographic corrections for age, education, and ethnicity. Assessment 1999; 6: 147-178. <https://doi.org/10.1177/107319119900600204>
4. Gladsjo JA, Schuman CC, Miller SW, Heaton RK. Norms for letter and category fluency: Demographic corrections for age, education, and ethnicity. PAR 1999.
5. Heaton RK, Miller SW, Taylor MJ, Grant I. Revised comprehensive norms for an expanded Halstead-Reitan battery: Demographically adjusted neuropsychological norms for African American and Caucasian adults. PAR 2004.
6. Ross TP. The reliability of cluster and switch scores for the Controlled Oral Word Association Test. Arch Clin Neuropsychol 2003; 18: 153-164.
7. Schmidt CSM, Schumacher LV, Römer P, Leonhart R, Beume L, Martin M, Dressing A, Weiller C, Kaller CP. (2017). Are semantic and phonological fluency based on the same or distinct sets of cognitive processes? Insights from factor analyses in healthy adults and stroke patients. Neuropsychologia 2017; 99: 148-155. <https://doi.org/https://doi.org/10.1016/j.neuropsychologia.2017.02.019>
8. Spreen O, Benton AL. Neurosensory Center Comprehensive Examination for Aphasia. Neuropsychology Laboratory, University of Victoria 1977.
9. Strauss E, Sherman EMS, Spreen O. A compendium of neuropsychological tests: Administration, norms, and commentary, 3rd ed. Oxford University Press 2006.
10. Tröster AI, Woods SP, Morgan, EE. Assessing cognitive change in Parkinson's disease: Development of practice effect-corrected reliable change indices. Archives of Clinical Neuropsychology 2007; 22: 711-718. <https://doi.org/https://doi.org/10.1016/j.acn.2007.05.004>

**Attention/working memory domain:** Recommended with caveats, Suggested and Listed neuropsychological tests including their psychometric properties.

**Rating test evaluation**

| **Test name: Letter-Number-Sequencing (LNS) subtest from WAIS-IV** | Responses |
| --- | --- |
| **Version**  Are there several versions (alternate or parallel) of the test? If so, which one has been assessed? | Letter-Number Sequencing (LNS) is a subtest of the Wechsler Adult Intelligence Scale, 4^th^ edition (WAIS-IV) (1, 2), with a previous version included in the WAIS-III. Some current research still uses this older version. There are no alternate/parallel versions available for WAIS-IV. |

| **Description of test**   - What population the test was originally developed for? - Which domains/components of domains do the test assess? - Are there multiple domains or a predominant domain assessed? - Does it score impaired/unimpaired? - Does it score severity   (subtle/mild/moderate/severe)? | - Subtest from WAIS-IV, which was developed to evaluate intelligence and cognition in adolescents and adults (16 to 90 years) in both cognitively normal and clinical populations - The LNS subtest evaluates primarily auditory attention, concentration and working memory - Does not explicitly score impaired/ unimpaired - Score is based on severity since higher scores reflect better cognitive performance. Raw scores can be converted to scaled scores, which can be converted to *z* scores and percentile ranks, allowing to compare the performance with the normative population. Regarding the ‘severity qualitative descriptors’, there are some controversies based on the applied validation/standardization[[10]](#footnote-10) |
| --- | --- |
| **Scaling Metric**: Type of response format? (e.g., Likert, Binary, multiple-choice, continuum, reaction time, etc.) | Each item requires a binary response (0-1 points) based on the correctness of the response.  The total raw score (= sum of correct responses) can be converted to an age-corrected scaled score (*M* = 10; *SD* = 3). |
| **Respondent: Patient:** any specific requirements for the target study population (e.g. non-demented, motor, auditory, visual abilities, etc)? | This test is an auditory measure and thus requires hearing sufficient to understand the examiner, and language skills sufficient to comprehend the test instructions and to repeat letter-number strings. The test can also be influenced by anxiety, ADHD, and learning disabilities. |
| **Access:** How can the test be obtained (mail address or website)? | Pearson serves most of the international service orders and products ([pearsonassessments.com](file:///Users/bchol/Library/Containers/com.apple.mail/Data/Library/Mail%20Downloads/ADD4FEF0-9065-4F6E-9351-6D4CB21063C5/pearsonassessments.com)). However, some countries have to refer to different publishers (e.g., Italy: [giuntipsy.it](file:///Users/bchol/Library/Containers/com.apple.mail/Data/Library/Mail%20Downloads/ADD4FEF0-9065-4F6E-9351-6D4CB21063C5/giuntipsy.it)) |
| Copyrighted or in public domain?  Conditions for use? Fee? | Copyrighted; the full WAIS-IV must be purchased. The fee is quite expensive about $1,500/ € 2,000. |
| **Translations:** Has the test been translated and validated in other languages besides English? | Yes. WAIS-IV has been translated in several languages[[11]](#footnote-11): English (US), English (Australia and New Zealand), English (Canada), English (UK), Chinese, Danish, Dutch, Finnish, French (Canada), French (France), German, Greek, Hebrew, Hungarian, Icelandic, Italian, Japanese, Korean, Lithuanian, Mandarin (Taiwan), Norwegian, Polish, Portuguese (Brazil), Spanish (Argentina), Spanish (Chile), Spanish (Mexico), Spanish (Spain), Swedish as well as Indonesian.  Some versions are translated, language adapted, and/or have local normative data. |

| **Test properties**  (Please provide references for all statements or note if this is your judgment) | |
| --- | --- |
| Which cognitive domain (or component/specific aspect of a cognitive domain) does this test *primarily* measure? | Auditory attention and working memory domains. In the WAIS-IV, this subtest is included as a supplemental Working Memory test for ages 16-69 |
| Which aspects of the domain you are assessing are not covered? | Visual working memory, simple visual and auditory attention, response inhibition, abstract reasoning and planning |

| **Use** |  |
| --- | --- |
| Was it *designed* to measure severity, screen or diagnosis of the domain? | It was designed to measure severity of auditory working memory deficits. If the raw score is converted into the scaled score, this allows comparison of the individual performance to the normative population (in terms of *z-*scores/percentile ranks). |
| Is there a cut-off score? Are there measures of screening/diagnostic performance of the test? (Receiver characteristic curve analyses; sensitivity/specificity; Positive Predictive Value/Negative Predictive Value), True Positive Rate, False Positive Rate etc.) | The test manual does not provide cut-off scores; rather, age-corrected scaled scores can be converted to *z-*scores/percentiles. No values given for diagnosis/screening. |
| Is this appropriate for the PD population? If not, why (low sensitivity and/or specificity values)? | Yes |
| **Clinimetric/psychometric properties in the target population** | |
| **Feasibility /Acceptability** |  |
| Length | Number of items: LNS is composed of 10 items, which have 3 levels each. The total maximum score is 30.  Average range: 5-10 minutes, including  the instructions and the example and practical items. Less time, if the discontinuation criterion is met (3 failures within the same item) |
| Ambiguities in instructions to patient | No |
| Ambiguities in rating anchors | No |
| Appropriateness of questions for population | Yes |
| Applicability across disease cognitive stages | Normal cognition, MCI, and early dementia |
| Applicability across disease motor severity? | H&Y: 1-4  Appropriate for all stages of motor disease as it does not require intact motor function for completion and is not a timed task. May be impacted if dysarthria is severe or if the participant has an excessively soft voice. |
| Clear instructions to raters | Yes, standard instructions for administration and scoring are included in the test manual. |
| Has the test been used by researcher other than developers?  By other groups  Not applicable? | Yes, widely used in both PD and non-PD research protocols |
| Are there instructions for dealing with missing data?  Not applicable? | Discontinue rule states that if participant fails all three items in the same trial, test should be discontinued as it is unlikely the participant would be able to attain the next level. Thus, the following scores are treated as scores of 0 rather than missing data. |
| Floor and ceiling effects, skewed score distributions? | Normalized distribution |

| **Have the component of dimensionality, variability and dependency structures been analyzed?**  (Exploratory and confirmatory factor analysis (EFA and CFA)? | Yes[[12]](#footnote-12). CFA confirms loading on working memory index, with LNS loading .60 on this index (1, 3). |
| --- | --- |
|  |  |
| **Reliability: assessed – good; not good. Not assessed**  Are alternative versions developed?  If so, were they validated with reliability measures? | Assessed – good, reported in the WAIS-IV Technical and Interpretive Manual (1).  However, data are only reported for ages up to 69; no reliability/validity/norms information for older age groups provided in the WAIS-IV manual. Others have found this is a valid and reliable test to administer to older age groups and normative data may be available for some languages (4).  No alternate forms available. |
| Internal consistency (5)  0.9 ≤ Cronbach’s alpha (α) Excellent  0.8 ≤ α < 0.9 Good  0.7 ≤ α < 0.8 Acceptable  0.6 ≤ α < 0.7 Questionable  0.5 ≤ α < 0.6 Poor  α < 0.5 Unacceptable | .81 - .88 average across ages 16-69 (1, 3) |
| “Corrected item-to-total correlation” | No |
| Test/retest | Yes, short-term test-retest stability was investigated for 298 individuals from four age groups with retest intervals ranging from 8–82 days (mean retest interval of 22 days)  Average across age-ranges: .76 (.80 after correcting for the variability of the sample) (1). |
| Inter-rater | Excellent.  Interscorer agreement was examined by comparing two independent scorers of all WAIS-IV standardization record forms.  All WAIS-IV subtests are .98 to .99(1) |
| Intra-rater | No |
| Other (e.g. Standard Error of Measurement) | Yes, average across age-ranges: 1.03 - 1.29 (1, 3). |
| **Validity: assessed –good; not good. Not assessed (reference)** | |
| Face and Content validity (does test appears to be measuring what it is intended to measure and do test’s items comprehensively represent the domain of interest?) | Good: WAIS-IV Technical and Interpretive Manual provides a discussion of content validity as determined by comprehensive literature and expert reviews (1). |
| Criterion validity (compared to gold-standard) or other criteria for  diagnosis) (e.g. other comparable tests or lesion or MRI research) | Compared to previous Wechsler Adult Intelligence Scales and Intelligence Scale for Children. |
| Construct validity (correlations with other convergent tests and divergent tests; known-groups comparisons) | Convergent validity: r=.66 with Digit Span, r=.51 with Arithmetic, and overall with the Working Memory Index r=.67 (1, 3). Reasonable convergent validity was also shown with RBANS index scores assessing Attention (1).  Divergent validity: lower correlations with processing speed, verbal comprehension and perceptual reasoning Indexes of the WAIS-IV: r=0.36, r=0.50 and 0.47 respectively (1, 3). |
| Reference group used to develop this test:   1. If available, please define the characteristic of the norm group adopted (demographic variables used for stratification: age, education, sex, etc). 2. What kind of standardized score generated (T score, Z score, derived IQ score, percentile ranks, etc)? 3. If translated/validated in other languages, are reference norms created? If so, please specify demographic variables used for stratification (age, education, gender etc) | Group characteristics: 2,200 examinees highly matching in age/sex/education/ US regions and ethnicity.  Age in years: Age range from 16 to 90 years; Analyzed while considering 13 age-groups (16-17, 18-19, 20-24, 25-29, 30-34, 35-44, 45-54, 55-64, 65-69, 70-74, 75-79, 80-84,85-90). Approximately 200 examinees per group, except in the oldest groups (ages 70-90) where the sample was about 100 examinees.  Education in years: Five groups: ≤8, 9-11, 12, 13-15, and ≥16 years.  Sex (% women): equal numbers of men and women in each age-group, except the five oldest groups, which were represented by more women than men (consistently with the 2005 US Census proportions).  Country: USA  Add the type of standardized score: scaled scores, z-scores and percentile rank.  WAIS-IV has been translated in many languages as previously reported. Some versions have local normative data. |

| **Responsiveness: assessed –good; not good. Not assessed (reference)** | |
| --- | --- |
| Demonstrated to be sensitive to change (change over time or due to treatment)*  * [determined by the strength of the relationship between change in the test scores and the criterion or anchor scores] | - NET-PD trial indicated LNS NOT sensitive to change over 12-18 months in PD (6). - Improvements in WAIS-IV-LNS, after 3 months of combined GPi + NBM stimulation in a PD-MCI patient (*single case study*) (Nombela et al. 2019). |
| Has the minimal clinically important change and minimal clinically relevant incremental difference been assessed?  (besides a statistically significant improvement) | No |
| Has this test been assessed or used in patients with other condition than that of interest? | Yes, several conditions |
| Other | WAIS-IV LNS score was significantly associated with cholinergic nuclei 1, 2 and 3 (Ch123), cholinergic nucleus 4 (Ch4) and neocortical densities at baseline and after 4-year from PD diagnosis (7).  Presence of APOE ε4 allele in PD was associated with lower performance in WAIS-IV LNS (8).  In PD-MCI, the LNS test scores correlated  with thickness in a restricted area comprising the postcentral, precentral, and superior parietal gyri (9).  In PD, an association between LNS and more severe gait impairments has been described (10).  Mean diffusivity increases within the cerebral cortex (a highly sensitive imaging indicator of early microstructural cortical damage) in the left frontal cortex correlated with a decrease in WAIS-IV LNS scores (11).  Punding in PD was selectively associated with lower scores on LNS (12).  *LRRK2* associated with better performance on LNS in PD (13). |
| **Overall impression** | |
| Strengths | This subtest from the WAIS-IV has strong normative data, based on a large sample, and strong clinimetric validation. Further, its instructions and example items are quick and easy to administer. There is no motor or timed component. It is available in many languages. It can be sensitive to subtle cognitive deficits in the early PD-stages. Verbal administration suitable in the context of marked motor deficits. |
| Weaknesses | No reliability/validity/norms data for patients over age 69 provided in the WAIS-IV manual. May be frustrating for older participants or with advancing dementia. Most research incorporates previous version (e.g., WAIS-III). |

| **Level of Recommendation** | |
| --- | --- |
| “Recommended” – The test was applied to the target population, AND was studied clinimetrically and found to be valid, reliable and sensitive to change, AND was used in studies by researchers, others than the developers  “Recommended with caveats” – Test’s properties were generally found to be adequate, but some of the measurement properties were not evaluated or not evaluated specifically in a PD cohort."  ‘Suggested’ – The test was applied to the target population, but only one of the other criteria applies  ‘Listed” – The test was applied to the target population, but none of the other criteria applies | Please choose one of these options:  “Recommended with caveats” |
| Which type of study is this test suitable for and which type of study is it not suitable for (screening, prevalence, etiological (e.g. case-control or genetic), treatment trial, correlation with biological markers or other scales, e.g. of parkinsonism, clinical practice for diagnosis/ screening). List all. | Suitable for:  Case-control study  Biomarker studies  Prevalence  Correlation with biological markers. Etc.  Clinical practice  Unsuitable for (please add just categories for which the measure is critically unsuitable):  Screening  Treatment trial (sensitivity to change not convincing) |

**REFERENCES**

1. Wechsler D**.** Wechsler Adult Intelligence Scale - Fourth Edition: Technical and Interpretive Manual. Bloomington, MN: NCS Pearson; 2008.

2. Wechsler D**.** Wechsler Adult Intelligence Scale-Fourth Edition (WAIS-IV). Bloomington, MN USA: NCS Pearson; 2008.

3. Orsini A, Pezzuti L**.** WAIS-4: Contributo alla taratura italiana (16-69 anni): Giunti OS; 2013.

4. Pezzuti L, Rossetti S**.** Letter-Number Sequencing, Figure Weights, and Cancellation subtests of WAIS-IV admisnitered to elders. Personailty and Individual Differences. 2017;104:352-6.

5. Streiner DL**.** Starting at the beginning: an introduction to coefficient alpha and internal consistency. J Pers Assess. 2003;80(1):99-103.

6. Schneider JS, Elm JJ, Parashos SA, Ravina BM, Galpern WR, Investigators N-P**.** Predictors of cognitive outcomes in early Parkinson disease patients: The National Institutes of Health Exploratory Trials in Parkinson Disease (NET-PD) experience. Parkinsonism Relat Disord. 2010;16(8):507-12.

7. Barrett MJ, Sperling SA, Blair JC, Freeman CS, Flanigan JL, Smolkin ME, et al. Lower volume, more impairment: reduced cholinergic basal forebrain grey matter density is associated with impaired cognition in Parkinson disease. J Neurol Neurosurg Psychiatry. 2019;90(11):1251-6.

8. Mata IF, Leverenz JB, Weintraub D, Trojanowski JQ, Hurtig HI, Van Deerlin VM, et al. APOE, MAPT, and SNCA genes and cognitive performance in Parkinson disease. JAMA Neurol. 2014;71(11):1405-12.

9. Pereira JB, Svenningsson P, Weintraub D, Bronnick K, Lebedev A, Westman E, et al. Initial cognitive decline is associated with cortical thinning in early Parkinson disease. Neurology. 2014;82(22):2017-25.

10. Kelly VE, Johnson CO, McGough EL, Shumway-Cook A, Horak FB, Chung KA, et al. Association of cognitive domains with postural instability/gait disturbance in Parkinson's disease. Parkinsonism Relat Disord. 2015;21(7):692-7.

11. Sampedro F, Martinez-Horta S, Marin-Lahoz J, Pagonabarraga J, Kulisevsky J**.** Longitudinal intracortical diffusivity changes in de-novo Parkinson's disease: A promising imaging biomarker. Parkinsonism Relat Disord. 2019;68:22-5.

12. Hinkle JT, Perepezko K, Mills KA, Pontone GM**.** Attentional dysfunction and the punding spectrum in Parkinson's disease. Parkinsonism Relat Disord. 2021;84:23-8.

13. Srivatsal S, Cholerton B, Leverenz JB, Wszolek ZK, Uitti RJ, Dickson DW, et al. Cognitive profile of LRRK2-related Parkinson's disease. Mov Disord. 2015;30(5):728-33.

**Rating test evaluation**

| **Test name:** | Symbol Digit Modalities Test |
| --- | --- |
| **Version**  Are there several versions (alternate or parallel) of the test? If so, which one has been assessed? | There is an oral version aimed to deal with the contribution of motor component during the task, however, there are no extensive validation/normative studies in the oral version so this review will focus on the original written version. |
| **Description of test**   - What population the test was originally developed for? - Which domains/components of domains do the test assess? - Are there multiple domains or a predominant domain assessed? - Does it score impaired/unimpaired? - Does it score severity   (subtle/mild/moderate/severe)? | Symbol Digit Modalities Test (SDMT)(1) measures divided attention, visual scanning, visual tracking, perceptual speed, motor speed, and memory(2). A coding key is presented, consisting of nine meaningless geometric designs each paired with a number. The subject is required to scan the key and write down the number corresponding to each design as rapidly as possible in 90 seconds. There is a practice period (10 boxes). The number of correct responses is recorded. The maximum score is 110.  It was developed as a fast-screening approach for cerebral dysfunction in both children and adults.  Because of the existence of normative data, it is possible to determine the severity and if performance is below or above normal range. |
| **Scaling Metric**: Type of response format? (e.g., Likert, Binary, multiple-choice, continuum, reaction time, etc.) | It is a total score based on the summatory of total correct responses. |
| **Respondent: Patient:** any specific requirements for the target study population (e.g. non-demented, motor, auditory, visual abilities, etc)? | Because of the visuomotor component of the task it is mandatory to take into account visual deficits and motor deficits that may contribute to performance. There is an oral version aimed to solve with the motor component of the task, but no extensive validation studies have been done in these oral versions. |
| **Access:** How can the test be obtained (mail address or website)? | It is copyrighted. Not free. Access through the different companies depending on the country |
| Copyrighted or in public domain?  Conditions for use? Fee? | Copyrighted |
| **Translations:** Has the test been translated and validated in other languages besides English? | Instructions provided in several languages. The task is not language-based so there is no need for translations |

| **Test properties**  (Please provide references for all statements or note if this is your judgment) |  |
| --- | --- |
| Which cognitive domain (or component/specific aspect of a cognitive domain) does this test *primarily* measure? | Psychomotor processing speed, although several components (sustained attention, immediate memory and visual scanning contributes to task performance) |
| Which aspects of the domain you are assessing are not covered? |  |

| **Use** |  |
| --- | --- |
| Was it *designed* to measure severity, screen or diagnosis of the domain? | Yes. |
| Is there a cut-off score? Are there measures of screening/diagnostic performance of the test? (Receiver characteristic curve analyses; sensitivity/specificity; Positive Predictive Value/Negative Predictive Value), True Positive Rate, False Positive Rate etc.) | Cut-off score in relation to the diagnostic unit (MCI/dementia)  No. |
| Is this appropriate for the PD population? If not, why (low sensitivity and/or specificity values)? | It has been extensively used in PD, MS and other movement disorders such as HD. Although the strong motor component that may interfere with the task, it has been recommended in several studies, and probably, there is a need to further understand if the sensitivity of this task in movement disorders is based on measuring psychomotor speed or is based on measuring subtle motor and oculomotor changes. |
| **Clinimetric/psychometric properties in the target population** |  |
| **Feasibility /Acceptability** |  |
| Length | 90 seconds |
| Ambiguities in instructions to patient | no |
| Ambiguities in rating anchors | No |
| Appropriateness of questions for population | Yes. There is no question, just instructions |
| Applicability across disease cognitive stages | MCI/Dementia. Yes, because during the first trials of examples the rater can identify if cognitive changes makes impossible to perform de task. |
| Applicability across disease motor severity? | H&Y score: 1 (Unilateral involvement only) 🡪 yes  H&Y score: 2 or 3(bilateral involvement with or without postural instability) 🡪 yes  H&Y score: ≥4(several disabilities or confined to bed) 🡪 probably no due to motor component |
| Clear instructions to raters | Y. Instructions are provided in the manual |
| Has the test been used by researcher other than developers?  By other groups  Not applicable? | Y. it has been used in several studies by many other groups(3, 4). |
| Are there instructions for dealing with missing data?  Not applicable? | Y, provided in the instructions |
| Floor and ceiling effects, skewed score distributions? | No floor/ceiling effects were seen in studies in multiple sclerosis and HD(5, 6). Floor effects can be expected in patients with extreme motor disability |

| **Have the component of dimensionality, variability and dependency structures been analyzed?**  (Exploratory and confirmatory factor analysis (EFA and CFA)? | NA |
| --- | --- |
|  |  |
| **Reliability: assessed – good; not good. Not assessed**  Are alternative versions developed?  If so, were they validated with reliability measures? | Yes, an alternative version was developed(7) and three other versions were classified as “reliable” in a study that aimed to assess the reliability and equivalence of five SDMT alternate forms(8). |
| Internal consistency (A rule of thumb)(9)  0.9 ≤ Cronbach’s alpha (α) Excellent  0.8 ≤ α < 0.9 Good  0.7 ≤ α < 0.8 Acceptable  0.6 ≤ α < 0.7 Questionable  0.5 ≤ α < 0.6 Poor  α < 0.5 Unacceptable | NA |
| “Corrected item-to-total correlation” | NA |
| Test/retest | Yes. Test-retest reliability was assessed in 123 young healthy adults in a 150 min interval(10). Half of the participants accomplished the same form in both occasions while the other half filled a different one. Reasonable test-retest reliabities (.70) for both conditions. Subjects that completed the same form revealed significant practice effects (p < .001, dz = 1.61) |
| Inter-rater | NA |
| Intra-rater | NA |
| Other (e.g. Standard Error of Measurement) | Y/N, if Y, specify how large |
| **Validity: assessed –good; not good. Not assessed (reference)** |  |
| Face and Content validity (does test appears to be measuring what it is intended to measure and do test’s items comprehensively represent the domain of interest?) |  |
| Criterion validity (compared to gold-standard) or other criteria for  diagnosis) (e.g. other comparable tests or lesion or MRI research) | Yes, against cognition, brain lesions and disease progression in multiple sclerosis and HD(11-13). |
| Construct validity (correlations with other convergent tests and divergent tests; known-groups comparisons) | Add data regarding:  Convergent validity  Divergent validity  Group-comparisons |
| Reference group used to develop this test:   1. If available, please define the characteristic of the norm group adopted (demographic variables used for stratification: age, education, sex, etc). 2. What kind of standardized score generated (T score, Z score, derived IQ score, percentile ranks, etc)? 3. If translated/validated in other languages, are reference norms created? If so, please specify demographic variables used for stratification (age, education, gender etc). | Group characteristics:   1. 1307 healthy adults from Omaha (Nebraska, US) divided in 6 age groups matched by sex: 18-24 years old, 25-34 years old, 35-44 years old, 45-54 years old, 55-64 years old and >65. In each age group, subjects were divided in two groups according to education level: <12 years of education and >12 years of education. 2. Z scores 3. Yes. Every country has its own studies:   Spanish(14)  Total sample: n=346  Sex (59.5% women)  Country = Spain  English(15)  Total sample: n=19.114  The average age in years (75, range 65 - 99)  The average education in years (range = 9 - 15)  Sex (56.7% women)  Country: Australia & US  Greek(16)  Total sample: n=460  The average age in years (M 41.7, SD 16.4)  The average education in years (M 11.4, SD 6.2)  Sex (58.2% women)  Country: Greece  Dutch(17)  Total sample: n=96  The average age in years (M 46, SD 10.6)  The average education in years (Median 15, range 6-18)  Sex (59.3% women)  Country: Netherlands |

| **Responsiveness: assessed –good; not good. Not assessed (reference)** |  |
| --- | --- |
| Demonstrated to be sensitive to change (change over time or due to treatment)*  * [determined by the strength of the relationship between change in the test scores and the criterion or anchor scores] | Yes |
| Has the minimal clinically important change and minimal clinically relevant incremental difference been assessed?  (besides a statistically significant improvement) | Minimal clinically important change assessed Y/N? Not in Parkinson’s disease |
| Has this test been assessed or used in patients with other condition than that of interest? | Yes, in multiple sclerosis and Huntington’s disease |
| Other |  |
| **Overall impression** |  |
| Strengths | Quick and easy to administer  Written and oral versions therefore suitable for patients with severe movement impairment.  Well recognized amongst neurodegenerative diseases |
| Weaknesses | Number of errors not used as an outcome  Lack of concurrent validity with similar measures in adults |

| **Level of Recommendation** |  |
| --- | --- |
| “Recommended” – The test was applied to the target population, AND was studied clinimetrically and found to be valid, reliable and sensitive to change, AND was used in studies by researchers, others than the developers  “Recommended with caveats” – Test’s properties were generally found to be adequate, but some of the measurement properties were not evaluated or not evaluated specifically in a PD cohort."  ‘Suggested’ – The test was applied to the target population, but only one of the other criteria applies  ‘Listed” – The test was applied to the target population, but none of the other criteria applies | “Recommended with Caveats” |
| Which type of study is this test suitable for and which type of study is it not suitable for (screening, prevalence, etiological (e.g. case-control or genetic), treatment trial, correlation with biological markers or other scales, e.g. of parkinsonism, clinical practice for diagnosis/ screening). List all. | Suitable for:  Screening  Prevalence  Case-control study  Treatment trial  Biomarker study  Unsuitable for (please add just categories for which the measure is critically unsuitable):  Differential reasoning (etiological) |

**REFERENCES**

1. Smith A**.** Symbol Digit Modalities Test: Manual: Western Psychological Services; 1973.

2. Shum DH, McFarland KA, Bain JD**.** Construct validity of eight tests of attention: Comparison of normal and closed head injured samples. Clinical Neuropsychologist. 1990;4(2):151-62.

3. Braisch J, Muche R, Rothenbacher D, Landwehrmeyer B, Long J, Orth M**.** Identification of symbol digit modality test score extremes in Huntington's disease. American Journal of Medical Genetics Part B: Neuropsychiatric Genetics. 2019;180.

4. Strober L, DeLuca J, Benedict RH, Jacobs A, Cohen JA, Chiaravalloti N, et al. Symbol Digit Modalities Test: A valid clinical trial endpoint for measuring cognition in multiple sclerosis. Mult Scler. 2019;25(13):1781-90.

5. Abreu D, Ware J, Georgiou-Karistianis N, Leavitt BR, Fitzer-Attas CJ, Lobo R, et al. Utility of Huntington's Disease Assessments by Disease Stage: Floor/Ceiling Effects. Front Neurol. 2021;12:595679.

6. Goldman MD, LaRocca NG, Rudick RA, Hudson LD, Chin PS, Francis GS, et al. Evaluation of multiple sclerosis disability outcome measures using pooled clinical trial data. Neurology. 2019;93(21):e1921-e31.

7. Hinton-Bayre A, Geffen G**.** Comparability, reliability, and practice effects on alternate forms of the Digit Symbol Substitution and Symbol Digit Modalities tests. Psychol Assess. 2005;17(2):237-41.

8. Benedict RH, Smerbeck A, Parikh R, Rodgers J, Cadavid D, Erlanger D**.** Reliability and equivalence of alternate forms for the Symbol Digit Modalities Test: implications for multiple sclerosis clinical trials. Mult Scler. 2012;18(9):1320-5.

9. Streiner DL**.** Starting at the beginning: An introduction to coefficient alpha and internal consistency. Journal of Personality Assessment. 2003;80(1):99-103.

10. Pereira DR, Costa P, Cerqueira JJ**.** Repeated Assessment and Practice Effects of the Written Symbol Digit Modalities Test Using a Short Inter-Test Interval. Arch Clin Neuropsychol. 2015;30(5):424-34.

11. Benedict RH, DeLuca J, Phillips G, LaRocca N, Hudson LD, Rudick R, et al. Validity of the Symbol Digit Modalities Test as a cognition performance outcome measure for multiple sclerosis. Mult Scler. 2017;23(5):721-33.

12. Schobel SA, Palermo G, Auinger P, Long JD, Ma S, Khwaja OS, et al. Motor, cognitive, and functional declines contribute to a single progressive factor in early HD. Neurology. 2017;89(24):2495-502.

13. Tabrizi SJ, Scahill RI, Owen G, Durr A, Leavitt BR, Roos RA, et al. Predictors of phenotypic progression and disease onset in premanifest and early-stage Huntington's disease in the TRACK-HD study: analysis of 36-month observational data. Lancet Neurol. 2013;12(7):637-49.

14. Pena-Casanova J, Quinones-Ubeda S, Quintana-Aparicio M, Aguilar M, Badenes D, Molinuevo JL, et al. Spanish Multicenter Normative Studies (NEURONORMA Project): norms for verbal span, visuospatial span, letter and number sequencing, trail making test, and symbol digit modalities test. Arch Clin Neuropsychol. 2009;24(4):321-41.

15. Ryan J, Woods RL, Britt CJ, Murray AM, Shah RC, Reid CM, et al. Normative Data for the Symbol Digit Modalities Test in Older White Australians and Americans, African-Americans, and Hispanic/Latinos. J Alzheimers Dis Rep. 2020;4(1):313-23.

16. Messinis L, Bakirtzis C, Kosmidis MH, Economou A, Nasios G, Anyfantis E, et al. Symbol Digit Modalities Test: Greek Normative Data for the Oral and Written Version and Discriminative Validity in Patients with Multiple Sclerosis. Arch Clin Neuropsychol. 2021;36(1):117-25.

17. Burggraaff J, Knol DL, Uitdehaag BMJ**.** Regression-Based Norms for the Symbol Digit Modalities Test in the Dutch Population: Improving Detection of Cognitive Impairment in Multiple Sclerosis? Eur Neurol. 2017;77(5-6):246-52.

**Rating test evaluation**

| **Test name and Assessor:**  PASAT  Prof Nicky Edelstyn, [n.edelstyn@keele.ac.uk](mailto:n.edelstyn@keele.ac.uk) | Responses (possible)  Most of the information provided in this review has been informed by material presented in:  Tombaug (2006). A comprehensive review of the Paced Auditory Serial Addition Test (PASAT). Archives of Clinical Neuropsychology 21 (2006) 53–76 |
| --- | --- |
| **Version**  Are there several versions (alternate or parallel) of the test?  If so, which one has been assessed? | YES: In the PASAT original version, called PASAT-244 (Gronwall, 1977), a series of 61 pseudo-random digits is presented through the auditory canal four times and each time the interval of presentation differs (2.4; 2.0; 1.6 and 1.2 sec).  With the aim of reducing the practice effect, Levin and colleagues (1987) proposed an alternative auditory version, called PASAT-200, which includes four different series of 50 digits, respectively presented with an inter-stimulus interval of 3.0, 2.4, 2.0 and 1.6 seconds.  Fos and colleagues (2000) created a visual modality version of PASAT, called Paced Visual Serial Addition Test (PVSAT), in which digits are visually presented and participants have to say the sum of the digits aloud.  Royan and colleagues (2004), created a computerized version of PASAT, called Adjusting-PSAT.  A recent virtual reality PASAT task (VR-PASAT) has also been developed (Parsons & Courtney, 2014).  The modified Levin’s PASAT has been assessed. The Levin’s version controls for possible within-session practice effects caused by using the same sequence of digits on each trial in the original version. It uses a shorter series of 50 digits (iii) and a different series of digits for each ISI (i.e, Inter-Stimulus Interval milliseconds).  Gronwall, D. & Sampson, H. (1974). The Paced Auditory Serial Addition Test (PASAT). The psychological effects of concussion. Auckland, New Zealand: Auckland University Press.  Levin, H. S., Mattis, S., Ruff, R., Eisenberg, H. M., Marshall, L. F., Tabaddor, K., et al. (1987). Neurobehavioral outcomes following minor head  injury: A three-center study. Journal of Neurosurgery, 66, 234–243. |
| **Description of test**   - What population the test was originally developed for? - Which domains/components of domains do the test assess? - Are there multiple domains or a predominant domain assessed? - Does it score impaired/unimpaired? - Does it score severity   (subtle/mild/moderate/severe)? | It was initially developed by Gronwall in 1977 to monitor the recovery of patients who had sustained mild head injuries.  Gronwall and Sampson (1974) originally assumed the PASAT measured auditory information processing speed and flexibility as well as calculation ability. It implies the engagement of working memory.  However, the PASAT is now recognized as a measure of multiple functional domains because it requires the successful completion of a variety of cognitive functions, primarily those related to attention.  Normative data is reported in four studies:   - Brittain et al. (1991). Effects of age and IQ on Paced Auditory Serial Addition Task (PASAT) performance. The Clinical Neuropsychologist, 5, 163–175.   145 persons aged <25  164 aged 25-39  95 aged 40-54  122 aged >55   - Roman et al. (1991). Extended norms for the Paced Auditory Serial Addition Task. The Clinical Neuropsychologist, 5, 33–40.   62 persons aged 18 to 27  40 aged 33 to 50  41 aged 60 to 75.   - Wiens et al. (1997). Paced Auditory Serial Addition Test: Adult norms and moderator variables. Journal of Clinical and Experimental Neuropsychology, 19, 473–483.   375 persons aged 20 to 29  295 aged 30-39  45 aged 40-49   - Diehr et al. (1998). The Paced Auditory Serial Addition Task (PASAT): Norms for age, education, and ethnicity. Assessment, 5, 375–387.   These normative data are inadequate for PD for various reasons: age at the top end is either not reported, heterogeneous or limited to a narrow age band (with a small n). IQ and mathematical ability impact on performance, but these moderators are not included. Finally, the Diehr’s study provides scaled scores and may address some of these limitations, but it is impossible to get hold of.  Both. |
| **Scaling Metric**: Type of response format? (e.g., Likert, Binary, multiple-choice, continuum, reaction time, etc.) | The score for the PASAT is the total number correct out of 60 possible answers (continuum). |
| **Respondent: Patient:** any specific requirements for the target study population (e.g. non-demented, motor, auditory, visual abilities, etc)? | NONE (patients with hearing loss or with hearing aids should be not administered the test). |
| **Access:** How can the test be obtained (mail address or website)? | Open access psychological software provides access. |
| Copyrighted or in public domain?  Conditions for use? Fee? | N/A  The PASAT is administered in person by a trained examiner. |
| **Translations:** Has the test been translated and validated in other languages besides English? | In Italy, normative data for PASAT have been previously published (Ciaramelli et al., 2006: 388 healthy individuals, 57% F, age range 14-76, with three educational levels, low-medium-high).  Saetti et al. (2021) have recently provided normative data for the Italian population using the standardized methodology of the equivalent scores. |

| **Test properties**  (Please provide references for all statements or note if this is your judgment) |  |
| --- | --- |
| Which cognitive domain (or component/specific aspect of a cognitive domain) does this test *primarily* measure? | Attention and working memory |
| Which aspects of the domain you are assessing are not covered? | Definite attentional processes are involved in the PASAT (Spikman et al., 1996): focal attention allows the distinction between the two digits-target and distractors; sustained attention aids concentration and reduces lapses; alertness influences the speed of response to each digit presentation. The central executive system also plays a prominent role during PASAT administration, as it allows for retention of digits while performing the test. |

| **Use** |  |
| --- | --- |
| Was it *designed* to measure severity, screen or diagnosis of the domain? | Screen for cognitive sequalae of TBI. It could be useful also for rehabilitation purposes (Serino et al., 2007). |
| Is there a cut-off score? Are there measures of screening/diagnostic performance of the test? (Receiver characteristic curve analyses; sensitivity/specificity; Positive Predictive Value/Negative Predictive Value), True Positive Rate, False Positive Rate etc.) | Scores less than the 5th percentile of the normal population are generally considered to be in the impaired range. |
| Is this appropriate for the PD population? If not, why (low sensitivity and/or specificity values)? | No: See Weaknesses.  Dujardin et al. (2007) stated that “*In clinical practice, the PASAT is not frequently used with PD patients. It is, however, among the tests recommended by the core assessment program for surgical interventional therapies in PD and its use has been proposed by some research teams for the evaluation of attention, working memory and processing speed in PD. However, a more accurate appraisal of the PASAT validity in this pathology seems necessary*”. |
| **Clinimetric/psychometric properties in the target population** |  |
| **Feasibility /Acceptability** |  |
| Length | The PASAT is presented using audio cassette tape or compact disk to ensure standardization in the rate of stimulus presentation. Single digits are presented every 3 seconds and the patient must add each new digit to the one immediately prior to it. Shorter inter-stimulus intervals, e.g., 2 seconds or less have also been used with the PASAT but tend to increase the difficulty of the task.  Administration time is approximately 10-15 minutes including practice sessions. |
| Ambiguities in instructions to patient | No. |
| Ambiguities in rating anchors | N/A |
| Appropriateness of questions for population | N/A |
| Applicability across disease cognitive stages | Yes (e.g.): Kawashima et al. (2020; 2021a) found that scores on the PASAT were lower in PD patients compared to HC and in PD-MCI compared to PD-NC (p < .05). |
| Applicability across disease motor severity? | H&Y score 1-4  Reuter et al., 2012  H&Y score: 2-3  Uc et al., 2006; Kawashima et al., 2020; 2021a; Kiesmann et al., 2013. |
| Clear instructions to raters | YES:  Manual and record form is available http://www.pasat.us/PDF/PASAT_Manual.pdf |
| Has the test been used by researcher other than developers?  By other groups  Not applicable? | YES |
| Are there instructions for dealing with missing data? | Yes. |
| Floor and ceiling effects, skewed score distributions? | YES: Ceiling effects reported at longer ISIs and floor effects at very short ISIs. |

| **Have the component of dimensionality, variability and dependency structures been analyzed?** | N/A |
| --- | --- |
|  |  |
| **Reliability: assessed – good; not good. Not assessed** | Reliability is high, but stability (test-retest consistency) is low due to practice effects. |
| Internal consistency (A rule of thumb) | A Cronbach alpha of .90 was obtained from scores on the four trials for the PASAT (Crawford et al., 1998)  Crawford, J. R., Obonsawin, M. C., & Allan, K. M. (1998). PASAT and components of WAIS-R performance: Convergent and discriminant validity. Neuropsychological Rehabilitation, 8, 255–272. |
| “Corrected item-to-total correlation” | N/A |
| Test/retest | Practice effects are present over a wide range of fixed ISIs (4.0 s, 3.0 s 2.4 s, 2.0 s, 1.6 s, 1.2 s), when threshold values are employed. The greatest practice effects occur between the first and second administration with relatively stable performance occurring after the second session.  One explanation for practice effects resides in the fact that the PASAT is a difficult and complex test. Due to this complexity, participants require time and practice to develop an effective strategy to perform on this type of test. Effective strategies are probably not fully developed until after the initial administration has been completed, and it is the general procedural knowledge about the test and strategies for effective performance that are retained and promote higher scores on subsequent administrations.  A second factor contributing to improved scores is the heightened emotional state that the PASAT induces. During the initial baseline performance, participants typically experience anxiety and frustration. However, the novelty of the test is diminished with repeated exposure, thereby reducing anxiety effects associated with the testing process. As a result, participants are more comfortable and better able to concentrate during repeated testing. |
| Inter-rater | N/A |
| Intra-rater | N/A |
| Other (e.g. Standard Error of Measurement) | N/A |
| **Validity: assessed –good; not good. Not assessed (reference)** |  |
| Face and Content validity (does test appears to be measuring what it is intended to measure and do test’s items comprehensively represent the domain of interest?) | YES: It is sensitive to speed of information processing, sustained attention, divided attention and working memory. |
| Criterion validity (compared to gold-standard) or other criteria for diagnosis) (e.g. other comparable tests or lesion or MRI research) | The relationship between PASAT and attentional processes has been confirmed by the correlation between the patient’s performance on PASAT and on other attentional tests, such as Digit Span Backward, Trail Making Test part B and Symbol Digit (Royan et al., 2004; Tombaugh, 2006).  Stimulus presentation rates of the PASAT were adapted for use with multiple sclerosis (MS) patients by Rao and colleagues (1989).  PET studies found that PASAT activates widespread, non-contiguous foci in the superior temporal gyri, bifrontal and biparietal sites and the anterior cingulated and bilateral cerebellar sites (Lockwood et al., 2004).  A similar distribution was found in fMRI and SPECT studies showing activation in the dorsolateral prefrontal cortex, right and left superior temporal gyrus, right and left medial frontal cortex and cerebellum (Hattori et al., 2009; Lazeron et al., 2003). |
| Construct validity (correlations with other convergent tests and divergent tests; known-groups comparisons) | The PASAT correlates with Digit symbol and Arithmetic subsets of the WAIS (Crawford et al., 1998). |
| Reference group used to develop this test:   1. If available, please define the characteristic of the norm group adopted (demographic variables used for stratification: age, education, sex, etc). 2. What kind of standardized score generated (T score, Z score, derived IQ score, percentile ranks, etc)? 3. If translated/validated in other languages, are reference norms created? If so, please specify demographic variables used for stratification (age, education, gender etc). | This information is not available, as the original research study was published as a book chapter, which I can’t get hold of.  Total correct (raw)/Percent correct.  Tables providing means and standard deviations for the total number of correct responses (maximum = 60) differentiated by education level (</> 12 yrs.) are reported.  146 healthy Italian individuals  Mean age and age range: 46.45±16.26, 18-83 Average education in years and range: 13.05± 4.05, 8-19 years.  Sex (52% women)  Country: Italy  For PASAT standardization, the Equivalent Scores (ES) procedure was followed (Capitani, 1997; Capitani & Laiacona, 1997; 2017). |

| **Responsiveness: assessed –good; not good. Not assessed (reference)** |  |
| --- | --- |
| Demonstrated to be sensitive to change (change over time or due to treatment)*  * [determined by the strength of the relationship between change in the test scores and the criterion or anchor scores] | No difference in the score of the PASAT between memantine intervention group and placebo group of a randomized double-blind cross-over design of PD-MCI patients (Kawashima et al., 2021b).  Reuter et al. (2012) found an improvement in PASAT score for PD patients after a combined cognitive training, transfer training and psychomotor and endurance training. Training was conducted for 4 weeks on a rehabilitation unit followed by 6 months training at home. |
| Has the minimal clinically important change and minimal clinically relevant incremental difference been assessed?  (besides a statistically significant improvement) | Not assessed. |
| Has this test been assessed or used in patients with other condition than that of interest? | YES: Chronic fatigue, TBI, rheumatism, neurological diseases which involve frontal or subcortical lesions in relatively young patients (< 65 years of age), Multiple Sclerosis and HIV infection (Moore et al., 2012; Rosti et al., 2006; Sonder et al., 2014). The PASAT has also been used in a variety of other clinical populations, including those with pain disorders (Sjøgren et al., 2000a), lupus erythematosus (Shucard et al., 2004), cancer (Sjøgren et al., 2000b), depression (Johnson et al., 1997), hypoglycemia (Gold et al., 1995) and asthma (Weersink et al., 1997). |
| Other | / |
| **Overall impression** |  |
| Strengths | The PASAT is a highly sensitive test. Indeed, with patients early in the course of PD and lacking overt cognitive decline, an impairment of PASAT performance is evident (Dujardin et al., 2007).  Two alternate forms have been developed. |
| Weaknesses | 1. In the opinion of Dujardin et al. (2007), despite its high sensitivity, results do not validate the PASAT as a measure of executive function in PD; 2. A low score on the PASAT does not necessarily indicate or confirm the presence of neurological pathology. Intellectual functioning, mathematic ability (i.e., calculation), and probably education (cf. Saetti et al., 2021) are moderators of performance and therefore these need to be taken into account when interpreting performance. 3. The effect of age on performance is equivocal. 4. Practice effects are present, with the greatest improvement occurring between trial 1 and trial 2. Therefore, Trial 2 performance should be used as a baseline when re-testing is required. Evidence suggests that practice effects are enduring – (evident at 6 months). It is unwise to generalize this procedure to all types of patients, and there is a need to determine which administration provides the best baseline for different   types of patients. In experimental situations the obvious solution is to use a control group that receives the same number of test administrations, and then “factor out” the effects of practice from the overall treatment effects.   1. Strategies such as chunking can be used to improve performance. 2. According to Dujardin e al. (2007), PD patients’ decline in PASAT performance is merely an index of cognitive slowing. |

| **Level of Recommendation** |  |
| --- | --- |
| “Recommended” – The test was applied to the target population, AND was studied clinimetrically and found to be valid, reliable and sensitive to change, AND was used in studies by researchers, others than the developers  “Recommended with caveats” – Test’s properties were generally found to be adequate, but some of the measurement properties were not evaluated or not evaluated specifically in a PD cohort.  ‘Suggested’ – The test was applied to the target population, but only one of the other criteria applies  ‘Listed” – The test was applied to the target population, but none of the other criteria applies | Please choose one of these options  Recommended with caveats. |

**REFERENCES**

1. Ciaramelli E, Serino A, Benassi M, et. al. Standardizzazione di tre test di memoria di lavoro. Italian J Psychol 2006; 33(3): 607-626.
2. Dujardin K, Denève C, Ronval M, et al. Is the paced auditory serial addition test (PASAT) a valid means of assessing executive function in Parkinson's disease? Cortex 2007; 43(5), 601-606.
3. Fos L, Greve K, South M, et al. Paced visual serial addition test: An alternative measure of information processing speed. Appl Neuropsy 2000; 7(3): 140-146.
4. Gold AE, MacLeod KM, Thomson KJ, et al. Cognitive function during insulin-induced hypoglycemia in humans: short-term cerebral adaptation does not occur. Psychopharmacol 1995; 119(3): 325- 333.
5. Gronwall DMA. Paced auditory serial-addition task: a measure of recovery from concussion. Percept Motor Skills 1977; 44(2): 367-373.
6. Johnson SK, Lange G, DeLuca J, et al. The effects of fatigue on neuropsychological performance in patients with chronic fatigue syndrome, multiple sclerosis, and depression. Appl Neuropsychol 1997; 4(3): 145-153.
7. Kawashima S, Shimizu Y, Ueki Y, et al. Visuospatial n-back test for early detection of mild cognitive impairment in patients with Parkinson’s Disease: an fMRI study. Biorxiv 2020;
8. Kawashima S, Shimizu Y, Ueki Y, et al (2021a). Impairment of the visuospatial, working memory in the patients with Parkinson’s Disease: an fMRI study. BMC Neurol 2021a; 21(1): 1-12.
9. Kawashima S, Matsukawa N, & RCIP-Nagoya Study Group. Memantine for the Patients with Mild Cognitive Impairment in Parkinson’s Disease: A Pharmacological fMRI Study. BMC Neurol 2021b; 13:175.
10. Moore DJ, Roediger MJ, Eberly LE, et al. (2012). Identification of an abbreviated test battery for detection of HIV-associated neurocognitive impairment in an early-managed HIV-infected cohort. PLoS One 2012; 7(11).
11. Parsons TD, Courtney CG. An initial validation of the virtual reality paced auditory serial addition test in a college sample. J Neurosci Methods, 2014; 222: 15-23.
12. Reuter I, Mehnert S, Sammer G. Efficacy of a multimodal cognitive rehabilitation including psychomotor and endurance training in Parkinson's disease. J Aging Res 2012; 2012:235765.
13. Rao SM, Leo GJ, Haughton VM, et al. Correlation of magnetic resonance imaging with neuropsychological testing in multiple sclerosis. Neurology 1989; 39: 161-166.
14. Rosti E, Hämäläinen P, Koivisto K. The PASAT performance among patients with multiple sclerosis: analyses of responding patterns using different scoring methods. Mult Scler J 2006; 12(5), 586-593.
15. Royan J, Tombaugh TN, Rees L. The Adjusting-Paced Serial Addition Test (Adjusting-PSAT): Thresholds for speed of information processing as a function of stimulus modality and problem complexity. Arch Clin Neuropsychol 2004; 19(1): 131-143.
16. Saetti MC, Difonzo T, Sartori MA, et al. The Paced Auditory Serial Addition Task (PASAT): normative data for the Italian population. Neuropsychol Trends 2021; (29): 65-82.
17. Serino A, Ciaramelli E, Santantonio AD, et al. A pilot study for rehabilitation of central executive deficits after traumatic brain injury. Brain Inj 2007; 21(1): 11-19.
18. Sjøgren P, Thomsen AB, Olsen A.K. Impaired neuropsychological performance in chronic nonmalignant pain patients receiving long-term oral opioid therapy. JPSM 2000a; 19(2): 100-108.
19. Sonder JM, Burggraaff J, Knol DL, et al. Comparing long-term results of PASAT and SDMT scores in relation to neuropsychological testing in multiple sclerosis. Mult Scler J 2014; 20(4): 481-488.
20. Spikman JM, Zomeren AHV, Deelman, BG. Deficits of attention after closed-head injury: Slowness only? J Clin Exp Neuropsychol 1996; 18(5): 755-767.
21. Uc EY, Rizzo M, Anderson SW, et al. Driving with distraction in Parkinson disease. Neurology 2006; 67(10): 1774-1780.
22. Weersink EJ, van Zomeren EH, Koeter GH, et al. Treatment of nocturnal airway obstruction improves daytime cognitive performance in asthmatics. AJRCCM 1997; 156(4): 1144-1150.

**Rating test evaluation**

| **Test name:** | **Corsi Block Tapping Test/Task (CBTT)** |
| --- | --- |
| **Version**  Are there several versions (alternate or parallel) of the test? If so, which one has been assessed? | Several versions, including the traditional three-dimensional physical version (with normally 9 blocks, but also 10 blocks across eft and right halves of the physical display), computerized versions (e.g. CANTAB Spatial Span). There is also a Modified Walking Corsi test (but the walking test emphasises navigation/path finding/topographic memory; Piccardi et al., 2013).  The primary focus here is the traditional physical CBTT, but computerised 2D and 3D versions were also considered (the latter produce similar scores; Arce and McMullen, 2021).  Similar physical and computerised Corsi tests produce similar results, at least in HC. |
| **Description of test**   - What population the test was originally developed for? - Which domains/components of domains do the test assess?      - Are there multiple domains or a predominant domain assessed? - Does it score impaired/unimpaired? - Does it score severity   (subtle/mild/moderate/severe)? | Epilepsy and right medial temporal lobe brain injury (Corsi, 1972; Milner, 1971).  Primarily Attention / visuospatial short-term memory (forwards span) and attention / visuospatial working memory (backwards span) – but see below.  Although focused on immediate visuospatial memory and attention, task characteristics also require memory for temporal (serial) order and memory for spatial path (configuration) learning (Busch et al., 2005; Zimmer, 2008). The latter function may explain instances of similar forwards and backwards span, although the difference between spans is often about 0.5SD on average in many studies (Kessels et al., 2008). Different demand characteristics may influence the relative importance of component processes. For example, path-finding may be more explicitly measured using walking Corsi versions (Piccardi et al., 2013).  Also, spatial span – especially backwards – can be regarded / used as an executive function / resource allocation measure (e.g. Horta-Barba et al., 2020; Kemps et al., 2005; Kulicka et al., 2011).  CANTAB Spatial span has also been described as a “fronto-executive” measure (e.g. Olde Dubbelink et al., 2014), but other work suggests more complicated brain networks are involved (Zimmer, 2008).  Impaired scores can be inferred from relative performance cut-offs, such as:  (a) -1.3SD (borderline / mild impairment) or -2SD (impaired / extremely low) below controls or standardised scores (e.g. Kessels et al., 2000);  (b) categorical data for each test defined a 0 to 3 scale (Aybeck et al., 2007):  0 _ normal performances (_C25: above the 25th percentile of the healthy population),  1 _ limited performances (_C10–C25: between the 25th and the 10th percentile of the healthy population);  2 _ moderate impairment (_C5–C10: between the 10th and the 5th percentile),  3 _severe impairment (_C5: below the 5th percentile).  (c) Equivalent Scores based on:  0 = scores lower than the 95 % tolerance limit;  1, 2 and 3 = scores lower than 10.4, 26.4 and 50% of the normative  sample distribution, respectively.  And 4 = scores higher than the median value of the sample.  However, different scores can be taken. The usual score is maximum correct span; also the total number (or percent) of spans or items correct (correct score) or a product score (span x number correct).  Through the use of cut-offs generated relative to norms or controls (see above). |
| **Scaling Metric**: Type of response format? (e.g., Likert, Binary, multiple-choice, continuum, reaction time, etc.) | Binary – correct (1) or incorrect items (0). A continuous score is used (highest span; number of items correct or percent correct). |
| **Respondent: Patient:** any specific requirements for the target study population (e.g. non-demented, motor, auditory, visual abilities, etc)? | Visual and motor requirements are not issues unless severe.  Clinician-based or researcher-based assessment: the patient has to watch the clinician’s / experimenter’s sequence of block tapping and repeat these manually (either forwards or backwards). Sometimes two sequences (trials) per span length are used and one (Kessels et al., 2000) or two correct longest lengths is taken as the span, which provides good specificity. Less frequently, four (trial) sequences are used (Kemps et al., 2005); more often, five sequences (trials) per length are used, and the participant progresses to a longer sequence if three correct, with three correct sequences taken as the span; it is suggested that this produces better sensitivity (Trojano et al., 1994).  In computerized versions, the 2D blocks often light up the sequence (e.g from light grey to a colour in the current CANTAB) and then the participant aims to repeat it.  In one computer version (eCorsi; Brunetti et al., 2014), one error is allowed for scoring the longest span, but this is rare for span tasks.  The demonstrated tapping rate is usually 1s per block, but sometimes 2s; in some computerised versions (CANTAB / Owen et al., 1992 version) each sequential target object stays lit for 3s (Note – I think this is the same for CANTAB and may be important for the findings reported by Olde Dubbelink et al., 2014). |
| **Access:** How can the test be obtained (mail address or website)? | Several versions available in the public domain through published papers.  Previously, a modified version used in the WAIS-R Neuropsychological Inventory and WMS-III (Kaplan et al., 1991), which used a 10-block version, with five blocks on each half of the board so as to permit an assessment of visual field neglect. It is suggested that in this version forwards and backwards may be the same due to a learning confound because the same sequence pairs are used across forwards and backwards conditions (Kessels et al., 2008). The WMS-R had two dimensional analogs of the block span task forward and backward with 9 blocks.  There is also a German version Schellig, D. (1997). *Block-Tapping test.* Frankfurt, Germany: Swets Test Services (but this uses smaller blocks; unable to find if this is still available.  Many computerised versions exist.  One is “Spatial Span” (as opposed to “Spatial Working Memory”) within the CANTAB battery of tests (<https://www.cambridgecognition.com/cantab/cognitive-tests/memory/spatial-span-ssp/>).  Also, a 3D computerised 9-block version that is similar to the original Corsi:  <https://www.schuhfried.com/vienna-test-system/> - the Vienna Test System includes Corsi Block tapping. https:/www.Millisecond.com offers English and German versions of the task (account required) |
| Copyrighted or in public domain?  Conditions for use? Fee? | Generally, in the public domain and descriptions in research studies apart from the physical (WMS-III) and computer “standardised” versions listed above.  CANTAB / Cambridge Brain Sciences may allow purchase of subtests, but I need to check (this was mentioned to me several years ago). CANTAB says it can provide 45 voice over languages.  The Corsi test is also available within the cognitive test system provided by <https://www.schuhfried.com/vienna-test-system/>, which has 20 language options. |
| **Translations:** Has the test been translated and validated in other languages besides English? | The test is relatively simple and instructions for various versions easily converted to local languages (see immediately above for two computerised versions). WMS-III and CANTAB have been validated, but not sure if this is true of the CANTAB language versions. Italian versions have also been validated (Spinnler, 1987; Orsini et al., 1987), but not sure if validated in non-Italian languages. The Italian version was used for example in a PD DBS study, which found no differences in Corsi Forwards Span (about 4) between 134 PD-N and 40 PD-MCI (Level I), either before DBS or at 1-year follow up (Merola et al., 2014). A related small study (n = 11) reported that Corsi backwards (not forwards) declined over 5 yrs in DBS patients (Contarino et al. (2007).  However, others have reported some evidence for PD-MCI and general forward vs backwards differences: Horta-Barba et al. (2020) reported that forward and backward spans (~5, forwards; 0.5SD less for backwards) in HC and PD-N were higher than those for PD-MCI (Level II; -1.5SD, two tests) (~4 forwards; 0.7SD less for backwards).  Women PD can be worse than men PD after 5 years’ duration, not before 5 years, but the comparison was cross-sectional (Chiara et al, 2013).  Sex differences on Corsi are often, but not always, found (Piccardi et al., 2008; Kessels et al., 2000; Kessels et al., 2008), including a 3D computerised 9-block version that is similar to the original Corsi:  <https://www.schuhfried.com/vienna-test-system/> - the Vienna Test System. |

| **Test properties**  (Please provide references for all statements or note if this is your judgment) |  |
| --- | --- |
| Which cognitive domain (or component/specific aspect of a cognitive domain) does this test *primarily* measure? | **Attention and working memory**  Arce and McMullen (2021)  Berch et al. (1998)  Kessels et al (2000; 2008)  Lezak et al. (2012; 5^th^ Ed.)  Wechsler WMS-III (1997).  Note: Some see the test, especially the CANTAB version, as measuring a “fronto-executive” function (Olde Dubbelink et al., 2014).  **Options:**  Attention and working memory  Executive function  Language  Memory  Visuospatial function |
| Which aspects of the domain you are assessing are not covered? | Not covered: Corsi Supra-span learning.  Not covered by Corsi Test: (a) Immediate span of attention (i.e. simultaneous, rather than sequential span); (b) Basic visuospatial skills. |

| **Use** |  |
| --- | --- |
| Was it *designed* to measure severity, screen or diagnosis of the domain? | Severity and diagnosis of lateralised spatial memory (Corsi, 1972; Milner, 1971). |
| Is there a cut-off score? Are there measures of screening/diagnostic performance of the test? (Receiver characteristic curve analyses; sensitivity/specificity; Positive Predictive Value/Negative Predictive Value), True Positive Rate, False Positive Rate etc.) | Cut-off score in relation to the diagnostic unit (MCI/dementia)  Cut-off scores are based on SD below norms or corresponding diagnostic categories based on percentiles (see above).  No diagnostic performance measures (that I have sighted); Biundo et al. (2014) ROC’s suggest that Corsi span is not useful for comparing PD-N vs PD-MCI, or PD-MCI vs PDD (I was not able to get the supplementary material to see the Corsi ROC values – perhaps Roberta has these?). However, PD-N and PD-MCI again no different, but PD-N / PD-MCI vs PDD differed for Corsi in Biundo et al. (2013); no details provided.  Without using any cut-off score, and basing performance on the median split within a group of PD, Olde Dubbelink et al. (2014) reported that Spatial Span on the CANTAB was a highly significant predictor of decline to PDD (30% of 63 patients) when followed over 7 years. They also found that CANTAB Pattern Recognition Memory (median split) was also predictive; Spatial Span combined with low beta EEG was the most predictive. |
| Is this appropriate for the PD population? If not, why (low sensitivity and/or specificity values)? | .  Yes, it has been used frequently for PD patients and is a commonly preferred test of visuospatial memory span (rather than immediate span of spatial attention).  However, the use of span scores in standard physical versions lacks sensitivity due to the generally limited range of scores even in HC and potential floor effects in impaired individuals. Total correct score is regarded as more sensitive (Kessels et al., 2000), but this seems seldom to have been used for PD. An experimental example of this use in PD is Kemps et al. (2005), but perhaps still a weak effect even then without dual-task interference.  It is possible that the CANTAB version (which I think uses a 3 sec display per item – more opportunity for distraction, longer recall times - and thus different to the general rule of 1 sec display) is more sensitive. This is based on the use of a median split spatial span score being predictive of decline to PDD (Olde Dubbelink et al., 2014). In that study, however, there was no assessment of whether any PD patients were already MCI as this began prior to the current MDS criteria. |
| **Clinimetric/psychometric properties in the target population** |  |
| **Feasibility /Acceptability** |  |
| Length | Number of items/domains  Average range in minutes  The number of items varies; the most common are 2 items (two trials per sequence) or 5 items (trials) at each span sequence, up to a span length of 9 blocks/boxes. The WMS-III is not preferred by some because it has 10 blocks rather than the original test of 9 blocks.  Generally about 5-10 minutes for both spans. Longer if more trials per span and if supra-span learning is added.  Different examples show wide degree of variation in procedures (summarised in Arce and McMullen, 2021). |
| Ambiguities in instructions to patient | Y/N, if Y, specify  No: Although there are differences in design and procedure, there should be little ambiguity in instructions to participants.  A good example of instructions would be in the WMS-III, but also in Kessels et al. (2000; 2008), which followed the same Wechsler format when looking at Corsi Backwards Span. Formal instructions are often not stated. |
| Ambiguities in rating anchors | Y/N, if Y, specify  No |
| Appropriateness of questions for population | Y/N, if Y, specify  Yes – appropriate to ask about visuospatial working memory in the PD population.  There are variable effects across different “visual” tasks in PD and across disease severity / general cognitive status. |
| Applicability across disease cognitive stages | MCI/Dementia  Spatial span is generally better suited for PDD than PD-MCI (e.g. Biundo et al., 2013, 2014).  More evidence on “Total Correct” and the product of span x total correct may suggest greater value for PD-MCI (Trojano et al., 1994; Kessels et al., 2000; Kemps et al., 2005).  There is limited evidence on the prediction of conversion to PDD using Corsi tasks. One exception is Olde Dubbelink et al. (2014), using the CANTAB Spatial Span, but this was based only on a median split rather than any statistically-derived cut-off. Need to check a little more whether others have followed that study in a similar way. So, this is an issue for the future. |
| Applicability across disease motor severity? | H&Y score: 1 (Unilateral involvement only)  H&Y score: 2 or 3(bilateral involvement with or without postural instability)  H&Y score: ≥4(several disabilities or confined to bed)  It has been used for Stages I, II and III. There is claim that computerised versions may be suitable for those confined to bed (Brunetti et al., 2014), but this seems unlikely to be of general use or needed. |
| Clear instructions to raters | Y/N, please specify  Yes – for most common versions. |
| Has the test been used by researcher other than developers?  By other groups  Not applicable? | Y/N, please specify  N/A |
| Are there instructions for dealing with missing data?  Not applicable? | NA  N/A |
| Floor and ceiling effects, skewed score distributions? | Please specify  For span, there is a limited range of scores, and floor effects with impairment, which limits Corsi span sensitivity. Total correct (or similar) may be better, especially when more than two trial sequences are used, but this measure is seldom reported. It may be that the CANTAB Sptial Span, with 3 sec exposure per block, is more sensitive (?). See earlier comments. |

| **Have the component of dimensionality, variability and dependency structures been analyzed?**  (Exploratory and confirmatory factor analysis (EFA and CFA)? | Y/N Please specify  Yes: Separation between - verbal and spatial working memory is well validated, but there is no clear evidence of any separation between forwards and backwards spatial span (Kessels et al., 2008).    Forward Corsi (Backwards not assessed, I think): Associated with “freedom from distractibility” in WISC-R; WAIS-R (see Kessels et al, 2008). Q whether this connects with Cantab Spatial Span version. |
| --- | --- |
|  |  |
| **Reliability: assessed – good; not good. Not assessed**  Are alternative versions developed?  If so, were they validated with reliability measures? | Generally acceptable / good (e.g. 079 for WMS-III; 0.76 for Vienna Test System).  However, these are seldom reported in other versions (when searching the word, “reliability” or “consistency”).  I do not have access to the information for CANTAB (some published studies using this with children).    Alternative versions include validated two-dimensional computerized versions (e.g. CANTAB; eCorsi; see above).  The Modified Walking Corsi test, which is focused on navigation / path finding / topographical memory, may produce deficits not found on the standard Corsi (Piccardi et al., 2013). |
| Internal consistency (A rule of thumb)  0.9 ≤ Cronbach’s alpha (α) Excellent  0.8 ≤ α < 0.9 Good  0.7 ≤ α < 0.8 Acceptable  0.6 ≤ α < 0.7 Questionable  0.5 ≤ α < 0.6 Poor  α < 0.5 Unacceptable  (Streiner, 2003 Journal of Personality Assessment, 80:1, 99-103, DOI: 10.1207/S15327752JPA8001_18) | Based on WMS-III reliability coefficient for internal consistency: 0.77. |
| “Corrected item-to-total correlation” | Y/N, if Y, specify how large  No |
| Test/retest | Y/N, if Y, specify how large  WMS-III: 0.79 |
| Inter-rater | Y/N, if Y, specify how large  N |
| Intra-rater | Y/N, if Y, specify how large  N |
| Other (e.g. Standard Error of Measurement) | Y/N, if Y, specify how large  WMS-III scaled scores: 1.43 |
| **Validity: assessed –good; not good. Not assessed (reference)** | Good (Kessels et al., 2008) |
| Face and Content validity (does test appears to be measuring what it is intended to measure and do test’s items comprehensively represent the domain of interest?) | Good; some debate as to whether the assumption of differences between forwards and backwards is valid. |
| Criterion validity (compared to gold-standard) or other criteria for  diagnosis) (e.g. other comparable tests or lesion or MRI research) | This test is taken as the gold-standard spatial working memory test. However, many similar-sounding tests around (Spatial Working Memory in CANTAB is a different test).  Doubts remain as to the influence of subtle (and not so subtle) variations in test conditions and procedures (Arce and McMullen, 2021). See earlier comments on CANTAB.  Brain networks: Zimmer (2008). |
| Construct validity (correlations with other convergent tests and divergent tests; known-groups comparisons) | Add data regarding:  Convergent validity  Divergent validity  Group-comparisons  Difficulty effect: Poorer performance with increasing length is well recognised and supports the use of the CBT (Fischer, 2001).  Divergent validity usually established by comparison to well-established differences with verbal working memory, although the separation of forwards and backwards is stronger for verbal span (Kessels et al., 2008).  The test has been used in numerous neurological patient groups (Arce and McMullen, 2021; Berch et al., 1998; Kessels et al., 2000, 2008; Lezak et al., 2012; Wechsler WMS-III, 1997).  Q: How much data is required for this section? |
| Reference group used to develop this test:   1. If available, please define the characteristic of the norm group adopted (demographic variables used for stratification: age, education, sex, etc). 2. What kind of standardized score generated (T score, Z score, derived IQ score, percentile ranks, etc)? 3. If translated/validated in other languages, are reference norms created? If so, please specify demographic variables used for stratification (age, education, gender etc). | Group characteristics:  Numerous standardized samples have been used – so, not sure how much detail or which would be best (Alex / Roberta: advice welcomed on this). Examples are Kessels et al., 2000, 2008; Orsini et al., 1987; Monaco et al., 2013, 2015; Wechsler WMS-III, 1997; CANTAB; Schuhfried, 1992). CANTAB does not have normative data. The largest normative samples are those for the Wechsler scales but limited data available for PD.  The average age in years (M, SD, range)  The average education in years (M, SD, range)  Sex (% women)  Country  Add the type of standardized score.  Add the language of translation and group characteristics:  The average age in years (M, SD, range)  The average education in years (M, SD, range)  Sex (% women)  Country  Generally, raw scores are common. Standard scores include Scaled Scores and percentile ranks (and categories based on those).  See 1). Generally validated within a country / language. Not sure if the WMS-III was validated in other countries. In general, the spatial nature of the task should buffer different language issues, but general cognitive ability may have some affect on performance (at least CANTAB; de Luca et al., 2003) and strategies used may be influenced by subvocal rehearsal. |

| **Responsiveness: assessed –good; not good. Not assessed (reference)** |  |
| --- | --- |
| Demonstrated to be sensitive to change (change over time or due to treatment)*  * [determined by the strength of the relationship between change in the test scores and the criterion or anchor scores] | If Yes, please specify  I have not seen strong evidence of sensitivity to change (but may need to check “training” / DBS studies again). |
| Has the minimal clinically important change and minimal clinically relevant incremental difference been assessed?  (besides a statistically significant improvement) | Minimal clinically important change assessed Y/N?  No. |
| Has this test been assessed or used in patients with other condition than that of interest? | The CBTT has been utilized to assess a wide range of neurological conditions. These include Huntington’s, Alzheimer’s, schizophrenia, Down syndrome, Williams syndrome, Korsakoff’s, acute brain injury, multiple sclerosis, dyscalculia (Berch et al., 1998; Kessels et al., 2000, 2008; van Asselen, 2006; CANTAB; Wechsler, 1997). |
| Other |  |
| **Overall impression** |  |
| Strengths | The primary strengths are its relative simplicity, wide use, and potential for remote (web-based) testing. |
| Weaknesses | A key issue is the limited variation of scores when “span” is the primary measure. It is likely that a combined forwards and backwards span score would be better. Moreover, it may be best if total score or the product score of items correct x span was used. However, this may require more sequences (trails) per span, per the methods commonly used in Italy.  It may also be that the duration of block tapping / exposure is an important variable to examine for PD, given the report by Olde Dubbelink et al. (2014), although I need to double-check the CANTAB Spatial Span procedure (will contact them).  Methodological inconsistencies across versions have been identified which are known or likely to affect scores (Arce and McMullen, 2021; Berch et al., 1998; Zimmer, 2008). Yes, both spatial configuration and path length affect the span.  A widely accepted version remains to be adopted and more work is needed to determine whether a Corsi test would usefully identify prediction of conversion to dementia in PD (from PD-N or PD-MCI), as only found the Olde Dubbelink et al. (2014) paper thus far.  Currently, there is little evidence that the Corsi test can reliably differentiate PD-N from PD-MCI. |

| **Level of Recommendation** |  |
| --- | --- |
| “Recommended” – The test was applied to the target population, AND was studied clinimetrically and found to be valid, reliable and sensitive to change, AND was used in studies by researchers, others than the developers  “Recommended with caveats” – Test’s properties were generally found to be adequate, but some of the measurement properties were not evaluated or not evaluated specifically in a PD cohort."  ‘Suggested’ – The test was applied to the target population, but only one of the other criteria applies  ‘Listed” – The test was applied to the target population, but none of the other criteria applies | Please choose one of these options  “Recommended with caveats”.  A direct comparison of at least two key methods across a large sample of PD patients, and followed longitudinally, is needed to clarify the value of the Corsi test. Ideally, these would be web-based open source options, once standardised procedural parameters and evidence base had been assessed. |
| Which type of study is this test suitable for and which type of study is it not suitable for (screening, prevalence, etiological (e.g. case-control or genetic), treatment trial, correlation with biological markers or other scales, e.g. of parkinsonism, clinical practice for diagnosis/ screening). List all. | Suitable for (see bold):  **Screening**  **Prevalence**  Differential reasoning (etiological)  **Case-control study**  **Treatment trial**  Biomarker study (**not at this point**)  Other  Unsuitable for (please add just categories for which the measure is critically unsuitable):  Screening  Prevalence  Differential reasoning (etiological)  Case-control study  Treatment trial  Biomarker study  Other |

**REFERENCES**

1. Arce T, McMullen K. The Corsi block-tapping test: Evaluating methodological practices with an eye towards modern digital frameworks. Computers in Human Behavior Reports. 2021 Jun 2:100099.
2. Aybek S, Gronchi‐Perrin A, Berney A, Chiuvé SC, Villemure JG, Burkhard PR, Vingerhoets FJ. Long‐term cognitive profile and incidence of dementia after STN‐DBS in Parkinson's disease. Movement Disorders. 2007 May 15;22(7):974-81.
3. Berch, D. B., Krikorian, R., & Huha, E. M. (1998). The Corsi block-tapping task:
4. Methodological and theoretical considerations. Brain and Cognition, 38(3), 317–338.
5. Biundo R, Weis L, Facchini S, Formento-Dojot P, Vallelunga A, Pilleri M, Antonini A. Cognitive profiling of Parkinson disease patients with mild cognitive impairment and dementia. Parkinsonism & related disorders. 2014 Apr 1;20(4):394-9.
6. Biundo R, Weis L, Pilleri M, Facchini S, Formento-Dojot P, Vallelunga A, Antonini A. Diagnostic and screening power of neuropsychological testing in detecting mild cognitive impairment in Parkinson’s disease. Journal of Neural Transmission. 2013 Apr 1;120(4):627-33.
7. Brunetti R, Del Gatto C, Delogu F. eCorsi: implementation and testing of the Corsi block-tapping task for digital tablets. Frontiers in psychology. 2014 Sep 2;5:939.
8. Busch RM, Farrell K, Lisdahl-Medina K, Krikorian R.J Clin Exp Neuropsychol. 2005 Jan;27(1):127-34.
9. Chiara P, Roberta Z, Elena S, Brigida M, Ilaria B, Claudio P. Cognitive function in Parkinson's disease: the influence of gender. Basal Ganglia. 2013 Jul 1;3(2):131-5.
10. Contarino MF, Daniele A, Sibilia AH, Romito LM, Bentivoglio AR, Gainotti G, Albanese A. Cognitive outcome 5 years after bilateral chronic stimulation of subthalamic nucleus in patients with Parkinson’s disease. Journal of Neurology, Neurosurgery & Psychiatry. 2007 Mar 1;78(3):248-52.
11. Corsi_PM. Human memory and the medial temporal region of the brain. Unpublished doctoral dissertation; McGill University, Montreal; 1972.
12. De Luca CR, Wood SJ, Anderson V, Buchanan JA, Proffitt TM, Mahony K, Pantelis C. Normative data from the CANTAB. I: development of executive function over the lifespan. Journal of clinical and experimental neuropsychology. 2003 Apr 1;25(2):242-54.
13. Fischer MH. Probing spatial working memory with the Corsi blocks task. Brain and cognition. 2001 Mar 1;45(2):143-54.
14. Horta-Barba A, Pagonabarraga J, Martínez-Horta S, Marín-Lahoz J, Sampedro F, Fernández-Bobadilla R, Botí M, Bejr-Kasem H, Aracil-Bolaños I, Pérez-Pérez J, Pascual-Sedano B. The free and cued selective reminding test in Parkinson's disease mild cognitive impairment: Discriminative accuracy and neural correlates. Frontiers in neurology. 2020 Apr 21;11:240.
15. Kemps E, Szmalec A, Vandierendonck A, Crevits L. Visuo-spatial processing in Parkinson's disease: evidence for diminished visuo-spatial sketch pad and central executive resources. Parkinsonism & related disorders. 2005 May 1;11(3):181-6.
16. Kessels RP, Van Zandvoort MJ, Postma A, Kappelle LJ, De Haan EH. The Corsi block-tapping task: standardization and normative data. Applied neuropsychology. 2000 Dec 1;7(4):252-8.
17. Kessels RP, van Den Berg E, Ruis C, Brands AM. The backward span of the Corsi Block-Tapping Task and its association with the WAIS-III Digit Span. Assessment. 2008 Dec;15(4):426-34.
18. Kudlicka A, Clare L, Hindle JV. Executive functions in Parkinson's disease: Systematic review and meta‐analysis. Movement disorders. 2011 Nov;26(13):2305-15.
19. Lezak MD, Howieson DB, Bigler ED. Neuropsychological assessment 5^th^ Edition. Oxford University Press, USA; 2012.
20. Merola A, Rizzi L, Artusi CA, Zibetti M, Rizzone MG, Romagnolo A, Bernardini A, Lanotte M, Lopiano L. Subthalamic deep brain stimulation: clinical and neuropsychological outcomes in mild cognitive impaired parkinsonian patients. Journal of Neurology. 2014 Sep;261(9):1745-51.
21. Milner B. Interhemispheric differences in the localization of psychological processes in man. *Br Med Bull.*1971;27:272–277.
22. Monaco M, Costa A, Caltagirone C, Carlesimo GA. Forward and backward span for verbal and visuo-spatial data: standardization and normative data from an Italian adult population. Neurological Sciences. 2013 May;34(5):749-54. See Erratum for tables: Neurol Sci (2015) 36:345–347).
23. Olde Dubbelink KT, Hillebrand A, Twisk JW, Deijen JB, Stoffers D, Schmand BA, Stam CJ, Berendse HW. Predicting dementia in Parkinson disease by combining neurophysiologic and cognitive markers. Neurology. 2014 Jan 21;82(3):263-70.
24. Orsini A, Grossi D, Capitani E, Laiacona M, Papagno C, Vallar G. Verbal and spatial immediate memory span: normative data from 1355 adults and 1112 children. The italian journal of neurological sciences. 1987 Dec;8(6):537-48.
25. Piccardi L, Bianchini F, Argento O, De Nigris A, Maialetti A, Palermo L, Guariglia C. The Walking Corsi Test (WalCT): standardization of the topographical memory test in an Italian population. Neurological Sciences. 2013 Jun;34(6):971-8.
26. Schuhfried G. *Wiener Testsystem, Vienna Reaction Unit, Basic Program. Development and Production of Scientific Equipment*. Molding, Austria; 1992.
27. Siegert RJ, Weatherall M, Taylor KD, Abernethy DA. A meta-analysis of performance on simple span and more complex working memory tasks in Parkinson's disease. Neuropsychology. 2008 Jul;22(4):450.
28. Spinnler H (1987) Italian standardization and classification of Neuropsychological tests. The Italian Group on the Neuropsychological Study of Aging. Ital J Neurol Sci Suppl 8:1–120.
29. Trojano L, Chiacchio L, De Luca G, Fragassi NA, Grossi D. Effect of testing procedure on Corsi's block-tapping task in normal subjects and Alzheimer-type dementia. Perceptual and motor skills. 1994 Jun;78(3):859-63.
30. van Asselen M, Kessels RP, Neggers SF, Kappelle LJ, Frijns CJ, Postma A. Brain areas involved in spatial working memory. Neuropsychologia. 2006 Jan 1;44(7):1185-94.
31. Wechsler, D. (1997). *Wechsler Memory Scale—Third Edition: Manual*. San Antonio, TX: Psychological Corporation.
32. Zimmer HD. Visual and spatial working memory: from boxes to networks. Neuroscience & Biobehavioral Reviews. 2008 Oct 1;32(8):1373-95.
33. Weingarten CP, Sundman MH, Hickey P, Chen NK. Neuroimaging of Parkinson's disease: Expanding views. Neuroscience & Biobehavioral Reviews. 2015 Dec 1;59:16-52.

**Rating test evaluation**

| **Test name: DIGIT ORDERING TEST** | Responses (possible) |
| --- | --- |
| **Version**  Are there several versions (alternate or parallel) of the test? If so, which one has been assessed? | If yes, please add complete titles.  Digit Ordering Test  Adaptive Digit Ordering Test (DOT-A/DOT-B) |
| **Description of test**   - What population the test was originally developed for? - Which domains/components of domains do the test assess? - Are there multiple domains or a predominant domain assessed? - Does it score impaired/unimpaired? - Does it score severity   (subtle/mild/moderate/severe)? | This test of verbal working memory was developed to assist in differentiating PD from controls. It was described in Cooper et al.^1^ study as a significant differentiator between early, untreated PD patients and controls. Cooper et al^2^ found that dopaminergic but not anticholinergic therapies led to improvement in performance on the DOT, and in untreated PD, it demonstrated the most significant deficit compared to other measures.  It is considered a dual task paradigm, a “verbal working memory test that requires maintenance and mental manipulation (ordering) of digit series.”^3^  It does not measure general cognitive slowing associated with PD, but specific cognitive slowing that selectively affects executive cognitive processes.^3^  Hoppe et al^3^ present norms, converting raw scores to T scores  Werheid et al^4^ present the Adaptive Digit Ordering Test (DOT-A), and an alternate version (DOT-B) based on the DOT. These versions include items of balanced structure and increasing difficulty which are administered at a standard presentation rate, and are analogous to Digit Span from the WMS-R. These authors replicate earlier studies^1,2,3^ and extend the findings to patients with frontal lobe lesions in addition to PD, and also establishes validity and reliability using a representative sample of healthy subjects. |
| **Scaling Metric**: Type of response format? (e.g., Likert, Binary, multiple-choice, continuum, reaction time, etc.) | Specify the scale of measurement:  Verbal response; 15 distinct trials, continuous variable  DOT-A/B: 6 distinct items, two trials each item |
| **Respondent: Patient:** any specific requirements for the target study population (e.g. non-demented, motor, auditory, visual abilities, etc)? | Patient interviewed by a clinician/examiner  Requires adequate hearing |
| **Access:** How can the test be obtained (mail address or website)? | Presented in the appendix of Hoppe et al^3^.  DOT-A/DOT-B presented in the appendix of Werheid et al^4^. |
| Copyrighted or in public domain?  Conditions for use? Fee? | Public domain; free |
| **Translations:** Has the test been translated and validated in other languages besides English? | Y/N, add language mutations with at least one validity study in the target population  German^3,4^  Italian – Pasotti et al^5^ included validation and norms  Chinese – Ma et al^6^. employed DOT but did not validate |

| **Test properties**  (Please provide references for all statements or note if this is your judgment) |  |
| --- | --- |
| Which cognitive domain (or component/specific aspect of a cognitive domain) does this test *primarily* measure? | Attention and working memory |
| Which aspects of the domain you are assessing are not covered? | Basic attention span, divided attention, sustained attention |

| **Use** |  |
| --- | --- |
| Was it *designed* to measure severity, screen or diagnosis of the domain? | Developed to measure verbal working memory with an executive component |
| Is there a cut-off score? Are there measures of screening/diagnostic performance of the test? (Receiver characteristic curve analyses; sensitivity/specificity; Positive Predictive Value/Negative Predictive Value), True Positive Rate, False Positive Rate etc.) | Cut-off score in relation to the diagnostic unit (MCI/dementia)  No cut-off score  T scores provided, mean 50 SD 10  %ile provided for DOT-A/DOT-B |
| Is this appropriate for the PD population? If not, why (low sensitivity and/or specificity values)? | Yes; this measure was developed to assist in differentiating between PD and normal population |
| **Clinimetric/psychometric properties in the target population** |  |
| **Feasibility /Acceptability** |  |
| Length | Number of items/domains  Average range in minutes  15 items (DOT)  6 items (DOT-A) |
| Ambiguities in instructions to patient | Y/N, if Y, specify  No (Werheid et al^4^ commented on instructions, examining whether patients responded incorrectly to the first trial of the first item, or made errors from incorrect numerical ordering, and concluded that DOT-A deficits do not arise from difficulty following instructions.) |
| Ambiguities in rating anchors | Y/N, if Y, specify  No |
| Appropriateness of questions for population | Y/N, if Y, specify  Y – appropriate for PD, PD-MCI, PDD |
| Applicability across disease cognitive stages | MCI/Dementia  Yes |
| Applicability across disease motor severity? | H&Y score: 1 (Unilateral involvement only)  H&Y score: 2 or 3(bilateral involvement with or without postural instability)  H&Y score: ≥4(several disabilities or confined to bed)  Applicable across all stages, provided the patient is able to communicate through speech  de Frias et al^7^ classified participants using H&Y scores of 1-4, into two groups – mild PD vs. severe PD, compared to health older adults. Using the DOT-A (among other measures), they found that the severe PD group had poorer performance on the DOT than the mild PD group and health older adults, with no other group comparisons using other measures being significant. A slower rate of neurocognitive speed was associated with poorer executive functioning (including DOT-A). |
| Clear instructions to raters | Yes  Hoppe et al^3^ described three measures: Cooper (from the original Cooper^1^, using position-related scoring; Pairs, where every pair of correctly recalled subsequent digits is scored, and Correct, with the number of completely correct answers.  Werheid et al^4^ describe the DOT-A, with similar instructions and different test stimuli. This is the version that seems to be cited in most subsequent research |
| Has the test been used by researcher other than developers?  By other groups | Y  Hoppe et al^3^  Pasotti et al^5^  Werheid et al^4^  de Frias et al^7^  Kavcic & Scheid^8^  Ma et al^6,9^  Muller et al^10^  Schwid et al^11^  Senturk et al^12^ |
| Are there instructions for dealing with missing data?  Not applicable? | Y/N, please specify  No instructions; each item needs to be completed to obtain total score. Hoppe et al^3^ mention that for standardization, normative data were linearly interpreted for missing values. |
| Floor and ceiling effects, skewed score distributions? | Please specify  Hoppe et al^3^ test is normally distributed. |

| **Have the component of dimensionality, variability and dependency structures been analyzed?**  (Exploratory and confirmatory factor analysis (EFA and CFA)? | Y/N Please specify  No |
| --- | --- |
|  |  |
| **Reliability: assessed – good; not good. Not assessed**  Are alternative versions developed?  If so, were they validated with reliability measures? | Y/N Please specify  DOT-A is an adapted version, and it has an alternate version (DOT-B) validated with reliability measures^4^, but not cited in subsequent research |
| Internal consistency (A rule of thumb)  0.9 ≤ Cronbach’s alpha (α) Excellent  0.8 ≤ α < 0.9 Good  0.7 ≤ α < 0.8 Acceptable  0.6 ≤ α < 0.7 Questionable  0.5 ≤ α < 0.6 Poor  α < 0.5 Unacceptable  (Streiner, 2003 Journal of Personality Assessment, 80:1, 99-103, DOI: 10.1207/S15327752JPA8001_18) | Hoppe et al^3^: Good in the clinical control group (patients from a day care unit at a dept of cognitive neurology) >.80. In the Control Group, it was questionable across each type of scoring (.61-.66)  Depending on the scoring, for the PD group it was poor (Correct: .59), or acceptable (Cooper: .71; Pairs .70).  Werheid et al^4^ DOT-A: used crossover item assignment (first test half was first trial of item 1, second trial of item 2, and so on). Spearman-Brown coefficnets were .75 (DOT-A) and .79 (DOT-B)  Parallel test reliability was r_par =_ .68 |
| “Corrected item-to-total correlation” | Y/N, if Y, specify how large  Y (Hoppe et al^3^). Not significant in PD group, but significantly correlated with total performance in healthy control and neurology samples. |
| Test/retest | Y/N, if Y, specify how large  Not evaluated in PD studies; note that  Schwid et al^11^ demonstrated poor test-retest reliability in the MS population |
| Inter-rater | Y/N, if Y, specify how large  Not evaluated (note that score is the # of correct responses) |
| Intra-rater | Y/N, if Y, specify how large  Not evaluated (note that score is the # of correct responses) |
| Other (e.g. Standard Error of Measurement) | Y/N, if Y, specify how large |
| **Validity: assessed –good; not good. Not assessed (reference)** |  |
| Face and Content validity (does test appears to be measuring what it is intended to measure and do test’s items comprehensively represent the domain of interest?) | Good |
| Criterion validity (compared to gold-standard) or other criteria for  diagnosis) (e.g. other comparable tests or lesion or MRI research) | Hoppe et al^3^ – two scores from the Modified Card Sorting Test: number of categories resolved, number of perseverative errors – external indices of executive functioning. Both scores were significantly correlated with the DOT scores.  Werheid et al^4^ DOT-A/B: three parameters of the two-back task. DOT-A/B scores were negatively correlated with omission errors and false positive responses. Speed of answering was not correlated. |
| Construct validity (correlations with other convergent tests and divergent tests; known-groups comparisons) | Add data regarding:  Convergent validity  Divergent validity  Group-comparisons  Werheid et al^4^ found that differences between patients and health controls were found in DOT-A but only in the digit span measures. Also, using pairwise *t* testing, the difference between DOT-A and DS forward does not reach significance, but the difference between DOT-A and DS backward does (p = .004). The authors suggest that directly comparing DS and DOT-A can help to discriminate between working memory (DOT-A) and short-term memory capacity (DS) in clinical practice. |
| Reference group used to develop this test:   1. If available, please define the characteristic of the norm group adopted (demographic variables used for stratification: age, education, sex, etc) 2. What kind of standardized score generated (T score, Z score, derived IQ score, percentile ranks, etc)? 3. If translated/validated in other languages, are reference norms created? If so, please specify demographic variables used for stratification (age, education, gender etc). | Group characteristics:  Hoppe et al (2002)  DOT  The average age in years (M, SD, range)  PD: 57.3 (12.4) (range not provided)  Healthy controls: 47.2 (16.9)  DOT-A^4^  PD: 58.2 (7.8; range not provided)  Controls: 58.8 (7.3)  DOT scores are significantly correlated with chronological age.  DOT-A (and DOT-B) normed with healthy subjects^4^  Age groups across decades  The average education in years (M, SD, range)  DOT-A^4^ provide total years of education across decades. Total sample 13.7 (2.2). Range not included, but range of mean years of education across decade groups is 13.0-15.4.  Sex (% women). (Werheid et al^4^) normative sample: 50% women  Country Werheid et al^4^: Germany  Add the type of standardized score.  DOT-A/B^4^ – sample sizes of the age-stratified subgroups not large enough and mean scores did not differ significantly, so the groups were collapsed and percentile scores were calculated for the entire sample.  Add the language of translation and group characteristics:  The average age in years (M, SD, range)  The average education in years (M, SD, range)  Sex (% women)  Country  T scores are generated^3^  Validated in German^3^  Italian^5^ (note that different versions were used – DOT-WM 0-8 longest sequence and DOT-T 0-12 recalled sequences) |

| **Responsiveness: assessed –good; not good. Not assessed (reference)** |  |
| --- | --- |
| Demonstrated to be sensitive to change (change over time or due to treatment)*  * [determined by the strength of the relationship between change in the test scores and the criterion or anchor scores] | If Yes, please specify  Not assessed (e.g., Werheid et al^4^) |
| Has the minimal clinically important change and minimal clinically relevant incremental difference been assessed?  (besides a statistically significant improvement) | Minimal clinically important change assessed Y/N?  N |
| Has this test been assessed or used in patients with other condition than that of interest? | Y/N, please specify if one or several condition  MS^11^ – poor test-retest reliability in this population  MS^8^; DOT-A  Vascular cognitive impairment^6^; DOT-A  Schizophrenia^10^: DOT-A  Alzheimer’s disease^12^ and anosognisia |
| Other |  |
| **Overall impression** |  |
| Strengths | The DOT and DOT-A appear to discriminate between PD and healthy controls, as well mild PD vs. severe PD. The measure is easy to administer, similar to Digit Span. Moreover, it is more sensitive than Digit Span backwards to impairment in verbal executive functions in Parkinson’s patients^3^. It was developed specifically to understand verbal working memory in PD, and the DOT-A was developed in analogy to Digit Span^4^.  Note that most studies after 2002 use the Werheid^4^ DOT-A, but typically refer to the measure as the DOT. |
| Weaknesses | There are questions about test-retest reliability^11^ in the MS population, and this has not been evaluated in the PD population, despite the measure having been developed with the PD population.  Sample sizes in psychometric studies have been relatively small.  There are a number of studies which include the DOT, with significant findings, but references for the measure are not included so it is not clear which version was used (e.g., Senturk et al^12^.)  The extent to which the measure can assess changes over time is not known. |

| **Level of Recommendation** |  |
| --- | --- |
| “Recommended” – The test was applied to the target population, AND was studied clinimetrically and found to be valid, reliable and sensitive to change, AND was used in studies by researchers, others than the developers  “Recommended with caveats” – Test’s properties were generally found to be adequate, but some of the measurement properties were not evaluated or not evaluated specifically in a PD cohort."  ‘Suggested’ – The test was applied to the target population, but only one of the other criteria applies  ‘Listed” – The test was applied to the target population, but none of the other criteria applies | Please choose one of these options  Suggested   - Internal consistency and split half reliability are questionable in patients with PD - The component of dimensionality, variability and dependency structure has not been analyzed. - The extent to which the measure can assess changes over time is not known. |
| Which type of study is this test suitable for and which type of study is it not suitable for (screening, prevalence, etiological (e.g. case-control or genetic), treatment trial, correlation with biological markers or other scales, e.g. of parkinsonism, clinical practice for diagnosis/ screening). List all. | Suitable for:  Screening  Prevalence  Differential reasoning (etiological)  Case-control study  Treatment trial  Biomarker study |

REFERENCES

1. Cooper, JA, Sagar, HJ, Jordan, N, Harvey, NS, Sullivan, EV. Cognitive impairment in early, untreated Parkinson’s disease and its relationship to motor disability. Brain. 1991; 114, 2095-2122.
2. Cooper, JA, Sagar, HJ, Doherty, SM, Jordan, N, Tidswell, P, Sullivan, EV. Different effects of dopaminergic and anticholinergic therapies on cognitve and motor function in Parkinson’s disease. Brain. 1992; 115, 1701-1725.
3. Hoppe, C, Muller, U, Werheid, K, Thone, A, von Cramon, DY. Digit ordering test: Clinical, psychometric, and experimental evaluation of a verbal working memory test. The Clinical Neuropsychologist. 2000; 14(1), 38-55.
4. Werheid, K, Hoppe, C, Thone, A, Muller, U, Mungersdorf, M, von Cramon, DY. The Adaptive Digit Ordering Test: Clinical application, reliability, and validity of a verbal working memory test. Archives of Clinical Neuropsychology. 2002. 17, 547-565.
5. Pasotti, F, De Luca, G, Aiello, EN, Gramegna, C, Di Gangi, M, Foderaro, et al. A multi-component, adaptive Working Memory Assessment Battery (WoMAB): validation and norms in an Italian population sample. Neurological Sciences. 2021. Retrieved from <https://doi.org/10.1007/s10072-021-0541607>
6. Ma, J, Zhang, Y, Guo, Q. Comparison of vascluar cognitive impairment - no dementia by multiple classification methods. International Journal of Neuroscience. 2015. 125(11), 823-830.
7. de Frias, CM, Dixon, R, Fisher, N, Camicioli, R. Intraindividual variability in neurocognitive speed: A comparison of Parkinson’s disease and normal older adults. Neuropsychologia. 2007; 45, 2499-2507.
8. Kavcic, V, Scheid, E. Attentional blink in patients with multiple sclerosis. Neuropsychologia. 2011; 49, 454-460.
9. Ma, J, Ma, S, Zou, H, Zhang, Y, Chan, P, Ye, Z. Impaired serial ordering in nondemented patients with mild Parkinson’s disease. PLoS ONE. 2018; 13(5), e0197489.
10. Muller, U, Werheid, K, Hammerstein, E, Jungmann, S, Becker, T. Prefrontal cognitive decifics in patients with schizophrenia treated with atypical or conventional antipsychotics. European Psychiatry. 2005. 20, 70-73.
11. Schwid, SR, Tyler, CM, Scheid, EA, Weinstein, A, Goodman, AD, McDermott, MP. Cognitive fatigue during a test requiring sustained attention: a pilot study. Multiple Sclerosis. 2003. 9, 503-508.
12. Senturk, G, Bilgic, B, Arslan, AB, Bayram, A, Hanagasi, H, Gurvit, H, et al. Cognitive and anatomical correlates of anosognosia in amnestic mild cognitive impairment and early-stage Alzheimer’s disease. International Psychogeriatrics. 2017. 29(2), 293-302.

**Rating test evaluation**

| **Test name:** | VISUAL SEARCH TEST (VST)  Also named: Digit Cancellation test (DCT); Attentive Matrices test |
| --- | --- |
| **Version**  Are there several versions (alternate or parallel) of the test? If so, which one has been assessed? | The original version was by Spinnler & Tognoni in 1987^1^.  Della Sala and colleagues ^2^ proposed a new version (nDCT) with different scoring system. |
| **Description of test**   - What population the test was originally developed for? - Which domains/components of domains do the test assess? - Are there multiple domains or a predominant domain assessed? - Does it score impaired/unimpaired? - Does it score severity   (subtle/mild/moderate/severe)? | In VST, the examinee is required to visualize three-digit matrices, each consisting of 13 lines and 10 numbers (from 0 to 9) randomly located.  The target stimuli are 10 in the first matrix, 20 in the second and 30 in the third one.  He/she is asked to bar the number/s equal to those printed on the top of the matrices (i.e., 5 for the first one; 2 and 6 for the second one; 1, 4, and 9 for the third one). The maximum time to complete each task is 45 s, and the number of corrected barred items is calculated as a raw score.   - VST was originally developed to assess AD^2^ - This test mostly evaluate attention, namely visual attention, visual processing speed and working memory. - Does not provide impairment - Does not score severity |
| **Scaling Metric**: Type of response format? (e.g., Likert, Binary, multiple-choice, continuum, reaction time, etc.) | Specify the scale of measurement:  Sum of the number of digits correctly barred within the time given, for all matrices. Range 0-60. |
| **Respondent: Patient:** any specific requirements for the target study population (e.g. non-demented, motor, auditory, visual abilities, etc)? | Good visual abilities (it may be difficult to complete the task for people with PSP if they cannot scrawl the eyes toward the bottom of the matrix); no limb paralysis or severe apraxia. |
| **Access:** How can the test be obtained (mail address or website)? | Instruction and matrices can be found in the original manual^1^ |
| Copyrighted or in public domain?  Conditions for use? Fee? | free |
| **Translations:** Has the test been translated and validated in other languages besides English? | The original test was developed in Italy^1,2^ and it has been standardized in Italian population.  Each task has digits only, therefore it is theoretically suitable for any language where Arabic number system is in use. |

| **Test properties**  (Please provide references for all statements or note if this is your judgment) |  |
| --- | --- |
| Which cognitive domain (or component/specific aspect of a cognitive domain) does this test *primarily* measure? | Attention (sustained attention)  Working memory and visual search are also involved |
| Which aspects of the domain you are assessing are not covered? | Divided and alternating attention |

| **Use** |  |
| --- | --- |
| Was it *designed* to measure severity, screen or diagnosis of the domain? | It was developed to assess visual attention. Normative data allow to detect pathological performances |
| Is there a cut-off score? Are there measures of screening/diagnostic performance of the test? (Receiver characteristic curve analyses; sensitivity/specificity; Positive Predictive Value/Negative Predictive Value), True Positive Rate, False Positive Rate etc.) | Cut-off score in relation to the diagnostic unit (MCI/dementia)  There is a cut off score which allows to discriminate between patients with and without dementia (AD)^2^..  In a sample of PD patients, DCT was able to discriminate PDD from PD-MCI and PD-NC (p<0.001)^3^. |
| Is this appropriate for the PD population? If not, why (low sensitivity and/or specificity values)? | Y |
| **Clinimetric/psychometric properties in the target population** |  |
| **Feasibility /Acceptability** |  |
| Length | The target digits are 10 in the first matrix, 20 in the second and 30 in the third. The time to complete the task is 45 seconds for each matrix.  The whole test takes about 3-4 minutes. |
| Ambiguities in instructions to patient | N |
| Ambiguities in rating anchors | N |
| Appropriateness of questions for population | N |
| Applicability across disease cognitive stages | MCI/Dementia  Y |
| Applicability across disease motor severity? | H&Y 1-3 |
| Clear instructions to raters | Y |
| Has the test been used by researcher other than developers?  By other groups  Not applicable? | Y by other groups, mostly by Italian researches |
| Are there instructions for dealing with missing data?  Not applicable? | Not applicable |
| Floor and ceiling effects, skewed score distributions? | Not specified |

| **Have the component of dimensionality, variability and dependency structures been analyzed?**  (Exploratory and confirmatory factor analysis (EFA and CFA)? | N |
| --- | --- |
|  |  |
| **Reliability: assessed – good; not good. Not assessed**  Are alternative versions developed?  If so, were they validated with reliability measures? | NO alternative version of the tasks; alternative scoring method only. |
| Internal consistency (A rule of thumb)  0.9 ≤ Cronbach’s alpha (α) Excellent  0.8 ≤ α < 0.9 Good  0.7 ≤ α < 0.8 Acceptable  0.6 ≤ α < 0.7 Questionable  0.5 ≤ α < 0.6 Poor  α < 0.5 Unacceptable  (Streiner, 2003 Journal of Personality Assessment, 80:1, 99-103, DOI: 10.1207/S15327752JPA8001_18) | N |
| “Corrected item-to-total correlation” | N |
| Test/retest | Tested in the original task^1^but authors report it to be over 0.50 without adding information  Della Sala^2^found a poor test-retest reliability of the nDCT (0.53) |
| Inter-rater | N |
| Intra-rater | N |
| Other (e.g. Standard Error of Measurement) | N |
| **Validity: assessed –good; not good. Not assessed (reference)** |  |
| Face and Content validity (does test appears to be measuring what it is intended to measure and do test’s items comprehensively represent the domain of interest?) | No information |
| Criterion validity (compared to gold-standard) or other criteria for  diagnosis) (e.g. other comparable tests or lesion or MRI research) | No information |
| Construct validity (correlations with other convergent tests and divergent tests; known-groups comparisons) | Della Sala and colleagues ^2^ found correlation between nDCT and Raven's Progressive Matrices, set A, B, C and D, Weigl's Sorting Test, a two-dimensional constructional apraxia, test an episodic memory test (a 10 disyllabic word learning test), the Token Test; Completion Test |
| Reference group used to develop this test:   1. If available, please define the characteristic of the norm group adopted (demographic variables used for stratification: age, education, sex, etc). 2. What kind of standardized score generated (T score, Z score, derived IQ score, percentile ranks, etc)? 3. If translated/validated in other languages, are reference norms created? If so, please specify demographic variables used for stratification (age, education, gender etc). | Group characteristics:  321 Italian healthy subjects  The age in years ranged from 40 yrs old to >85 about 40-50 subjects in each age class apart from >85 (n=19)  The education in years range was from <3 to >13  134 males  Italy  Adjustment values based on age and education were calculated and divided into equivalent scores (0-4) |

| **Responsiveness: assessed –good; not good. Not assessed (reference)** |  |
| --- | --- |
| Demonstrated to be sensitive to change (change over time or due to treatment)*  * [determined by the strength of the relationship between change in the test scores and the criterion or anchor scores] | N |
| Has the minimal clinically important change and minimal clinically relevant incremental difference been assessed?  (besides a statistically significant improvement) | N |
| Has this test been assessed or used in patients with other condition than that of interest? | Alzheimer^2,4-6^; people with PKU^7^; stroke^8^; epilepsy^9,10^, PD, PSP, MSA^11,12^ and (Huntington's Disease^13^ |
| Other |  |
| **Overall impression** |  |
| Strengths | Easy to administer, quick; sensitive to dementia also in PD |
| Weaknesses | Not widely used; few studies in PD. Not really studied from a psychometric point of view. |

| **Level of Recommendation** |  |
| --- | --- |
| “Recommended” – The test was applied to the target population, AND was studied clinimetrically and found to be valid, reliable and sensitive to change, AND was used in studies by researchers, others than the developers  “Recommended with caveats” – Test’s properties were generally found to be adequate, but some of the measurement properties were not evaluated or not evaluated specifically in a PD cohort."  ‘Suggested’ – The test was applied to the target population, but only one of the other criteria applies  ‘Listed” – The test was applied to the target population, but none of the other criteria applies | Suggested |
| Which type of study is this test suitable for and which type of study is it not suitable for (screening, prevalence, etiological (e.g. case-control or genetic), treatment trial, correlation with biological markers or other scales, e.g. of parkinsonism, clinical practice for diagnosis/ screening). List all. | Suitable for:  Screening  Unsuitable for (please add just categories for which the measure is critically unsuitable):  Treatment trial  Biomarker study |

**REFERENCES**

1. Spinnler H, Tognoni G. Standardizzazione e taratura italiana di test neurpsicologici. Ital J Neurol Sci (suppl. 8). 1987.
2. Della Sala S, Laiacona M, Spinnler H, Ubezio C. A cancellation test: its reliability in assessing attentional deficits in Alzheimer's disease. Psychol Med. 1992;22(4):885-901. doi:10.1017/s0033291700038460. PMID: 1488486.
3. Biundo R, Weis L, Pilleri M, et al. Diagnostic and screening power of neuropsychological testing in detecting mild cognitive impairment in Parkinson's disease. J Neural Transm (Vienna). 2013;120(4):627-33. doi:10.1007/s00702-013-1004-2. PMID: 23483334.
4. Solfrizzi V, Panza F, Torres F, et al. Selective attention skills in differentiating between Alzheimer's disease and normal aging. J Geriatr Psychiatry Neurol. 2002;15(2):99-109. doi:10.1177/089198870201500209. PMID: 12083601.
5. Parasuraman R, Haxby JV. Attention and brain function in Alzheimer's disease: A review. Neuropsychology. 1993;7(3):242-72. doi:10.1037/0894-4105.7.3.242.
6. Perry RJ, Hodges JR. Attention and executive deficits in Alzheimer's disease: A critical review. Brain. 1999;122(3):383-404. doi:10.1093/brain/122.3.383.
7. Huijbregts SCJ, de Sonneville LMJ, Licht R, van Spronsen FJ, Sergeant JA. Sustained attention and planning in phenylketonuria. Dev Neuropsychol. 2002;21(3):243-69. doi:10.1207/S15326942DN2103_3.
8. Buxbaum LJ, Ferraro MK, Veramonti T, et al. Hemispatial neglect: Subtypes, neuroanatomy, and disability. Neurology. 2004;62(5):749-56. doi:10.1212/01.WNL.0000113730.73031.F4.
9. Helmstaedter C, Witt JA. Cognitive outcome of antiepileptic drug treatment in epilepsy: The role of executive functions. Epilepsy Behav. 2012;24(4):448-54. doi:10.1016/j.yebeh.2012.05.003.
10. Aldenkamp AP, Bodde N. Behavioural and cognitive effects of seizures: The role of epilepsy surgery. Epilepsy Res. 2005;68(2):167-74. doi:10.1016/j.eplepsyres.2005.09.018.
11. Soliveri P, Monza D, Paridi D, et al. Neuropsychological follow up in patients with Parkinson's disease, striatonigral degeneration-type multisystem atrophy, and progressive supranuclear palsy. J Neurol Neurosurg Psychiatry. 2000;69(3):313-8. doi:10.1136/jnnp.69.3.313. PMID: 10945805; PMCID: PMC1737110.
12. Siri C, Duerr S, Canesi M, et al., A cross-sectional multicenter study of cognitive and behavioural features in multiple system atrophy patients of the parkinsonian and cerebellar type. J Neural Transm (Vienna). 2013;120(4):613-8. doi:10.1007/s00702-013-0997-x. PMID: 23462799.
13. Zappacosta B, Monza D, Meoni C, et al. Psychiatric symptoms do not correlate with cognitive decline, motor symptoms, or CAG repeat length in Huntington's disease. Arch Neurol. 1996;53(6):493-7. doi:10.1001/archneur.1996.00550060035012. PMID: 8660149.

**Rating test evaluation**

| **Test name:** The Brief Test of Attention (BTA) | Responses (possible) |
| --- | --- |
| **Version**  Are there several versions (alternate or parallel) of the test? If so, which one has been assessed? | If yes, please add complete titles.    The Brief Test of Attention (BTA)  There is only one version with two parallel forms, both forms have to be administered to obtain the total BTA scores.^1^ |
| Description of test    · What population the test was originally developed for?  · Which domains/components of domains do the test assess?  · Are there multiple domains or a predominant domain assessed?    · Does it score impaired/unimpaired?  · Does it score severity  (subtle/mild/moderate/severe)? | · It was not specifically developed for a target population.  · BTA measures auditory divided attention reducing confounding task demands such as psychomotor speed or conceptual reasoning. It is sensitive to subtle attentional impairments.  · The test author emphasises that the  BTA was designed to identify deficits in attention rather than to differentiate between levels of normal attention.^2^  · Both the perceptual (i.e., distinguishing letters from numbers) and conceptual (counting from 1 to 12) requirements of the BTA are simple.  · It scores impairment vs unimpairment.  · It does not score severity per se.  But the scores can be classified as*^2,3^:  >74h Above average  25yh-74^th^ Average  10^th^-24^th^ Low average  2^nd^-9^th^ Borderline impaired  <2^nd^ Impaired. |
| **Scaling Metric**: Type of response format? (e.g., Likert, Binary, multiple-choice, continuum, reaction time, etc.) | Specify the scale of measurement:  The subject reports the response verbally (number of letters or numbers were recited in each item list). The number of correct monitored lists (10 in total) is summed across forms. Total BTA scores range from 0 to 20. |
| **Respondent: Patient:** any specific requirements for the target study population (e.g. non-demented, motor, auditory, visual abilities, etc)? | Auditory capabilities should be preserved. Literacy is needed.  It can be used with individuals  with visual and/or motor impairments.^1^ |
| **Access:** How can the test be obtained (mail address or website)? | The original paper^1^ refers that The Brief Test of Attention may be obtained by contacting the first author (David Schretlen, Ph.D.). However, nowadays the BTA^TM^ test is commercialized by PAR:<https://www.parinc.com/Products/Pkey/29> |
| Copyrighted or in public domain?  Conditions for use? Fee? | PARinc: BTA^TM^ introductory Kit including manual, Stimulus Audio CD and 50 Scoring forms costs $142.00. |
| **Translations:** Has the test been translated and validated in other languages besides English? | Y/N, add language mutations with at least one validity study in the target population  Normative data was generated for the Latin American Spanish-speaking adult population^4^, for European Portuguese adult population^5^, and for Ecuadorian adult population^6^. |

| **Test properties** (Please provide references for all statements or note if this is your judgment) |  |
| --- | --- |
| Which cognitive domain (or component/specific aspect of a cognitive domain) does this test *primarily* measure? | **Attention and working memory**  Executive function  Language  Memory  Visuospatial function  In my opinion, this is a screening task to measure attention and working memory, however, executive function and language are also involved.  This test has also been studied as embedded performance validity indicators to detect non-credible responding.^7,8^ |
| Which aspects of the domain you are assessing are not covered? | The author emphasises the test only measures on auditory selective and divided attention, and not visual attention or other aspects of attentional functioning in the auditory modality. |

| **Use** |  |
| --- | --- |
| Was it *designed* to measure severity, screen or diagnosis of the domain? | The test is designed to measure presence of deficit but not normal functioning or severity. This is just a screening task, and the addition of other tests is required for comprehensive evaluation of attention. |
| Is there a cut-off score? Are there measures of screening/diagnostic performance of the test? (Receiver characteristic curve analyses; sensitivity/specificity; Positive Predictive Value/Negative Predictive Value), True Positive Rate, False Positive Rate etc.) | Cut-off score in relation to the diagnostic unit (MCI/dementia)  Impairment classification based on BTA percentiles are presented in the manual and summarised previously, see above *.  No specific Cut-off score for MCI or dementia have been found.  BTA has been recognized as a possible embedded performance validity test to detect suboptimal cognitive effort (non-credible responding) during the neuropsychological assessment in clinical samples. Previous study^7^reported AUC=0.83 and a cut-off=15, whereas another study^8^ found an AUC=between 0.79 and 0.87, as well as a cut-off=9. Finally, in the mild Traumatic brain injury population a AUC=0.78 was found^9^.  Adapted BTA form has been used in a clinical trial including PDD patients, but it has been used as secondary efficacy measure.^10^ |
| Is this appropriate for the PD population? If not, why (low sensitivity and/or specificity values)? | Limited information regarding these questions is available in the literature.  David et al^11^ reported BTA is sensitive to changes after a 24-month physical exercise intervention in non-demented PD.  Neely KA et al^12^ reported that BTA scores and number of pulses in a visually guided grip force task discriminated PSP from PD, MSAp, and controls with high degrees of sensitivity and specificity (AUC>0.99). However, the results demonstrated that the combination of these variables were not useful in distinguishing MSAp from PD or health subjects (highest AUC=0.747). This study included a limited sample of patients (12 PD, 12 MSAp, 8 PSP, and 12 healthy controls).  Dubois et al^10^ performed an efficacy and safety study of the acetylcholinesterase inhibitor donepezil hydrochloride in PDD. PDD patients (n =550) were randomised to donepezil (5 or 10 mg) or placebo for 24 weeks. BTA assessments showed highly significant benefits in patients treated with donepezil 10 or 5 mg compared with placebo. In this study BTA was adapted, however, details about BTA adaptation were not provided. |
| **Clinimetric/psychometric properties in the target populatio****n** |  |
| **Feasibility /Acceptability** |  |
| Length | Number of items/domains  Average range in minutes  Form N and L consist of 10 lists of letters and numbers (e.g., “M-6-3-R-2”) that increase in length from 4 to 18 items.  Eight minutes to administer the complete test. Each form requires 4 minutes to administer and score. |
| Ambiguities in instructions to patient | Y/N, if Y, specify No. Directions are read to the subject by the examiner. Moreover, there are two sample items before the entire form administration. |
| Ambiguities in rating anchors | Y/N, if Y, specify  No. Correct responses receive a score of 1. Each form scores range from 0 to 10. The total score ranges from 1 to 20 and it is converted to a percentile using an Appendix A in the manual. Normative scores derived by age group. Because age ranges for the BTA overlap, users are instructed to select the age range with the midpoint corresponding to the examinee’s age. The highest possible percentile on the test is >74. |
| Appropriateness of questions for population | Y/N, if Y, specify  Y, this test is appropriate for the literate population. |
| Applicability across disease cognitive stages | MCI/Dementia  As mentioned before, an adapted BTA form has been used in a clinical trial including PDD patients^10^, but the specific modifications from the original form were not reported.  The original BTA version has been used in other studies including PDD.^13^ |
| Applicability across disease motor severity? | H&Y score: 1 (Unilateral involvement only)  H&Y score: 2 or 3(bilateral involvement with or without postural instability)  H&Y score: 4(several disabilities or confined to bed)  Yes, this test is designed to reduce the confounding effect of motor impairment. |
| Clear instructions to raters | Y/N, please specify  Yes |
| Has the test been used by researcher other than developers?  By other groups  Not applicable? | Y/N, please specify  Yes, by other groups |
| Are there instructions for dealing with missing data?  Not applicable? | Y/N, please specify  No |
| Floor and ceiling effects, skewed score distributions? | Please specify  Unknown |

| **Have the component of dimensionality, variability and dependency structures been analyzed?**  (Exploratory and confirmatory factor analysis (EFA and CFA)? | Y/N Please specify  In the original paper a principal components analysis based on a subsample of 107 psychiatric patients who completed a battery of neuropsychological tests was performed. Three factors accounting for 71.9% total variance with eigenvalues greater than unity (7.26, 1.08, and 1.01, respectively) were identified. Factor 1 is aptly described as reflecting general and verbal mental abilities. Factor 2 clearly reflects attentional abilities, as the BTA, WAIS-R Digit Span, and Digit Symbol composite scores, and all three Stroop scores loaded most highly on it. Factor 3 appears to be defined by perceptual abilities and psychomotor speed.^1^ | | |
| --- | --- | --- | --- |
| **Reliability: assessed – good; not good. Not assessed**              Are alternative versions developed?  If so, were they validated with reliability measures? | | Y/N Please specify  Yes, internal consistency was assessed as “good” but test-retest reliability as “Low to adequate”.^2^  Acceptable: based on the 349 normal adults and children, the Pearson correlation (r) between forms N and L was 0.69. However, the between-forms correlation increased to 0.81 for the combined (n=926) normal and clinical samples.^1^  Paired-sample t-tests revealed that normal adults produced virtually identical scores on Forms L and N (8.5, SD = 1.6 vs. 8.5, SD = 1.5; t(274)= 0.67; p = 0.50). The patients produced lower and more variable scores overall, but they also showed virtually identical performance on Forms L and N (5.6, SD = 2.9 vs. 5.5, SD = 2.9; t(596)= 1.02; p = 0.31), as did the normal children (t(73) = -0.62; P =0.54).^1^  Following Strauss^3^, Schrelten et al^2^ with a N=1231 reported reliability r=0.90 for the Total BTA and 0.82 and 0.81 respectively, for Forms L and N.  Separate internal reliability estimates for adults and children are not available.  There are no order or practice effects between these forms in normal samples. The mean difference score of the 765 patients and normal subjects for whom order was recorded did approach zero (M = .15, SD = 1.8 points). The distribution of difference scores was mildly leptokurtic (0.30), indicating that more subjects than expected produced difference scores of zero. The distribution showed negligible skewness (-0.07), suggesting that exposure to the first form given neither facilitated nor impeded performance on the second form. In fact, over 97% of normal subjects and 93% of patients produced scores that differed by <;3 points between Forms L and N.^1^ | |
| Internal consistency (A rule of thumb)  0.9 ≤ Cronbach’s alpha (α) Excellent  0.8 ≤ α < 0.9 Good  0.7 ≤ α < 0.8 Acceptable  0.6 ≤ α < 0.7 Questionable  0.5 ≤ α < 0.6 Poor  α < 0.5 Unacceptable  (Streiner, 2003 Journal of Personality Assessment, 80:1, 99-103, DOI: 10.1207/S15327752JPA8001_18) | | Good.^1,2^  ﻿Based on the 349 normal adults and children, internal consistency analyses yielded a coefficient alpha of .82 for the BTA (.71 for Form L and .66 for Form N). When these data, were pooled with those of 480 patients for whom item scores were recorded, and internal consistency analyses were repeated using this combined sample (n = 829), the coefficient alpha increased to .91 for the BTA (.83 for Form Land .84 for Form N).^1^ | |
| “Corrected item-to-total correlation” | | Y/N, if Y, specify how large  Not assessed. | |
| Test/retest | | Y/N, if Y, specify how large  Test-retest reliability estimates were not available on 1996 publication.  Strauss^2^ reported Test -restest stability as 0.70 based on Scheretlen et al^2^ using a sample of old hypertense adults (9 months test-retest interval). No access to this data. | |
| Inter-rater | | Y/N, if Y, specify how large  Not assessed. | |
| Intra-rater | | Y/N, if Y, specify how large  Not assessed. | |
| Other (e.g. Standard Error of Measurement) | | Y/N, if Y, specify how large  N | |
| **Validity: assessed –good; not good. Not assessed (reference)** | |  | |
| Face and Content validity (does test appears to be measuring what it is intended to measure and do test’s items comprehensively represent the domain of interest?) | | Good.^2^ | |
| Criterion validity (compared to gold-standard) or other criteria for  diagnosis) (e.g. other comparable tests or lesion or MRI research) | | Schretlen et al^14^ showed that patients with impaired attention (Huntington disease patients) performed poorly on BTA, whereas impaired patients whose deficits do not include attention (amnesic) performed like normal controls. The sample of this article is limited.  Following Strauss^3^, Schrelten et al^2^ reported that BTA scores were also related to individual differences in cerebral volume as measured by MRI. Further, the effects of age on BTA performance may have been mediated by age-related differences in cerebral volume.  Valos^15^ established validity of the BTA amongst children who had sustained moderate and severe traumatic brain injury. However, I have no full-access to this document to report more data. | |
| Construct validity (correlations with other convergent tests and divergent tests; known-groups comparisons) | | In a subsample of 452 patients and 149 normal subjects (age range 6-86 mean=38.1 SD=19.7; education mean 11.0, SD = 4.0). BTA scores correlated more highly with backward (r = .53, p < .001) than forward (r = .43, p < .001) digit span. Disparity between these two correlations was statistically significant U(59)=3.12, p < .01.^1^  In a subsample of 311 patients and 73 normal adults (age range 15 to 86 Mean=41.1, SD = 15.4). Total BTA scores correlated more highly with Part B (r =-.55, p < .001) than Part A (r= -.48, p = .001) of the Trail Making Test. Again, the difference between these correlations was statistically significant (t(381)= 2.42, P <.02).^1^  In a subsample of 183 patients and 44 normal control subjects (age range from 15 to 72 mean =37.9,SD= 11.7; education average of 12.1, SD = 3.3 years). BTA scores correlated significantly with all three parts of the Stroop (word reading, r = .66; color naming, r = .68; color-word naming, r = 67; all p < .001). However, BTA scores did not correlate more strongly with the interference trial than with word reading or colour naming trials.^1^  Principal Components analysis based on a subsample of 107 psychiatric patients who completed a battery of neuropsychological Tests (age range from 19 to 68 years, mean=10.7 SD=2.6 years of school) was reported. Factor 2 clearly reflects attentional abilities, as the BTA (0.61), WAIS-R Digit Span, and Digit Symbol (WAIS-R Attention Digit span, Digits Symbol, 0.60) composite scores, and all three Stroop scores (Words 0.81, Colour 0.84 and words-colour 0.61) loaded most highly on it.^1^ | |
| Reference group used to develop this test:  1) If available, please define the characteristic of the norm group adopted (demographic variables used for stratification: age, education, sex, etc).    2) What kind of standardized score generated (T score, Z score, derived IQ score, percentile ranks, etc)?    3) If translated/validated in other languages, are reference norms created? If so, please specify demographic variables used for stratification (age, education, gender etc). | | Group characteristics:  Normal Samples  Adults:  N=275  Age=47.1 (19.3)  Sex= M112/F163  White=205  Black=30  Other=0  Education 14.4 (2.7)      Children:  N=74  Age= 10.1 (2.5)  Sex= M38/F36  White=70  Black=4  Other=0  Education=4.0(2.5)      Clinical Sample:  N= 577  Age= 40.4 (15.7)  Sex= M327/F250  White=336  Black=201  Other=7  Education=12.2 (3.2)    Country= USA  Sandardised score= Percentile    - Race was not recorded for 40 normal control subjects and 33 patients. Education not recorded for 37 patients. 45 adult normal control subjects did not undergo any screening procedures, the remaining (n = 213) adult subjects were screened for dementia, severe psychiatric disorders, and current substance abuse.^1^    Language of translation and group characteristics:    Latin American Spanish-speaking  adult population^4^:  N=3970 (See supplementary table 1).    European Portuguese adult population^5^ (see supplementary table 2).    Ecuadorian adults from Quito between the ages of 18 and 85, Education: Mean=13.2; SD = 4.6. Total sample: 322 (174 Woman/148 Man).^16^ | |
|  | |  |  |

| **Responsiveness: assessed –good; not good. Not assessed (reference)** |  |
| --- | --- |
| Demonstrated to be sensitive to change (change over time or due to treatment)*    * [determined by the strength of the relationship between change in the test scores and the criterion or anchor scores] | If Yes, please specify    See the three reported studies in the previous sections:    David et al^11^ reported that BTA is sensitive to changes after a 24-month physical exercise intervention in non-demented PD (limited p value=0.048). While other tests were sensitive to 12-months follow-up changes (Digit span, Stroop), BTA was only significantly different after a longer period.    Five-hundred fifty PDD patients were randomised to donepezil (5 or 10 mg) or placebo for 24 weeks. BTA assessments showed highly significant benefits in patients treated with donepezil 10 (treatment difference (confidence interval): 1.00 (0.42–1.57) p= < 0.001)) or 5 mg (treatment difference (confidence interval): 0.78 (0.22–1.34) p=0.007) compared with placebo.^10^  Nine consecutive PD patients undergoing unilateral deep brain-stimulating electrode implantation in the globus pallidus interna were evaluated approximately 1 month before and 3 months after surgery. Preoperative (mean 15.8 (2.7)) and postoperative (15.2 (4.2)) BTA were similar. One patient improved 1SD whereas two patients worsen (one 1SD and the other 2SD).^17^ |
| Has the minimal clinically important change and minimal clinically relevant incremental difference been assessed?  (besides a statistically significant improvement) | Minimal clinically important change assessed Y/N?  N |
| Has this test been assessed or used in patients with other condition than that of interest? | Y/N, please specify if one or several condition  Yes. Traumatic brain injury^18,19^, sleep apnea^20^, cancer^21,22^, as well as bipolar disorder and schizophrenia^23^. |
| Other |  |
| **Overall impression** |  |
| Strengths | This test is a bedside, short screening test.  It is suitable for individuals with visual and/or motor impairments. |
| Weaknesses | Limited to auditory divided attention  Only screen for deficits. No normal function evaluation.  More research is needed on test stability over time.  Translations and validations in other languages besides English are limited to Spanish and Portuguese.  Cut-off score in relation to the diagnostic unit (MCI/dementia) was not found. |

| **Level of Recommendation** |  |
| --- | --- |
| “Recommended” – The test was applied to the target population, AND was studied clinimetrically and found to be valid, reliable and sensitive to change, AND was used in studies by researchers, others than the developers    “Recommended with caveats” – Test’s properties were generally found to be adequate, but some of the measurement properties were not evaluated or not evaluated specifically in a PD cohort."    ‘Suggested’ – The test was applied to the target population, but only one of the other criteria applies    ‘Listed” – The test was applied to the target population, but none of the other criteria applies | Please choose one of these options  ‘Suggested’ – The test was applied to the target population, but only one of the other criteria applies |
| Which type of study is this test suitable for and which type of study is it not suitable for (screening, prevalence, etiological (e.g. case-control or genetic), treatment trial, correlation with biological markers or other scales, e.g. of parkinsonism, clinical practice for diagnosis/ screening). List all. | Suitable for:  **Screening**  **Prevalence**  Differential reasoning (etiological)  **Case-control study**  **Treatment trial**  Biomarker study  Other    Unsuitable for (please add just categories for which the measure is critically unsuitable):  Screening  Prevalence  Differential reasoning (etiological)  Case-control study  Treatment trial  Biomarker study  Other |

· Supplementary table 1, Sample distribution by age, education, and gender from Guàrdia-Olmos et al^24^.

|  | n Total | Age | Education | | Gender | |
| --- | --- | --- | --- | --- | --- | --- |
|  |  | Mean (SD) | 1 to 12 | >12 | Male | Female |
|  |  |  | n (%) | n (%) | n (%) | n (%) |
| Argentina | 320 | 45.7 (19.5) | 148 (46.3%) | 172 (53.8%) | 96 (30.0%) | 224 (70.0%) |
| Bolivia | 274 | 55.8 (22.0) | 226 (82.5%) | 48 (17.5%) | 99 (36.1%) | 175 (63.9%) |
| Chile | 320 | 55.1 (19.6) | 241 (75.3%) | 79 (24.7%) | 134 (41.9%) | 186 (58.1%) |
| Cuba | 306 | 53.0 (19.7) | 234 (76.5%) | 72 (23.5%) | 142 (46.4%) | 164 (53.6%) |
| El Salvador | 257 | 56.0 (20.7) | 203 (79.0%) | 54 (21.0%) | 100 (38.9%) | 157 (61.1%) |
| Guatemala | 214 | 53.2 (17.4) | 133 (62.1%) | 81 (37.9%) | 95 (44.4%) | 119 (55.6%) |
| Honduras | 184 | 48.6 (18.8) | 140 (76.1%) | 44 (23.9%) | 67 (36.4%) | 117 (63.6%) |
| Mexico | 1300 | 52.5 (20.5) | 1005 (77.3%) | 295 (22.7%) | 431 (33.2%) | 869 (66.8%) |
| Paraguay | 263 | 53.0 (14.8) | 216 (82.1%) | 47 (17.9%) | 101 (38.4%) | 162 (61.6%) |
| Peru | 245 | 43.4 (20.6) | 87 (35.5%) | 158 (64.5%) | 87 (35.5%) | 158 (64.5%) |
| Puerto Rico | 294 | 50.9 (18.5) | 160 (54.4%) | 134 (45.6%) | 126 (42.9%) | 168 (57.1%) |

· Supplementary table 2, Demographic characteristics of the sample from Vicente et al^5^.

| Age group | ni | Age (M) | Age (SD) | Edu (M) | Edu (SD) | Woman: N | Man: N |
| --- | --- | --- | --- | --- | --- | --- | --- |
| 20 ± 2 year | 19 | 20.7 | 1.5 | 12.7 | 2.1 | 13 | 6 |
| 25 ± 2 year | 56 | 24.5 | 1.3 | 15.3 | 2.1 | 37 | 19 |
| 30 ± 2 year | 16 | 30.4 | 1.6 | 15.3 | 3.8 | 11 | 5 |
| 35 ± 2 year | 11 | 34.8 | 1.7 | 13.6 | 4.7 | 4 | 7 |
| 40 ± 2 year | 18 | 40.4 | 1.4 | 12.1 | 4.4 | 7 | 11 |
| 45 ± 2 year | 13 | 44.7 | 1.5 | 11.4 | 5.5 | 6 | 7 |
| 50 ± 2 year | 28 | 50.2 | 1.2 | 9.6 | 4.8 | 15 | 13 |
| 55 ± 2 year | 11 | 54.9 | 1.8 | 11.2 | 4.8 | 9 | 2 |
| 60 ± 2 year | 21 | 59.8 | 1.5 | 9.4 | 4.3 | 15 | 6 |
| 65 ± 2 year | 28 | 65.4 | 1.3 | 7.2 | 4.0 | 20 | 8 |
| 70 ± 2 year | 22 | 69.4 | 1.5 | 7.8 | 4.4 | 16 | 6 |
| 75 ± 2 year | 26 | 75.0 | 1.6 | 5.7 | 3.6 | 14 | 12 |
| 80 ± 2 year | 12 | 79.3 | 1.6 | 4.3 | 1.5 | 8 | 4 |
| >82 | 19 | 87.0 | 3.0 | 4.6 | 2.2 | 13 | 6 |
| **Total** | **300** | **50.4** | **21.2** | **10.4** | **5.2** | **188** | **112** |

**REFERENCES**

1. Schretlen D, Bobholz JH, Brandt J. Development and psychometric properties of the brief test of attention. Clinical Neuropsychologist. 1996;10(1):80–9.
2. Schretlen D, Jayaram G, Maki P, Robinson H, & Devilliers C. Functional correlates of neurocognitive deficits in adults with severe mental disorders. Journal of the International Neuropsychological Society. 1997; 3: 25.
3. Strauss E. A Compendium of neuropsychological tests: administration, norms, and commentary. 3rd ed. Spreen Otfried, Sherman EMS, editors. Oxford University Press; 2006.
4. Rivera D, Perrin PB, Aliaga A, et al. Brief Test of Attention: Normative data for the Latin American Spanish speaking adult population. NeuroRehabilitation. 2015;37(4):663–76.
5. Vicente SG, Rivera D, Barbosa F, et al. Normative data for tests of attention and executive functions in a sample of European Portuguese adult population. Aging, Neuropsychology, and Cognition. 2020;1–20.
6. Rodríguez-Lorenzana A, Ramos-Usuga D, Díaz LA, et al. Normative data of neuropsychological tests of attention and executive functions in Ecuadorian adult population. Aging, Neuropsychology, and Cognition. 2020;1–20.
7. Busse M, Whiteside D. Detecting suboptimal cognitive effort: Classification accuracy of the conner’s continuous performance test-II, brief test of attention, and trail making test. Clinical Neuropsychologist. 2012; 26(4):675–87.
8. Sharland MJ, Waring SC, Johnson BP, et al. Further examination of embedded performance validity indicators for the Conners’ Continuous Performance Test and Brief Test of Attention in a large outpatient clinical sample. Clinical Neuropsychologist. 2018; 32(1):98–108.
9. Whiteside DM, Gaasedelen OJ, Hahn-Ketter AE, et al. Derivation of a cross-domain embedded performance validity measure in traumatic brain injury. Clinical Neuropsychologist. 2015;29(6):788–803.
10. Dubois B, Tolosa E, Katzenschlager R, et al. Donepezil in Parkinson’s disease dementia: A randomized, double-blind efficacy and safety study. Movement Disorders. 2012; 27(10):1230–8.
11. David FJ, Robichaud JA, Leurgans SE, et al. Exercise improves cognition in Parkinson’s disease: The PRET-PD randomized, clinical trial. Movement Disorders. 2015; 30(12):1657–63.
12. Neely KA, Planetta PJ, Prodoehl J, et al. Force Control Deficits in Individuals with Parkinson’s Disease, Multiple Systems Atrophy, and Progressive Supranuclear Palsy. PLoS ONE. 2013; 8(3).
13. Leroi I, Brandt J, Reich SG, et al. Randomized placebo-controlled trial of donepezil in cognitive impairment in Parkinson’s disease. Int J Geriatr Psychiatry. 2004;19:1–8.
14. Schretlen D, Brandt J, Bobholz JH. Validation of the brief test of attention in patients with Huntington’s disease and amnesia. Clinical Neuropsychologist. 1996;10(1):90–5.
15. Valos A.M. Validity and sensitivity of the Brief Test of Attention for children who have sustained moderate and severe traumatic brain injury. Dissertation Abstracts International: Section B: The Sciences and Engineering. 2006;66(7-B):3962.
16. Rodríguez-Lorenzana A, Ramos-Usuga D, Díaz LA, et al. Normative data of neuropsychological tests of attention and executive functions in Ecuadorian adult population. Aging, Neuropsychology, and Cognition. 2020.
17. Troster AI, Fields JA, Wilkinson, et al. Unilateral pallidal stimulation for Parkinson’s disease: Neurobehavioral functioning before and 3 months after electrode implantation. Neurology. 1997; 49(4):1078-83.
18. Rao V, Bertrand M, Rosenberg P, et al. Predictors of New-Onset Depression After Mild Traumatic Brain Injury. Journal of Neuropsychiatry. 2010;22(1):100–4.
19. Wong TM. Validity and sensitivity of the brief test of attention with acute brain injury and mild head injury patients. Archives of Clinical Neuropsychology.1999 14(8):728–9.
20. Aloia M, Ilniczky N, Pasquelina DD, et al. Neuropsychological changes and treatment compliance in older adults with sleep apnea. Journal of psychosomatic research. 2003; 54(1):71–6.
21. Butler RW, Copeland DR, Fairclough DL, et al. A Multicenter, Randomized Clinical Trial of a Cognitive Remediation Program for Childhood Survivors of a Pediatric Malignancy. Journal of Consulting and Clinical Psychology. 2008;76(3):367–78.
22. Correa DD, DeAngelis LM, Shi W, Thaler H, Glass A, Abrey LE. Cognitive functions in survivors of primary central nervous system lymphoma. Neurology. 2004; 62(4):548-55.
23. Schretlen DJ, Cascella NG, Meyer SM, et al. Neuropsychological Functioning in Bipolar Disorder and Schizophrenia. Biol Psychiatry. 2007; 62(2): 179–186.
24. Guardia-Olmos J, Però-Cebollero M, Rivera D, & Arango-Lasprilla JC. Methodology for the development of normative data for ten Spanish-language neuropsychological tests in eleven Latin American countries. NeuroRehabilitation. 2015; 37: 493-499.

**Rating test evaluation**

| **Test name:** Test of everyday attention (TEA). Map mission and Visual Elevator subtests. | Responses (possible) | | |
| --- | --- | --- | --- |
| **Version**  Are there several versions (alternate or parallel) of the test? If so, which one has been assessed? | If yes, please add complete titles.  Three parallel versions were included in the original TEA Manual. Separate norms are presented for version A and B, but limited information is available for C version.^1,2^  The mean of the practice effects from B to C are provided in the manual, therefore, the change in subject’s score can be interpreted against the practice effect.  TEA-Ch and TEA Ch2 are also available for children and adolescents (TEA-Ch: 6-15 years; TEA-Ch2: 5-15 years)). | | |
| **Description of test**  · What population the test was originally developed for?  · Which domains/components of domains do the test assess?  · Are there multiple domains or a predominant domain assessed?  · Does it score impaired/unimpaired?  · Does it score severity  (subtle/mild/moderate/severe)? | The TEA was principally designed to offer a clinically valid assessment of individuals from 18 to 80 years of age, who have experienced some form of acquired neurological insult.^1^  TEA includes 8 subtests which assess sustained attention (i.e. Elevator Counting and Lottery), selective attention (i.e. Map Search and Telephone Search), switching attention (i.e. Visual Elevator), working memory (i.e. Elevator Counting with Distraction and Auditory Elevator with Reversal) and divided attention (i.e. Telephone Search while Counting).^2^  TEA was designed to be ecologically valid, i.e. many of the subtests are designed to mimic everyday activities.  Map Search subtest was designed as a measure of visual selective attention. The examinee has to search for symbols (e.g. a knife and fork representing eating facilities) on a tourist map of a city.  The visual elevator subtest was designed to measure visual attention switching hence, cognitive flexibility. Subjects have to count up and down as they follow a series of visually presented “floors” in the elevator.  The TEA consists of eight subtests (standardised in a similar way to the WAIS-III and WMS-III to have an-age adjusted mean of 10 with a standard deviation of 3). Therefore, the impairment, as well as severity scores can be determined easily. Scaled scores and approximate conversion to percentiles are provided for each subtest, as well as the impaired/unimpaired cut-off by age group.^1,2^ | | |
| **Scaling Metric**: Type of response format? (e.g., Likert, Binary, multiple-choice, continuum, reaction time, etc.) | Map Search subtest: number of items found in 1 minutes or 2 minutes.  The visual elevator subtest:  Accuracy score: how many final floor numbers the subject gets correct out of 10. Timing score, or time per switch. Total time taken for the correct items divided for the total number of switches for the correct items in seconds.^1^ | | |
| Respondent: Patient: any specific requirements for the target study population (e.g. non-demented, motor, auditory, visual abilities, etc)? | Visual acuity must be preserved. Some subjects may have difficulties in detecting the symbols in the Map Search or in the visual elevator subtests.  In some samples (i.e stroke), the ability to comprehend the task could limit the administration of these tests.  Visual Elevator test would not be feasible for subgroups of older adults (>80 years old) with serious cognitive impairments (missing data in 19% of participants due to failure to comprehend test instructions despite repetition and practice)^3^. Moreover, vision problems played a role in not completing tests, mainly on the Map Search Test.  Visual Elevator number correct score has a correlation exceeding 0.3 (0.39) with NART-measured verbal intelligence. In this context, Robertson and colleagues suggested that participants with intelligence below the average and a Visual Elevator score just below the average score should not be seen as an impaired performance on the attentional domain.^1^  However, because of the battery nature of the TEA, it would appear reasonable to suggest that the best accommodation would be to use those sub-tests which fit the individual’s needs for assessment, and to combine those sub-tests with other tests which may allow for more appropriate assessment of an individual’s abilities.^1^ | | |
| **Access:** How can the test be obtained (mail address or website)? | The TEA is commercialized by Pearson:  <https://www.pearsonclinical.co.uk/Psychology/AdultCognitionNeuropsychologyandLanguage/AdultAttentionExecutiveFunction/TestofEverydayAttention(TEA)/TestofEverydayAttention(TEA).aspx>  ISBN: 9780749171803 | | |
| Copyrighted or in public domain?  Conditions for use? Fee? | Complete Kit (Includes manual, pack of 25 scoring sheets, cue book, stimulus cards, maps and USB in a bag) costs £522.29 | | |
| **Translations:** Has the test been translated and validated in other languages besides English? | Y/N, add language mutations with at least one validity study in the target population  Reliability and validity of Cantonese version of TEA among Hong Kong Chinese was studied.^4^  In a previous study, authors obtained the permission of the publisher to translate the English version to Mandarin.^5^ They examine the measurement properties of TEA in patients with chronic stroke including test-retest reliability between parallel forms, practice effect and critical values for detecting true change corrected for practice effect and measurement error. No specific information about the Mandarin validation was provided.^5^ | | |
| **Test properties** (Please provide references for all statements or note if this is your judgment) | |  |
| Which cognitive domain (or component/specific aspect of a cognitive domain) does this test *primarily* measure? | | The tests were designed to evaluate visual attention, specifically selective attention and attentional switching.^2^ |
| Which aspects of the domain you are assessing are not covered? | | Sustained attention and divided attention are not covered by these subtests.^2^ |

| **Use** |  |
| --- | --- |
| Was it *designed* to measure severity, screen or diagnosis of the domain? | The interpretation of the subtest scores provides information to measure severity as well the impairment presence/absence. |
| Is there a cut-off score? Are there measures of screening/diagnostic performance of the test? (Receiver characteristic curve analyses; sensitivity/specificity; Positive Predictive Value/Negative Predictive Value), True Positive Rate, False Positive Rate etc.) | Cut-off score in relation to the diagnostic unit (MCI/dementia)    No specific cut-off score was found for MCI or dementia. However, scaled scores for four age bands are provided by the authors, as well as specific scaled score cut-off to determine subtest scores in normal or abnormal range. |
| Is this appropriate for the PD population? If not, why (low sensitivity and/or specificity values)? | Limited information regarding these questions is available in the literature.    It has been suggested a regression model in which Trail making part B, TEA Map Search first minute, and CVLT-II SF total immediate recall showed a high AUC (0.90) for detecting conversion to PDD in four years.^6^ The same authors stated that no other group has used the Map Search task to predict PDD.  Performance in the TEA deteriorated significantly in the PDD vs. PD, correlating with blood pressure response.^7^ |
| **Clinimetric/psychometric properties in the target population** |  |
| **Feasibility /Acceptability** |  |
| Length | Number of items/domains  Average range in minutes    There are eight sub-tests. Completition time for the whole battery: 45 to 60 minutes, based on Pearson website:  https://www.pearsonassessments.com/store/usassessments/en/Store/Professional-Assessments/Cognition-%26-Neuro/The-Test-of-Everyday-Attention/p/100000182.html?tab=overview  Map Search: involves searching a map for a total of two minutes and circling a particular symbol on the map when located. There are eighty symbols to be found.^2^ Visual elevator subtest: The test is self-paced by the subject. Each trial is timed from the moment the first number is spoken by the subject until the last number is spoken. Ten items are presented excluding the practice items.^1^ |
| Ambiguities in instructions to patient | Y/N, if Y, specify  No |
| Ambiguities in rating anchors | Y/N, if Y, specify  No |
| Appropriateness of questions for population | Y/N, if Y, specify  Yes, it is based on everyday materials. |
| Applicability across disease cognitive stages | MCI/Dementia  Yes, it has been administered both in PD-MCI and PDD.^6,7^ |
| Applicability across disease motor severity? | H&Y score: 1 (Unilateral involvement only)    H&Y score: 2 or 3(bilateral involvement with or without postural instability)    H&Y score: 4(several disabilities or confined to bed)    The map search could be difficult for patients in advanced stages    It has been administered in PD patients with H&Y 1-3.^6,7^ |
| Clear instructions to raters | Y/N, please specify  Yes |
| Has the test been used by researcher other than developers?  By other groups  Not applicable? | Y/N, please specify Y    TEA subtests have been used by different groups to evaluate:    Attentional deficits in stuttering population. Map search 1 evidenced significant differences (F=4.483 o p=0.03 Cohen’s d=0.638)^8^    Effectiveness of a visual attention retraining program in stroke patients.^9^ No significant results were reported.    TEA was used to measure generalisation of gain in TBI patients with limited results.^10^    Previous study investigated the measurement properties of Test of Everyday Attention (TEA) in patients with chronic stroke including: test–retest reliability between parallel forms (i.e. forms AB, BC and CA), practice effect and critical values for detecting true change corrected for practice effect and measurement error.^5^      Previous studies investigated the Attentional deficits in patients with closed head injuries, discriminant validity as well as the latent structure of the test.^11,12^ Reliability and validity of the Cantonese Version of TEA has also been investigated.^4^    Richmond et al.^13^ trained working memory in Older Adults and tested transfer to other measures including TEA. Visual elevator accuracy showed ceiling effect.    Stapleton and Connelly^14^ explored the clinical pre-driving assessment practices and recommendations of a group of Irish occupational therapists for people with stroke. Consensus emerged for the use of TEA, among others, to give an indication of the patient’s underlying cognitive status.    Attentional deficits have been found in small young-adult learning disabilities group (n=8) in comparison to controls (n=8) using Map Search (F(1,14) = 41.8; P < .01 ) and visual elevator accuracy (F(1,12) = 11.9; P < .01 ) and timing (F(1,12) = 11.8; P < .01).^15^    A population of adults aged 80 years and older (n=249) was studied and it has been showed that this population-based sample of very old adults was able to complete most of the TEA subscales. However, the Visual Elevator Test, which measures the domain of attentional switching, was the most challenging test for these older participants.^16^    In a previous study, a virtual reality cognitive training application was used to detect MCI in persons using the application at home without the help of an examiner. A very limited sample was used (MCI=6 and Healthy control=6). Average performance (mean duration) correlated significantly with cognitive tests including TEA visual elevator raw score r=-0.617 p=0.040), but not Map Search.^17^ |
| Are there instructions for dealing with missing data?  Not applicable? | Y/N, please specify. NA |
| Floor and ceiling effects, skewed score distributions? | Please specify    Map Search shows ceiling effect in young age groups (18-34 and 35-49).  Visual Elevator raw accuracy shows ceiling effect in all age groups, whereas this effect is less evident in timing scores.^1^ |

| **Have the component of dimensionality, variability and dependency structures been analyzed?**  (Exploratory and confirmatory factor analysis (EFA and CFA)? | | Y/N Please specify    Factor analysis of attention tests for standardisation sample (N=154) explained 64% of the variance. In the Test Manual, four factors were reported. The first factor, labelled visual selective attention/speed, included  the Map Search and the Telephone search. The second factor, labelled attentional switching, included the Visual Elevator task. The third factor, sustained attention, was formed by the Lottery, Elevator Counting and the Dual Task Telephone Search. The last factor involved Auditory-Verbal Working Memory, and involved the Elevator Counting with Distraction task.  However, the same authors suggested a three-factor model provides a better account of attentional performance. Map Search loaded on Visual Selective attention/Speed factor (0.84) factor. Visual Elevator (number correct) loaded on Attentional switching (0.78) factor. No information was reported for Visual elevator timing.^1,2^  Chan et al.^12^ performed a factor analysis among a group of 92 patients with traumatic brain injury. Comparisons were made of the fit of the previously identified models based on exploratory factor analysis, comprising three-to-four factors. The results indicated that the 3-factor model with a visual selection component, a sustained attention component and a switching component provided an appropriate account of attentional performance than 4-factor models also in the clinical population. However, in that study visual elevator visual elevator raw score load in switching attention (0.68) whereas visual elevator time score load on sustained attention (0.85). | |
| --- | --- | --- | --- |
|  |  | | |
| **Reliability: assessed – good; not good. Not assessed**  Are alternative versions developed?  If so, were they validated with reliability measures? | Y/N Please specify    Yes. Good for the original version.^1,2^    Three different versions exist.    Reliability coefficients for one-week test-retest on version A and B for 118 subjects from normal sample^2^:  Map Search 1 min: =0.83  Map Search 2 min: =0.86  Visual Elevator accuracy=0.71  Visual Elevator timing score=0.79  Only 39 controls were assessed also with Version C further weeks after B, the reliability coefficient was^2^:  Map Search 1 min: =0.87  Map Search 2 min: =0.80  Visual Elevator accuracy=0.76  Visual Elevator timing score=0.70    Reliability coefficients for one-week test-retest on version A and B for 74 stroke patients^2^:  Map Search 1 min: =0.84  Map Search 2 min: =.0.85  Visual Elevator accuracy=0.90  Visual Elevator timing score=Not calculated    Moreover, in the Cantonese version, no significance was found between the order of implementation of versions A and B.^4^    Correlation coefficients between A and B in this study^4^ showed:  Map search 1 minute: 0.753  Map search 2 minutes: 0.690  Visual elevator accuracy= 0.462  Visual Elevator timing score= 0.939      In patients with Chronic stroke^5^ :  For Map Search, the intra class correlation coefficients (ICC) varied from 0.57 (forms BC) to 0.72 (forms AB). Comparing the two scores of Visual Elevator, the reliability was good-to-excellent for the time-per-switch score  (ICC=0.70–0.92); the accuracy score had poor reliability for forms AB (ICC=0.49) and BC (ICC=0.36) but good for forms CA (ICC=0.83). | | |
| Internal consistency (A rule of thumb)  0.9 ≤ Cronbach’s alpha (α) Excellent  0.8 ≤ α < 0.9 Good  0.7 ≤ α < 0.8 Acceptable  0.6 ≤ α < 0.7 Questionable  0.5 ≤ α < 0.6 Poor  α < 0.5 Unacceptable  (Streiner, 2003 Journal of Personality Assessment, 80:1, 99-103, DOI: 10.1207/S15327752JPA8001_18) |  | | |
| “Corrected item-to-total correlation” | Y/N, if Y, specify how large  N | | |
| Test/retest | Y/N, if Y, specify how large    Coefficients for the test-retest reliability of versions A to B of the TEA ranged from 0.59 to 0.86 utilising 118 of the normative sample. For versions B to C a sub-sample of 39 from the normative group produced test-retest reliability coefficients ranging from 0.61 to 0.90, while 74 of the stroke sample produced a range of coefficients from 0.41 to 0.90.^1^  Because improvements with practice are seen in some of the subtests, repeated use of the TEA should follow the A, B, C order.^2^  Practice effect:  Significant test-retest differences were consistently observed in Visual Elevator-time per switch (p=0.001–0.019; absolute value of Cohen’s d=0.15– 0.56, small to medium effect) | | |
| Inter-rater | Y/N, if Y, specify how large  Not assessed | | |
| Intra-rater | Y/N, if Y, specify how large  Not assessed | | |
| Other (e.g. Standard Error of Measurement) | Y/N, if Y, specify how large  Not assessed | | |
| **Validity: assessed –good; not good. Not assessed (reference)** | Good.^2^ | | |
| Face and Content validity (does test appears to be measuring what it is intended to measure and do test’s items comprehensively represent the domain of interest?) | The ecological approach the TEA has adopted in using everyday items provides the TEA  with a feeling of high face validity, however, for those with questionable auditory and sensory acuity, its validity may be undermined if steps are not taken to ensure that extraneous sensory difficulties are not ruled out as a confounding factor prior to assessment.^1^ | | |
| Criterion validity (compared to gold-standard) or other criteria for  diagnosis) (e.g. other comparable tests or lesion or MRI research) | No specific data was found. | | |
| Construct validity (correlations with other convergent tests and divergent tests; known-groups comparisons) | Add data regarding:    Convergent validity    As it was mentioned before, factor analysis of attention tests for standardisation sample (N=154) explained 64% of the variance.^2^ Four factors were identified. Map Search loaded on Visual Selective attention/Speed factor (0.84), together with Telephone Search (-0.80), Stroop Test (0.72), Trail B (-0.74) and d2 total (0.67). Visual Elevator (number correct) loaded on Attentional switching (0.78) with WCST categories (0.68).    The TEA subtests correlated with existing tests of attention^2^:  -Map search correlated to Stroop (0.51);^18^  -Visual elevator accuracy and WCST number of categories (0.51).^19^  In stroke patients, Map Search subtests correlate with functional measures Barthel Index (0.45), Extended Activities of Daily Living Scale (0.48). Moreover, a close relative who was able to observe the recovering patient’s behaviour was asked to complete the Rating Scale of Attentional Behaviour, the correlation between measures was -0.30.^1^    In the validation of the Cantonese version on a sample of 49 healthy participants with a mean age of 25.9 years, Chan et al.^4^ showed:    Map search correlated with TMTb (-0.505), SDMT (0.373) and SCWT (0.564). Visual elevator accuracy with Modified Six Elements test (–0.326), whereas Visual elevator timing correlated with TMTb (-0.398), Color trail test 2 (0.421) and SDMT (-0.461).    Factor analyses (63.5% of variance explained) showed that Map search load in *Factor I: Psychomotor efficiency and selective attention* also including Stroop Color-Word Test, Telephone search, Color Trails Test (part 2), Elevator counting with Reversal and Symbol Digit Modalities Test (oral).    Visual elevator timing score load on *Factor III: Attentional switching* also including Elevator counting with distraction.    In van der Leeuw et al 2018(14), the authors reported significant correlation between Visual Elevator and TMT A (r=0.60 p=0.01), TMT B (r=0.49 p=0.01), TMTB-A (r=0.33 p=0.01), but also between Map Search and TMT A (r=-0.49 p=0.01), TMT B (r=-0.51 p=0.01), TMTB-A (r=-0.41 p=0.01).      Divergent validity    Authors reported small correlations with NART-measured verbal intelligence. Only Visual Elevator number correct score has a correlation exceeding 0.3 (0.39), following their suggestion, it is sufficiently small to be ignored for practical purposes. However, as mentioned before, TEA score just below the average in participants with low intelligence should be interpreted with caution. (1)    Following Chan et al 2002 (4), in Cantonese version validation, no significant correlation was found between TEA subtests and the Dysexecutive Questionnaire (only based on n=26) and only a very weak correlation (-0.455 p<0.05) was found between Visual elevator (correct number) and the Cognitive Failures Questionnaire (informant-rated version)    However, in van der Leeuw et al 2018 (14), the authors reported significant correlation between Visual Elevator and Clock in a box (r=-0.41 P=0.01), FAS (r=-0.33 p=0.01) , HVLT Immediate recall (r=-0.17 p=0.05), HVLT delayed recall (r=-0.18 p=0.01) and MMSE (r=-0.37 p=0.01). Map search correlated with Clock in a box (r=0.35 P=0.01), FAS (r=0.34 p=0.01) , HVLT Immediate recall (r=0.37 p=0.01), HVLT delayed recall (r=0.36 p=0.01) and MMSE (r=0.39 p=0.01).      Group-comparisons    Map Search significantly differentiates Controls and Head injured (t=-3.22 p=0.003), but Visual Elevator does not. (1,2)      Map Search, visual elevator accuracy and visual elevator timing significantly differed between controls and stroke patients (p<0.001) in two different age groups (50-64 and 65-80) (1,2)    Chan et al 2000(9) reported significant differences between patients with closed head injury and healthy controls (Map Search ꭕ^2^=14.18 p=0.000, visual elevator ꭕ^2^= 12.040 p=0.001 and visual elevator timing ꭕ^2^=11.620 p=0.001).    Sterr (2003)(13) showed attentional deficits in small young-adult learning disabilities group (n=8) in comparison to controls (n=8) using Map Search (F(1,14) = 41.8; P < .01 ) and visual elevator accuracy (F(1,12) = 11.9; P < .01) and timing (F(1,12) = 11.8; P < .01). | | |
| Reference group used to develop this test:  1) If available, please define the characteristic of the norm group adopted (demographic variables used for stratification: age, education, sex, etc).    2) What kind of standardized score generated (T score, Z score, derived IQ score, percentile ranks, etc)?    3) If translated/validated in other languages, are reference norms created? If so, please specify demographic variables used for stratification (age, education, gender etc). | Group characteristics:    1. N= 154 normal volunteers  Age range 18-80 stratified in four age bands (18-34, 35-49, 50-64 and 65-80) and two levels of education attainment. Sex: 69 males and 85 females  Country: England    The whole sample was included to standardised Version A, only 118 subjects (no reported details) completed Version B one week later. Only 39 subjects completed Version C some weeks (not specified) later.  In addition to this general population sample, a sample of 80 unilateral stroke patients were assessed 2 months post injury. While each sub-group was stratified further with regard to IQ, as measured by the National Adult Reading Test, no data was reported on socio economic, cultural or academic background.^1,2^    2. Scaled-scores (M=10, SD= 3) and percentiles are derived for each subtest based on normative data presented in the manual. There are no composite score.      3. There are no reference norms created for Cantonese version^4^, but the validation was done based on a sample including:    49 normal volunteers whose age ranged from 17 to 51 years with a mean of 25.94 years (SD = 9.42).  The education level ranged from 8 years to 17 years (mean = 13.12, SD = 2.17).  Thirty-three were females and 16 were males.  Country: China (Hong-Kong) | | |
|  |  |  | |
| **Responsiveness: assessed –good; not good. Not assessed (reference)** | Not good | |
| Demonstrated to be sensitive to change (change over time or due to treatment)*    * [determined by the strength of the relationship between change in the test scores and the criterion or anchor scores] | If Yes, please specify    No.    Mazer et al.^9^ compared the effectiveness of a visual attention retraining program using the Useful Field of View with a traditional visuoperception treatment program on the driving performance of clients with stroke. There were no significant differences between groups on any of the outcome measures including TEA subtests.    Richmond et al.^13^ trained working memory in Older Adults and tested transfer to other measures including TEA. There were no significant improvements exhibited on any subtest by either group (specific data was not shown by the authors). They reported that the pattern of the results suggested that there was a ceiling effect (Visual elevator accuracy).    Zickefoose et al.^20^ also studied computer-based treatments in four adult males with severe TBI, the participants demonstrated statistically significant  progress in reaching new levels of difficulty on intervention tasks over the course of treatment. However, limited evidence on generalization of the results measured by TEA were found.    Dymowski et al.^10^ reported that TEA was insensitive to small changes in attentional performance after interventions in very small sample of traumatic brain injured (TBI) patients    A pilot study of Dance for Parkinson’s -a community-based program- compare effect sizes across multiple outcomes between two PD patients groups (intervention and control). The largest between-group differences were observed for Visual Elevator timing, as a measure of cognitive switching (Between Group: Effect Size Difference +2.06 (0.45, 3.68)).^21^ | |
| Has the minimal clinically important change and minimal clinically relevant incremental difference been assessed?  (besides a statistically significant improvement) | Minimal clinically important change assessed Y/N?    No | |
| Has this test been assessed or used in patients with other condition than that of interest? | Y/N, please specify if one or several condition    Yes (e.g., stroke, trauma brain injury, Alzheimer’s disease, Progressive Supranuclear Palsy, Parkinson’s disease). | |
| Other |  | |
| **Overall impression** |  | |
| Strengths | The assessed subtests measure important clinical and theoretical aspects of attention.    TEA has three versions which allow testing on three successive occasions with parallel material.  It can be used in pathology and normal subjects. Sensitive enough to show normal age effects in the normal population.  Scaled scores are provided.  Practice effects can be controlled.  TEA subtests are based on everyday materials. | |
| Weaknesses | General:  Evidence in PD is scarce.  TEA subtests showed low sensitivity to change.  Whereas convergent validity is good, the results regarding divergent validity are less conclusive.  Map Search:  Vision problems can limit the use of this subtest.  The map search could be difficult for patients in advanced motor impairment stages.  Map Search shows ceiling effect in young age groups (18-34 and 35-49).  Visual Elevator:  Visual Elevator raw accuracy showed ceiling effect, correlates with intelligence measures and would not be feasible for older adults (>80) with severe cognitive impairments. | |

| **Level of Recommendation** |  |
| --- | --- |
| “Recommended” – The test was applied to the target population, AND was studied clinimetrically and found to be valid, reliable and sensitive to change, AND was used in studies by researchers, others than the developers    “Recommended with caveats” – Test’s properties were generally found to be adequate, but some of the measurement properties were not evaluated or not evaluated specifically in a PD cohort."    ‘Suggested’ – The test was applied to the target population, but only one of the other criteria applies    ‘Listed” – The test was applied to the target population, but none of the other criteria applies | Please choose one of these options      Map Search: Suggested      Visual Elevator: Suggested |
| Which type of study is this test suitable for and which type of study is it not suitable for (screening, prevalence, etiological (e.g. case-control or genetic), treatment trial, correlation with biological markers or other scales, e.g. of parkinsonism, clinical practice for diagnosis/ screening). List all. | Suitable for:  Screening  Prevalence  Differential reasoning (etiological)  **Case-control study**  Treatment trial  Biomarker study  Other    Unsuitable for (please add just categories for which the measure is critically unsuitable):  Screening  Prevalence  Differential reasoning (etiological)  Case-control study  **Treatment trial**  Biomarker study  Other |

**REFERENCES**

1. Robertson IH, Ward T, Ridgeway V, Nimmo-Smith Ian. TEA Test of Everyday Attention [Internet]. Harcourt Assessment. Protcter Houe. 1 protecter Street. London. 1994

2. Robertson IH, Ward T, Ridgeway V, Nimmo-Smith I. The structure of normal human attention: The Test of Everyday Attention. Journal of the International Neuropsychological Society. 1996;2(6):525–34.

3. van der Leeuw G, Leveille SG, Jones RN, et al. Measuring attention in very old adults using the Test of Everyday Attention. Aging, Neuropsychology, and Cognition. 2017; 24(5):543–54.

4. Chan RCK, Hoosain R, Lee TMC. Reliability and validity of the Cantonese version of the test of everyday attention among normal Hong Kong Chinese: A preliminary report. Clinical Rehabilitation. 2002; 16(8):900–9.

5. Chen HC, Koh CL, Hsieh CL, Hsueh IP. Test of everyday attention in patients with chronic stroke: Test-retest reliability and practice effects. Brain Injury. 2013;27(10):1148–54.

6. Myall DJ, Horne KL, MacAskill MR, et al. Cognitive tests that identify high risk of conversion to dementia in Parkinson’s disease. bioRxiv. 2020-05.

7. Peralta C, Stampfer-Kountchev M, Karner E, et al. Orthostatic hypotension and attention in Parkinson’s disease with and without dementia. Journal of neural transmission. 2007; 114: 585-588.

8. Doneva S, Davis S, Cavenagh P. Comparing the performance of people who stutter and people who do not stutter on the Test of Everyday Attention. Journal of Clinical and Experimental Neuropsychology. 2018; 40(6):544–58.

9. Mazer BL, Sofer S, Korner-Bitensky N, Gelinas I, Hanley J, Wood-Dauphinee S. Effectiveness of a visual attention retraining program on the driving performance of clients with stroke. Archives of Physical Medicine and Rehabilitation. 2003;84(4):541–50.

10. Dymowski AR, Ponsford JL, Willmott C. Cognitive training approaches to remediate attention and executive dysfunction after traumatic brain injury: A single-case series. Neuropsychological Rehabilitation. 2016;26(5–6):866–94.

11. Chan RCK. Attentional deficits in patients with closed head injury: A further study to the discriminative validity of the test of everyday attention. Brain Injury. 2000;14(3):227–36.

12. Chan RCK, Lai MK. Latent structure of the Test of Everyday Attention: Convergent evidence from patients with traumatic brain injury. Brain Injury. 2006;20(6):653–9.

13. Richmond LL, Morrison AB, Chein JM, Olson IR. Working memory training and transfer in older adults. Psychology and Aging. 2011;26(4):813–22.

14. Stapleton T, Connolly D. Occupational therapy practice in predriving assessment post stroke in the irish context: Findings from a nominal group technique meeting. Topics in Stroke Rehabilitation. 2010;17(1):58–68.

15. Sterr AM. Attention performance in young adults with learning disabilities. Learning and Individual Differences. 2004;14(2):125–33.

16. van der Leeuw G, Leveille SG, Jones RN, et al. Measuring attention in very old adults using the Test of Everyday Attention. Aging, Neuropsychology, and Cognition. 2017;24(5):543–54.

17. Zygouris S, Ntovas K, Giakoumis D, et al. A Preliminary Study on the Feasibility of Using a Virtual Reality Cognitive Training Application for Remote Detection of Mild Cognitive Impairment. Journal of Alzheimer’s Disease. 2017;56(2):619–27.

18. Trenerry MR, Crosson B, DeBoe J, Leber WR.  Stroop Neuropsychological Screening Test. Odessa, FL: Psychological Assessment; 1989.

19. Nelson, H.  A modified card sorting test sensitive to frontal lobe deficits. Cortex. 1976; 12: 313–324.

20. Zickefoose S, Hux K, Brown J, Wulf K. Let the games begin: A preliminary study using Attention Process Training-3 and Lumosity^TM^ brain games to remediate attention deficits following traumatic brain injury. Brain Injury. 2013;27(6):707–16.

21. Ventura MI, Barnes DE, Ross JM, Lanni KE, Sigvardt KA, Disbrow EA. A pilot study to evaluate multi-dimensional effects of dance for people with Parkinson’s disease. Contemporary clinical trials. 2016; 51:50.

**Rating test evaluation**

| **Test name:** | **Serial Reaction Time Task** |
| --- | --- |
| **Version**  Are there several versions (alternate or parallel) of the test? If so, which one has been assessed? | Yes.  In the classical version a visual stimulus repeatedly appears in one of four spatial locations on a computer display i.e., 4 choice SSRT (1).  Other versions exist e.g., 5-choice serial reaction time task, ocular version of the serial reaction time task, versions in which ﻿participants indicate the location of the visual stimulus with a verbal response (2–5).  Versions may vary in the ﻿choice of sequence type (6), in the length of the sequence or in the number of times the sequence is presented, which may have an impact on studies’ findings (7,8).  Only implicit versions of the task (i.e., participants are not informed of the sequence), but not explicit versions of the task have been assessed here. |
| **Description of test**   - What population the test was originally developed for? - Which domains/components of domains do the test assess? - Are there multiple domains or a predominant domain assessed? - Does it score impaired/unimpaired? - Does it score severity   (subtle/mild/moderate/severe)? | - Patients with amnesia (Korsakoff’s syndrome). - Memory (Implicit sequence learning) - No multiple domains - The task assesses whether individuals are able to learn sequence. The increase in the reaction times (RT) from the sequence block to the random block suggests that knowledge about the sequence has been learnt. - It has mainly used in experimental paradigms. No cut-off score or severity indexes have been found. |
| **Scaling Metric**: Type of response format? (e.g., Likert, Binary, multiple-choice, continuum, reaction time, etc.) | ﻿The key dependent variable is reaction time i.e., faster response times to the repeating sequential pattern vs randomly  sequenced material or material following an alternative (non-practiced) sequential structure (9).  Other responses are accuracy data (error rates), minimum reaction time (10) |
| **Respondent: Patient:** any specific requirements for the target study population (e.g. non-demented, motor, auditory, visual abilities, etc)? | Motor ability of pressing on a button with one of four fingers, preserved vision. |
| **Access:** How can the test be obtained (mail address or website)? | The test is free to use. Information on how to design the task can be found in the original paper (1) or in other works (11). |
| Copyrighted or in public domain?  Conditions for use? Fee? | It is not copyrighted.  It is free. The task can be designed using computer software such as e-prime (11). |
| **Translations:** Has the test been translated and validated in other languages besides English? | It is a visuospatial task. No translation is needed. |

| **Test properties**  (Please provide references for all statements or note if this is your judgment) |  |
| --- | --- |
| Which cognitive domain (or component/specific aspect of a cognitive domain) does this test *primarily* measure? | Memory - implicit sequence learning. |
| Which aspects of the domain you are assessing are not covered? | Episodic memory, semantic memory, prospective memory (personal judgements) |

| **Use** |  |
| --- | --- |
| Was it *designed* to measure severity, screen or diagnosis of the domain? | Screen. In particular, it has been designed “﻿to investigate the attentional requirements of learning as assessed by performance measures rather than by introspection” (1).  However, it is an experimental paradigm and no cut-off score (for screening purposes) or severity index have been found. |
| Is there a cut-off score? Are there measures of screening/diagnostic performance of the test? (Receiver characteristic curve analyses; sensitivity/specificity; Positive Predictive Value/Negative Predictive Value), True Positive Rate, False Positive Rate etc.) | No cut-off score found. |
| Is this appropriate for the PD population? If not, why (low sensitivity and/or specificity values)? | No cut-off score found. |
| **Clinimetric/psychometric properties in the target population** |  |
| **Feasibility /Acceptability** |  |
| Length | ﻿In the original task, there were eight blocks of 100 trials. Successive blocks were separated by a short rest period of 1.5 to 2 min (1).  ﻿Stimulus presentations are usually grouped into blocks typically comprising around 80 to 100 stimuli, although this figure varies substantially between studies (12).  There is also variability across studies in the number of blocks of trials e.g., 15 blocks (13). |
| Ambiguities in instructions to patient | No |
| Ambiguities in rating anchors | No |
| Appropriateness of questions for population | Yes |
| Applicability across disease cognitive stages | Yes. It has been used in MCI (14), or in patients with dementia e.g., Alzheimer’s disease (3).  Firouzi et al (2021) evaluate the effect of tDCS over the primary motor cortex to enhance implicit motor sequence learning in a sample of PD MCI patients(15) |
| Applicability across disease motor severity? | Yes, it has been administered in PD patients with H&Y score: 1 to 3 (3,13,16–18).  No studies using the task in PD patients with H&Y >4 have been found. |
| Clear instructions to raters | Yes |
| Has the test been used by researcher other than developers?  By other groups  Not applicable? | Yes, and by many other groups |
| Are there instructions for dealing with missing data?  Not applicable? | Not found. |
| Floor and ceiling effects, skewed score distributions? | Not found. |

| **Have the component of dimensionality, variability and dependency structures been analyzed?**  (Exploratory and confirmatory factor analysis (EFA and CFA)? | Not found. |
| --- | --- |
|  |  |
| **Reliability: assessed – good; not good. Not assessed**  Are alternative versions developed?  If so, were they validated with reliability measures? | Mainly not assessed. |
| Internal consistency (A rule of thumb)  0.9 ≤ Cronbach’s alpha (α) Excellent  0.8 ≤ α < 0.9 Good  0.7 ≤ α < 0.8 Acceptable  0.6 ≤ α < 0.7 Questionable  0.5 ≤ α < 0.6 Poor  α < 0.5 Unacceptable  (Streiner, 2003 Journal of Personality Assessment, 80:1, 99-103, DOI: 10.1207/S15327752JPA8001_18) | Not found. |
| “Corrected item-to-total correlation” | Not found. |
| Test/retest | Not found in PD.  In healthy adults, reliability is poor: Baseline RTs were reliable across runs performed the same day (r = 0.63, *p=* 0.001, 95% CI [0.43, 0.77]). The measure of variability, the SD of these baseline RTs, was not reliable (r = - 0.12, *p* = 0.38, 95% CI [-0.38, 0.15]). The measures of sequence learning were not reliable when taken at the end of the runs (r = 0.07, *p =* 0.63, 95% CI [-0.21, 0.33]). On an individual basis, learning in one run was not predictive of learning in a second run (19). |
| Inter-rater | Not found |
| Intra-rater | Not found |
| Other (e.g. Standard Error of Measurement) | NA |
| **Validity: assessed –good; not good. Not assessed (reference)** |  |
| Face and Content validity (does test appears to be measuring what it is intended to measure and do test’s items comprehensively represent the domain of interest?) | Not found. |
| Criterion validity (compared to gold-standard) or other criteria for  diagnosis) (e.g. other comparable tests or lesion or MRI research) | In healthy adults, SRTT variants revealed significant brain activation convergence between cortical foci in bilateral dorsal premotor cortex (dPMC) and suplementary motor cortex (SMA), as well as left M1, left superior parietal lobe, left thalamus and right cerebellum. Moreover, activations in the bilateral dPMC and SMA, as well as in left SPL and thalamus were stronger when participants had explicit awareness of the presence of a repeating sequence, whereas only the head of the left caudate was more consistently recruited in implicit tasks. (20). |
| Construct validity (correlations with other convergent tests and divergent tests; known-groups comparisons) | Convergent validity  In PD:   - scores on the SCOPA-COG (r = 0.62, p < 0.05), and in particular scores on the subscales ‘memory and learning’ (r = 0.68, p < 0.05) and ‘executive functions’ (r = 0.50, p < 0.05) correlated positively with SRTT performance (17). - the accuracy after the initial learning in the SRTT (i.e., the difference between the fifth and fourth blocks) is highly correlated to visual span capacity WMS-R (r = 0.59) (2).   Divergent validity  In PD:   - No correlation between WCST and SRTT (18)   In healthy adults:   - ﻿no correlation between visuomotor adaptation task and the SRTT (19). |
| Reference group used to develop this test:   1. If available, please define the characteristic of the norm group adopted (demographic variables used for stratification: age, education, sex, etc). 2. What kind of standardized score generated (T score, Z score, derived IQ score, percentile ranks, etc)? 3. If translated/validated in other languages, are reference norms created? If so, please specify demographic variables used for stratification (age, education, gender etc). | ﻿From Nissen and Bullemer (1987), experiment 4:  Individuals with memory disorders resulting from Korsakoff’s syndrome:  Mean age of 65.2 years (range 58-72)  no information about education  Sex (16.7% women)  USA  The normal control group:  Mean age of 65.7 years (range 61-71)  no information about education  Sex (14.3% women)  USA  2) no standard score has been generated.  3) not validated in other languages (no verbal stimuli) |

| **Responsiveness: assessed –good; not good. Not assessed (reference)** |  |
| --- | --- |
| Demonstrated to be sensitive to change (change over time or due to treatment)*  * [determined by the strength of the relationship between change in the test scores and the criterion or anchor scores] | Previous studies evidenced sensitivity to change after intervention (15,20–22) |
| Has the minimal clinically important change and minimal clinically relevant incremental difference been assessed?  (besides a statistically significant improvement) | Not found. |
| Has this test been assessed or used in patients with other condition than that of interest? | Yes.  e.g., stroke (23), Korsakoff’s syndrome (1), bipolar disorder (24), schizophrenia, autism spectrum disorder, dyslexia, specific language impairment, developmental coordination disorder (25). |
| Other |  |
| **Overall impression** |  |
| Strengths | It is a very basic and simple task. Unlike other tasks that are confounded with language (e.g., artificial grammar, mirror reading) or executive functions (e.g., Tower of Hanoi, Tower of Toronto) the SRT task only requires quick response to a square illuminated in red appearing on the screen (26). |
| Weaknesses | - ﻿Lacking information on reliability. - Motor demand, although this can be reduced by asking participants to provide verbal responses (4,7,27) - If the sequence structure is insufficiently concealed, mechanisms of explicit learning may be engaged (13). - Participants had greater difficulty relearning at Time 2 than Time 1 indicating potential interference from the previous administration (28). |

| **Level of Recommendation** |  |
| --- | --- |
| “Recommended” – The test was applied to the target population, AND was studied clinimetrically and found to be valid, reliable and sensitive to change, AND was used in studies by researchers, others than the developers  “Recommended with caveats” – Test’s properties were generally found to be adequate, but some of the measurement properties were not evaluated or not evaluated specifically in a PD cohort."  ‘Suggested’ – The test was applied to the target population, but only one of the other criteria applies  ‘Listed” – The test was applied to the target population, but none of the other criteria applies | ‘Suggested’ – The test was applied to the target population AND was used in studies by researchers, others than the developers. |
| Which type of study is this test suitable for and which type of study is it not suitable for (screening, prevalence, etiological (e.g. case-control or genetic), treatment trial, correlation with biological markers or other scales, e.g. of parkinsonism, clinical practice for diagnosis/ screening). List all. | Suitable for:  case-control study  correlation with biological markers or other scales  Not suitable:  screening, prevalence – no cut-off scores available.  treatment trials – no studies are available assessing minimal clinically important change or sensitivity to change. |

**REFERENCES**

1. Nissen MJ, Bullemer P. Attention Requirements of Learning Evidence from Performance Measures. Cognitive Psychology. 1987;19:1–32.

2. Stefanova ED, Kostic VS, Ziropadja L, Markovic M, Ocic GG. Visuomotor skill learning on serial reaction time task in patients with early Parkinson’s disease. Movement Disorders. 2000;15(6):1095–103.

3. Van Tilborg IADA, Hulstijn W. Implicit motor learning in patients with Parkinson’s and Alzheimer’s disease: Differences in learning abilities? Motor Control. 2010;14(3):344–61.

4. Smith JG, McDowall J. Impaired higher order implicit sequence learning on the verbal version of the serial reaction time task in patients with Parkinson’s disease. Neuropsychology. 2004;18:679–691.

5. Sommer M, Grafman J, Clark K, Hallett M. Learning in Parkinson’s disease: Eyeblink conditioning, declarative learning, and procedural learning. Journal of Neurology Neurosurgery and Psychiatry. 1999;67:27–34.

6. Schwarb H, Schumacher EH. Generalized lessons about sequence learning from the study of the serial reaction time task. Advances in Cognitive Psychology. 2012;8(2):165–78.

7. Clark GM, Lum JAG, Ullman MT. A meta-analysis and meta-regression of serial reaction time task performance in Parkinson’s disease. Neuropsychology. 2014;28(6):945–58.

8. Siegert RJ, Taylor KD, Weatherall M, Abernethy DA. Is implicit sequence learning impaired in Parkinson’s disease? A meta-analysis. Neuropsychology. 2006;20(4):490–5.

9. Zhao F, Gaschler R, Nöhring DO, Röttger E, Haider H. Sequential modulation of across-task congruency in the serial reaction time task. Acta Psychologica. 2020;205(March):103043.

10. You ZF, Wu YY, Wu R, Xu ZX, Wu X, Wang XP. Efforts of subthalamic nucleus deep brain stimulation on cognitive spectrum: From explicit to implicit changes in the patients with Parkinson’s disease for 1 year. CNS Neuroscience and Therapeutics. 2020;26(9):972–80.

11. Trofimova O, Mottaz A, Allaman L, Chauvigné LAS, Guggisberg AG. The “implicit” serial reaction time task induces rapid and temporary adaptation rather than implicit motor learning. Neurobiology of Learning and Memory. 2020;175(July):107297.

12. Lum JAG, Ullman MT, Conti-Ramsden G. Procedural learning is impaired in dyslexia: Evidence from a meta-analysis of serial reaction time studies. Research in Developmental Disabilities. 2013;34(10):3460–3476.

13. Beigi M, Wilkinson L, Gobet F, Parton A, Jahanshahi M. Levodopa medication improves incidental sequence learning in Parkinson’s disease. Neuropsychologia. 2016;93(September):53–60.

14. Hong Y, Alvarado RL, Jog A, Greve DN, Salat DH. Serial Reaction Time Task Performance in Older Adults with Neuropsychologically Defined Mild Cognitive Impairment. Journal of Alzheimer’s Disease. 2020;74(2):491–500.

15. Firouzi M, Van Herk K, Kerckhofs E, Swinnen E, Baeken C, Van Overwalle F, et al. Transcranial direct-current stimulation enhances implicit motor sequence learning in persons with Parkinson’s disease with mild cognitive impairment. J Neuropsychol [Internet]. 2021 Sep 1 [cited 2025 Mar 20];15(3):363–78. Available from: https://onlinelibrary.wiley.com/doi/full/10.1111/jnp.12231

16. Muslimović D, Post B, Speelman JD, Schmand B. Motor procedural learning in Parkinson’s disease. Brain. 2007;130(11):2887–97.

17. Deroost N, Kerckhofs E, Coene M, Wijnants G, Soetens E. Learning sequence movements in a homogenous sample of patients with Parkinson’s disease. Neuropsychologia. 2006;44(10):1653–62.

18. Gawrys L, Szatkowska I, Jamrozik Z, Janik P, Friedman A. Nonverbal deficits in explicit and implicit memory of PD patients. acta neurobiol Exp. 2008;68:58–72.

19. Stark-Inbar A, Raza M, Taylor JA, Ivry RB. Individual differences in implicit motor learning: Task specificity in sensorimotor adaptation and sequence learning. Journal of Neurophysiology. 2017;117(1):412–28.

20. Benninger DH, Lomarev M, Lopez G, Wassermann EM, Li X, Considine E, et al. Transcranial direct current stimulation for the treatment of Parkinson’s disease. J Neurol Neurosurg Psychiatry [Internet]. 2010 [cited 2025 Mar 20];81(10):1105–11. Available from: https://pubmed.ncbi.nlm.nih.gov/20870863/

21. Beigi M, Wilkinson L, Gobet F, Parton A, Jahanshahi M. Levodopa medication improves incidental sequence learning in Parkinson’s disease. Neuropsychologia [Internet]. 2016 Dec 1 [cited 2025 Mar 20];93(Pt A):53–60. Available from: https://pubmed.ncbi.nlm.nih.gov/27686948/

22. Duchesne C, Lungu O, Nadeau A, Robillard ME, Boré A, Bobeuf F, et al. Enhancing both motor and cognitive functioning in Parkinson’s disease: Aerobic exercise as a rehabilitative intervention. Brain Cogn. 2015 Oct 1;99:68–77.

23. Kal E, Winters M, Van Kamp J Der, Houdijk H, Groet E, Bennekom C, et al. Is implicit motor learning preserved after stroke? A systematic review with meta-analysis. PLoS ONE. 2016;11(12):1–23.

24. Chrobak AA, Siuda-Krzywicka K, Siwek GP, Arciszewska A, Siwek M, Starowicz-Filip A, et al. Implicit motor learning in bipolar disorder. Journal of Affective Disorders. 2015;174:250–6.

25. Clark GM, Lum JAG. Procedural learning in Parkinson’s disease, specific language impairment, dyslexia, schizophrenia, developmental coordination disorder, and autism spectrum disorders: A second-order meta-analysis. Brain and Cognition. 2017;117(July):41–8.

26. Vakil E, Schwizer Ashkenazi S, Nevet-Perez M, Hassin-Baer S. Implicit sequence learning in individuals with Parkinson’s disease: The added value of using an ocular version of the serial reaction time (O-SRT) task. Brain and Cognition. 2021;147(October 2020):105654.

27. Smith J, Siegert RJ, McDowall J, Abernethy D. Preserved implicit learning on both the serial reaction time task and artificial grammar in patients with Parkinson’s disease. Brain and Cognition. 2001;45:378–391.

28. Buelow MT, Amick MM, Queller S, Stout JC, Friedman JH, Grace J. Feasibility of use of probabilistic reversal learning and serial reaction time tasks in clinical trials of Parkinson’s disease. Parkinsonism and Related Disorders. 2015;21(8):894–8.

**Rating test evaluation**

| **Test name:** | Digit Span WMS(1) |
| --- | --- |
| **Version**  Are there several versions (alternate or parallel) of the test? If so, which one has been assessed? | There are several previous versions of the WMS. The Digit Span subtest, however, is equal for all the versions. For the present evaluation I assessed the Digit Span subtest of the WMS-IIII(1) |
| **Description of test**   - What population the test was originally developed for? - Which domains/components of domains do the test assess? - Are there multiple domains or a predominant domain assessed? - Does it score impaired/unimpaired? - Does it score severity   (subtle/mild/moderate/severe)? | The test was developed to assess people between 16 and 89.  Subjects are read a sequence of numbers and asked to repeat the same sequence back to the examiner in order (forward span) or in reverse order (backward span).  Forward span is mostly associated with immediate auditory-verbal memory and attention. Backward span is associated with working memory.  The digit forward span has a relatively restricted range and 89% of the subjects show spans within the 5–8 digit range(2). The normal range for forward digits is 6+1(3), and education has an effect on the score(4, 5). Age minimally affects forward span beyond the ages of 65 or 70 years(6). It is important to point out that differences in digit span forward performance between English and Spanish speakers (and probably other languages) are probably due to the greater number of syllables per digit in the Spanish language(7).  The backward span involves mental tracking with verbal and visual processes and includes a strong WM component. It is distinct from the more passive span measured by Digits Forward. The normal raw score difference between forward digits and backward digits tends to range around 1 (from 0.59 to 2). The normal span for backward digits is 4–5, and a score of 3 is considered borderline defective or defective depending on the educational level of the subject. Scores typically decrease about one point beyond age 70, but not all older subjects get lower scores than younger ones.  It is assumed that attention and working memory are different cognitive domains. However, because of the cognitive resources recruited to overcome backward span, it is assumed that in this condition, mental imagery also participates.  The digit span can be scores as a total score from the sum of correct responses in the forward and backward condition. There is age-normed data when the test is scored following this method and thus, both impairment and severity can be addressed. The forward and backward condition can be scored separately. In this approach, performance can be scored based on number of correct items OR maximum span achieved.  Depending on the country, there are normative age-education corrected data (i.e.: Neuronorma study for Spanish population)(6).  Normative data allows the conversion of raw scores into scaled scores and percentile ranks. Thus, severity and presence/absence of impairment can be easily achieved. |
| **Scaling Metric**: Type of response format? (e.g., Likert, Binary, multiple-choice, continuum, reaction time, etc.) | Binary (correct / Incorrect) for each trial. The summatory score for overall performance |
| **Respondent: Patient:** any specific requirements for the target study population (e.g. non-demented, motor, auditory, visual abilities, etc)? | Because of the auditory nature of the task, hearing deficits must be taken into account. |
| **Access:** How can the test be obtained (mail address or website)? | The Digit Span test is a subtest the Wechsler Memory Scales (WMS). The WMS is licensed by Pearson Clinical. Formally, the subtest is copyrighted and although its use in clinical and research setting is free, the test must be acquired by purchasing the entire scale. |
| Copyrighted or in public domain?  Conditions for use? Fee? | Copyrighted |
| **Translations:** Has the test been translated and validated in other languages besides English? | Yes. No modifications have been done in the existing versions(6). |

| **Test properties**  (Please provide references for all statements or note if this is your judgment) |  |
| --- | --- |
| Which cognitive domain (or component/specific aspect of a cognitive domain) does this test *primarily* measure? | Attention and working memory(6) |
| Which aspects of the domain you are assessing are not covered? | The attention domain is covered just taking into account immediate verbal-auditory attention. Sustained or selective attention, or other forms of attention are not covered. |

| **Use** |  |
| --- | --- |
| Was it *designed* to measure severity, screen or diagnosis of the domain? | It was designed to assess functional (below and beyond the normal mean) of the processes covered by this task. It allows the diagnostic approach to deficits at level of immediate verbal memory/attention and working memory and to address severity of the deficit |
| Is there a cut-off score? Are there measures of screening/diagnostic performance of the test? (Receiver characteristic curve analyses; sensitivity/specificity; Positive Predictive Value/Negative Predictive Value), True Positive Rate, False Positive Rate etc.) | Cut-off score in relation to the diagnostic unit (MCI/dementia)  NO |
| Is this appropriate for the PD population? If not, why (low sensitivity and/or specificity values)? | Is it appropriate and has been extensively used in PD population. However, other test to assess attention and working memory appears to be more useful in PD population(8). |
| **Clinimetric/psychometric properties in the target population** |  |
| **Feasibility /Acceptability** |  |
| Length | 14 trials for forward + 14 trials for backward conditions. Time is of about 5-mins |
| Ambiguities in instructions to patient | No |
| Ambiguities in rating anchors | No |
| Appropriateness of questions for population | Yes, just required to repeat numbers in a given order |
| Applicability across disease cognitive stages | Yes |
| Applicability across disease motor severity? | H&Y score: 1 (Unilateral involvement only) = YES  H&Y score: 2 or 3 (bilateral involvement with or without postural instability) = YES  H&Y score: ≥4 (several disabilities or confined to bed) = difficulties due to language impairment/production |
| Clear instructions to raters | Y. Detailed instructions about stimuli presentation and scoring are provided in the manual. |
| Has the test been used by researcher other than developers?  By other groups  Not applicable? | Yes. Used in several studies(9-11). |
| Are there instructions for dealing with missing data?  Not applicable? | NA |
| Floor and ceiling effects, skewed score distributions? | In a validation study with HC, no ceiling/floor effects were reported(12), but education level is a predictor of performance in both the forward and backward condition |

| **Have the component of dimensionality, variability and dependency structures been analyzed?**  (Exploratory and confirmatory factor analysis (EFA and CFA)? | N |
| --- | --- |
|  |  |
| **Reliability: assessed – good; not good. Not assessed**  Are alternative versions developed?  If so, were they validated with reliability measures? | Psychometric properties are just provided for the whole WMS, not the subtests. In any case, several language-validation studies and normative data studies has been done in different languages, providing each of them the specific psychometric properties (if addressed) |
| Internal consistency (A rule of thumb)  0.9 ≤ Cronbach’s alpha (α) Excellent  0.8 ≤ α < 0.9 Good  0.7 ≤ α < 0.8 Acceptable  0.6 ≤ α < 0.7 Questionable  0.5 ≤ α < 0.6 Poor  α < 0.5 Unacceptable  (Streiner, 2003 Journal of Personality Assessment, 80:1, 99-103, DOI: 10.1207/S15327752JPA8001_18) | The Digit Span subscale score internal consistency reliabilities associated with the Wechsler-Bellevue and the WAIS normative samples were reported to be .67 and .68, respectively(13, 14).  Based on the work of Blackburn and Benton(15), later editions of the Wechsler scales revised the administration protocol of the DSF and DSB tests such that both trials within an item were administered, regardless of whether the first trial was completed successfully by the participant.  The consequence of this change in administration procedure was an appreciable increase in the internal consistency reliability of the Digit Span subscale scores.  The mean (across age groups) internal consistency reliability of the DSF and DSB test scores was reported at .81and .82, respectively(16). |
| “Corrected item-to-total correlation” | N |
| Test/retest | Yes. Test-retest reliabilities for all age groups over a two- to twelve-week interval mostly ranged between .62 and .82 for the individual subtests of the WMS and between .75 and .88 for the indexes. |
| Inter-rater | Specific inter-rater and intra-rater values are provided in the WMS-IV technical manual. |
| Intra-rater | Specific inter-rater and intra-rater values are provided in the WMS-IV technical manual. |
| Other (e.g. Standard Error of Measurement) | Specific inter-rater and intra-rater values are provided in the WMS-IV technical manual. |
| **Validity: assessed –good; not good. Not assessed (reference)** |  |
| Face and Content validity (does test appears to be measuring what it is intended to measure and do test’s items comprehensively represent the domain of interest?) | Yes. Extensive review available(17). |
| Criterion validity (compared to gold-standard) or other criteria for  diagnosis) (e.g. other comparable tests or lesion or MRI research) | Yes, against several lesion studies and MRI metrics(18). |
| Construct validity (correlations with other convergent tests and divergent tests; known-groups comparisons) | Good. |
| Reference group used to develop this test:   1. If available, please define the characteristic of the norm group adopted (demographic variables used for stratification: age, education, sex, etc). 2. What kind of standardized score generated (T score, Z score, derived IQ score, percentile ranks, etc)? 3. If translated/validated in other languages, are reference norms created? If so, please specify demographic variables used for stratification (age, education, gender etc). | Group characteristics:  N=1250 subjects aged between 16 - 89  IQ Score, percentile rank and E score.  Add the language of translation and group characteristics:  The WMS-III is available in several languages. For each language-validation study different samples and populations were included. Will be needed to gain access to each specific technical manual for each specific language.  In parallel, several independent studies performed country/language-specific validation studies providing related normative data.  For next steps in the production of this work, we will have to decide if there is a need to review the technical manuals or the validation studies performed independently |

| **Responsiveness: assessed –good; not good. Not assessed (reference)** |  |
| --- | --- |
| Demonstrated to be sensitive to change (change over time or due to treatment)*  * [determined by the strength of the relationship between change in the test scores and the criterion or anchor scores] | Not assessed |
| Has the minimal clinically important change and minimal clinically relevant incremental difference been assessed?  (besides a statistically significant improvement) | No |
| Has this test been assessed or used in patients with other condition than that of interest? | Yes, several conditions (i.e.: ADHD, neurodevelopmental disorders, HD, AD, FTD, TBI, vascular dementia, Stroke, psychiatric conditions,…) |
| Other |  |
| **Overall impression** |  |
| Strengths | It is easy to administer and is not really affected at all by education. Moreover, there are age-education corrected normative data available in several languages. It is a well-recognized instrument. Short time of administration. |
| Weaknesses | Does not appear as the best to be used in the context of PD for the purpose of measurement of attention. |

| **Level of Recommendation** |  |
| --- | --- |
| “Recommended” – The test was applied to the target population, AND was studied clinimetrically and found to be valid, reliable and sensitive to change, AND was used in studies by researchers, others than the developers  “Recommended with caveats” – Test’s properties were generally found to be adequate, but some of the measurement properties were not evaluated or not evaluated specifically in a PD cohort."  ‘Suggested’ – The test was applied to the target population, but only one of the other criteria applies  ‘Listed” – The test was applied to the target population, but none of the other criteria applies | Suggested |
| Which type of study is this test suitable for and which type of study is it not suitable for (screening, prevalence, etiological (e.g. case-control or genetic), treatment trial, correlation with biological markers or other scales, e.g. of parkinsonism, clinical practice for diagnosis/ screening). List all. | Suitable for:  Clinical assessment  Screening  Prevalence  Case-control study  Treatment trial  Biomarker study  Unsuitable for (please add just categories for which the measure is critically unsuitable):  Differential reasoning (etiological) |

**REFERENCES**

1. Corporation PJSA, TX: Author**.** WAIS-III WMS-III Technical Manual. 1997.

2. Kaplan E**.** WAIS-R as a Neuropsychological Instrument (WAIS-R NI): Psychological Corporation; 1991.

3. Miller GA**.** The magical number seven plus or minus two: some limits on our capacity for processing information. (0033-295X (Print)).

4. Ardila A, Rosselli MJDN**.** Neuropsychological characteristics of normal aging. 1989;5:307-20.

5. Kaufman AS, McLean Je Fau - Reynolds CR, Reynolds CR**.** Sex, race, residence, region, and education differences on the 11 WAIS-R subtests. (0021-9762 (Print)).

6. Pena-Casanova J, Quinones-Ubeda S, Quintana-Aparicio M, Aguilar M, Badenes D, Molinuevo JL, et al. Spanish Multicenter Normative Studies (NEURONORMA Project): norms for verbal span, visuospatial span, letter and number sequencing, trail making test, and symbol digit modalities test. Arch Clin Neuropsychol. 2009;24(4):321-41.

7. Olazaran J, Jacobs Dm Fau - Stern Y, Stern Y**.** Comparative study of visual and verbal short-term memory in English and Spanish speakers: testing a linguistic hypothesis. (1355-6177 (Print)).

8. Goldman JG, Holden S, Ouyang B, Bernard B, Goetz CG, Stebbins GT**.** Diagnosing PD-MCI by MDS Task Force criteria: how many and which neuropsychological tests? Mov Disord. 2015;30(3):402-6.

9. Warden C, Hwang J, Marshall A, Fenesy M, Poston KL**.** The effects of dopamine on digit span in Parkinson's disease. J Clin Mov Disord. 2016;3:5.

10. Salmi J, Ritakallio L, Fellman D, Ellfolk U, Rinne JO, Laine M**.** Disentangling the Role of Working Memory in Parkinson's Disease. Front Aging Neurosci. 2020;12:572037.

11. Fallon SJ, Gowell M, Maio MR, Husain M**.** Dopamine affects short-term memory corruption over time in Parkinson's disease. NPJ Parkinsons Dis. 2019;5:16.

12. Hester RL, Kinsella GJ, Ong B**.** Effect of age on forward and backward span tasks. J Int Neuropsychol Soc. 2004;10(4):475-81.

13. Wechsler D**.** The measurement of adult intelligence. Baltimore, MD, US: Williams & Wilkins Co; 1939.

14. Wechsler D**.** Manual for the Wechsler Adult Intelligence Scale. Oxford, England: Psychological Corp.; 1955.

15. Blackburn HL, Benton AL**.** Revised administration and scoring of the Digit Span Test. Journal of Consulting Psychology. 1957;21(2):139-43.

16. Wechsler D**.** WAIS-IV : Wechsler adult intelligence scale. 4th ed ed. San Antonio, Tex.: Pearson; 2008.

17. Lezak MD, Howieson DB, Bigler ED, Tranel D**.** Neuropsychological assessment, 5th ed. New York, NY, US: Oxford University Press; 2012.

18. Gerton BK, Brown TT, Meyer-Lindenberg A, Kohn P, Holt JL, Olsen RK, et al. Shared and distinct neurophysiological components of the digits forward and backward tasks as revealed by functional neuroimaging. Neuropsychologia. 2004;42(13):1781-7.

**Rating test evaluation**

| **Test name and Assessor:**  Odd Man Out Test  Prof Nicky Edelstyn, n.edelstyn@bathspa.ac.uk | Responses (possible) |
| --- | --- |
| **Version**  Are there several versions (alternate or parallel) of the test? If so, which one has been assessed? | Two experimental versions described in the original paper, the first version presented 3 items and the second presented 4 items. Findings were consistent across both stimulus sets.  The psychometric properties of the paradigm has not been evaluated against a standard (WCST), and studies fail to report which version has been used. |
| **Description of test**  What population the test was originally developed for?  Which domains/components of domains do the test assess?  Are there multiple domains or a predominant domain assessed?  Does it score impaired/unimpaired?  Does it score severity  (subtle/mild/moderate/severe)? | The test was originally developed to examine concept formation and the ability to maintain set in PD (Flowers & Robertson, 1985).  The original paper reports control data for age matched control patients (movement problems, but no neurological/psychiatric history). However, the 2 groups are not matched for IQ.  Flowers KA, Robertson C. The effect of Parkinson’s disease on the ability to maintain a mental set. Journal of Neurology, Neurosurgery & Psychiatry [Internet]. 1985 Jun 1;48(6):517–29. Available from: https://jnnp.bmj.com/content/48/6/517.short |
| **Scaling Metric**: Type of response format? | Continuum (error/accuracy) |
| **Respondent: Patient:** any specific requirements for the target study population (e.g. non-demented, motor, auditory, visual abilities, etc)? | The OMO lacks the complexity of the WCST and allows concept formation to be dissociated from ability to maintain set. |
| **Access:** How can the test be obtained (mail address or website)? | Paradigm needs to be developed by researcher/clinician. |
| Copyrighted or in public domain?  Conditions for use? Fee? | See above |
| **Translations:** Has the test been translated and validated in other languages besides English? | N/A |

| **Test properties**  (Please provide references for all statements or note if this is your judgment) |  |
| --- | --- |
| Which cognitive domain (or component/specific aspect of a cognitive domain) does this test *primarily* measure? | Executive function – concept formation and ability to maintain set. |
| Which aspects of the domain you are assessing are not covered? | N/A |

| ***Use*** |  |
| --- | --- |
| Was it *designed* to measure severity, screen or diagnosis of the domain? | Diagnose |
| Is there a cut-off score? Are there measures of screening/diagnostic performance of the test? (Receiver characteristic curve analyses; sensitivity/specificity; Positive Predictive Value/Negative Predictive Value), True Positive Rate, False Positive Rate etc.) | Control data is presented in the Flowers paper, but application is limited by lack of IQ matching. |
| Is this appropriate for the PD population? If not, why (low sensitivity and/or specificity values)? | Yes: the paradigm provides an opportunity to dissociate concept formation from ability to maintain set, and is not compromised by working memory, reasoning ability, distractibility or perseveration. |
| **Clinimetric/psychometric properties in the target population** |  |
| **Feasibility /Acceptability** |  |
| Length | Two rules, 5 trials per rule, 16 choices per trial. So a guess-estimate 10 mins to administer. |
| Ambiguities in instructions to patient | Clear instructions for administration are provided in Flowers paper. |
| Ambiguities in rating anchors | N/A |
| Appropriateness of questions for population | Yes |
| Applicability across disease cognitive stages | YES: It has been used in a longitudinal study of de novo patients (the DATATOP trial) and mild, mod and severe medicated PD patients (Flowers study). It’s use in the DATATOP trial that largely accounts for it’s ‘popularity’. |
| Applicability across disease motor severity? | Yes, see above |
| Clear instructions to raters | Not available: This is a paradigm to be developed by researchers, rather than a standardized neuropsychological test. See previous responses. |
| Has the test been used by researcher other than developers? | YES |
| Are there instructions for dealing with missing data?  Not applicable? | Not available: This is a paradigm to be developed by researchers, rather than a standardized neuropsychological test. |
| Floor and ceiling effects, skewed score distributions? | NOT ASSESSED |

| **Have the component of dimensionality, variability and dependency structures been analyzed?** | NOT APPLICABLE |
| --- | --- |
|  |  |
| **Reliability: assessed – good; not good. Not assessed** | NOT APPLICABLE |
| **Validity: assessed –good; not good. Not assessed** | NOT APPLICABLE |

| **Responsiveness: assessed –good; not good. Not assessed (reference)** |  |
| --- | --- |
| Demonstrated to be sensitive to change (change over time or due to treatment)* | No evidence |
| Has the minimal clinically important change and minimal clinically relevant incremental difference been assessed? | NO |
| Has this test been assessed or used in patients with other condition than that of interest? | HIV |
| Other |  |
| ***Overall impression*** |  |
| Strengths | 1. The Wisconsin Card Sort Test can be challenging to administer due to the length and complex instructions. 2. The WCST also engages additional cognitive processes such as working memory, which is difficult for patients with compromised memory and   attentional systems, so that failure may reflect some of these mechanisms.   1. The WCST tests abstraction and shifting, these two components are more clearly delineated within a single trial on the OMO test |
| Weaknesses | 1. Lack of a standardized test, with access to instructions, test material etc |

| **Level of Recommendation** |  |
| --- | --- |
| “Recommended” – The test was applied to the target population, AND was studied clinimetrically and found to be valid, reliable and sensitive to change, AND was used in studies by researchers, others than the developers  “Recommended with caveats” – Test’s properties were generally found to be adequate, but some of the measurement properties were not evaluated or not evaluated specifically in a PD cohort."  ‘Suggested’ – The test was applied to the target population, but only one of the other criteria applies  ‘Listed” – The test was applied to the target population, but none of the other criteria applies | Listed” – The test was applied to the target population, but none of the other criteria applies |
| Which type of study is this test suitable for and which type of study is it not suitable for (screening, prevalence, etiological (e.g. case-control or genetic), treatment trial, correlation with biological markers or other scales, e.g. of parkinsonism, clinical practice for diagnosis/ screening). List all. | Suitable for:  Screening  Unsuitable for (please add just categories for which the measure is critically unsuitable):  Prevalence  Differential reasoning (etiological)  Case-control study  Treatment trial |

**Rating test evaluation**

| **Test name: Mental Control (WMS-III)** | Responses (possible) |
| --- | --- |
| **Version**  Are there several versions (alternate or parallel) of the test? If so, which one has been assessed? | Mental Control is a supplemental (optional) subtest of the WMS-III that was already contemplated in previous tests version. The author used it to assess “*cases of organic brain disease that are not too far gone but show defects which would not be made evident by simple rote memory items*” (Wechsler, 1945, pp. 87-88).  Due to rare use in the WMS-III (customer surveys revealed rarely administration), this subtest, such as Information and Orientation ones, were dropped from the WMS-IV (and some items were incorporated into the Brief Cognitive Screening Exam, BCSE).  No parallel forms. |
| **Description of test**   - What population the test was originally developed for? - Which domains/components of domains do the test assess? - Are there multiple domains or a predominant domain assessed? - Does it score impaired/unimpaired? - Does it score severity   (subtle/mild/moderate/severe)? | - Initially included in the WMS, originally intended for assessment of head injuries post-WWII as a portion of the registration section, designed to assess overlearned material. Expanded in the WAIS-III to include more items assessing verbal working memory. - It measures the speeded performance of overlearned material and ability to quickly manipulate commonly known sequences. - Score is based on severity since higher scores reflect better cognitive performance; raw scores can be converted to scaled scores using age-based normative data for interpretation of level of impairment. |
| **Scaling Metric**: Type of response format? (e.g., Likert, Binary, multiple-choice, continuum, reaction time, etc.) | The test is scored on a continuum. |
| **Respondent: Patient:** any specific requirements for the target study population (e.g. non-demented, motor, auditory, visual abilities, etc.)? | Requires hearing sufficient to understand the instructions, and ability to quickly recite sequences, so may be affected by dysarthria, hypophonia, tachyphemia, or slowed speech and reaction time. |
| **Access:** How can the test be obtained (mail address or website)? | Wechsler, D. (1997b). Wechsler Memory Scale–Third Edition. San Antonio, TX: Psychological Corporation.  [www.pearsonassessment.com](http://www.pearsonassessment.com) |
| Copyrighted or in public domain?  Conditions for use? Fee? | Copyright, purchase through website (although the WMS-III forms may no longer be available; Mental Control subtest was not included in the WAIS-IV). |
| **Translations:** Has the test been translated and validated in other languages besides English? | The WMS-III was translated into multiple languages, including (list is not exhaustive): Finnish, French, Spanish, Chinese, Norwegian, etc., with normative data available for some.^1-4^ |

| **Test properties**  (Please provide references for all statements or note if this is your judgment) |  |
| --- | --- |
| Which cognitive domain (or component/specific aspect of a cognitive domain) does this test *primarily* measure? | Attention and **working memory**. |
| Which aspects of the domain you are assessing are not covered? | Mental control does not measure visual attention and visual working memory, switching, response inhibition (cf. Kent et al., 2017)[[13]](#footnote-13). |

| **Use** |  |
| --- | --- |
| Was it *designed* to measure severity, screen or diagnosis of the domain? | Designed to measure severity. |
| Is there a cut-off score? Are there measures of screening/diagnostic performance of the test? (Receiver characteristic curve analyses; sensitivity/specificity; Positive Predictive Value/Negative Predictive Value), True Positive Rate, False Positive Rate etc.) | No established cut-off score. |
| Is this appropriate for the PD population? If not, why (low sensitivity and/or specificity values)? | There is not enough data to support this measure as an appropriate tool in PD research. It is seldom used, and only in smaller studies. One study found that MC demonstrates meaningful change ~4.5 years prior to PDD onset and reported that transition from PD to PD-MCI first begins with mild deficit in working memory.^5^ However, the sample size was small.  Another very small study incorporated it as part of an executive composite that differentiated between PD and controls (but did not evaluate MC alone).^6^  A recent study showed no association between MC (but from the WMS-R) and gait.^7^ |
| **Clinimetric/psychometric properties in the target population** |  |
| **Feasibility /Acceptability** |  |
| Length | 8 items, 3-4 minutes. |
| Ambiguities in instructions to patient | No. |
| Ambiguities in rating anchors | No. |
| Appropriateness of questions for population | Yes. |
| Applicability across disease cognitive stages | MCI/Dementia |
| Applicability across disease motor severity? | Appropriate for all stages of motor disease as it does not require intact motor function for completion. May be impacted by slowing as the disease progresses. May be impacted if dysarthria is severe or if the participant has an excessively soft voice.  H&Y score: 1, Yes;  H&Y score: 2 or 3, Yes (Parrao et al., 2011)[[14]](#footnote-14);  (H&Y 1-3, Drag et al., 2009)[[15]](#footnote-15);  H&Y score: ≥4, Yes. |
| Clear instructions to raters | Yes, well described in test manual. |
| Has the test been used by researcher other than developers?  By other groups  Not applicable? | Yes, but minimally. It is an optional subtest and so often not included in research that uses total WMS-III or composite scores. |
| Are there instructions for dealing with missing data?  Not applicable? | If unable to complete, score is 0. |
| Floor and ceiling effects, skewed score distributions? | Likely ceiling effects^8^. |

| **Have the component of dimensionality, variability and dependency structures been analyzed?**  (Exploratory and confirmatory factor analysis (EFA and CFA)? | As a supplemental test, this subtest was not included in primary studies validating the factor structure of the WMS-III (Manual; Tulsky 2003),^9,10^ or in other WMS-III factor analytic studies^11,12^. |
| --- | --- |
|  |  |
| **Reliability: assessed – good; not good. Not assessed**  Are alternative versions developed?  If so, were they validated with reliability measures? | Assessed-good, reported in the WAIS-III/WMS-III Technical and Interpretive Manual. |
| Internal consistency (A rule of thumb)  0.9 ≤ Cronbach’s alpha (α) Excellent  0.8 ≤ α < 0.9 Good  0.7 ≤ α < 0.8 Acceptable  0.6 ≤ α < 0.7 Questionable  0.5 ≤ α < 0.6 Poor  α < 0.5 Unacceptable  (Streiner, 2003 Journal of Personality Assessment, 80:1, 99-103, DOI: 10.1207/S15327752JPA8001_18) | 0.89 for ages 55-89. |
| “Corrected item-to-total correlation” | - |
| Test/retest | Stability coefficient = 0.80. |
| Inter-rater | From the WAIS-III/WMS-III Technical and Interpretive Manual:^9^ “Because the scoring criteria for most of the WAIS-III and WMS-III subtests are simple and objective, interscorer agreement is very high, averaging in the high .90s.”  It is not clear from the manual whether interscorer agreement was assessed for the optional measures on the WMS-III; no tables were provided. |
| Intra-rater | None reported in test manual. |
| Other (e.g. Standard Error of Measurement) | Not provided for supplemental tests. |
| **Validity: assessed –good; not good. Not assessed (reference)** | Not assessed (or reported) in the WAIS-III/WMS-III Technical and Interpretive Manual. Validity studies incorporated only the primary subtests/indices. |
| Face and Content validity (does test appears to be measuring what it is intended to measure and do test’s items comprehensively represent the domain of interest?) | N/A |
| Criterion validity (compared to gold-standard) or other criteria for  diagnosis) (e.g. other comparable tests or lesion or MRI research) | N/A |
| Construct validity (correlations with other convergent tests and divergent tests; known-groups comparisons) | Findings from studies suggest that the WMS-III measures similar construct to those measured in the WMS-VI despite major changes made to content and structure (Wechsler, 2008; pg. 66)[[16]](#footnote-16) |
| Reference group used to develop this test:   1. If available, please define the characteristic of the norm group adopted (demographic variables used for stratification: age, education, sex, etc). 2. What kind of standardized score generated (T score, Z score, derived IQ score, percentile ranks, etc)? 3. If translated/validated in other languages, are reference norms created? If so, please specify demographic variables used for stratification (age, education, gender etc). | Group characteristics:  WAIS-III/WMS-III normative sample^9^ (1,023 based on 1995 Census; weighting procedures used to extend sample to 1,250) was used (with stratification by age, education level, sex, race/ethnicity, and US geographic region based on 1995 US Bureau of the Census data). Age norms are capped at 89 (with 100 participants in each age group except for the two oldest age groups (75 each). Except for the oldest age groups (65 +), equal numbers of males/females were included. For the older age groups, there were more women, consistent with census data.  Age-corrected standard scores (mean= 10, sd=3) are generated from raw scores.  Translated and validated in multiple countries and languages (see references listed above). |

| **Responsiveness: assessed –good; not good. Not assessed (reference)** |  |
| --- | --- |
| Demonstrated to be sensitive to change (change over time or due to treatment)*  * [determined by the strength of the relationship between change in the test scores and the criterion or anchor scores] | Mental Control showed meaningful prodromal changepoint 4½ years prior to PDD in a small sample^5^. |
| Has the minimal clinically important change and minimal clinically relevant incremental difference been assessed?  (besides a statistically significant improvement) | No. |
| Has this test been assessed or used in patients with other condition than that of interest? | Yes. It has been used in some studies of AD, and vascular dementia.^13,14,15^. The WMS-III version is not commonly used however. |
| Other | / |
| **Overall impression** |  |
| Strengths | Reported good reliability (although not assessed as carefully as the primary WMS-III subtests).  Easy and quick to administer.  Translated into multiple languages. |
| Weaknesses | May be difficult to purchase or obtain permission to use due to being out of print and lack of inclusion in the current WMS-IV. Not included in the WMS-III validity studies. Rarely used in PD research (only small studies to date), so validity and sensitivity to change in this population is largely unknown.  MC has not been used in large-scale PD research studies. |

| **Level of Recommendation** |  |
| --- | --- |
| “Recommended” – The test was applied to the target population, AND was studied clinimetrically and found to be valid, reliable and sensitive to change, AND was used in studies by researchers, others than the developers  “Recommended with caveats” – Test’s properties were generally found to be adequate, but some of the measurement properties were not evaluated or not evaluated specifically in a PD cohort."  ‘Suggested’ – The test was applied to the target population, but only one of the other criteria applies  ‘Listed” – The test was applied to the target population, but none of the other criteria applies | Listed |
| Which type of study is this test suitable for and which type of study is it not suitable for (screening, prevalence, etiological (e.g. case-control or genetic), treatment trial, correlation with biological markers or other scales, e.g. of parkinsonism, clinical practice for diagnosis/ screening). List all. | Screening (the Brief Cognitive Status Exam, that is an optional tool in the WMS-IV was derived, in part, from the Information and Orientation and Mental Control subtests of the WMS-III). |

**REFERENCES**

1. Wechsler, D. Pere**ñ**a J. WMS-III: escala de memoria de Wechsler-III. Madrid: TEA Ediciones; 2004.

2. Bosnes O, Troland K, Torsheim T. A Confirmatory Factor Analytic Study of the Wechsler Memory Scale-III in an Elderly Norwegian Sample. Arch Clin Neuropsychol 2016; 31:12-17.

3. Hua MS, Chang BS, Lin KN, et al. Wechsler Memory Scale 3rd edition Manual (Chinese). Taipei: Behavioral Science Corporation; 2005.

4. Tulsky DS, Saklofske DH, Chelune GJ, et al. Clinical Interpretation of the WAIS-III and WMS-III. New York: Academic Press; 2003.

5. Johnson DK, Langford Z, Garnier-Villarreal M, et al. Onset of Mild Cognitive Impairment in Parkinson Disease. Alzheimer Dis Assoc Disord 2016; 30:127-133.

6. Drag LL, Bieliauskas LA, Kaszniak AW, et al. Source memory and frontal functioning in Parkinson's disease. J Int Neuropsychol Soc 2009; 15:399-406.

7. Sousa NMF, Macedo RC. Relationship between cognitive performance and mobility in patients with Parkinson’s disease: A cross-sectional study. Dement Neuropsychol 2019; 13:403-409.

8. Flanagan DP, McGrew KS, Ortiz SO. The Wechsler Intelligence Scales and Gf-Gc theory: A contemporary approach to interpretation. Boston, MA: Allyn & Bacon; 2000.

9. Wechsler D. WAIS-III/WMS-III Technical Manual: San Antonio, TX: The Psychological Corporation; 2002.

10. Tulsky DS, Price LR. The joint WAIS-III and WMS-III factor structure: development and cross-validation of a six-factor model of cognitive functioning. Psychol Assess 2003; 15:149-162.

11. Wilde NJ, Strauss E, Chelune GJ, et al. Confirmatory factor analysis of the WMS-III in patients with temporal lobe epilepsy. Psychol Assess 2003; 15:56-63.

12. Price L, Tulsky D, Millis S, et al. Redefining the factor structure of the Wechsler Memory Scale-III: confirmatory factor analysis with cross-validation. J Clin Exp Neuropsychol 2002; 24:574-585.

13. Giovannetti T, Lamar M, Cloud BS, et al. Different underlying mechanisms for deficits in concept formation in dementia. Arch Clin Neuropsychol 2001; 16:547-560.

14. Wagner MT, Wymer JH, Carlozzi NE, et al. Preliminary examination of progression of Alzheimer's disease in a rural Southern African American cohort. Arch Clin Neuropsychol 2007; 22:405-414.

15. Jefferson, A.L., Cosentino, S.A., Ball, et al. (2002). Errors produced on the mini-mental state examination and neuropsychological test performance in Alzheimer's disease, ischemic vascular dementia, and Parkinson's disease. J Neuropsychiatry Clin Neurosci 2002; 14(3): 311-320.

**Executive domain:** Recommended with caveats, Suggested and Listed neuropsychological tests including their psychometric properties.

**Rating test evaluation**

| **Test name:** | Tower of London (Tower of London-Drexel) |
| --- | --- |
| **Version**  Are there several versions (alternate or parallel) of the test? If so, which one has been assessed? | The original version by Shallice, 1982^1^ comprised 12 problems ranging from 2 to 5 moves.  The revised version^2^ comprised 30 items.  Other versions are:  - TOLDX^3-5^,   - five-disk Tower of London^6^   ,   - the 4-rod Tower of   London^7^,   - the TOL±R^2,8^   ;  Computerised versions:  TOL-Freiburg version (TOL-F): 24 items using four- to six-move TOL problems only^9^  Cambridge Neuropsychological Test Automated Battery (CANTAB) version of the TOL^10^;  The original, manual (non-computerised) version of the TOL is among the  most widely used and it has therefore been assessed as primary version. |
| **Description of test**   - What population the test was originally developed for? - Which domains/components of domains do the test assess? - Are there multiple domains or a predominant domain assessed? - Does it score impaired/unimpaired? - Does it score severity   (subtle/mild/moderate/severe)? | The goal of the Tower of London is to match the pattern and color of balls to a pictured arrangement. In doing this, no more than two balls may be placed on the middle peg, and no more than one ball may be placed on the smallest peg.  The target array is presented as a colored drawing and the subject's task is to solve the problem with the minimum number of possible moves. Twelve levels of cognitive difficulty were tested. It reflects the overall performance of the task including planning of moves and task execution.   - To assess executive dysfunctions, such as following brain injuries. - It assesses executive functions and in particular planning, inhibition, and working memory. - Executive functions are predominately assessed - Does not provide impairment - Does not score severity |
| **Scaling Metric**: Type of response format? (e.g., Likert, Binary, multiple-choice, continuum, reaction time, etc.) | There are two scoring systems for the original TOL:  System 1 (S1), planning effectiveness score, expresses the **number of first time correctly solved** (without an error) tasks out of a total of 12.  System 2 (S2), the **time score**, is obtained as the sum of points assigned based on the time needed to successfully solve a task^1^. Other scoring systems have been proposed by Anderson and colleagues^11^ and Krikorian and colleagues^12^. |
| **Respondent: Patient:** any specific requirements for the target study population (e.g. non-demented, motor, auditory, visual abilities, etc)? | Not suitable for patients with severe apraxia or paraplegia or severe motor deficits (i.e., dyskinesias) ; people with color blindness may have difficulties (depending on the severity of the problem) |
| **Access:** How can the test be obtained (mail address or website)? | The original 12 problems are reported in Shallice^1^as well as the instructions for the board |
| Copyrighted or in public domain?  Conditions for use? Fee? | The original version is free, but the board with three vertical pegs and the three balls should be build according to the standardize measures  TOL_F can be purchased @ <https://marketplace.schuhfried.com/en/TOL>  TOL DX material and manual can be found @ <https://www.cognitivecentre.com/assessment/tower-london-dx-2nd-edition-tol-dx-2nd-edition/>  CANTAB battery is available @ <https://www.cambridgecognition.com/cantab/> |
| Translations: Has the test been translated and validated in other languages besides English? | Y  Original version:  Czeck^13^  Italian ^14,15^  TOL-F:  German^16^.  TOLDX:  Spanish^17 18^ |

| **Test properties**  (Please provide references for all statements or note if this is your judgment) |  |
| --- | --- |
| Which cognitive domain (or component/specific aspect of a cognitive domain) does this test *primarily* measure? | **Executive function:** planning and inhibition ^19, 20^  Attention and working memory: working memory is also involved ^21,22^  Visuospatial functions are involved |
| Which aspects of the domain you are assessing are not covered? | They are almost all covered |

| **Use** |  |
| --- | --- |
| Was it *designed* to measure severity, screen or diagnosis of the domain? | TOL was first intended for experimental  purposes as a test of planning ability in  patients with frontal lobe lesion^1^ |
| Is there a cut-off score? Are there measures of screening/diagnostic performance of the test? (Receiver characteristic curve analyses; sensitivity/specificity; Positive Predictive Value/Negative Predictive Value), True Positive Rate, False Positive Rate etc.) | Cut-off score in relation to the diagnostic unit (MCI/dementia)  The study by Michalec and coll.,^23^demonstrate that TOL scores (all the four correction systems mentioned above) significantly discriminate PD-MCI from healthy subjects. They provide normative data but not a cut off for MCI |
| Is this appropriate for the PD population? If not, why (low sensitivity and/or specificity values)? | It demonstrated good discriminative validity in PD the original version^23^ as well as different versions of ToL ^24,25^ |
| **Clinimetric/psychometric properties in the target population** |  |
| **Feasibility /Acceptability** |  |
| Length | 12 items  15-20 minutes for administration |
| Ambiguities in instructions to patient | N |
| Ambiguities in rating anchors | There are different methods, but each method is well explained with no ambiguities |
| Appropriateness of questions for population | Y |
| Applicability across disease cognitive stages | MCI/Dementia  Patients with severe dementia may find it difficult to complete the task and they could become really frustrated |
| Applicability across disease motor severity? | Patients with important dyskinesia may not be able to fit the beads in the pegs.  H&Y score: ≥4 patients would not be able to complete the task because of the manual ability it requires |
| Clear instructions to raters | Y |
| Has the test been used by researcher other than developers?  By other groups  Not applicable? | Y  The test has been widely used by several international groups both for clinical and research purposes |
| Are there instructions for dealing with missing data?  Not applicable? | N |
| Floor and ceiling effects, skewed score distributions? | In an Italian validation, raw data are reported as skewed due to ceiling effect but it was possible to apply the logit transformation and rescale the data^15^. |

| **Have the component of dimensionality, variability and dependency structures been analyzed?**  (Exploratory and confirmatory factor analysis (EFA and CFA)? | The original version has poor psychometric properties Cronbach alpha= .25; split-half reliability = .19 ^7,26^  the TOL-F has been shown to possess an adequate absolute and relative test–retest reliability ^27^as well as a sufficiently high internal consistency ^28^ |
| --- | --- |
|  |  |
| **Reliability: assessed – good; not good. Not assessed**  Are alternative versions developed?  x  If so, were they validated with reliability measures? | Y there are different version of the test (in terms of scoring and administration)  The TOLDX has a second version |
| Internal consistency (A rule of thumb)  0.9 ≤ Cronbach’s alpha (α) Excellent  0.8 ≤ α < 0.9 Good  0.7 ≤ α < 0.8 Acceptable  0.6 ≤ α < 0.7 Questionable  0.5 ≤ α < 0.6 Poor  α < 0.5 Unacceptable  (Streiner, 2003 Journal of Personality Assessment, 80:1, 99-103, DOI: 10.1207/S15327752JPA8001_18) | Original version: Unaccettable ^7,26^  TOL-F: questionable ^29^ |
| “Corrected item-to-total correlation” | Y/N, if Y, specify how large |
| Test/retest | Y  TOL-F:  accuracy at time 1 and 2 significantly correlated at r = .739 (95% confidence interval [CI] [.498, .873], p = .001; coefficient of variation at 9.55% ^27^ .  Initial thinking time (*r* _ .405 (95% CI [.030, .680], *p* _ .036), with an ICC(3,1) of *r* _ .390 (95% CI [.019, .667], *p* _ .020) and an ICC(2,1) of *r* _ .274 (95% CI [–.072, .578], *p* _ .020; CV of 32.90%) and Movement execution time (*r* _ .519, 95% CI [.174, .751], *p* _ .006), ICC(3,1; *r* _ .475; 95% CI [.124,.721], *p* _ .005), and ICC(2,1; *r* _ .348; 95% CI [–.045, .648], *p* _.005 had low reliability.  TOLDX2nd edition: high test–retest reliability r = .80 for total moves; but r = .42 for total correct^5^ |
| Inter-rater | N |
| Intra-rater | N |
| Other (e.g. Standard Error of Measurement) | N |
| **Validity: assessed –good; not good. Not assessed (reference)** |  |
| Face and Content validity (does test appears to be measuring what it is intended to measure and do test’s items comprehensively represent the domain of interest?) | TOL-F: the analysis suggests a one-factor solution: the item responses are mainly determined by one underlying latent trait, namely planning ability. ^30^ |
| Criterion validity (compared to gold-standard) or other criteria for  diagnosis) (e.g. other comparable tests or lesion or MRI research) | Y: neural correlates have been extensively studied^31^ |
| Construct validity (correlations with other convergent tests and divergent tests; known-groups comparisons) | Add data regarding:  TOL DX ^5^.  Convergent validity significant correlations between the TOLDX scores and the executive measures: total move score, time violation score, and stimulus-bound score each correlated significantly with the TMT-B time score (*pr* = .50, *p* < .005 and *pr* = .64, *p* <.05, respectively; *pr* = .47, *p* < .005) and CDT-A/O score (*pr* = .62, *p* < .005 and *pr* = .52, *p* <.05,; *pr* = .59, *p* < .005 respectively) whereas the TOLDX rule violation and stimulus-bound score associated with the TMT-B error score. (*pr* = .66, *p* < .001 and *pr* = .47, *p* = .004, respectively).  Divergent validity no association with memory or intelligence measures.  Group-comparisons Y |
| Reference group used to develop this test:   46) If available, please define the characteristic of the norm group adopted (demographic variables used for stratification: age, education, sex, etc). 47) What kind of standardized score generated (T score, Z score, derived IQ score, percentile ranks, etc)? 48) If translated/validated in other languages, are reference norms created? If so, please specify demographic variables used for stratification (age, education, gender etc). | Group characteristics:  Children^11^  257 Australian Women  Age range: 56 to 67 years  Low(<12 years) vs high (>12 years) education level  Provides means and DS for total score, the number of correct and failed attempts, and the total solution time ^32^  Add the language of translation and group characteristics:  Original version: Italian  216 healthy participants (88 men and 128 women) age between 18 and 89 years (Mean age = 45.7 years, SD = 18.7), level of education between 5 and 24 years (Mean education = 13.6 years,  SD = 4)  Means, Standard Deviations and Observed Ranges for Time and Accuracy Scores are reported; Equivalent scores and percentiles as a function of demographics for Time and Accuracy  Country: Italy^15^  .  TOL-F: German  7703 healthy subjects (51.4% man)  Mean Age: 59.5yrs sd=10.6yrs  Mean education: 12.9 sd=2.01  Provides Percentile ranks of TOL performance, separately for low and high education and age groups and separately for males and women.  Country: Germany^16^      TOL DX: Spanish  179 cognitively normal participants (65 males)  from 18 to 49 years old  education from 8 to >15 years  Country: Spain^18^ |

| **Responsiveness: assessed –good; not good. Not assessed (reference)** |  |
| --- | --- |
| Demonstrated to be sensitive to change (change over time or due to treatment)*  * [determined by the strength of the relationship between change in the test scores and the criterion or anchor scores] | Y  Patients with PD ON and OFF medications: patients performed significantly better on L-dopa for both the accuracy and latency variables. As expected, initial thinking time was longer as a function of problem difficulty^33^.  Yoga training for a month reduced the planning and execution time in simple (2-moves) as well as complex tasks (4, 5-moves) and facilitated reaching  the target with a smaller number of moves in a complex task (4-moves)^34^.  The number of correct decisions in The Tower of London task was significantly affected by marijuana (THC)^35^ |
| Has the minimal clinically important change and minimal clinically relevant incremental difference been assessed?  (besides a statistically significant improvement) | Minimal clinically important change assessed Y/N?  N |
| Has this test been assessed or used in patients with other condition than that of interest? | Alzheimer’s disease and related dementias^36-39^ ,attention-deficit/hyperactivity disorder (ADHD;  e.g., ^,40,41^, autism^42,43^, obsessive– compulsive disorder^44-46^ ,and schizophrenia ^47-50^ |
| Other |  |
| **Overall impression** |  |
| Strengths | The test measures the construct it was developed to measure.  It is sensitive to planning deficit in PD patients. |
| Weaknesses | The most used in clinical practice is the original version which shows inconsistency in psychometric studies on the reliability and construct validity.  In elderly, performing the TOL loads all components of working memory, whereas for the younger participants the TOL more specifically loads executive functioning^51^ |

| **Level of Recommendation** |  |
| --- | --- |
| “Recommended” – The test was applied to the target population, AND was studied clinimetrically and found to be valid, reliable and sensitive to change, AND was used in studies by researchers, others than the developers  “Recommended with caveats” – Test’s properties were generally found to be adequate, but some of the measurement properties were not evaluated or not evaluated specifically in a PD cohort."  ‘Suggested’ – The test was applied to the target population, but only one of the other criteria applies  ‘Listed” – The test was applied to the target population, but none of the other criteria applies | Recommended with caveats |
| Which type of study is this test suitable for and which type of study is it not suitable for (screening, prevalence, etiological (e.g. case-control or genetic), treatment trial, correlation with biological markers or other scales, e.g. of parkinsonism, clinical practice for diagnosis/ screening). List all. | Suitable for:  Screening  Case-control study  Treatment trial  Biomarker study |

**REFERENCES**

1. Shallice T. Specific impairments of planning. Philos Trans R Soc Lond B Biol Sci. 1982;298(1089):199-209.
2. Schnirman GM, Welsh MC, Retzlaff PD. Development of the Tower of London—Revised. Assessment. 1998;5(4):355-360.
3. Culbertson WC, Zillmer EA. The Tower of LondonDX: A standardized approach to assessing executive functioning in children. Arch Clin Neuropsychol. 1998;13(3):285-301.
4. Culbertson WC, Zillmer EA. The construct validity of the Tower of LondonDX as a measure of the executive functioning of ADHD children. Assessment. 1998 Sep;5(3):215 226. doi:10.1177/107319119800500302. PMID: 9728029.
5. Culbertson W, Zillmer EA. Tower of London Drexel University – 2nd edition (TOL-DX 2nd edition). North Tonawanda: Multi-Health Systems; 2005.
6. Ward G, Allport A. Planning and problem-solving using the 5-disc Tower of London task. Q J Exp Psychol A. 1997;50A(1):49-78.
7. Kafer KL, Hunter M. On testing the validity of planning/problem-solving tasks in a normal population. J Int Neuropsychol Soc. 1997;3(2):108-119.
8. Welsh MC, Satterlee-Cartmell T, Stine M. Towers of Hanoi and London: contribution of working memory and inhibition to performance. Brain Cogn. 1999;41(2):231-242. doi:10.1006/brcg.1999.1123. PMID: 10590821.
9. Kaller CP, Rahm B, Köstering L, Unterrainer JM. Reviewing the impact of problem structure on planning: A software tool for analyzing tower tasks. Behav Brain Res. 2011;216:1-8. doi:10.1016/j.bbr.2010.07.029.
10. Owen AM, Downes JJ, Sahakian BJ, Polkey CE, Robbins TW. Planning and spatial working memory following frontal lobe lesions in man. Neuropsychologia. 1990;28(10):1021-1034. doi:10.1016/0028-3932(90)90137-d. PMID: 2267054.
11. Anderson P, Anderson V, Lajoie G. The Tower of London test: Validation and standardization for pediatric populations. Clin Neuropsychol. 1996;10:54-65. doi:10.1080/13854049608406663.
12. Krikorian R, Bartok J, Gay N. Tower-of-London procedure: A standard method and developmental data. J Clin Exp Neuropsychol. 1994;16:840-850. doi:10.1080/01688639408402697.
13. Michalec J, Bezdicek O, Nikolai T, et al. Standardization of the Czech Version of the Tower of London Test – Administration, Scoring, Validity. Česka a Slovenská Neurologie a Neurochirurgie. 2014;77/110(5):596-601. doi:10.14735/amcsnn2014596.
14. Boccia M, Marin D, D’Antuono G, et al. The Tower of London (ToL) in Italy: Standardization of the ToL test in an Italian population. Neurol Sci. 2017;38(7):1263-1270. doi:10.1007/s10072-017-2957-y. PMID: 28432516.
15. Bruni F, Toraldo A, Scarpina F. Italian normative data for the original version of the Tower of London test: A bivariate analysis on speed and accuracy scores. Assessment. 2020;29(2):209-224. doi:10.1177/1073191120961834.
16. Unterrainer JM, Rahm B, Kaller CP, et al. Assessing planning ability across the adult life span in a large population-representative sample: Reliability estimates and normative data for the Tower of London (TOL-F) task. J Int Neuropsychol Soc. 2019;25(5):520-529. doi:10.1017/S1355617718001248. PMID: 30696511; PMCID: PMC6669988.
17. Peña-Casanova J, Quiñones-Ubeda S, Gramunt-Fombuena N, et al. NEURONORMA Study Team. Spanish multicenter normative studies (NEURONORMA Project): Norms for the Stroop color-word interference test and the Tower of London-Drexel. Arch Clin Neuropsychol. 2009;24(4):413-429. doi:10.1093/arclin/acp043. PMID: 19661108.
18. Rognoni T, Casals-Coll M, Sánchez-Benavides G, et al. Estudios normativos españoles en población adulta joven (proyecto NEURO-NORMA jóvenes): Normas para las pruebas Stroop Color—Word Interference Test y Tower of London-Drexel University. Neurología. 2013;28:73-80.
19. Levin HS, Song J, Scheibel RS, et al. Concept formation and problem-solving following closed head injury in children. J Int Neuropsychol Soc. 1997;3(6):598-607. PMID: 9448373.
20. Luciana M, Nelson CA. The functional emergence of prefrontally-guided working memory systems in four- to eight-year-old children. Neuropsychologia. 1998;36(3):273-293. doi:10.1016/s0028-3932(97)00109-7. PMID: 9622192.
21. Phillips LH, Wynn V, Gilhooly KJ, Della Sala S, Logie RH. The role of memory in the Tower of London task. Memory. 1999;7(2):209-231. doi:10.1080/741944066. PMID: 10645380.
22. Michalec J, Bezdicek O, Nikolai T, et al. A comparative study of Tower of London scoring systems and normative data. Arch Clin Neuropsychol. 2017;32(3):328-338. doi:10.1093/arclin/acw111. PMID: 28096070.
23. Köstering L, McKinlay A, Stahl C, Kaller CP. Differential patterns of planning impairments in Parkinson's disease and sub-clinical signs of dementia? A latent-class model-based approach. PLoS One. 2012;7(6):e38855. doi:10.1371/journal.pone.0038855. PMID: 22715417; PMCID: PMC3371002.
24. Köstering L, Schmidt CS, Egger K, et al. Assessment of planning performance in clinical samples: Reliability and validity of the Tower of London task (TOL-F). Neuropsychologia. 2015;75:646-655. doi:10.1016/j.neuropsychologia.2015.07.017. PMID: 26197091.
25. Humes GE, Welsh MC, Retzlaff P, Cookson N. Towers of Hanoi and London: Reliability and validity of two executive function tasks. Assessment. 1997;4(3):249-257. doi:10.1177/107319119700400305. PMID: 26613774.
26. Köstering L, Nitschke K, Schumacher FK, Weiller C, Kaller CP. Test-retest reliability of the Tower of London planning task (TOL-F). Psychol Assess. 2015;27(3):925-931. doi:10.1037/pas0000097. PMID: 25822835.
27. Kaller CP, Debelak R, Köstering L, et al. Assessing planning ability across the adult life span: Population-representative and age-adjusted reliability estimates for the Tower of London (TOL-F). Arch Clin Neuropsychol. 2016;31(2):148-164. doi:10.1093/arclin/acv088. PMID: 26715472.
28. Kaller CP, Unterrainer JM, Stahl C. Assessing planning ability with the Tower of London task: Psychometric properties of a structurally balanced problem set. Psychol Assess. 2012;24(1):46-53. doi:10.1037/a0025174. PMID: 21859218.
29. Debelak R, Egle J, Köstering L, Kaller CP. Assessment of planning ability: Psychometric analyses on the unidimensionality and construct validity of the Tower of London task (TOL-F). Neuropsychology. 2016;30(3):346-360. doi:10.1037/neu0000238. PMID: 26523519.
30. Ruocco AC, Rodrigo AH, Lam J, Di Domenico SI, Graves B, Ayaz H. A problem-solving task specialized for functional neuroimaging: Validation of the Scarborough adaptation of the Tower of London (S-TOL) using near-infrared spectroscopy. Front Hum Neurosci. 2014;8:185. doi:10.3389/fnhum.2014.00185. PMID: 24734017; PMCID: PMC3975118.
31. Lee YC, Anderson JF, Dennerstein L, Henderson VW, Szoeke C. Normative data for the Tower of London performance in Australian midlife women. Aust Psychol. 2013;48(6):402-407. doi:10.1111/ap.12033.
32. Lange KW, Robbins TW, Marsden CD, James M, Owen AM, Paul GM. L-dopa withdrawal in Parkinson's disease selectively impairs cognitive performance in tests sensitive to frontal lobe dysfunction. Psychopharmacology (Berl). 1992;107(2-3):394-404. doi:10.1007/BF02245167. PMID: 1615139.
33. Manjunath NK, Telles S. Improved performance in the Tower of London test following yoga. Indian J Physiol Pharmacol. 2001;45(3):351-354. PMID: 11881575.
34. Ramaekers JG, Kauert G, van Ruitenbeek P, Theunissen EL, Schneider E, Moeller MR. High-potency marijuana impairs executive function and inhibitory motor control. Neuropsychopharmacology. 2006;31(10):2296-2303. doi:10.1038/sj.npp.1301068. PMID: 16572123.
35. Carlin D, Bonerba J, Phipps M, Alexander G, Shapiro M, Grafman J. Planning impairments in frontal lobe dementia and frontal lobe lesion patients. Neuropsychologia. 2000;38(5):655-665. doi:10.1016/S0028-3932(99)00102-5.
36. De Paula VJR, Guimarães FM, Diniz BS, Forlenza OV. Neurobiological pathways to Alzheimer's disease: Amyloid-beta, TAU protein or both? Dement Neuropsychol. 2012;6(2):85-93. doi:10.1590/S1980-57642012DN06020004.
37. Franceschi M, Caffarra P, Savarè R, Cerutti R, Grossi E, De Vreese LP. Tower of London test: A comparison between conventional and Rasch-based scoring systems in Alzheimer's disease. Int J Geriatr Psychiatry. 2007;22(5):465-470. doi:10.1002/gps.1794.
38. Marchegiani F, Giannelli S, Odetti P. Cognitive impairment and Alzheimer's disease: A review. Arch Gerontol Geriatr. 2010;50(1):1-8. doi:10.1016/j.archger.2009.02.001.
39. Tucha L, Tucha O, Sontag TA, Stasik D, Laufkötter R, Lange KW. Differential effects of methylphenidate on problem solving in adults with ADHD. J Atten Disord. 2011;15(2):161-173. doi:10.1177/1087054709356391.
40. Perrin S, Sutter C, Wade K. Executive functioning in children with ADHD: A meta-analytic review. J Clin Child Adolesc Psychol. 2014;43(5):706-727. doi:10.1080/15374416.2013.850699.
41. Hughes C, Russell J, Robbins TW. Evidence for executive dysfunction in autism. Neuropsychologia. 1994;32(4):477-492. doi:10.1016/0028-3932(94)90092-2.
42. Robinson S, Goddard L, Dritschel B, Wisley M, Howlin P. Executive functions in children with autism spectrum disorders. Brain Cogn. 2009;71(3):362-368. doi:10.1016/j.bandc.2009.06.007.
43. Krishna R, Udupa S, George CM, Kumar KJ, Viswanath B. Neuropsychological performance in OCD: A study in medication-naïve patients. Prog Neuropsychopharmacol Biol Psychiatry. 2011;35(8):1969-1976. doi:10.1016/j.pnpbp.2011.07.010.
44. van den Heuvel OA, Veltman DJ, Groenewegen HJ, et al. Disorder-specific neuroanatomical correlates of attentional bias in obsessive-compulsive disorder, panic disorder, and hypochondriasis. Arch Gen Psychiatry. 2005;62(8):922-933. doi:10.1001/archpsyc.62.8.92.
45. Veale DM, Sahakian BJ, Owen AM, Marks IM. Specific cognitive deficits in tests sensitive to frontal lobe dysfunction in obsessive-compulsive disorder. Psychol Med. 1996;26(6):1261-1269. doi:10.1017/S0033291700035984.
46. Greenwood KE, Wykes T, Sigmundsson T, Landau S, Morris RG. Executive functioning in schizophrenia: A meta-analytic review. Psychol Med. 2011;41(6):1249-1261. doi:10.1017/S0033291710001847.
47. Lam M, Raine A, Lee TM. Executive functioning and its relationship to schizophrenia. Schizophr Res. 2014;157(1-3):1-7. doi:10.1016/j.schres.2014.05.009.
48. Pantelis C, Barnes TRE, Nelson HE, et al. Frontal-striatal cognitive deficits in patients with chronic schizophrenia. Brain. 1997;120(10):1823-1843. doi:10.1093/brain/120.10.1823.
49. Zhu X, Wang X, Xiao J,et al. Evidence of a dissociation pattern in resting-state default mode network connectivity in first-episode, treatment-naive major depression patients. Biol Psychiatry. 2010;71(7):611-617. doi:10.1016/j.biopsych.2011.10.035.
50. Phillips LH, Gilhooly K, Logie R, Sala SD, Wynn V. Age, working memory, and the Tower of London task. Eur J Cogn Psychol. 2003;15:291-312. doi:10.1080/09541440244000148.

**Rating test evaluation**

| **Test name:** | **10 POINTS CLOCK DRAWING TEST** |
| --- | --- |
| Version  Are there several versions (alternate or parallel) of the test? If so, which one has been assessed? | There are several versions of the 10 CDT.  In most of them subject is asked to draw the face of a clock (a pre-drawn circle is provided in some versions) with all the numbers and set a time (most frequently used hour setting:11:10; Sunderland’s version 2:45).^1–5^  Literature supports the correlation of different clock scoring systems  with each other and with scores on structured mental status examinations.^6–8^ |
| **Description of test**   - What population the test was originally developed for? - Which domains/components of domains do the test assess? - Are there multiple domains or a predominant domain assessed? - Does it score impaired/unimpaired? - Does it score severity   (subtle/mild/moderate/severe)? | - Initially utilized as a “parietal lobe” test to measure visuospatial neglect and inattention.^9^ - The test assesses multiple domains: selective and sustained attention, auditory comprehension, verbal working memory, numerical knowledge, visual memory and reconstruction, visuospatial abilities, on-demand motor execution (praxis) and executive function.^10–14^ - Most versions categorize as impaired/unimpaired based on a cut off. - Does not provide severity score |
| **Scaling Metric**: Type of response format? (e.g., Likert, Binary, multiple-choice, continuum, reaction time, etc.) | Specify the scale of measurement:  The drawing is scored on a 10 points scale.  Some versions have a qualitative evaluation in addition to the quantitative one.^3,15^ |
| **Respondent: Patient:** any specific requirements for the target study population (e.g. non-demented, motor, auditory, visual abilities, etc)? | Not suitable for patients with severe apraxia, paraplegia or severe movement disorders (dyskinesias); spared visual abilities are needed. |
| **Access:** How can the test be obtained (mail address or website)? | There is no need for material. All the instructions are in the original paper, comprised circle dimensions for the versions where the circle is provided by the examinator |
| Copyrighted or in public domain?  Conditions for use? Fee? | Public domain |
| **Translations:** Has the test been translated and validated in other languages besides English? | Y  English^16^  Italian^17,18^  French^19^  Chinese^20^  Persian^21^  Turkish^22^ |

| **Test properties**  (Please provide references for all statements or note if this is your judgment) |  |
| --- | --- |
| Which cognitive domain (or component/specific aspect of a cognitive domain) does this test *primarily* measure? | Executive functions involved are planning, organization and parallel processing.  Many other cognitive domains are involved:  Attention and working memory  Memory (semantic representation)  Visuospatial function  Praxis abilities |
| Which aspects of the domain you are assessing are not covered? | Abstraction, Response inhibition. |

| **Use** |  |
| --- | --- |
| Was it *designed* to measure severity, screen or diagnosis of the domain? | It was a screening test for visuospatial abilities in Alzheimer dementia.^1^ |
| Is there a cut-off score? Are there measures of screening/diagnostic performance of the test? (Receiver characteristic curve analyses; sensitivity/specificity; Positive Predictive Value/Negative Predictive Value), True Positive Rate, False Positive Rate etc.) | Cut-off score in relation to the diagnostic unit (MCI/dementia)  Acceptable sensitivity and specificity for moderate to severe cognitive impairment (77% and 87%,respectively), low for mild impairment.^23^  A cut off of 7 has 75.2% sensitivity and 97.7% specificity for distinguishing  between demented and ‘‘mentally normal’’ elderly.^2^    CDT was proved to diagnose MCI subjects from normal elderly individuals, with higher sensitivity (74.7%) and specificity (75.6%).^24^ |
| Is this appropriate for the PD population? If not, why (low sensitivity and/or specificity values)? | CDT was found to be able to discriminate PDD from PD-CNT (p<0.001) and PD-MCI (p<0.001).^25^  CDT is sensitive across different H&Y stages (p<0.002).^26^ |
| **Clinimetric/psychometric properties in the target population** |  |
| **Feasibility /Acceptability** |  |
| Length | The test takes usually 2/3 minutes to be administrated; it can take longer in patients with movement disorders. |
| Ambiguities in instructions to patient | N |
| Ambiguities in rating anchors | N |
| Appropriateness of questions for population | Y |
| Applicability across disease cognitive stages | MCI/Dementia  Y  CDT is considered a good screening tool for dementia.^27^  Rouleau version is sensitive to the type of errors seen in non-demented patients with mild to moderate cognitive impairment.^28^  Particularly useful in the differential diagnosis between PD-NC and PD-MCI,^29^ as well as in the differential diagnosis PDD and DLB through the qualitative assessment.^30^ |
| Applicability across disease motor severity? | H&Y score: 1 (Unilateral involvement only)  Y  H&Y score: 2 or 3 (bilateral involvement with or without postural instability)  Y  H&Y score: ≥4 (several disabilities or confined to bed)  Probably N (depending on patient’s condition) |
| Clear instructions to raters | Y  Most versions clearly explain how to assign the scores |
| Has the test been used by researcher other than developers?  By other groups  Not applicable? | Y  The test has been widely used by other groups mostly for clinical purposes |
| Are there instructions for dealing with missing data?  Not applicable? | NA |
| Floor and ceiling effects, skewed score distributions? | Tendency toward higher scores. Skewed distribution.^19^ |

| **Have the component of dimensionality, variability and dependency structures been analyzed?**  (Exploratory and confirmatory factor analysis (EFA and CFA)? | N  There is a study on the 18 points CDT version.  I was not able to find studies on the 10 points CDT |
| --- | --- |
|  |  |
| **Reliability: assessed – good; not good. Not assessed**  Are alternative versions developed?  If so, were they validated with reliability measures? | N |
| Internal consistency (A rule of thumb)  0.9 ≤ Cronbach’s alpha (α) Excellent  0.8 ≤ α < 0.9 Good  0.7 ≤ α < 0.8 Acceptable  0.6 ≤ α < 0.7 Questionable  0.5 ≤ α < 0.6 Poor  α < 0.5 Unacceptable  (Streiner, 2003 Journal of Personality Assessment, 80:1, 99-103, DOI: 10.1207/S15327752JPA8001_18) | NA |
| “Corrected item-to-total correlation” | N |
| Test/retest | Y/N, if Y, specify how large |
| Inter-rater | Y  The 10 points CDT (Cahn et 1996) showed a “substantial” inter-rater reliability (0.91 the total score; 0.65 the qualitative score;^5^ 0.95 the quantitative score; all p<0.001).^16^  The 10-points CDT version by Wolf-Klein (1989) showed a intra rater reliability =0.81.^31^  A study involving Alzheimer’s patients and controls used the scales by Wolf Klein, Rouleau and Watson and found good inter-rater reliability.^27^ |
| Intra-rater | The 10 points CDT version by Wolf-Klein (1989) showed a good intra rater reliability (0.96).^2,31^ |
| Other (e.g. Standard Error of Measurement) | N |
| **Validity: assessed –good; not good. Not assessed (reference)** |  |
| Face and Content validity (does test appears to be measuring what it is intended to measure and do test’s items comprehensively represent the domain of interest?) | The test assesses a wide range of cognitive abilities besides the visuospatial capacities. It is therefore comparable to global cognitive scales such as MMSE or DRS with good correlation.^4,7,8^ |
| Criterion validity (compared to gold-standard) or other criteria for  diagnosis) (e.g. other comparable tests or lesion or MRI research) | Poor performance on the CDT is associated with focal brain damage in right parietal and left inferior frontoparietal opercular cortex.^32^  Visuospatial errors were mainly related to right hemisphere damage, whereas errors in time setting were mostly caused by left hemisphere lesions.^32^  The Rouleau total score was positively correlated with rCBF in the bilateral parietal and posterior temporal lobes and the right middle frontal gyrus. Numbers score was significantly positively correlated with rCBF in the right posterior temporal lobe and the left posterior middle temporal lobe, and hands score was significantly positively correlated with rCBF in the bilateral parietal lobes, the right posterior temporal lobe, the right middle frontal gyrus, and the right occipital lobe.^33^ |
| Construct validity (correlations with other convergent tests and divergent tests; known-groups comparisons) | Correlation with test: Trail Making Test Part A (r =0.48, p<0.001),  MMSE (r = 0.50, p<0.001),  Block Design Test (r = 0.56, p<0.001).^4^  Lack of reliance on verbal abilities.^1,34^ |
| Reference group used to develop this test:   49) If available, please define the characteristic of the norm group adopted (demographic variables used for stratification: age, education, sex, etc). 50) What kind of standardized score generated (T score, Z score, derived IQ score, percentile ranks, etc)? 51) If translated/validated in other languages, are reference norms created? If so, please specify demographic variables used for stratification (age, education, gender etc). | Group characteristics:  The average age in years (71.3±8.4, range 55-98)  The average education in years (16.6±2.7, range 11-24)  Sex (64.7% women)  Country USA  z-Scores and t-scores by age group are provided (Cahn version).^20^  Add the language of translation and group characteristics:  Language: Italian  872 healthy individuals. Age(yrs): range:20-94; mean: 53.16±18.27yrs. Education (yrs): range 1-27; mean:11.17±4.98; Sex (M/F)389/483  Country: Italy^17^  Language: Turkish  244 healthy individuals. Age(yrs): range:50-92; mean: 66.06±8.67yrs. Education (yrs): range 0-23; mean:12.11±4.32; Sex (M/F)89/155  Country: Turkey^22^  Language: Chinese  418 healthy volunteers Age(yrs): range: 35–84; mean: 63.03±7.79yrs. Education (yrs): range 0-18; mean: 9.32±3.14; 60.53% Males.  Country: China^20^ |

| **Responsiveness: assessed –good; not good. Not assessed (reference)** |  |
| --- | --- |
| Demonstrated to be sensitive to change (change over time or due to treatment)*  * [determined by the strength of the relationship between change in the test scores and the criterion or anchor scores] | Y  In a rTMS study in patients with PD, CDT showed improvement (6.5%) in T2 favoring active group.^35^ |
| Has the minimal clinically important change and minimal clinically relevant incremental difference been assessed?  (besides a statistically significant improvement) | N |
| Has this test been assessed or used in patients with other condition than that of interest? | Y in many conditions (AD, other dementias and neurological conditions -HD, MS) |
| Other |  |
| **Overall impression** |  |
| Strengths | It is a fast, easy to administer instrument with clear scoring instructions. It provides information on a wide range of cognitive abilities (especially when qualitative approach is used).  The different scoring systems have been found to be highly correlated therefore the version used is not a matter of concern.  Rouleau version is sensitive to the type of errors seen in non-demented patients with mild to moderate cognitive impairment.^28^  Used in PD population, an included among the best cognitive test to diagnose PD-MCI.^29^ |
| Weaknesses | Severe motor deficits can hamper its administration. |

| **Level of Recommendation** |  |
| --- | --- |
| “Recommended” – The test was applied to the target population, AND was studied clinimetrically and found to be valid, reliable and sensitive to change, AND was used in studies by researchers, others than the developers  “Recommended with caveats” – Test’s properties were generally found to be adequate, but some of the measurement properties were not evaluated or not evaluated specifically in a PD cohort."  ‘Suggested’ – The test was applied to the target population, but only one of the other criteria applies  ‘Listed” – The test was applied to the target population, but none of the other criteria applies | Please choose one of these options  Recommended with caveats |
| Which type of study is this test suitable for and which type of study is it not suitable for (screening, prevalence, etiological (e.g. case-control or genetic), treatment trial, correlation with biological markers or other scales, e.g. of parkinsonism, clinical practice for diagnosis/ screening). List all. | Suitable for:  Screening  Case-control study |

**REFERENCES**

1.	Sunderland T, Hill JL, Mellow AM, et al. Clock Drawing in Alzheimer’s Disease. Journal of the American Geriatrics Society [online serial]. 1989;37:725–729.

2.	Wolf-Klein GP, Silverstone FA, Levy AP, Brod MS. Screening for Alzheimer’s disease by clock drawing. J Am Geriatr Soc. 1989;37:730–734.

3.	Rouleau I, Salmon DP, Butters N, Kennedy C, McGuire K. Quantitative and qualitative analyses of clock drawings in Alzheimer’s and Huntington’s disease. Brain Cogn. 1992;18:70–87.

4.	Manos PJ, Wu R. The ten point clock test: a quick screen and grading method for cognitive impairment in medical and surgical patients. Int J Psychiatry Med. 1994;24:229–244.

5.	Cahn DA, Salmon DP, Monsch AU, et al. Screening for dementia of the alzheimer type in the community: the utility of the Clock Drawing Test. Arch Clin Neuropsychol. 1996;11:529–539.

6.	Juby A. Correlation between the Folstein Mini-Mental State Examination and three methods of clock drawing scoring. J Geriatr Psychiatry Neurol. 1999;12:87–91.

7.	Schramm U, Berger G, Müller R, Kratzsch T, Peters J, Frölich L. Psychometric properties of Clock Drawing Test and MMSE or Short Performance Test (SKT) in dementia screening in a memory clinic population. International Journal of Geriatric Psychiatry [online serial]. 2002;17:254–260.

8.	Richardson HE, Glass JN. A comparison of scoring protocols on the Clock Drawing Test in relation to ease of use, diagnostic group, and correlations with Mini-Mental State Examination. J Am Geriatr Soc. 2002;50:169–173.

9.	Kaplan E. A process approach to neuropsychological assessment. Clinical neuropsychology and brain function: Research, measurement, and practice. Washington, DC, US: American Psychological Association; 1988. p. 127–167.

10.	Shulman KI. Clock-drawing: is it the ideal cognitive screening test? Int J Geriatr Psychiatry. 2000;15:548–561.

11.	Mendez MF, Ala T, Underwood KL. Development of scoring criteria for the clock drawing task in Alzheimer’s disease. J Am Geriatr Soc. 1992;40:1095–1099.

12.	Royall DR, Cordes JA, Polk M. CLOX: an executive clock drawing task. J Neurol Neurosurg Psychiatry. 1998;64:588–594.

13.	Freedman M. Clock drawing: A neuropsychological analysis. Oxford University Press; 1994.

14.	Shulman KI, Shedletsky R, Silver IL. The challenge of time: Clock-drawing and cognitive function in the elderly. International Journal of Geriatric Psychiatry [online serial]. 1986;1:135–140.

15.	Lee HJ. Assembly-dependent endocytosis and clearance of extracellular [alpha]-synuclein. Int J Biochem Cell Biol. 2008;40:1835–1849.

16.	Hubbard EJ, Santini V, Blankevoort CG, et al. Clock drawing performance in cognitively normal elderly. Arch Clin Neuropsychol. 2008;23:295–327.

17.	Siciliano M, Santangelo G, D’Iorio A, et al. Rouleau version of the Clock Drawing Test: age- and education-adjusted normative data from a wide Italian sample. Clin Neuropsychol. 2016;30:1501–1516.

18.	Mondini S, Mapelli D, Vestri A, Arcara G, Bisiacchi PS. L’Esame neuropsicologico breve-2 (brief neuropsychological examination-2). Milano: Raffaello Cortina Editore. Epub 2011.

19.	Turcotte V, Gagnon M-E, Joubert S, et al. Normative data for the Clock Drawing Test for French-Quebec mid- and older aged healthy adults. The Clinical Neuropsychologist [online serial]. Routledge; Epub 2018 Nov 30.

20.	Shao K, Dong F, Guo S, et al. Clock‐drawing test: Normative data of three quantitative scoring methods for Chinese‐speaking adults in Shijiazhuang City and clinical utility in patients with acute ischemic stroke. Brain Behav [online serial]. 2020;10:e01806.

21.	Asl AM, Mehdizadeh M, Roudbari PR, Habibi S-A, Khatoon JN, Taghizadeh G. Reliability and validity of the Persian version of the clock drawing test in Iranian patients with idiopathic Parkinson’s disease. Journal of Clinical Physiotherapy Research. 2018;3:78–81.

22.	Savaş DDE, Yerlikaya D, Yener GG. Validity, Reliability and Turkish Norm Values of the Clock Drawing Test for Two Different Scoring Systems. Turk J Neurol [online serial]. Bayçınar Medical Publishing; 2018;24:143–152.

23.	Nishiwaki Y, Breeze E, Smeeth L, Bulpitt CJ, Peters R, Fletcher AE. Validity of the Clock-Drawing Test as a screening tool for cognitive impairment in the elderly. Am J Epidemiol. 2004;160:797–807.

24.	Yamamoto S, Mogi N, Umegaki H, et al. The clock drawing test as a valid screening method for mild cognitive impairment. Dement Geriatr Cogn Disord. 2004;18:172–179.

25.	Biundo R, Weis L, Pilleri M, et al. Diagnostic and screening power of neuropsychological testing in detecting mild cognitive impairment in Parkinson’s disease. J Neural Transm (Vienna). 2013;120:627–633.

26.	Siciliano M, De Micco R, Trojano L, et al. Cognitive impairment is associated with Hoehn and Yahr stages in early, de novo Parkinson disease patients. Parkinsonism Relat Disord. 2017;41:86–91.

27.	Aprahamian I, Martinelli JE, Neri AL, Yassuda MS. The Clock Drawing Test: A review of its accuracy in screening for dementia. Dement Neuropsychol. 2009;3:74–81.

28.	Seichepine DR, Neargarder S, Davidsdottir S, Reynolds GO, Cronin-Golomb A. Side and Type of Initial Motor Symptom Influences Visuospatial Functioning in Parkinson’s Disease. J Parkinsons Dis [online serial]. 2015;5:75–83.

29.	Goldman JG, Holden S, Ouyang B, Bernard B, Goetz CG, Stebbins GT. Diagnosing PD-MCI by MDS Task Force criteria: how many and which neuropsychological tests? Mov Disord. 2015;30:402–406.

30.	Cahn-Weiner DA, Williams K, Grace J, Tremont G, Westervelt H, Stern RA. Discrimination of dementia with lewy bodies from Alzheimer disease and Parkinson disease using the clock drawing test. Cogn Behav Neurol. 2003;16:85–92.

31.	Tuokko H, Hadjistavropoulos T, Rae S, O’Rourke N. A comparison of alternative approaches to the scoring of clock drawing. Arch Clin Neuropsychol. 2000;15:137–148.

32.	Tranel D, Rudrauf D, Vianna EPM, Damasio H. Does the Clock Drawing Test have focal neuroanatomical correlates? Neuropsychology. 2008;22:553–562.

33.	Matsuoka T, Narumoto J, Okamura A, et al. Neural correlates of the components of the clock drawing test. Int Psychogeriatr. 2013;25:1317–1323.

34.	Spreen O, Strauss E. A compendium of neuropsychological tests: Administration, norms, and commentary, 2nd ed. New York, NY, US: Oxford University Press; 1998. p. xvi, 736.

35.	Allosh T, Elbalkemy M, El Nahas N, Shalash A, Emara T, Elshiekh M. ROLE OF REPETITIVE TRANSCRANIAL MAGNETIC STIMULATION IN TREATMENT OF COGNITIVE MANIFESTATIONS IN PARKINSON’S DISEASE. Ain Shams Medical Journal [online serial]. 2020;71:281–292.

**Rating test evaluation**

| **Test name: Matrix Reasoning from WAIS-IV** | Responses (possible) |
| --- | --- |
| **Version**  Are there several versions (alternate or parallel) of the test? If so, which one has been assessed? | If yes, please add complete titles.  No |
| **Description of test**   - What population the test was originally developed for? - Which domains/components of domains do the test assess? - Are there multiple domains or a predominant domain assessed? - Does it score impaired/unimpaired? - Does it score severity   (subtle/mild/moderate/severe)? | - Subtest from Wechsler Adult Intelligence Scale, 4^th^ edition - Developed to evaluate intelligence and cognition in normal and clinical populations - This subtest (Matrix Reasoning) evaluates primarily visuoperceptual function and executive function (concept formation and abstract reasoning) - Does not provide impairment - Provides scaled score |
| **Scaling Metric**: Type of response format? (e.g., Likert, Binary, multiple-choice, continuum, reaction time, etc.) | Specify the scale of measurement:   - Raw score (total correct) is converted to age-corrected scaled score (mean 10, SD 3) |
| **Respondent: Patient:** any specific requirements for the target study population (e.g. non-demented, motor, auditory, visual abilities, etc)? | - Patient is respondent - Adequate auditory and visual function |
| **Access:** How can the test be obtained (mail address or website)? | In USA: pearsonassessments.com  Available in multiple other countries from the local representative of Pearson |
| Copyrighted or in public domain?  Conditions for use? Fee? | Copyrighted; test must be purchased |
| **Translations:** Has the test been translated and validated in other languages besides English? | Y/N, add language mutations with at least one validity study in the target population   - Yes - WAIS-IV is available in Canada, Germany, France, Italy, Japan, Greece, Taiwan, South Korea, Sweden, Lithuania, Slovenia, Belgium, Netherlands, Austria, Switzerland, Belgium, Netherlands, Australia and New Zealand. Some versions are translated, language adapted, and/or have local normative data |

| **Test properties**  (Please provide references for all statements or note if this is your judgment) |  |
| --- | --- |
| Which cognitive domain (or component/specific aspect of a cognitive domain) does this test *primarily* measure? | Visual perception and executive function (concept formation, abstraction, and reasoning)  Highest correlation of Matrix Reasoning is with other Perceptual Reasoning Index tests (Wechsler et al., 2008) |
| Which aspects of the domain you are assessing are not covered? | Cognitive flexibility, planning, response inhibition |

| **Use** |  |
| --- | --- |
| Was it *designed* to measure severity, screen or diagnosis of the domain? | Measures perceptual reasoning, an aspect of executive functioning; can set one’s own impairment cutoff |
| Is there a cut-off score? Are there measures of screening/diagnostic performance of the test? (Receiver characteristic curve analyses; sensitivity/specificity; Positive Predictive Value/Negative Predictive Value), True Positive Rate, False Positive Rate etc.) | No cutoff score: age-corrected scaled scores can be related to percentiles. No values given for diagnosis/screening |
| Is this appropriate for the PD population? If not, why (low sensitivity and/or specificity values)? | Yes |
| **Clinimetric/psychometric properties in the target population** |  |
| **Feasibility /Acceptability** |  |
| Length | Number of items/domains: 2 sample items and 26 test items  Average range in minutes: About 2-4 minutes for instructions and sample completion; no strict time limit for test items but response is encouraged after 30 sec. Should not take much more than 10-15 minutes, less if discontinuation criterion of 3 consecutive item failures is met |
| Ambiguities in instructions to patient | No |
| Ambiguities in rating anchors | No |
| Appropriateness of questions for population | Yes |
| Applicability across disease cognitive stages | Normal and MCI and early dementia. |
| Applicability across disease motor severity? | H&Y 1-3 |
| Clear instructions to raters | Yes, standard manualized instructions to test administrator |
| Has the test been used by researcher other than developers?  By other groups  Not applicable? | Yes, widely used |
| Are there instructions for dealing with missing data?  Not applicable? | Y/N, please specify  No |
| Floor and ceiling effects, skewed score distributions? | Please specify  Normalized distribution |

| **Have the component of dimensionality, variability and dependency structures been analyzed?**  (Exploratory and confirmatory factor analysis (EFA and CFA)? | Y/N Please specify  Yes, confirmatory factor analysis confirms loading on perceptual reasoning index (Wechsler et al., 2008). Matrix Reasoning loads 0.73 on the Perceptual Reasoning factor |
| --- | --- |
|  |  |
| **Reliability: assessed – good; not good. Not assessed**  Are alternative versions developed?  If so, were they validated with reliability measures? | Y/N Please specify  No alternate form |
| Internal consistency (A rule of thumb)  0.9 ≤ Cronbach’s alpha (α) Excellent  0.8 ≤ α < 0.9 Good  0.7 ≤ α < 0.8 Acceptable  0.6 ≤ α < 0.7 Questionable  0.5 ≤ α < 0.6 Poor  α < 0.5 Unacceptable  (Streiner, 2003 Journal of Personality Assessment, 80:1, 99-103, DOI: 10.1207/S15327752JPA8001_18) | 0.90 |
| “Corrected item-to-total correlation” | Y/N, if Y, specify how large  N |
| Test/retest | Y/N, if Y, specify how large  0.76 across 22 days |
| Inter-rater | Y/N, if Y, specify how large  N but presumably high |
| Intra-rater | Y/N, if Y, specify how large  N |
| Other (e.g. Standard Error of Measurement) | Y/N, if Y, specify how large  0.95 scaled score units |
| **Validity: assessed –good; not good. Not assessed (reference)** |  |
| Face and Content validity (does test appears to be measuring what it is intended to measure and do test’s items comprehensively represent the domain of interest?) | Y: expert review for face and content validity; interview with examines re, for example, strategy use |
| Criterion validity (compared to gold-standard) or other criteria for  diagnosis) (e.g. other comparable tests or lesion or MRI research) | Compared to prior Wechsler Adult Intelligence Scales (WAIS-III) (r=0.70) and Wechsler Intelligence Scale for Children (not relevant for PD) |
| Construct validity (correlations with other convergent tests and divergent tests; known-groups comparisons) | Convergent validity: r=0.54 with Block design, r=0.57 with Figure Weights, r=0.53 with Visual Puzzles, r=0.82 with Perceptual Reasoning Index  Divergent validity: correlations with working memory, verbal and processing speed subtests of the WAIS-IV r=0.39 to 0.52 (Wechsler et al, 2008) |
| Reference group used to develop this test:   52) If available, please define the characteristic of the norm group adopted (demographic variables used for stratification: age, education, sex, etc). 53) What kind of standardized score generated (T score, Z score, derived IQ score, percentile ranks, etc)? 54) If translated/validated in other languages, are reference norms created? If so, please specify demographic variables used for stratification (age, education, gender etc). | Group characteristics: 2,200 examinees closely matching in age, sex, education, geographic region and ethnicity distribution to US Census 2005  The average age in years (M, SD, range) Range 16-90 years; 13 age groups: 16-17, 18-19, 20-24, 25-29, 30-34, 35-44, 45-54, 55-64, 65-69, 70-74, 75-79, 80-84,85-90. N= 200 per group, except 4 oldest groups N=100  The average education in years (M, SD, range) 5 groups: ≤8, 9-11, 12, 13-15, and ≥16 years  Sex (% women): equal numbers of men and women in each age group, except 5 oldest groups that had more women than men to be consistent with US Census proportions.  Country:  Translations of WAIS-IV for many countries |

| **Responsiveness: assessed –good; not good. Not assessed (reference)** |  |
| --- | --- |
| Demonstrated to be sensitive to change (change over time or due to treatment)*  * [determined by the strength of the relationship between change in the test scores and the criterion or anchor scores] | 1. Matrix Reasoning unchanged 1 year after STN DBS (Yakufujiang et al, 2021) |
| Has the minimal clinically important change and minimal clinically relevant incremental difference been assessed?  (besides a statistically significant improvement) | Minimal clinically important change assessed Y/N?  N |
| Has this test been assessed or used in patients with other condition than that of interest? | Y/N, please specify if one or several condition  Y several, including Alzheimer’s, MCI |
| Other | MR score negatively associated with freezing of gait (Nantel et al 2012) |
| **Overall impression** |  |
| Strengths | Large normative sample, strong clinimetric validation, easy to administer, simple pointing response or verbal response (number) required, thus minimal if any motor demand;  Not impacted by apathy in PD (Brown et al 2019) |
| Weaknesses | small literature in PD for WAIS-IV; Walton, L., Domellöf, M. E., Boraxbekk, C. J., et al. (2021). Brown, D. S., Barrett, M. J., Flanigan, J. L., & Sperling, S. A. (2019). |

| **Level of Recommendation** |  |
| --- | --- |
| “Recommended” – The test was applied to the target population, AND was studied clinimetrically and found to be valid, reliable and sensitive to change, AND was used in studies by researchers, others than the developers  “Recommended with caveats” – Test’s properties were generally found to be adequate, but some of the measurement properties were not evaluated or not evaluated specifically in a PD cohort."  ‘Suggested’ – The test was applied to the target population, but only one of the other criteria applies  ‘Listed” – The test was applied to the target population, but none of the other criteria applies | Please choose one of these options  Recommended with caveats |
| Which type of study is this test suitable for and which type of study is it not suitable for (screening, prevalence, etiological (e.g. case-control or genetic), treatment trial, correlation with biological markers or other scales, e.g. of parkinsonism, clinical practice for diagnosis/ screening). List all. | Suitable for:  Case-control study  Unsuitable for (please add just categories for which the measure is critically unsuitable):  Screening  Prevalence  Differential reasoning (etiological)  Clinical Trials |

**Rating test evaluation**

| **Test name:** | Frontal Assessment Battery (FAB) |
| --- | --- |
| **Version**  Are there several versions (alternate or parallel) of the test? If so, which one has been assessed? | no |
| **Description of test**   - What population the test was originally developed for? - Which domains/components of domains do the test assess? - Are there multiple domains or a predominant domain assessed? - Does it score impaired/unimpaired? - Does it score severity   (subtle/mild/moderate/severe)? | The Frontal Assessment Battery (FAB) is a brief battery of six neuropsychological tasks that was specifically designed to assess frontal lobe function at bedside. The battery consists of six subtests exploring the following: conceptualization, mental flexibility, motor programming, sensitivity to interference, inhibitory control, and environmental autonomy.  The maximum score for each subtest is 3 points and the total score of test is calculated by adding the scores of the six subtests (maximum score=18).  It scores impaired/unimpaired.  It does not score severity (subtle/mild/moderate/severe) |
| **Scaling Metric**: Type of response format? (e.g., Likert, Binary, multiple-choice, continuum, reaction time, etc.) | Binary |
| **Respondent: Patient:** any specific requirements for the target study population (e.g. non-demented, motor, auditory, visual abilities, etc)? | Intact or well compensated auditory and  visual abilities. Motor impairments may influence the performance on test. |
| **Access:** How can the test be obtained (mail address or website)? | Dubois B, et al^1^ |
| Copyrighted or in public domain?  Conditions for use? Fee? | Public domain |
| **Translations:** Has the test been translated and validated in other languages besides English? | Italian^2, 3^  Spanish^4^  Brazilian^5^  German^6^  Spanish, Specific to PD^7^  Chinese^8^  Taiwanese (“Traditional Chinese”) ^9^  Japanese^10^ |

| **Test properties**  (Please provide references for all statements or note if this is your judgment) |  |
| --- | --- |
| Which cognitive domain (or component/specific aspect of a cognitive domain) does this test *primarily* measure? | Executive function |
| Which aspects of the domain you are assessing are not covered? |  |

| **Use** |  |
| --- | --- |
| Was it *designed* to measure severity, screen or diagnosis of the domain? | It was designed to measure and diagnose executive syndrome |
| Is there a cut-off score? Are there measures of screening/diagnostic performance of the test? (Receiver characteristic curve analyses; sensitivity/specificity; Positive Predictive Value/Negative Predictive Value), True Positive Rate, False Positive Rate etc.) | Cut-off score in relation to the diagnostic unit (MCI/dementia)  Slachevsky et al. ^11^ identified that a cutoff score of 12 on the FAB was optimal to distinguish FTD from AD in mildly demented patients.  Barulli et al. ^12^ noted that the receiver-operating characteristic curve showed that the optimal cut-off for the FAB in the whole sample of patients with amyotrophic lateral sclerosis was 16, with a sensitivity of 0.889 (95% CIs 0.545 to 1.000), a specificity of 0.593 (95% CI 0.450 to 0.907) and a moderate overall discriminatory power of 0.809 |
| Is this appropriate for the PD population? If not, why (low sensitivity and/or specificity values)? | The FAB is valid and appropriate for PD^7, 13, 14^ |
| **Clinimetric/psychometric properties in the target population** |  |
| **Feasibility /Acceptability** |  |
| Length | 6 tasks  Approximately 10-15 minutes |
| Ambiguities in instructions to patient | no |
| Ambiguities in rating anchors | no |
| Appropriateness of questions for population |  |
| Applicability across disease cognitive stages | Yes  The FAB can be recommended as a valid instrument for PD-MCI Level I screening^15^. |
| Applicability across disease motor severity? | H&Y score: 1 (Unilateral involvement only)  H&Y score: 2 or 3(bilateral involvement with or without postural instability)  H&Y score: ≥4(several disabilities or confined to bed) |
| Clear instructions to raters | Yes |
| Has the test been used by researcher other than developers?  By other groups  Not applicable? | Yes |
| Are there instructions for dealing with missing data?  Not applicable? |  |
| Floor and ceiling effects, skewed score distributions? | not reported |

| **Have the component of dimensionality, variability and dependency structures been analyzed?**  (Exploratory and confirmatory factor analysis (EFA and CFA)? | | No |
| --- | --- | --- |
|  |  | |
| **Reliability: assessed – good; not good. Not assessed**  Are alternative versions developed?  If so, were they validated with reliability measures? | yes, it is assessed as good  there are not alternative versions. | |
| Internal consistency (A rule of thumb)  0.9 ≤ Cronbach’s alpha (α) Excellent  0.8 ≤ α < 0.9 Good  0.7 ≤ α < 0.8 Acceptable  0.6 ≤ α < 0.7 Questionable  0.5 ≤ α < 0.6 Poor  α < 0.5 Unacceptable  (Streiner, 2003 Journal of Personality Assessment, 80:1, 99-103, DOI: 10.1207/S15327752JPA8001_18) | The Spanish version of the FAB^7^ showed good internal consistency (α = 0.751) in PD patients. | |
| “Corrected item-to-total correlation” | No | |
| Test/retest | The test-retest reliability indicated strong correlations with estimates ranging between 0.494 and 0.864 in the same Spanish version of the FAB subtests^7^ as observed in the intra-rater coefficients. | |
| Inter-rater | No | |
| Intra-rater | Yes, Intra-rater reliability of the Spanish version of the FAB subtests was moderately good or good, with estimates ranging from 0.559 (conflicting instructions item) to 0.891 (prehension behaviour item) ^7.^ | |
| Other (e.g. Standard Error of Measurement) | No. | |
| **Validity: assessed –good; not good. Not assessed (reference)** | The validity in PD patients is good | |
| Face and Content validity (does test appears to be measuring what it is intended to measure and do test’s items comprehensively represent the domain of interest?) | Yes from observation of the tasks | |
| Criterion validity (compared to gold-standard) or other criteria for  diagnosis) (e.g. other comparable tests or lesion or MRI research) | Yes,  The total and subtest scores of the FAB-E generally showed strong correlations for the MMSE and the tests of EFs, except for the prehension behaviour item of the FAB-E and the Interference and Go/no-go tasks of the EXIT-25 that globally presented low estimates for all the neuropsychological tests^7^. | |
| Construct validity (correlations with other convergent tests and divergent tests; known-groups comparisons) | Convergent validity   - In PD, FAB scores were significantly decreased compared to normal controls, and correlated with measures of executive functions such as phonemic and semantic verbal fluency tests, Wisconsin Card Sorting Test and Trail Making Test Part A and Part B. The FAB is a useful tool for the screening of executive dysfunction in PD, showing good discriminant and concurrent validities^13^. - In PD, highly significant correlations were found between the total FAB score and tests of attention, executive functions, and memory^16^.   Group-comparisons  The FAB has shown discriminative validity for the differentiation of PD-MCI from PD-NC and controls (area under the curve >.80) ^15^. | |
| Reference group used to develop this test:  If available, please define the characteristic of the norm group adopted (demographic variables used for stratification: age, education, sex, etc).  What kind of standardized score generated (T score, Z score, derived IQ score, percentile ranks, etc)?  If translated/validated in other languages, are reference norms created? If so, please specify demographic variables used for stratification (age, education, gender etc). | Group characteristics:  The average age in years (M, SD, range): Forty-two normal control subjects (mean 6 SD; age, 58 6 14.4 years), without any neurologic or psychiatric history, were included.  The average education in years (M, SD, range): not reported  Sex (% women): not reported  Country: France  Add the type of standardized score: not reported  Add the language of translation and group characteristics:  German version^6^  The average age in years (M: 69,03, SD: 3.0, range 50-95).  The average education in years (M: 12,96, SD: 3.3, range, not reported)  Sex (53% women)  Country: Germany  Japanese version^10.^ See table 1 for characteristics of patients and controls   Diagnoses in the outpatients were as follows: AD, n=58 (14 men, 44 women; age range, 46–90 years; mean age, 75.3±7.8 years), VaD or vascular cognitive impairment, n=24 (16 men, 8 women; age range, 57–87 years; mean age, 75.1±9.3 years), and FTD, n=23 (9 men, 14 women; age range, 50–83 years; mean age, 64.7±9.5 years).  Slovak version of FAB^18^  The final sample consisted of 487 healthy adults (54% of them female). The mean age in our sample was M = 55.29 (SD = 19.96).    -Italian version of the FAB^3^  The sample was of 364 with 215 women (59%). Mean age for the whole sample was 57.4±17.9 years (range 20–94 years) and mean duration of education was 10.4±4.3 years (range 1–17 years, the latter being conventionally assigned to all subjects with a degree). The subjects were recruited in different districts of the provinces of Milan and Modena, both in rural or suburban areas and in city centres  Chinese version^4^  41 controls, 30 patients with small subcortical infarcts. Mean age of controls (SD) was 69.6 (9.9). Years of education was 5.4 (4.9). 58.5% female. | |

| **Responsiveness: assessed –good; not good. Not assessed (reference)** | Not evaluated |
| --- | --- |
| Demonstrated to be sensitive to change (change over time or due to treatment)*  * [determined by the strength of the relationship between change in the test scores and the criterion or anchor scores] | If Yes, please specify |
| Has the minimal clinically important change and minimal clinically relevant incremental difference been assessed?  (besides a statistically significant improvement) | Minimal clinically important change assessed Y/N? |
| Has this test been assessed or used in patients with other condition than that of interest? | Y/N, please specify if one or several condition |
| Other |  |
| **Overall impression** |  |
| Strengths | The strength of the FAB in clinical practice is the brevity of the scale, it is easy to administer and valid. |
| Weaknesses | The Weaknesses include the presence of items with motor response (for example, Go-no- go task). Severe parkinsonism might influence negatively on cognitive performance. |

| **Level of Recommendation** |  |
| --- | --- |
| “Recommended” – The test was applied to the target population, AND was studied clinimetrically and found to be valid, reliable and sensitive to change, AND was used in studies by researchers, others than the developers  “Recommended with caveats” – Test’s properties were generally found to be adequate, but some of the measurement properties were not evaluated or not evaluated specifically in a PD cohort."  ‘Suggested’ – The test was applied to the target population, but only one of the other criteria applies  ‘Listed” – The test was applied to the target population, but none of the other criteria applies | “Recommended with caveats” |
| Which type of study is this test suitable for and which type of study is it not suitable for (screening, prevalence, etiological (e.g. case-control or genetic), treatment trial, correlation with biological markers or other scales, e.g. of parkinsonism, clinical practice for diagnosis/ screening). List all. | Suitable for:  Screening: X  Prevalence: X  Differential reasoning (etiological)  Case-control study:X  Treatment trial  Biomarker study  Other  Unsuitable for (please add just categories for which the measure is critically unsuitable):  Screening  Prevalence  Differential reasoning (etiological)  Case-control study  Treatment trial: X  Biomarker study:X  Other |

**REFERENCES**

(1) Dubois B, Slachevsky A, Litvan I, Pillon B. The FAB: a Frontal Assessment Battery at bedside. Neurology. 2000 Dec 12;55(11):1621-6. doi: 10.1212/wnl.55.11.1621.
(2) Aiello EN, Esposito A, Gramegna C, Gazzaniga V, Zago S, Difonzo T, Appollonio IM, Bolognini N. The Frontal Assessment Battery (FAB) and its sub-scales: validation and updated normative data in an Italian population sample. Neurol Sci. 2021 Jun 29. doi: 10.1007/s10072-021-05392-y. Epub ahead of print. PMID: 34184168.
(3) Appollonio I, Leone M, Isella V, Piamarta F, Consoli T, Villa ML, Forapani E, Russo A, Nichelli P. The Frontal Assessment Battery (FAB): normative values in an Italian population sample. Neurol Sci. 2005 Jun;26(2):108-16. doi: 10.1007/s10072-005-0443-4. PMID: 15995827.
(4) Hurtado-Pomares M, Valera-Gran D, Sánchez-Pérez A, Peral-Gómez P, Navarrete-Muñoz EM, Terol-Cantero MC. Adaptation of the Spanish version of the Frontal Assessment Battery for detection of executive dysfunction. Med Clin (Barc). 2021 Mar 12;156(5):229-232. English, Spanish. doi: 10.1016/j.medcli.2020.04.028. Epub 2020 Sep 9. PMID: 32919777.
(5) Beato R, Amaral-Carvalho V, Guimarães HC, Tumas V, Souza CP, Oliveira GN, Caramelli P. Frontal assessment battery in a Brazilian sample of healthy controls: normative data. Arq Neuropsiquiatr. 2012 Apr;70(4):278-80. doi: 10.1590/s0004-282x2012005000009. Epub 2012 Feb 24. PMID: 22358310.
(6) Benke T, Karner E, Delazer M. FAB-D: German version of the Frontal Assessment Battery. J Neurol. 2013 Aug;260(8):2066-72. doi: 10.1007/s00415-013-6929-8. Epub 2013 May 7. PMID: 23649609.
(7) Hurtado-Pomares M, Terol-Cantero MC, Sánchez-Pérez A, Leiva-Santana C, Peral-Gómez P, Valera-Gran D, Navarrete-Muñoz EM. Measuring executive dysfunction in Parkinson's disease: Reliability and validity of the Spanish version of Frontal Assessment Battery (FAB-E). PLoS One. 2018 Nov 19;13(11):e0207698. doi: 10.1371/journal.pone.0207698. PMID: 30452476; PMCID: PMC6242688.
(8) Mok VC, Wong A, Yim P, Fu M, Lam WW, Hui AC, Yau C, Wong KS. The validity and reliability of Chinese frontal assessment battery in evaluating executive dysfunction among Chinese patients with small subcortical infarct. Alzheimer Dis Assoc Disord. 2004 Apr-Jun;18(2):68-74. doi: 10.1097/01.wad.0000126617.54783.7. PMID: 15249850.
(9) Tzu-Lan Wang, Yi-Hsiang Hung & Chi-Cheng Yang (2016) Psychometric Properties of the Taiwanese (Traditional Chinese) Version of the Frontal Assessment Battery: A Preliminary Study, Applied Neuropsychology: Adult, 23:1, 11-20, DOI: [10.1080/23279095.2014.995792](https://doi.org/10.1080/23279095.2014.995792)
(10) Kugo A, Terada S, Ata T, Ido Y, Kado Y, Ishihara T, Hikiji M, Fujisawa Y, Sasaki K, Kuroda S. Japanese version of the Frontal Assessment Battery for dementia. Psychiatry Res. 2007 Sep 30;153(1):69-75. doi: 10.1016/j.psychres.2006.04.004. Epub 2007 Jun 27. PMID: 17599465.
(11) Slachevsky A, Villalpando JM, Sarazin M, Hahn-Barma V, Pillon B, Dubois B. Frontal assessment battery and differential diagnosis of frontotemporal dementia and Alzheimer disease. Arch Neurol. 2004 Jul;61(7):1104-7. doi: 10.1001/archneur.61.7.1104. PMID: 15262742.
(12) Barulli MR, Fontana A, Panza F, Copetti M, Bruno S, Tursi M, Iurillo A, Tortelli R, Capozzo R, Simone IL, Logroscino G. Frontal assessment battery for detecting executive dysfunction in amyotrophic lateral sclerosis without dementia: a retrospective observational study. BMJ Open. 2015 Sep 3;5(9):e007069. doi: 10.1136/bmjopen-2014-007069. PMID: 26338835; PMCID: PMC4563243.
(13) Lima CF, Meireles LP, Fonseca R, Castro SL, Garrett C. The Frontal Assessment Battery (FAB) in Parkinson's disease and correlations with formal measures of executive functioning. J Neurol. 2008 Nov;255(11):1756-61. doi:10.1007/s00415-008-0024-6. Epub 2008 Sep 25. PMID: 18821046.
(14) Paviour DC, Winterburn D, Simmonds S, Burgess G, Wilkinson L, Fox NC, Lees AJ, Jahanshahi M. Can the frontal assessment battery (FAB) differentiate bradykinetic rigid syndromes? Relation of the FAB to formal neuropsychological testing. Neurocase. 2005 Aug;11(4):274-82. doi: 10.1080/13554790590962933. PMID:16093228.
(15) Bezdicek O, Růžička F, Fendrych Mazancova A, Roth J, Dušek P, Mueller K, Růžička E, Jech R. Frontal Assessment Battery in Parkinson's Disease: Validity and Morphological Correlates. J Int Neuropsychol Soc. 2017 Sep;23(8):675-684. doi: 10.1017/S1355617717000522. Epub 2017 Jul 18. PMID: 28716165.
(16) Cohen OS, Vakil E, Tanne D, Molshatzki N, Nitsan Z, Hassin-Baer S. The frontal assessment battery as a tool for evaluation of frontal lobe dysfunction in patients with Parkinson disease. J Geriatr Psychiatry Neurol. 2012 Jun;25(2):71-7. doi: 10.1177/0891988712445087. PMID: 22689698
(17) Benke T, Karner E, Delazer M. FAB-D: German version of the Frontal Assessment Battery. J Neurol. 2013 Aug;260(8):2066-72. doi: 10.1007/s00415-013-6929-8. Epub 2013 May 7. PMID: 23649609)
(18) Abrahámová M, Smolejová E, Dančík D, Pribišová K, Heretik A Jr, Hajdúk M. Normative data for the Slovak version of the Frontal Assessment Battery (FAB). Appl Neuropsychol Adult. 2020 Apr 16:1-6. doi:10.1080/23279095.2020.1748031

**Rating test evaluation**

| **Test name:** | **Iowa Gambling Task** |
| --- | --- |
| **Version**  Are there several versions (alternate or parallel) of the test? If so, which one has been assessed? | Yes.  The original version has been developed in 1994 (1). The original IGT utilized four decks of paper cards and a set of play money. Participants were given $2000 and told to maximize profit by selecting 100 cards from four decks (A,B,C, and D). Participants were not told the relative risks/benefits of each deck, learning this information through feedback. Originally, the IGT was administered using decks of paper, however the computerized version of task is now commonly used, with no differences between the two versions (2–4).  There is another version of the task: IGT, version 2 (5). In this version, Decks A and B are still disadvantageous and Decks C and D advantageous, but the amounts gained/lost vary. Furthermore, on the original IGT there is an unlimited number of cards in each deck, whereas on the IGT-2 participants are limited to 60 selections from one deck.  Alternative versions of the IGT have been developed for addressing the issue of practice effect i.e., the K-IGT, an alternative version of the IGT with decks K′L′M′N′, and the Q-IGT, an alternative version of the IGT with decks Q′R′S′T′ (6).  Other computerized versions of the task exist (e.g., IGT-Open (7)) and they may differ in some aspects, like instructions, timing, and visual presentation (e.g., (8,9).  Here, the original version of the task has been assessed as it is the version most commonly used. When a different version (e.g., IGT-2) has been assessed, this has been specified in the text. |
| **Description of test**   - What population the test was originally developed for? - Which domains/components of domains do the test assess? - Are there multiple domains or a predominant domain assessed? - Does it score impaired/unimpaired? - Does it score severity   (subtle/mild/moderate/severe)? | - The IGT has been developed for ventromedial prefrontal cortex damaged patients (1). - Executive function, in particular decision-making under uncertainty (first decks) or decision-making under risk (second decks) (10). - There are no multiple domains assessed. - It scores impaired/unimpaired. - Lower net scores indicate poorer performances. |
| **Scaling Metric**: Type of response format? (e.g., Likert, Binary, multiple-choice, continuum, reaction time, etc.) | Multiple-choice: participants have to choose between 4 decks. Decks A and B are disadvantageous and Decks C and D are advantageous.  The standard performance measure of the IGT is the “net score” (obtained from subtracting the number of cards selected from the disadvantageous decks from the number selected from the advantageous decks).  Other indicators are net scores for each block of 20 cards/trials (as measure of learning across trials), the total number of cards selected from each deck and the total amount of money won. |
| **Respondent: Patient:** any specific requirements for the target study population (e.g. non-demented, motor, auditory, visual abilities, etc)? | Preserved vision. |
| **Access:** How can the test be obtained (mail address or website)? | A computerized version of the original task (1) can be found here: <https://www.psytoolkit.org/experiment-library/igt.html>  IGT version 2 can be obtained here: <https://www.parinc.com/Products/Pkey/203> |
| Copyrighted or in public domain?  Conditions for use? Fee? | Apparently, the original version is in public domain e.g., psytoolkit (11) and free to use.  IGT version 2 is copyrighted. IGT version 2 kit (manual and software) costs $735.00.  Another free version of the task is the IGT-Open (7).  The IGT requires skills/appropriate training in the administration and interpretation of psychological tests. |
| **Translations:** Has the test been translated and validated in other languages besides English? | Yes.  Brazilian Portuguese (12). |

| **Test properties**  (Please provide references for all statements or note if this is your judgment) |  |
| --- | --- |
| Which cognitive domain (or component/specific aspect of a cognitive domain) does this test *primarily* measure? | Executive function – decision-making |
| Which aspects of the domain you are assessing are not covered? | It does not cover other executive function abilities (e.g., inhibition, set-shifting, etc.) (personal opinion). |

| **Use** |  |
| --- | --- |
| Was it *designed* to measure severity, screen or diagnosis of the domain? | It was designed to measure severity of decision-making impairments.  Poorer scores do not automatically imply prefrontal cortex, or ventromedial prefrontal cortex damage or dysfunction ([https://www.parinc.com/WebUploads/ samplerpts/IGT2_Impaired.pdf](https://www.parinc.com/WebUploads/samplerpts/IGT2_Impaired.pdf)). |
| Is there a cut-off score? Are there measures of screening/diagnostic performance of the test? (Receiver characteristic curve analyses; sensitivity/specificity; Positive Predictive Value/Negative Predictive Value), True Positive Rate, False Positive Rate etc.) | Normative data and cut-off scores are provided in the manual (5) (no access to the manual). Normative data is reported in T scores and percentiles scores. |
| Is this appropriate for the PD population? If not, why (low sensitivity and/or specificity values)? | There is not specific information regarding the appropriateness of IGT in PD in the original manual. Results regarding the IGT impairment in PD are still controversial ((13) (14)). A recent meta-analysis found an small effect size and high heterogeneity across studies (13) |
| **Clinimetric/psychometric properties in the target population** |  |
| **Feasibility /Acceptability** |  |
| Length | 100 trials (number of choices to be made).  It takes around 5-10 minutes to be completed (5). |
| Ambiguities in instructions to patient | No (personal judgment) |
| Ambiguities in rating anchors | No |
| Appropriateness of questions for population | Yes (personal judgement) |
| Applicability across disease cognitive stages | Yes. It has been extensively used in PD (see the meta-analysis of (13–15)).  It has also been used in PDD (16). |
| Applicability across disease motor severity? | It has been used across H&Y stages 1 to 4 [for a review see (17) and Colautti et al 2021)].  It has also been used in early-onset PD (18), Poletti et al 2010, (19–21) |
| Clear instructions to raters | Yes (personal judgment) |
| Has the test been used by researcher other than developers?  By other groups  Not applicable? | Yes, and by many other groups. |
| Are there instructions for dealing with missing data?  Not applicable? | No instructions for missing data found (no access to the manual).  However, net score is normally computed when participant complete all 100 trials. |
| Floor and ceiling effects, skewed score distributions? | The performance of healthy participants is characterized by high within-group variability, and a substantial number of participants violate the assumption that healthy participants prefer the good decks over the bad decks therefore performing deficiently (22). |

| **Have the component of dimensionality, variability and dependency structures been analyzed?**  (Exploratory and confirmatory factor analysis (EFA and CFA)? | In undergraduate students, confirmatory factor analysis based on the results of exploratory factor analysis showed that the best fitting model for comparing IGT, BART, and CCT-cold performance is a model in which the IGT and BART assess separate components of decision making, and the early trials on the IGT assess a different type of decision making than the later trials. The CCT-cold does not fit well into this model (23). |
| --- | --- |
|  |  |
| **Reliability: assessed – good; not good. Not assessed**  Are alternative versions developed?  If so, were they validated with reliability measures? | Yes.  Among the alternative versions of the task, the IGT-2 (5) has been tested for test/retest reliability.  When the IGT-2 has been used, this has been specified (see “test/retest section”). |
| Internal consistency (A rule of thumb)  0.9 ≤ Cronbach’s alpha (α) Excellent  0.8 ≤ α < 0.9 Good  0.7 ≤ α < 0.8 Acceptable  0.6 ≤ α < 0.7 Questionable  0.5 ≤ α < 0.6 Poor  α < 0.5 Unacceptable  (Streiner, 2003 Journal of Personality Assessment, 80:1, 99-103, DOI: 10.1207/S15327752JPA8001_18) | Not found. |
| “Corrected item-to-total correlation” | Not found. |
| Test/retest | Not found in PD.  In non-PD populations [if not otherwise specified, IGT original version (1) has been tested]:   - Tests-retest reliability within one session: r = .60, based on N = 60 undergraduate students (24). - 2-weeks interval, probably using the IGT-2 (Bechara et al., 2007): within the range of r =.35 to r = .65, based on N = 40 students (25). - 3-weeks interval using the IGT-2, (Bechara et al., 2007): Time 1 performance in Trials 1–40 was not correlated with Time 2 performance, but Time 1 performance in Trials 41–100 was weakly correlated with all Time 2 IGT trials (r = .27), based on N = 98 undergraduate students (10) - years interval: r = .19–.74, based on 11-18 years twins (26). |
| Inter-rater | Not found. |
| Intra-rater | Not found. |
| Other (e.g. Standard Error of Measurement) | NA |
| **Validity: assessed –good; not good. Not assessed (reference)** |  |
| Face and Content validity (does test appears to be measuring what it is intended to measure and do test’s items comprehensively represent the domain of interest?) | Not found. |
| Criterion validity (compared to gold-standard) or other criteria for  diagnosis) (e.g. other comparable tests or lesion or MRI research) | PET data showed bilateral activation of Dorsolateral PFC, right mesial OFC, right cingulate and left caudate in healthy controls (n=5), In PD (n=5), dlPFC bilateral activation, left caudate was remarkable but OFC was hypoactivein PD. Impairment in cortical-subcortical loop was suggested. (27)  In PD patients, Ibarretxe-Bilbao et al (21) showed that left lateral GM volume in OFC showed a slight correlation with the IGT. Later, Kobayakawa et al (28) also showed a correlation between the lateral orbitofrontal volume and IGT performance.  Lesion and fMRI data support the notion that IGT performance is related to frontal lobe functioning, in particular ventromedial prefrontal cortex (29). |
| Construct validity (correlations with other convergent tests and divergent tests; known-groups comparisons) | In PD [if not otherwise specified, IGT original version (1) has been tested]:   - IGT measure was negatively associated to BIS-11 (impulsivity) and BAS (reward) scale (respectively r=−0.513, p≤0.001; r=- 0.530, p≤0.001) (30). - No correlation between IGT and the Tower of London or Stroop tests (18). - No correlation between the IGT and the WCST, digit span forward and backward, Ray figure (31) - No correlations between IGT and other executive function measures (28,32–35) - No correlation between the IGT and the WCST or Stroop test in early PD, late PD or healthy (36). But significant correlation was obtained with BDI-II in early PD. - Negative correlations between IGT and general intellectual functioning, delayed free recall, visuospatial abilities and verbal fluency (37)   In non-PD samples:   - in a meta-analysis, correlations between IGT and other executive function tasks: r = .17 [.05, .27] (38). - minimal correlations between IGT and the BART, the CCT and the GDT in undergraduate students, providing support that each task likely measures a separate component of decision-making – using the IGT-2 (10). - No relationship between IGT and the WCST in healthy population (39). - No relationship between IGT and WCST among alcohol and stimulant abusers (40,41). - No correlation between IGT and the BART among smokers (24). - In normal healthy subjects, Block net raw scores (2, 3, 4 and 5) significantly correlated with different measures of the WCST (low to moderate Cohen effect). No significant correlations were reported with Tower of Hanoi (42) |
| Reference group used to develop this test:   55) If available, please define the characteristic of the norm group adopted (demographic variables used for stratification: age, education, sex, etc). 56) What kind of standardized score generated (T score, Z score, derived IQ score, percentile ranks, etc)? 57) If translated/validated in other languages, are reference norms created? If so, please specify demographic variables used for stratification (age, education, gender etc). | Group characteristics:  From Bechara et al. (1994) (1):  Healthy controls  Age range: 20-79 years  Education: “about half the number of subjects had a high school education, and the other half had a college education” (Bechara et al., 1994).  Sex (47.72 % women)  US  The standardized scores generated and reported in the original manual are T scores and percentiles.  Translation in Brazilian Portuguese (12):  Average age in years: M = 32.1, SD = 8.5.  Average education in years: M = 13, SD = 2.2.  Sex (60% women)  Brazil |

| **Responsiveness: assessed –good; not good. Not assessed (reference)** |  |
| --- | --- |
| Demonstrated to be sensitive to change (change over time or due to treatment)*  * [determined by the strength of the relationship between change in the test scores and the criterion or anchor scores] | In PD, Preoperative STN-DBS performance on the IGT was significantly impaired compared to after surgery (43). |
| Has the minimal clinically important change and minimal clinically relevant incremental difference been assessed?  (besides a statistically significant improvement) | No |
| Has this test been assessed or used in patients with other condition than that of interest? | Yes  It has also been used other conditions e.g., pathological gamblers, cocaine users, HIV-infected, schizophrenia, alcohol and substance abuse, anorexia nervosa, Binge Eating Disorder, ADHD, bipolar disorder, mania, Huntington’s disease, Alzheimer’s disease, borderline personality disorder, behavioural variant frontotemporal dementia. |
| Other | Neither dopaminergic drug level, nor disease duration or severity of motor symptoms explained heterogeneity in IGT performance (15). |
| **Overall impression** |  |
| Strengths | It is not affected by dopaminergic level, disease duration, severity of motor symptoms.  It is scarcely associated to performance in other executive tasks. |
| Weaknesses | Performance on the IGT is more advantageous at Time 2 compared to Time 1, which could be indicative of practice effects (10,44,45)  Practice effect may limit its use for assessing performance over time (e.g., in pre/post intervention studies) (29).  Performance could be influenced by the frequency of losses rather than the long-term outcomes resulting in poor performance and complicating the interpretation of the data (22) |

| **Level of Recommendation** |  |
| --- | --- |
| “Recommended” – The test was applied to the target population, AND was studied clinimetrically and found to be valid, reliable and sensitive to change, AND was used in studies by researchers, others than the developers  “Recommended with caveats” – Test’s properties were generally found to be adequate, but some of the measurement properties were not evaluated or not evaluated specifically in a PD cohort."  ‘Suggested’ – The test was applied to the target population, but only one of the other criteria applies  ‘Listed” – The test was applied to the target population, but none of the other criteria applies | Recommended with caveats: The test was applied to the target population AND was used in studies by researchers, others than the developers. However, only test/retest reliability and construct validity have been investigated in a PD cohort. |
| Which type of study is this test suitable for and which type of study is it not suitable for (screening, prevalence, etiological (e.g. case-control or genetic), treatment trial, correlation with biological markers or other scales, e.g. of parkinsonism, clinical practice for diagnosis/ screening). List all. | Suitable for case-control studies (between group differences).  It should be used with cautious in treatment trials due to possible practice effect bias.    Not suitable for screening/prevalence studies as it is not a screening measure. |

**REFERENCES**

1.	Bechara A, Damasio AR, Damasio H, Anderson SW. Insensitivity to future consequences following damage to human prefrontal cortex. Cognition. 1994;50:7–15.

2.	Bechara A, Tranel D, Damasio H. Characterization of the decision-making deficit of patients with ventromedial prefrontal cortex lesions. Brain. 2000;123(11):2189–202.

3.	Bowman CH, Turnbull OH. Real versus facsimile reinforcers on the Iowa Gambling Task. Brain Cogn. 2003;53(2):207–10.

4.	Bowman CH, Evans CEY, Turnbull OH. Artificial time constraints on the Iowa Gambling Task: The effects on behavioural performance and subjective experience. Brain Cogn. 2005;57(1):21–5.

5.	Bechara A. Iowa Gambling Task Professional Manual. Psychological Assessment Resources, Inc. 2007.

6.	Xiao L, Wood SMW, Denburg NL, Moreno GL, Hernandez M, Bechara A. Is there a recovery of decision-making function after frontal lobe damage? A study using alternative versions of the Iowa Gambling Task. J Clin Exp Neuropsychol. 2013;35(5):518–29.

7.	Dancy CL, Ritter FE. IGT-Open: An open-source, computerized version of the Iowa Gambling Task. Behav Res Methods. 2017;49(3):972–8.

8.	Balodis IM, MacDonald TK, Olmstead MC. Instructional cues modify performance on the Iowa Gambling Task. Brain Cogn. 2006;60(2):109–17.

9.	Overman WH, Pierce A. Iowa Gambling Task with non-clinical participants: Effects of using real + virtual cards and additional trials. Front Psychol. 2013;4(DEC):1–15.

10.	Buelow MT, Barnhart WR. Test-retest reliability of common behavioral decision making tasks. Archives of Clinical Neuropsychology. 2018;33(1):125–9.

11.	Stoet G. PsyToolkit: A Novel Web-Based Method for Running Online Questionnaires and Reaction-Time Experiments. Teaching of Psychology. 2017;44(1):24–31.

12.	Malloy-Diniz LF, Leite WB, De Moraes PHP, Correa H, Bechara A, Fuentes D. Brazilian Portuguese version of the Iowa Gambling Task: Transcultural adaptation and discriminant validity. Revista Brasileira de Psiquiatria. 2008;30(2):144–8.

13.	Colautti L, Iannello P, Silveri MC, Antonietti A. Decision making in Parkinson’s disease: An analysis of the studies using the Iowa Gambling Task. Vol. 54, European Journal of Neuroscience. John Wiley and Sons Inc; 2021. p. 7513–49.

14.	Salvatore MF, Soto I, Alphonso H, Cunningham R, James R, Nejtek VA. Is there a Neurobiological Rationale for the Utility of the Iowa Gambling Task in Parkinson’s Disease? Vol. 11, Journal of Parkinson’s Disease. IOS Press BV; 2021. p. 405–19.

15.	Evens R, Hoefler M, Biber K, Lueken U. The Iowa Gambling Task in Parkinson’s disease: A meta-analysis on effects of disease and medication. Neuropsychologia. 2016;91:163–72.

16.	Delazer M, Sinz H, Zamarian L, Stockner H, Seppi K, Wenning GK, et al. Decision making under risk and under ambiguity in Parkinson’s disease. Neuropsychologia. 2009;47(8):1901–8.

17.	Salvatore MF, Soto I, Alphonso H, Cunningham R, James R, Nejtek VA. Is there a Neurobiological Rationale for the Utility of the Iowa Gambling Task in Parkinson’s Disease? J Parkinsons Dis. 2021;11(2):405–19.

18.	Gescheidt T, Czekóová K, Urbánek T, Mareček R, Mikl M, Kubíková R, et al. Iowa Gambling Task in patients with early-onset Parkinson’s disease: Strategy analysis. Neurological Sciences. 2012;33(6):1329–35.

19.	Poletti M, Frosini D, Lucetti C, Del Dotto P, Ceravolo R, Bonuccelli U. Iowa gambling task in de novo Parkinson’s disease: A comparison between good and poor performers. Movement Disorders [Internet]. 2012 Feb 1 [cited 2025 Mar 20];27(2):330–2. Available from: https://onlinelibrary.wiley.com/doi/full/10.1002/mds.23982

20.	Poletti M, Frosini D, Lucetti C, Del Dotto P, Ceravolo R, Bonuccelli U. Decision making in de novo Parkinson’s disease. Movement Disorders. 2010 Jul 30;25(10):1432–6.

21.	Ibarretxe-Bilbao N, Junque C, Tolosa E, Marti MJ, Valldeoriola F, Bargallo N, et al. Neuroanatomical correlates of impaired decision-making and facial emotion recognition in early Parkinson’s disease. European Journal of Neuroscience. 2009;

22.	Steingroever H, Wetzels R, Horstmann A, Neumann J, Wagenmakers EJ. Performance of healthy participants on the Iowa Gambling Task. Psychol Assess. 2013;25(1):180–93.

23.	Buelow MT, Blaine AL. The assessment of risky decision making: A factor analysis of performance on the Iowa Gambling Task, Balloon Analogue Risk Task, and Columbia Card Task. Psychol Assess. 2015;27(3):777–85.

24.	Lejuez CW, Aklin WM, Jones HA, Richards JB, Strong DR, Kahler CW, et al. The Balloon Analogue Risk Task (BART) Differentiates Smokers and Nonsmokers. Exp Clin Psychopharmacol. 2003;11(1):26–33.

25.	Xu S, Korczykowski M, Zhu S, Rao H. Risk-taking and impulsive behaviors: A comparative assessment of three tasks. Soc Behav Pers. 2013;41(3):477–86.

26.	Tuvblad C, Gao Y, Wang P, Raine A, Botwick T, Baker LA. The genetic and environmental etiology of decision-making: A longitudinal twin study. J Adolesc. 2013;36(2):245–55.

27.	Thiel A, Hilker R, Kessler J, Habedank B, Herholz K, Heiss WD. Activation of basal ganglia loops in idiopathic Parkinson’s disease: a PET study. J Neural Transm (Vienna) [Internet]. 2003 Nov [cited 2025 Mar 20];110(11):1289–301. Available from: https://pubmed.ncbi.nlm.nih.gov/14628193/

28.	Kobayakawa M, Tsuruya N, Kawamura M. Decision-making performance in Parkinson’s disease correlates with lateral orbitofrontal volume. J Neurol Sci [Internet]. 2017 Jan 15 [cited 2025 Mar 20];372:232–8. Available from: https://pubmed.ncbi.nlm.nih.gov/28017221/

29.	Buelow MT, Suhr JA. Construct validity of the Iowa gambling task. Neuropsychol Rev. 2009;19(1):102–14.

30.	Balconi M, Angioletti L, Siri C, Meucci N, Pezzoli G. Gambling behavior in Parkinson’s Disease: Impulsivity, reward mechanism and cortical brain oscillations. Psychiatry Res. 2018;270(March):974–80.

31.	Kobayakawa M, Tsuruya N, Kawamura M. Sensitivity to reward and punishment in Parkinson’s disease: An analysis of behavioral patterns using a modified version of the Iowa gambling task. Parkinsonism Relat Disord. 2010;16(7):453–7.

32.	Rossi M, Gerschcovich ER, de Achaval D, Perez-Lloret S, Cerquetti D, Cammarota A, et al. Decision-making in Parkinson’s disease patients with and without pathological gambling. Eur J Neurol. 2010;17(1):97–102.

33.	Euteneuer F, Schaefer F, Stuermer R, Boucsein W, Timmermann L, Barbe MT, et al. Dissociation of decision-making under ambiguity and decision-making under risk in patients with Parkinson’s disease: A neuropsychological and psychophysiological study. Neuropsychologia. 2009;47(13):2882–90.

34.	Mimura M, Oeda R, Kawamura M. Impaired decision-making in Parkinson’s disease. Parkinsonism Relat Disord. 2006;12(3):169–75.

35.	Oyama G, Shimo Y, Natori S, Nakajima M, Ishii H, Arai H, et al. Acute effects of bilateral subthalamic stimulation on decision-making in Parkinson’s disease. Parkinsonism Relat Disord. 2011;17(3):189–93.

36.	Perretta JG, Pari G, Beninger RJ. Effects of Parkinson disease on two putative nondeclarative learning tasks: probabilistic classification and gambling. Cogn Behav Neurol [Internet]. 2005 Dec [cited 2025 Mar 20];18(4):185–92. Available from: https://pubmed.ncbi.nlm.nih.gov/16340390/

37.	Pagonabarraga J, García-Sánchez C, Llebaria G, Pascual-Sedano B, Gironell A, Kulisevsky J. Controlled study of decision-making and cognitive impairment in Parkinson’s disease. Movement Disorders [Internet]. 2007 Jul 30 [cited 2025 Mar 20];22(10):1430–5. Available from: https://onlinelibrary.wiley.com/doi/full/10.1002/mds.21457

38.	Duckworth AL, Kern ML. A meta-analysis of the convergent validity of self-control measures. J Res Pers. 2011;45(3):259–68.

39.	Overman WH. Sex differences in early childhood, adolescence, and adulthood on cognitive tasks that rely on orbital prefrontal cortex. Brain Cogn. 2004;55(1):134–47.

40.	Bechara A, Dolan S, Denburg N, Hindes A, Anderson SW, Nathan PE. Decision-making deficits, linked to a dysfunctional ventromedial prefrontal cortex, revealed in alcohol and stimulant abusers. Neuropsychologia. 2001;39(4):376–89.

41.	Grant S, Contoreggi C, London ED. Drug abusers show impaired performance in a laboratory test of decision making. Neuropsychologia. 2000;38(8):1180–7.

42.	Brand M, Recknor EC, Grabenhorst F, Bechara A. Decisions under ambiguity and decisions under risk: Correlations with executive functions and comparisons of two different gambling tasks with implicit and explicit rules. J Clin Exp Neuropsychol [Internet]. 2007 Jan 10 [cited 2025 Mar 20];29(1):86–99. Available from: https://www.tandfonline.com/doi/abs/10.1080/13803390500507196

43.	Castrioto A, Funkiewiez A, Debû B, Cools R, Lhommée E, Ardouin C, et al. Iowa gambling task impairment in Parkinson’s disease can be normalised by reduction of dopaminergic medication after subthalamic stimulation. J Neurol Neurosurg Psychiatry. 2015;86(2):186–190.

44.	Waters-Wood SM, Xiao L, Denburg NL, Hernandez M, Bechara A. Failure to learn from repeated mistakes: Persistent decision-making impairment as measured by the iowa gambling task in patients with ventromedial prefrontal cortex lesions. Journal of the International Neuropsychological Society. 2012;18:927–930.

45.	Bechara A, Damasio H, Damasio AR. Emotion, decision making and the orbitofrontal cortex. Cerebral Cortex. 2000;10:295–307.

**Rating test evaluation**

| **Test name: Behavioural Assessment of the dysexecutive Syndrome** | Responses (possible) |
| --- | --- |
| **Version**  Are there several versions (alternate or parallel) of the test? If so, which one has been assessed? | There are no alternate/parallel versions of the full measure.  There is a parallel version of the Modified Six Elements Test, which is a subtest within the BADS^1^. |
| **Description of test**   - What population the test was originally developed for? - Which domains/components of domains do the test assess? - Are there multiple domains or a predominant domain assessed? - Does it score impaired/unimpaired? - Does it score severity   (subtle/mild/moderate/severe)? | The BADS was developed for individuals with Age Range: 16 years to 87 years  BADS specifically assesses the skills and demands involved in everyday life.  BADS includes:  Temporal judgement - This test uses four questions to assess subjects’ ability to estimate how long various complete events (such as a dental appointment) last.  Rule shift cards - Tests the ability to change an established pattern of responding, using familiar materials. In part 1 a response pattern is established according to a simple rule. In part 2 the rule is changed and subjects have to adapt their responses, inhibiting their original response set.  Action program - Tests practical problem solving. A cork has to be extracted from a tall tube, a result which can only be achieved by the planned use of various other materials provided.  Key search - A test of strategy formation. In an analogue of a common problem, subjects are required to demonstrate how they would search a field for a set of lost keys and their strategy is scored according to its functionality.  Zoo map - This is a test of planning. It provides information about subjects’ ability to plan a route to visit six of a possible 12 locations in a zoo, firstly in a demanding, open-ended situation where little external structure is provided, and secondly in a situation that involves simply following a concrete, externally imposed strategy.  Modified six elements - This is a test of planning, task scheduling and performance monitoring. It is a simplified version of the original Shallice Burgess (1991) test. Subjects have to schedule their time to work on six tasks over a ten minute period.  Therefore, the BADS evaluates a predominant domain: executive functions  Behavioral Assessment of the Dysexecutive Syndrome – BADS is composed of six sub-tests, with a maximum score of 24 points. An overall Total Score (TS) for the whole battery is obtained by adding together the individual score for each subtest. The Total Score for each subject is converted into a Standard Score (SS) with a mean of 100 and a standard deviation of 15, and into an Age Standard Score (ASS). An overall classification is obtained: impaired, borderline, low average, average, high aver­age, superior, and very superior.    The BADS does not score severity (subtle/mild/moderate/severe) |
| **Scaling Metric**: Type of response format? (e.g., Likert, Binary, multiple-choice, continuum, reaction time, etc.) | Binary |
| **Respondent: Patient:** any specific requirements for the target study population (e.g. non-demented, motor, auditory, visual abilities, etc)? | Intact or well compensated auditory and visual abilities. Motor impairments may influence the performance on test. |
| **Access:** How can the test be obtained (mail address or website)? | Website of Pearson  The link in the UK is:  <https://www.pearsonclinical.co.uk/Psychology/AdultCognitionNeuropsychologyandLanguage/AdultAttentionExecutiveFunction/BehaviouralAssessmentoftheDysexecutiveSyndrome(BADS)/BehaviouralAssessmentoftheDysexecutiveSyndrome(BADS).aspx>  The link in the USA is:  https://www.pearsonassessments.com/store/usassessments/en/Store/Professional-Assessments/Cognition-%26-Neuro/Behavioural-Assessment-of-the-Dysexecutive-Syndrome/p/100000427.html |
| Copyrighted or in public domain?  Conditions for use? Fee? | Copyrighted, there is a fee.  Condition for use: Qualification Level CL2 in the UK (see the link: <https://www.pearsonclinical.co.uk/information/qualificationcodes.aspx#CL2>); Qualification Level B in the US. |
| **Translations:** Has the test been translated and validated in other languages besides English? | Yes, there are Japanese^2^, Spanish^3^, Italian^4^, and Portuguese^5,6^ versions |

| **Test properties**  (Please provide references for all statements or note if this is your judgment) |  |
| --- | --- |
| Which cognitive domain (or component/specific aspect of a cognitive domain) does this test *primarily* measure? | Executive function;  The battery of tests evaluates executive functions such as: inhibitory control and monitor behavior, planning, priorities, problem solving, and cognitive flexibility. |
| Which aspects of the domain you are assessing are not covered? | Language, Memory, Visuospatial function, |

| **Use** |  |
| --- | --- |
| Was it *designed* to measure severity, screen or diagnosis of the domain? | The BADS measures executive functions.  Wilson affirmed that “The BADS is designed to predict “everyday problems arising from the dysexecutive syndrome (DES)” ^5^. |
| Is there a cut-off score? Are there measures of screening/diagnostic performance of the test? (Receiver characteristic curve analyses; sensitivity/specificity; Positive Predictive Value/Negative Predictive Value), True Positive Rate, False Positive Rate etc.) | Cut-off score in relation to the diagnostic unit (MCI/dementia) ^1,7^ |
| Is this appropriate for the PD population? If not, why (low sensitivity and/or specificity values)? | The use of the BADS in the assessment of Executive dysfunctions in PD without dementia is supported by Perfetti et al. ^8^    The authors stated that “Twenty-five non-demented PD patients and 24 demographically-matched controls were administered BADS and other commonly used executive tools. Comparisons between groups indicated that two of the six BADS subtests (Temporal Judgement and Action Program) did not have sufficient sensitivity to executive impairments. However, when we explored group-predictive capabilities among the tests, the BADS total score was the most sensitive, followed by the Tower of London (TOL). We obtained similar results when we disentangled the sensitivity of the six BADS subtests. The BADS Six Elements task was the best group predictor followed by the TOL. Our findings showed that BADS is more sensitive to executive dysfunction than some of the tools commonly used to assess this construct in PD. However, we also demonstrated that, to assess executive impairments in PD without dementia adequately, this battery should be administered in combination with the TOL” |
| **Clinimetric/psychometric properties in the target population** |  |
| **Feasibility /Acceptability** |  |
| Length | Number of items/domains: 6 tests  Average range in minutes: 40 minutes |
| Ambiguities in instructions to patient | No |
| Ambiguities in rating anchors | No |
| Appropriateness of questions for population | Yes |
| Applicability across disease cognitive stages | not evaluated |
| Applicability across disease motor severity? | the changes in each proﬁle score of the 6  subtests with the progression of H&Y stage.  the changes in each proﬁle score of the 6  subtests with the progression of H&Y stage.  The changes im profile score of the 6 subtests with the progression of H6Y stage were evaluated in Kamei et al., 2008. The profile scores of all 6 subtests decreased with progression of the H&Y stages. However, the patterns of these changes were not uniform among the subcomponents of the executive function. |
| Clear instructions to raters | Yes |
| Has the test been used by researcher other than developers?  By other groups  Not applicable? | Yes ^8,9,10^ |
| Are there instructions for dealing with missing data?  Not applicable? | not evaluated |
| Floor and ceiling effects, skewed score distributions? | not evaluated |

| **Have the component of dimensionality, variability and dependency structures been analyzed?**  (Exploratory and confirmatory factor analysis (EFA and CFA)? | not evaluated |
| --- | --- |
|  |  |
| **Reliability: assessed – good; not good. Not assessed**  Are alternative versions developed?  If so, were they validated with reliability measures? | -not evaluated in PD  Evaluated in AD^11^ |
| Internal consistency (A rule of thumb)  0.9 ≤ Cronbach’s alpha (α) Excellent  0.8 ≤ α < 0.9 Good  0.7 ≤ α < 0.8 Acceptable  0.6 ≤ α < 0.7 Questionable  0.5 ≤ α < 0.6 Poor  α < 0.5 Unacceptable  (Streiner, 2003 Journal of Personality Assessment, 80:1, 99-103, DOI: 10.1207/S15327752JPA8001_18) | not evaluated |
| “Corrected item-to-total correlation” | not evaluated |
| Test/retest | In Wilson et al. ^5^, test-retest reliability was also examined with subjects generally performing slightly higher after the second administration, but the differences were not statistically significant.  Correlations between the first and second test administrations were moderate, with the exception of the Zoo Map Test, where virtually no correlation was found. This was attributed to the presence of outliers and small sample size (*n* = 25). The test-retest reliability of the BADS was similar in pattern to other tests of frontal lobe functioning administered at the same time (e.g., Modified Card Sorting Test; Nelson [1976](https://link.springer.com/referenceworkentry/10.1007%2F978-3-319-57111-9_166#CR2266)).^11^ |
| Inter-rater | The intraclass correlation coefﬁcient for the total pro-  ﬁle scores in the ﬁrst 15 registered patients among the 3  raters was 0.994 (95% CI: 0.985 to 0.998).  in PD patients the intraclass correlation coefficient for the total profile scores in the first 15 registered patients among the 3 raters was 0.994 (95% CI: 0.985 to 0.998).  In non-PD. Wilson et al. ^5^ found that inter-rater reliability was high, ranging from 0.88 to 1.00. |
| Intra-rater | Not evaluated |
| Other (e.g. Standard Error of Measurement) | Not evaluated |
| **Validity: assessed –good; not good. Not assessed (reference)** | Not assessed |
| Face and Content validity (does test appears to be measuring what it is intended to measure and do test’s items comprehensively represent the domain of interest?) | Not assessed |
| Criterion validity (compared to gold-standard) or other criteria for  diagnosis) (e.g. other comparable tests or lesion or MRI research) | In PD, concurrent validity was good^8^ |
| Construct validity (correlations with other convergent tests and divergent tests; known-groups comparisons) | in PD^8^  Convergent validity: Correlation values (Spearman's rho) between the BADS-T and the other traditional indices were also computed. With the exception of the TMT (−0.153, *p* = 0.3) the BADS-T showed moderate correlations (absolute values) ranging from 0.43 to 0.51 (*p* < 0.01).  Divergent validity: not evaluated  Group-comparisons:  BADS composite profile score did differentiate between the PD and control groups (BADS-T, p < 0.001). Nonetheless, only four out of six BADS subtests reached statistical significances in their analysis. Compared to the controls, the PD patients made more errors when asked to shift between two rules (BADS-RS, p < 0.001), they had greater difficulties with the key search test (BADS-KS, p < 0.01), they obtained lower scores on the zoo map (BADS-ZM, p < 0.001) and finally they had more difficulty in managing the Six Elements test demands (BADS-6E, p < 0.001). In contrast, no differences in performance were evident on the action program (BADS-AP) and temporal judgement (BADS-TJ) subtests.  In Participants with and without brain damage, the BADS and most of its subtests correlate significantly with the standard executive tests indicating that it possesses adequate concurrent validity. In terms of construct validity, it is comparable to standard executive tests in discriminating between neurological and non-brain-damaged participants. The ecological validity of the BADS is superior to standard executive tests in terms of predicting competency in role functioning. ^12^ |
| Reference group used to develop this test:   58) If available, please define the characteristic of the norm group adopted (demographic variables used for stratification: age, education, sex, etc). 59) What kind of standardized score generated (T score, Z score, derived IQ score, percentile ranks, etc)? 60) If translated/validated in other languages, are reference norms created? If so, please specify demographic variables used for stratification (age, education, gender etc). | Group characteristics:  Performance on each of the 6 subtests in the BADS Is scored according to the BADS manual. These scores are summed to derive a composite total proﬁle score, providing an overall measure of ExD. The total proﬁle score is then converted into a standardized score with a mean(M) of 100 and a standard deviation (SD) of 15 based on data obtained from normal control subjects in the same manner as for IQ scores on the Wechsler Adult Intelli-gence Scale, Revised.  Since executive function decreases even in normal, aged  subjects, a controlled standardized score is also provided  for the BADS based on data obtained from different age  groups of normal control subjects.  16,2  Since executive function decreases even in normal, aged subjects, a controlled standardized score is also provided for the BADS based on data obtained from different age groups of normal control subjects  Italian version and Italian normative value^4^ |

| **Responsiveness: assessed –good; not good. Not assessed (reference)** | Not assessed |
| --- | --- |
| Demonstrated to be sensitive to change (change over time or due to treatment)*  * [determined by the strength of the relationship between change in the test scores and the criterion or anchor scores] | If Yes, please specify |
| Has the minimal clinically important change and minimal clinically relevant incremental difference been assessed?  (besides a statistically significant improvement) | Minimal clinically important change assessed Y/N? |
| Has this test been assessed or used in patients with other condition than that of interest? | Y/N, please specify if one or several condition |
| Other |  |
| **Overall impression** |  |
| Strengths | The battery focuses on revealing dysexecutive syndromes which are part of PD; the measure has been used in PD and in a variety of other disorders affecting frontal systems. |
| Weaknesses | The clinicometric properties of the battery are poorly evaluated and deserve to be investigated (i.e. responsiveness, different types of validity) |

| **Level of Recommendation** |  |
| --- | --- |
| “Recommended” – The test was applied to the target population, AND was studied clinimetrically and found to be valid, reliable and sensitive to change, AND was used in studies by researchers, others than the developers  “Recommended with caveats” – Test’s properties were generally found to be adequate, but some of the measurement properties were not evaluated or not evaluated specifically in a PD cohort."  ‘Suggested’ – The test was applied to the target population, but only one of the other criteria applies  ‘Listed” – The test was applied to the target population, but none of the other criteria applies | “Suggested” – Test’s properties were generally found to be adequate, but some of the measurement properties were not evaluated or not evaluated specifically in a PD cohort." |
| Which type of study is this test suitable for and which type of study is it not suitable for (screening, prevalence, etiological (e.g. case-control or genetic), treatment trial, correlation with biological markers or other scales, e.g. of parkinsonism, clinical practice for diagnosis/ screening). List all. | Suitable for:  - clinical practice screening, screening  for research.  - Prevalence studies  - Correlation with other scales  Not suitable:  -treatment trials  - Unless more studies are available that determine minimal clinically important change) |

**REFERENCES**

(1) Bertens D, Fasotti L, Egger JI, Boelen DH, Kessels RP. Reliability of an Adapted Version of the Modified Six Elements Test as a Measure of Executive Function. Appl Neuropsychol Adult. 2016;23(1):35-42. doi: 10.1080/23279095.2015.1012258. Epub 2015 Jun 25. PMID: 26111243.
(2) Kashima H., Mimura M., Tabuchi H., Moriyama Y., Kato M. Behavioural Assessment of the Dysexecutive Syndrome. Tokyo, Japan: Shinkoh Igakushuppan; 2003. (Japanese)
(3) Pedrero Pérez EJ, Ruiz Sánchez de León JM, Rojo Mota G, Llanero Luque M, Olivar Arroyo A, Bouso Saiz JC, Puerta García C. Versión española del Cuestionario Disejecutivo (DEX-Sp): propiedades psicométricas en adictos y población no clínica [Spanish version of the Dysexecutive Questionnaire (DEX-Sp): psychometric properties in addicts and non-clinical sample]. Adicciones. 2009;21(2):155-66. Spanish. PMID: 19578733.
(4) Antonucci, G; Spitoni, G; Orsini, A; D'Olimpio, Francesca; Cantagallo, A. Taratura Italiana della Batteria per la valutazione della Sindrome Disesecutiva: BADS. GIUNTI O.S. Organizzazioni Speciali. Firenze, 2014.
(5) Wilson BA, Alderman N, Burguess PW, Emslie JJ. Manual of the behavioral assessment of the dysexecutive syndrome. Bury St. Edmunds U.K.: Thames Valley Test Company Trans: Ricardo O. Souza, Sergio L. Schmidt. Rio de Janeiro: Cognicao; 1996.
(6) Canali, F., Brucki, S.M.D., Berolucci, P.H.F., Bueno, O.F.A. Reliability study of the Behavioral Assessment of the Dysexecutive Syndrome adapted for a Brazilian sample of older-adult controls and probable early Alzheimer’s disease patients. Revista brasileira de psiquiatria. 2011; 33(4), 338-346.
(7) Emmanouel A, Kessels RP, Mouza E, Fasotti L. Sensitivity, specificity and predictive value of the BADS to anterior executive dysfunction. Neuropsychol Rehabil. 2014;24(1):1-25. doi: 10.1080/09602011.2013.863731. Epub 2013 Dec 19. PMID: 24354937.
(8) Perfetti B, Varanese S, Mercuri P, Mancino E, Saggino A, Onofrj M. Behavioural assessment of dysexecutive syndrome in Parkinson's disease without dementia: a comparison with other clinical executive tasks. Parkinsonism Relat Disord. 2010 Jan;16(1):46-50. doi: 10.1016/j.parkreldis.2009.07.011. Epub 2009 Aug 7. PMID: 19665420.
(9) Kamei S, Hara M, Serizawa K, Murakami M, Mizutani T, Ishiburo M, Kawahara R, Takagi Y, Ogawa K, Yoshihashi H, Shinbo S, Suzuki Y, Yamaguchi M, Morita A, Takeshita J, Hirayanagi K. Executive dysfunction using behavioral assessment of the dysexecutive syndrome in Parkinson's disease. Mov Disord. 2008 Mar 15;23(4):566-73. doi: 10.1002/mds.21890. PMID: 18098279.
(10) Ninomiya, S., Morita, A., Teramoto, H., Akimoto, T, Shiota, H., Kamei, S. Relationship between postural deformities and frontal function in Parkinson’s disease. Parkinson’s Disease. 2015 462143. doi:10.1155/2015/462143
(11) Nelson HE. A modified card sorting test sensitive to frontal lobe defects. Cortex. 1976 Dec;12(4):313-24. doi: 10.1016/s0010-9452(76)80035-4. PMID: 1009768.
(12) Gus Norris & Robyn L. Tate (2000) The Behavioural Assessment of the Dysexecutive Syndrome (BADS): Ecological, Concurrent and Construct Validity, Neuropsychological Rehabilitation, 10:1, 33-45, DOI: [10.1080/096020100389282](https://doi.org/10.1080/096020100389282)

Other references in schizophrenia

(13) Katz N, Tadmor I, Felzen B, Hartman-Maeir A. The Behavioural Assessment of the Dysexecutive Syndrome (BADS) in schizophrenia and its relation to functional outcomes. Neuropsychol Rehabil. 2007 Apr;17(2):192-205. doi: 10.1080/09602010600685053. PMID: 17454693.
(14) Krabbendam L, de Vugt ME, Derix MM, Jolles J. The behavioural assessment of the dysexecutive syndrome as a tool to assess executive functions in schizophrenia. Clin Neuropsychol. 1999 Aug;13(3):370-5. doi: 10.1076/clin.13.3.370.1739. PMID: 10726608.
(15) Thai ML, Andreassen AK, Bliksted V. A meta-analysis of executive dysfunction in patients with schizophrenia: Different degree of impairment in the ecological subdomains of the Behavioural Assessment of the Dysexecutive Syndrome. Psychiatry Res. 2019 Feb;272:230-236. doi: 10.1016/j.psychres.2018.12.088. Epub 2018 Dec 20. PMID: 30590277.
(16) Evans JJ, Chua SE, McKenna PJ, Wilson BA. Assessment of the dysexecutive syndrome in schizophrenia. Psychol Med. 1997 May;27(3):635-46. doi: 10.1017/s0033291797004790. PMID: 9153684.
(17) Jelicic M, Henquet CE, Derix MM, Jolles J. Test-retest stability of the behavioural assessment of the dysexecutive syndrome in a sample of psychiatric patients. Int J Neurosci. 2001 Sep;110(1-2):73-8. doi: 10.3109/00207450108994222. PMID: 11697212.

Other references in traumatic brain injury

(18) Bennett PC, Ong B, Ponsford J. Assessment of executive dysfunction following traumatic brain injury: comparison of the BADS with other clinical neuropsychological measures. J Int Neuropsychol Soc. 2005 Sep;11(5):606-13. doi: 10.1017/S1355617705050721. PMID: 16212688.
(19) Sawamura, D., Ikoma, K., Ogawa, K., Sakai, S. Clinical utility of neuropsychological tests for employment outcomes in persons with cognitive impairment after moderate to severe traumatic brain injury. Brain Injury, 32: 13-14, 1670-1677.
(20) Shinagawa Y, Nakaaki S, Hongo J, Murata Y, Sato J, Matsui T, Tatsumi H, Akechi T, Furukawa TA. Reliability and validity of the Japanese version of the Dysexecutive Questionnaire (DEX) in Alzheimer's disease: validation of a behavioral rating scale to assess dysexecutive symptoms in Japanese patients with Alzheimer's disease. Int J Geriatr Psychiatry. 2007 Oct;22(10):951-6. doi: 10.1002/gps.1768. PMID: 17299806.

References for use in other disorders:

(21) Moriyami, Y., Mimura, M., Kato ,M., Yoshino, A., Hara, T., Kashima, H., Kato, A., Watanabe, A. Executive dysfunction and clinical outcome in chronic alcoholics. Alcoholism: Clinical and Experimental Research. 2002. 26: 8, 1239:1244
(22) Raczka, K.A., Becker, G., Seese, A., Frisch, S., Heiner, S., Marschhauser, A., Barthel, H, Scheid, R. Sabri, O., Schroeter, M. Executive and behavioral deficits share common neural substrates in frontotemporal lobar degeneration – A pilot FDG-PET study. Psychiatry Research: Neuroimaging. 2010. 182: 274-280.
(23) Spitoni, G.F., Aragonaa, M., Bevacqua, S., Cotugno, A. An ecological approach to the behavioral assessment of executive functions in anorexia nervosa. Psychiatry Research. 2018. 259: 283-288.

**Rating test evaluation**

| **Test name and Assessor:**  Burgess, P. & Shallice, T. (1997) *The Hayling and Brixton Tests. Test manual.* Bury St Edmunds, UK: Thames Valley Test Company. | Responses (possible) |
| --- | --- |
| **Version**  Are there several versions (alternate or parallel) of the test?  If so, which one has been assessed? | There is only one version (publication date 1997). |
| **Description of test**   - What population was the test originally developed for? - Which domains/components of domains do the test assess? - Are there multiple domains or a predominant domain assessed? - Does it score impaired/unimpaired? - Does it score severity   (subtle/mild/moderate/severe)? | - Anterior cortical lesion patients - Executive functioning   The Hayling Sentence Completion Test evaluates both response initiation and suppression. It consists of two sections, each featuring 15 sentences with the final word omitted. In the first section, participants complete the sentences to gauge their response speed. The second section requires participants to finish sentences with nonsensical words while suppressing appropriate ones, allowing for the assessment of their response suppression abilities and cognitive processing time.   - Yes - Yes |
| **Scaling Metric**: Type of response format? (e.g., Likert, Binary, multiple-choice, continuum, reaction time, etc.) | Response time and error rates. |
| **Respondent: Patient:** any specific requirements for the target study population (e.g. non-demented, motor, auditory, visual abilities, etc)? | Intact language: Comprehension and production. |
| **Access:** How can the test be obtained (mail address or website)? | Pearson Assessment https://www.pearsonclinical.co.uk/Psychology/AdultCognitionNeuropsychologyandLanguage/AdultAttentionExecutiveFunction/HaylingandBrixtonTests/HaylingandBrixtonTests.aspx |
| Copyrighted or in public domain?  Conditions for use? Fee? | Copyrighted  £230.73 inc VAT (includes the Rixton Test, the Hayling tests cannot be purchased separately). |
| **Translations:** Has the test been translated and validated in other languages besides English? | Yes: most European languages, Chinese and Arabic. |

| **Test properties**  (Please provide references for all statements or note if this is your judgment) |  |
| --- | --- |
| Which cognitive domain (or component/specific aspect of a cognitive domain) does this test *primarily* measure? | Response initiation and response suppression.  See Test manual |
| Which aspects of the domain you are assessing are not covered? |  |

| **Use** |  |
| --- | --- |
| Was it *designed* to measure severity, screen or diagnosis of the domain? | Severity  See Response sheet |
| Is there a cut-off score? Are there measures of screening/diagnostic performance of the test? (Receiver characteristic curve analyses; sensitivity/specificity; Positive Predictive Value/Negative Predictive Value), True Positive Rate, False Positive Rate etc.) | YES  See Response sheet |
| Is this appropriate for the PD population? If not, why (low sensitivity and/or specificity values)? | YES  Siquier A, Andrés P. Cognitive and Behavioral Inhibition Deficits in Parkinson’s Disease: The Hayling Test as a Reliable Marker. *Frontiers in Aging Neuroscience.* 2021 Jan 15;12. [*https://doi.org/10.3389/fnagi.2020.621603*](https://doi.org/10.3389/fnagi.2020.621603) |
| **Clinimetric/psychometric properties in the target population** |  |
| **Feasibility /Acceptability** |  |
| Length | 5 minutes |
| Ambiguities in instructions to patient | N/A |
| Ambiguities in rating anchors | NO |
| Appropriateness of questions for population | YES |
| Applicability across disease cognitive stages | YES |
| Applicability across disease motor severity? | YES |
| Clear instructions to raters | YES |
| Has the test been used by researcher other than developers? | YES |
| Are there instructions for dealing with missing data? | NO |
| Floor and ceiling effects, skewed score distributions? | NO |

| **Have the components of dimensionality, variability and dependency structures been analyzed?** | NO |
| --- | --- |
|  |  |
| **Reliability: assessed – good; not good. Not assessed** | Low (see Manual, section 4.1 Split-half reliability) |
| Internal consistency (A rule of thumb) | Internal Consistency: (Burgess & Shallice, 1997). Split-half reliability  coefficients were variable for healthy adults (.35-.83) but respectable for  patients with anterior lesions (.72-.93). |
| “Corrected item-to-total correlation” |  |
| Test/retest | High: (Burgess & Shallice, 1997). Reliabilities assessed in  31 healthy adults between 2 days-4 weeks following the first assessment  were adequate for the overall score (.76) and Section 2 time (.78), but  weaker for other scores, Section 1 time (.62) and Section 2 errors (.52). |
| Inter-rater | No information regarding inter-rater reliability is provided in the test manual, even though significant judgment is required to assign responses to categories. Andres and Van der Linden (2000) reported that two raters agreed on only 76.5% of 1425 responses.  Andres P, Van der Linden M. Age-Related Differences in Supervisory Attentional System Functions. *The Journals of Gerontology Series B: Psychological Sciences and Social Sciences*. 2000 Nov 1;55(6):P373–80. |
| Intra-rater | NO information available. |
| Other (e.g. Standard Error of Measurement) |  |
| **Validity: assessed –good; not good. Not assessed (reference)** |  |
| Face and Content validity (does test appears to be measuring what it is intended to measure and do test’s items comprehensively represent the domain of interest?) | Concurrent Validity: Burgess and Shallice (1997) found that patients with lesions including the frontal cortex were slower and made more errors than controls and patients with posterior lesions. PET in healthy individuals showed activation of left frontal regions and right anterior cingulate during sentence suppression (part 2) (Nathaniel-James et al., 1997). However, there is evidence for activation in other areas and equivalent performance in individuals with discrete frontal lobe lesions (see Strauss, Sherman & Spreen for overview). Performance is impaired in a variety of conditions that disrupt EF, including schizophrenia, Parkinson’s disease, Alzheimer’s disease, alcoholism and ADHD (see Strauss, Sherman and Spreen (2006) for overview).  Nathaniel-James DA, Fletcher P, Frith CD. The functional anatomy of verbal initiation and suppression using the Hayling Test. *Neuropsychologia*. 1997;35(4):559-566. doi:10.1016/s0028-3932(96)00104-2.  Strauss E, Sherman EMS, Otfried Spreen. A compendium of neuropsychological tests: administration, norms, and commentary. New York, N.Y.: Oxford University Press; 2006. |
| Criterion validity (compared to gold-standard) or other criteria for diagnosis) (e.g. other comparable tests or lesion or MRI research) | There is no gold standard for assessment of executive functions |
| Construct validity (correlations with other convergent tests and divergent tests; known-groups comparisons) | The Hayling Test shows moderate correlations with other measures of EF including the Six Elements Test (.40-.65; Clark et al., 2000) and the Tower of London (.40-.64; Andres & Van der Linden, 2000; Marczewski et al., 2001). Bajo and Nathaniel-James (2001) assessed 48 persons with moderate to severe traumatic brain injury. Hayling part 1 (initiation) correlated with all three DEX factors (response suppression, intentionality, and executive memory) although only weakly (.21–.28). Hayling part 2 (response suppression) did not correlate with scores on any of the three factors  Clark C, Prior M, Kinsella GJ. Do Executive Function Deficits Differentiate Between Adolescents with ADHD and Oppositional Defiant/Conduct Disorder? A Neuropsychological Study Using the Six Elements Test and Hayling Sentence Completion Test. Journal of Abnormal Child Psychology. 2000;28(5):403–14.  Marczewski P, de Linden MV, Lar⊘i F. Further investigation of the Supervisory Attentional System in schizophrenia: Planning, inhibition, and rule abstraction. Cognitive Neuropsychiatry. 2001 Aug;6(3):175–92.  Bajo A, Nathaniel-James D. The Hayling and Brixton Tests of Dysexecutive Syndrome. What do they measure in everyday life? In: 4th World Congress on Brain Injury. 2001. |
| Reference group used to develop this test:   61) If available, please define the characteristic of the norm group adopted (demographic variables used for stratification: age, education, sex, etc). 62) What kind of standardized score generated (T score, Z score, derived IQ score, percentile ranks, etc)? 63) If translated /validated in other languages, are reference norms created? If so, please specify demographic variables used for stratification (age, education, gender etc). | 1). The published test provides normative data for 118 individuals stratified by age and IQ.  Characteristics of the normative sample; 61 F and 57 M  Age distribution: 18-45, n=48; 46-65, n=51; 66-80, n=19  2). Scaled scores running from 1 to 10.  3). Normative data available in most languages  Spanish  Normative data stratified by age and education  <https://academic.oup.com/acn/article/31/5/411/2364792>  Italian  Normative data stratified by age  https://academic.oup.com/acn/article/33/4/466/4091076  French  Normative data stratified by age and education  <https://www.semanticscholar.org/paper/French-Version-of-the-Hayling-Sentence-Completion-Bayard-G%C3%A9ly-Nargeot/8f5bcd13d24e2040214d65dd907aebe5394e53f0>  Brazilian  Normative data stratified by age and education  DOI: 10.1590/2237-6089-2016-0082  Arabic  Normative data stratified by age  <https://head-face-med.biomedcentral.com/articles/10.1186/s13005-020-00251-1>  Chinese  Normative data stratified by age and education  https://bpspsychub.onlinelibrary.wiley.com/doi/abs/10.1348/174866410X516803 |

| **Responsiveness: assessed –good; not good. Not assessed (reference)** |  |
| --- | --- |
| Demonstrated to be sensitive to change (change over time or due to treatment)*  * [determined by the strength of the relationship between change in the test scores and the criterion or anchor scores] | The Hayling Test shows sensitivity to and specificity for frontal lobe damage and our findings suggest that the right lateral frontal region is crucial for verbal response suppression and strategy generation and use.  Robinson GA, Cipolotti L, Walker DG, Biggs V, Bozzali M, Shallice T. Verbal suppression and strategy use: a role for the right lateral prefrontal cortex? Brain. 2015 Feb 9;138(4):1084–96. |
| Has the minimal clinically important change and minimal clinically relevant incremental difference been assessed?  (besides a statistically significant improvement) | NO |
| Has this test been assessed or used in patients with other condition than that of interest? | YES: Neurological, neurodegenerative, psychiatric |
| Other |  |
| **Overall impression** |  |
| Strengths | Quick to administer and easy to score.  Entirely spoken therefore suitable for patients with reading and movement impairment. Reasonably well priced.  Well recognized.  Less chance of ceiling effects than tasks like the BADS. |
| Weaknesses | No evidence for inter-rater reliability despite significant judgment required for scoring.  Variable concurrent validity.  Modest normative sample |

| **Level of Recommendation** |  |
| --- | --- |
| “Recommended” – The test was applied to the target population, AND was studied clinimetrically and found to be valid, reliable and sensitive to change, AND was used in studies by researchers, others than the developers  “Recommended with caveats” – Test’s properties were generally found to be adequate, but some of the measurement properties were not evaluated or not evaluated specifically in a PD cohort."  ‘Suggested’ – The test was applied to the target population, but only one of the other criteria applies  ‘Listed” – The test was applied to the target population, but none of the other criteria applies | Please choose one of these options  “Suggested” |

**Rating test evaluation**

| **Test name:** | Brixton spatial anticipation test |
| --- | --- |
| Version  Are there several versions (alternate or parallel) of the test? If so, which one has been assessed? | There is only one version of the test. (1) |
| **Description of test**   - What population the test was originally developed for? - Which domains/components of domains do the test assess? - Are there multiple domains or a predominant domain assessed? - Does it score impaired/unimpaired? - Does it score severity   (subtle/mild/moderate/severe)? | - It has been developed for people with specific frontal lobe damage. - It assesses executive function, in particular rule detection in sequence of stimuli. - There are not multiple domains. - Yes, the scale scores impaired/unimpaired. - The raw score (total number of errors) is converted in scaled score which indicates severity (i.e., very superior, superior, good, high average, average, moderate average, low average, poor, abnormal, impaired). Only accuracy, not response latency, is recorded for the Brixton. |
| **Scaling Metric**: Type of response format? (e.g., Likert, Binary, multiple-choice, continuum, reaction time, etc.) | Multiple-choice; responses 1 to 10 are allowed. Number 1 to 10 refers to the position of the blue dot in the following page. |
| **Respondent: Patient:** any specific requirements for the target study population (e.g. non-demented, motor, auditory, visual abilities, etc)? | It requires spared visuospatial abilities.  It does not require a verbal response therefore it is appropriate for those suffering a wide range of deficits such as those involving speech production or reading.  It can be administered either verbally or manually by pointing. |
| **Access:** How can the test be obtained (mail address or website)? | It can be purchased at the web site: <https://www.pearsonclinical.co.uk/Psychology/AdultCognitionNeuropsychologyandLanguage/AdultAttentionExecutiveFunction/HaylingandBrixtonTests/HaylingandBrixtonTests.aspx> |
| Copyrighted or in public domain?  Conditions for use? Fee? | The material is copyrighted. To be used, the test has to be bought in the website. The complete kit, which includes manual, stimulus book and a pack of 25 scoring sheets costs £230.73 inc VAT.  The test is provided together with the Hayling sentence completion test. |
| **Translations:** Has the test been translated and validated in other languages besides English? | The task does not include verbal stimuli; therefore, no translation is needed.  It has been validated in the UK (1), Canadian (2), Dutch (3) and Italian (4) populations. |

| **Test properties**  (Please provide references for all statements or note if this is your judgment) |  |
| --- | --- |
| Which cognitive domain (or component/specific aspect of a cognitive domain) does this test *primarily* measure? | Executive function.  According to the manual, it is “a concept (or ‘rule’) attainment test” (1).  However, it also requires mental flexibility, set-shifting and working memory abilities (5). |
| Which aspects of the domain you are assessing are not covered? | Not all executive function abilities are specifically investigated. |

| **Use** |  |
| --- | --- |
| Was it *designed* to measure severity, screen or diagnosis of the domain? | It has been designed to measure severity of impairment in concept attainment (specific aspect of executive function). The raw score is converted in scale score which indicates severity (i.e., very superior, superior, good, high average, average, moderate average, low average, poor, abnormal, impaired). |
| Is there a cut-off score? Are there measures of screening/diagnostic performance of the test? (Receiver characteristic curve analyses; sensitivity/specificity; Positive Predictive Value/Negative Predictive Value), True Positive Rate, False Positive Rate etc.) | There is not a cut-off score in relation to the diagnostic unit (MCI/dementia). |
| Is this appropriate for the PD population? If not, why (low sensitivity and/or specificity values)? | There is not a cut-off score in relation to the diagnostic unit (MCI/dementia) in PD population. |
| **Clinimetric/psychometric properties in the target population** |  |
| **Feasibility /Acceptability** |  |
| Length | It includes 55 items.  According to the Pearson website, it takes between 5 to 10 minutes to give. |
| Ambiguities in instructions to patient | No (personal judgment). |
| Ambiguities in rating anchors | No. |
| Appropriateness of questions for population | Yes (personal judgment). |
| Applicability across disease cognitive stages | There are no studies investigating feasibility of the test across PD cognitive stages.  However, the test has been used in PD-MCI patients (6–8).  No studies using the test in PDD have been found. However, it has been used in other populations with dementia such as AD (9,10), behavioural variant frontotemporal dementia and semantic dementia (11). |
| Applicability across disease motor severity? | It can be administered also in patients with H&Y score >4 [for example, see (12)], as responses can be provided either verbally or manually by pointing.  According to the manual (1), it can also be administered whilst sitting alongside the subject as in the case of bed-side administration. |
| Clear instructions to raters | Yes (personal statement). |
| Has the test been used by researcher other than developers?  By other groups  Not applicable? | Yes. It has been used by the developers and by many other groups. |
| Are there instructions for dealing with missing data?  Not applicable? | There are no instructions for dealing with missing data. Participants have to provide their answer in order to continue with the task. No total score can be calculated if there are missing data (personal judgement). |
| Floor and ceiling effects, skewed score distributions? | Not found in PD. |

| **Have the component of dimensionality, variability and dependency structures been analyzed?**  (Exploratory and confirmatory factor analysis (EFA and CFA)? | Not found in PD.  In healthy older adults, confirmatory factor analyses were used to test the underlying structure of four indicators of executive functioning. A two-factor model of Inhibition (i.e., Hayling overall scaled score, Stroop Interference) and Shifting (i.e., Brixton overall scaled score, CTT-2 latency) proved to be a poor fit to the data. An alternative model that specified a single executive factor fit the data well (13). |
| --- | --- |
|  |  |
| **Reliability: assessed – good; not good. Not assessed**  Are alternative versions developed?  If so, were they validated with reliability measures? | Reliability characteristics have not been directly assessed in PD but rather in healthy older adults (1,3,14).  There are no alternative versions of the test. |
| Internal consistency (A rule of thumb)  0.9 ≤ Cronbach’s alpha (α) Excellent  0.8 ≤ α < 0.9 Good  0.7 ≤ α < 0.8 Acceptable  0.6 ≤ α < 0.7 Questionable  0.5 ≤ α < 0.6 Poor  α < 0.5 Unacceptable  (Streiner, 2003 Journal of Personality Assessment, 80:1, 99-103, DOI: 10.1207/S15327752JPA8001_18) | Internal consistency (split half reliability) was found to be 0.62 (*p*<0.001) (1). |
| “Corrected item-to-total correlation” | Not assessed. |
| Test/retest | Overall test-retest reliability was r = 0.71 (*p*<0.001) (1).  Test-retest reliability was r = 0.61 (3).  Three-year retest reliability was r = 0.35 (14). |
| Inter-rater | Not assessed. |
| Intra-rater | Not assessed. |
| Other (e.g. Standard Error of Measurement) | Not assessed. |
| **Validity: assessed –good; not good. Not assessed (reference)** |  |
| Face and Content validity (does test appears to be measuring what it is intended to measure and do test’s items comprehensively represent the domain of interest?) | Not assessed. |
| Criterion validity (compared to gold-standard) or other criteria for  diagnosis) (e.g. other comparable tests or lesion or MRI research) | Specific data not available for PD/PD-MCI/PDD. However, there were no differences in Brixton between healthy controls and PD-MCI or PD-MCI and +aMCI (Cammisuli & Sportiello 2017). Nagano Saito (2014) stated that PD-MCI patients performed significantly worse on Brixton than non-MCI patients, but not data was reported. Finally, no differences were observed between PD freezers and nonfreezers.(Nieuwboer et al 2009).  ROC analyses:  Korsakoff’s syndrome versus healthy controls (AUC = 0.74 (0.66 – 0.82)) (3).  Stroke patients versus healthy controls (AUC = 0.56 (0.50 – 0.63)) (3).  Patients with MCI/early dementia showed clear impairments on the RAVLT compared to the controls and most patient groups, they did not show worse performance on the Brixton test (3).  Brixton raw score between the frontal cortical lesion group (M = 17.24, SD = 5.31) and the subcortical lesion group (M = 22.35, SD = 7.53) yielded significantly poorer Brixton scores for patients with subcortical lesions, *t*(55) = –2.54, *p* = .01 (5).  In frontotemporal dementia, Behavioural variant (bvFTD) group performed significantly more poorly (U = 348, p < .0001, r = .58), 53% of patients scoring in the poor-impaired range compared with 6% of semantic dementia (SD) patients. Whereas bvFTD patients showed problems in rule acquisition and switching, SD patients did not, despite their impaired conceptual knowledge (15) |
| Construct validity (correlations with other convergent tests and divergent tests; known-groups comparisons) | In PD samples:   - In PD-MCI, no correlation between the Brixton and Digit Span test, the Brixton and the Stroop test (both time and errors) (6).   In healthy older adults:   - In healthy older adults, the Brixton correlates with the Hayling Sentence Completion Test (r = 0.12, *p*<0.05) and the Color Trail Test part 2 (r = 0.21, *p*<0.001) (13). - In healthy older adults, Brixton (number of errors) correlates with the NART-IQ (r = –.22, *p*<.001) (3). - In healthy older adults, no correlation between the Brixton and the Stroop test (13,14).   In non-PD clinical populations:   - In stroke patients, Brixton age-corrected T-scores correlated with T-scores on the Trail Making Test Part A (r = .48, *p* = .005), Trail Making Test Part B, (r = .52, *p* = .007), RBANS Attention Index (r = .40, *p* = .03), RBANS Visuoconstruction Index (r = .48, *p* = .005), and RBANS Delayed Memory Index (r = .40, *p* = .02), RBANS Figure Copy (r = .50, *p* = .004), Digit Span (r = .43, *p* = .007), and Figure Free Recall (r = .56, *p* = .001) subtests (5). - In patients with schizophrenia, the Brixton total error scores correlated with Tower of London test (r=.52, *p* < .05) as well as with the Hayling sentence Completion Test (r=.68, *p* < .01) (16). - In patients with eating disorders Brixton loaded on the same factor as Trail B, named “simple alternation” in contrast to mental flexibility, perseveration or perceptual shift (17) |
| Reference group used to develop this test:   64) If available, please define the characteristic of the norm group adopted (demographic variables used for stratification: age, education, sex, etc). 65) What kind of standardized score generated (T score, Z score, derived IQ score, percentile ranks, etc)? 66) If translated/validated in other languages, are reference norms created? If so, please specify demographic variables used for stratification (age, education, gender etc). | Group characteristics (Burgess & Shallice, 1997 - manual (1)):  1) Control group: average age in years (M: 45.6, SD: 17.8, range:18-80)  The average age in years (M, SD, range)  No information about education is provided, however the manual states that: “the control group consisted of a rather greater number of people with NART WAIS-R FSIQ equivalent score of 100 or above than would actually appear in the general population (M: 110.9, SD: 12.0)  Sex (67 female, 54 male)  UK, London  Data from Canadian (18), Dutch (19) and Italian (20) population is also available  Lesion groups:  40 individuals with anterior brain lesion (age: M = 45.1, SD = 14.1), 24 individuals with posterior brain lesion (age: M = 43.0, SD = 15.2), 13 individuals with anterior brain lesion (age: M = 53.5, SD = 13.6). All were for patients referred for neuropsychological assessment at the Department of Clinical Neuropsychology at the National Hospital for Neurology and Neurosurgery, London.  2) Scaled score  3) No |

| **Responsiveness: assessed –good; not good. Not assessed (reference)** |  |
| --- | --- |
| Demonstrated to be sensitive to change (change over time or due to treatment)*  * [determined by the strength of the relationship between change in the test scores and the criterion or anchor scores] | Not found for PD/PDD.  In PSP, after a mean interval of 1.2 years, the mean change in score is very close to zero (0.1 points) (21).  In anorexia nervosa, there was a significant improvement on the Brixton test of set-shifting after individual cognitive remediation therapy [F(141) = 54.79, p< 0.001, P(AH) = 1.00; start of treatment: M= 13.34 (SD = 6.01); end of treatment: M= 9.16 (SD = 5.54)],(Leppanen et al 2018)  In early AD patients, after cognitive training, repeated measures general linear model considering groups' performance at the three assessment points (before training, after training and at the 6-month follow-up) showed a significant interaction effect for Brixton test (F(2,74) = 8.783, p < 0.001), showing better performance in experimental group than in controls (22) |
| Has the minimal clinically important change and minimal clinically relevant incremental difference been assessed?  (besides a statistically significant improvement) | Not assessed. |
| Has this test been assessed or used in patients with other condition than that of interest? | It has been used in PD e.g.: (12,23–29), dystonia (30) PD-MCI (6–8).  It has also been used in other populations e.g., progressive supranuclear palsy (21), stroke patients (5), psychopathy (31), anorexia nervosa (17,32,33), schizophrenia (34) healthy older adults (2,3,13), brain lesion (35,36), frontotemporal dementia (11), or early stage Alzheimer disease (22) |
| Other |  |
| **Overall impression** |  |
| Strengths | It is easy to be administered, scored and interpreted. Easier than similar measures of rule attainment (e.g., WCST). It is fast to be completed (5-10 minutes).  It can be administered also in bed. It does not require a verbal or complex motor response by patients, making suitable also for being used with PD patients H&Y > 4. |
| Weaknesses | Standard scores in the manual are not specific to age groups, as scores are not corrected for demographic factors (e.g., age, education level).  For the Brixton a significant effect of age on the number of errors has been reported ((37); (20)). Some specific validations included age range in the norms.  Although it has been used in PD, data about reliability, validity and responsiveness are not available for this population. |

| **Level of Recommendation** |  |
| --- | --- |
| “Recommended” – The test was applied to the target population, AND was studied clinimetrically and found to be valid, reliable and sensitive to change, AND was used in studies by researchers, others than the developers  “Recommended with caveats” – Test’s properties were generally found to be adequate, but some of the measurement properties were not evaluated or not evaluated specifically in a PD cohort."  ‘Suggested’ – The test was applied to the target population, but only one of the other criteria applies  ‘Listed” – The test was applied to the target population, but none of the other criteria applies | “Suggested” - the test was applied to the target population AND it was used in studies by researchers other than the developers. However, it has NOT been studied clinimetrically in PD. |
| Which type of study is this test suitable for and which type of study is it not suitable for (screening, prevalence, etiological (e.g. case-control or genetic), treatment trial, correlation with biological markers or other scales, e.g. of parkinsonism, clinical practice for diagnosis/ screening). List all. | Suitable for case-control studies (between group differences).  Not clear whether it may be suitable for treatment trials as data on minimal clinically important change and minimal clinically relevant incremental difference in PD (or in other populations) has not been assessed.  Not suitable for screening or prevalence of MCI/dementia. |

**REFERENCES**

1.	Burgess PW, Shallice T. The Hayling and Brixton tests. Bury St Edmunds, UK: Thames Valley Test Company Limited. 1997;

2.	Bielak AAM, Mansueti L, Strauss E, Dixon RA. Performance on the Hayling and Brixton tests in older adults: Norms and correlates. Archives of Clinical Neuropsychology. 2006;21(2):141–9.

3.	Van Den Berg E, Nys GMS, Brands AMA, Ruis C, Van Zandvoort MJE, Kessels RPC. The Brixton spatial anticipation test as a test for executive function: Validity in patient groups and norms for older adults. Journal of the International Neuropsychological Society. 2009;15(5):695–703.

4.	Spitoni GF, Bevacqua S, Cerini C, Ciurli P, Piccardi L, Guariglia P, et al. Normative data for the hayling and brixton tests in an Italian population. Archives of Clinical Neuropsychology. 2018;33(4):466–76.

5.	Vordenberg JA, Barrett JJ, Doninger NA, Contardo CP, Ozoude KA. Application of the brixton spatial anticipation test in stroke: Ecological validity and performance characteristics. Clinical Neuropsychologist. 2014;28(2):300–16.

6.	Cammisuli DM, Sportiello MT. Conscious attention defect and inhibitory control deficit in Parkinson’s disease-mild cognitive impairment: A comparison study with amnestic mild cognitive impairment multiple domain. Psychiatr Danub. 2017;29(4):437–45.

7.	Nagano-Saito A, Habak C, Mejía-Constaín B, Degroot C, Monetta L, Jubault T, et al. Effect of mild cognitive impairment on the patterns of neural activity in early parkinson’s disease. Neurobiol Aging. 2014;35(1):223–31.

8.	Yágüez L, Costello A, Moriarty J, Hulse N, Selway R, Clough C, et al. Cognitive predictors of cognitive change following bilateral subthalamic nucleus deep brain stimulation in Parkinson’s disease. Journal of Clinical Neuroscience. 2014;21(3):445–50.

9.	Buhl C, Stokholm J, Gade A. Clinical Utility of Short Social Cognitive Tests in Early Differentiation of Behavioral Variant Frontotemporal Dementia from Alzheimer’s Disease. Dement Geriatr Cogn Dis Extra. 2013;3(1):376–85.

10.	Wong S, Flanagan E, Savage G, Hodges JR, Hornberger M. Contrasting prefrontal cortex contributions to episodic memory dysfunction in behavioural variant frontotemporal dementia and alzheimer’s disease. PLoS One. 2014;9(2).

11.	Rao SP, Nandi R, Dutt A, Kapur N, Harris JM, Thompson JC, et al. Distinct performance profiles on the Brixton test in frontotemporal dementia. J Neuropsychol. 2021;15(2):162–85.

12.	Nieuwboer A, Baker K, Willems AM, Jones D, Spildooren J, Lim I, et al. The short-term effects of different cueing modalities on turn speed in people with parkinson’s disease. Neurorehabil Neural Repair. 2009;23(8):831–6.

13.	De Frias CM, Dixon RA, Strauss E. Structure of four executive functioning tests in healthy older adults. Neuropsychology. 2006;20(2):206–14.

14.	de Frias CM, Dixon RA, Strauss E. Characterizing Executive Functioning in Older Special Populations: From Cognitively Elite to Cognitively Impaired. Neuropsychology. 2009;23(6):778–91.

15.	Rao SP, Nandi R, Dutt A, Kapur N, Harris JM, Thompson JC, et al. Distinct performance profiles on the Brixton test in frontotemporal dementia. J Neuropsychol. 2021 Jun 1;15(2):162–85.

16.	Marczewski P, Van der Linden M, Laroøi F. Further investigation of the Supervisory Attentional System in schizophrenia: Planning, inhibition, and rule abstraction. Cogn Neuropsychiatry. 2001;6(3):175–92.

17.	Tchanturia K, Anderluh MB, Morris RG, Rabe-Hesketh S, Collier DA, Sanchez P, et al. Cognitive flexibility in anorexia nervosa and bulimia nervosa. Journal of the International Neuropsychological Society. 2004 Jul;10(4):513–20.

18.	Bielak AAM, Mansueti L, Strauss E, Dixon RA. Performance on the Hayling and Brixton tests in older adults: norms and correlates. Arch Clin Neuropsychol [Internet]. 2006 [cited 2025 Mar 18];21(2):141–9. Available from: https://pubmed.ncbi.nlm.nih.gov/16242905/

19.	Van Den Berg E, Nys GMS, Brands AMA, Ruis C, Van Zandvoort MJE, Kessels RPC. The Brixton spatial anticipation test as a test for executive function: Validity in patient groups and norms for older adults. Journal of the International Neuropsychological Society. 2009;15(5):695–703.

20.	Spitoni GF, Bevacqua S, Cerini C, Ciurli P, Piccardi L, Guariglia P, et al. Normative Data for the Hayling and Brixton Tests in an Italian Population. Arch Clin Neuropsychol [Internet]. 2018 Jun 1 [cited 2025 Mar 18];33(4):466–76. Available from: https://pubmed.ncbi.nlm.nih.gov/28961765/

21.	Ghosh BCP, Carpenter RHS, Rowe JB. A longitudinal study of motor, oculomotor and cognitive function in progressive supranucelar palsy. PLoS One. 2013;8(9):e74486.

22.	Cavallo M, Hunter EM, van der Hiele K, Angilletta C. Computerized Structured Cognitive Training in Patients Affected by Early-Stage Alzheimer’s Disease is Feasible and Effective: A Randomized Controlled Study. Arch Clin Neuropsychol [Internet]. 2016 Dec 1 [cited 2021 Dec 14];31(8):868–76. Available from: http://www.ncbi.nlm.nih.gov/pubmed/27600448

23.	Lord S, Baker K, Nieuwboer A, Burn D, Rochester L. Gait variability in Parkinson’s disease: An indicator of non-dopaminergic contributors to gait dysfunction? J Neurol. 2011;258(4):566–72.

24.	Saenz A, Doé de Maindreville A, Henry A, de Labbey S, Bakchine S, Ehrlé N. Recognition of facial and musical emotions in Parkinson’s disease. Eur J Neurol. 2013;20(3):571–7.

25.	Martini A, Ellis SJ, Grange JA, Tamburin S, Dal Lago D, Vianello G, et al. Risky decision-making and affective features of impulse control disorders in Parkinson’s disease. J Neural Transm. 2018;125:131–43.

26.	Rochester L, Hetherington V, Jones D, Nieuwboer A, Willems AM, Kwakkel G, et al. Attending to the task: Interference effects of functional tasks on walking in Parkinson’s disease and the roles of cognition, depression, fatigue, and balance. Arch Phys Med Rehabil. 2004;85(10):1578–85.

27.	Rochester L, Nieuwboer A, Baker K, Hetherington V, Willems AM, Kwakkel G, et al. Walking speed during single and dual tasks in Parkinson’s disease: Which characteristics are important? Movement Disorders. 2008;23(16):2312–8.

28.	Mole JA, Josephs L, Prangnell SJ. Impaired option generation underpins deficient reasoning in Parkinson’s disease patients with apathy. Applied Neuropsychology:Adult. 2020;0(0):1–6.

29.	Zacharia SJ, Sokratous G, Samuel M, Costello A, Ashkan K, Shotbolt P. Neuropsychological and Neuropsychiatric Concerns for Deep Brain Stimulation in Dystonia: Preoperative Profiles in a Deep Brain Stimulation Cohort and Postoperative Changes in Three Case Series Reports. Cureus. 2018;10(10).

30.	Zacharia SJ, Sokratous G, Samuel M, Costello A, Ashkan K, Shotbolt P. Neuropsychological and Neuropsychiatric Concerns for Deep Brain Stimulation in Dystonia: Preoperative Profiles in a Deep Brain Stimulation Cohort and Postoperative Changes in Three Case Series Reports. Cureus. 2018 Oct 29;

31.	Bagshaw R, Gray NS, Snowden RJ. Executive function in psychopathy: The Tower of London, Brixton Spatial Anticipation and the Hayling Sentence Completion Tests. Psychiatry Res. 2014;220(1–2):483–9.

32.	Leppanen J, Adamson J, Tchanturia K. Impact of Cognitive Remediation Therapy on Neurocognitive Processing in Anorexia Nervosa. Front Psychiatry [Internet]. 2018 Mar 20 [cited 2021 Dec 14];9(MAR):96. Available from: http://www.ncbi.nlm.nih.gov/pubmed/29615940

33.	Lounes N, Khan G, Tchanturia K. Assessment of cognitive flexibility in anorexia nervosa--self-report or experimental measure? A brief report. J Int Neuropsychol Soc [Internet]. 2011 Sep [cited 2021 Dec 14];17(5):925–8. Available from: http://www.ncbi.nlm.nih.gov/pubmed/21729407

34.	Lorente-Rovira E, Santos-GÓmez JL, Moro M, Villagrán JM, McKenna PJ. Confabulation in schizophrenia: A neuropsychological study. Journal of the International Neuropsychological Society. 2010 Nov;16(6):1018–26.

35.	Mole J, Foddai E, Chan E, Xu T, Cipolotti L. Is the Brixton Spatial Anticipation Test sensitive to frontal dysfunction? Evidence from patients with frontal and posterior lesions. J Clin Exp Neuropsychol. 2020;00(00):531–43.

36.	Wood RL, Liossi C. The ecological validity of executive tests in a severely brain injured sample. Archives of Clinical Neuropsychology. 2006;21(5):429–37.

37.	Andrés P, Van der Linden M. Age-related differences in supervisory attentional system functions. J Gerontol B Psychol Sci Soc Sci [Internet]. 2000 [cited 2025 Mar 18];55(6):P373–80. Available from: https://pubmed.ncbi.nlm.nih.gov/11078107/

**Rating test evaluation**

| **Test name:** | Design Fluency Test |
| --- | --- |
| **Version**  Are there several versions (alternate or parallel) of the test? If so, which one has been assessed? | Yes. Here we review: the Delis-Kaplan Executive Function System (D-KEFS) version of DF(1)  Other versions:  Design Fluency(2); Five-Point Test; Ruff Figural fluency Test(3) |
| **Description of test**   - What population the test was originally developed for? - Which domains/components of domains do the test assess? - Are there multiple domains or a predominant domain assessed? - Does it score impaired/unimpaired? - Does it score severity   (subtle/mild/moderate/severe)? | The Design Fluency Test is part of the Delis–Kaplan Executive Function System (D-KEFS), which is a neuropsychological test used to measure a variety of verbal and nonverbal executive functions for both children and adults (ages 8–89 years).  The Design Fluency Test measures planning, cognitive flexibility, and fluency in generation of visual patterns, above and beyond contributions from motor speed.  Examinees draw as many different designs as possible in one minute, while avoiding repeating prior designs. There are several versions of DF tests, most of which require that designs be drawn by connecting dots in a series  of five dot matrices.  The DF consists of three trials in which participants create novel designs by connecting dots in a series of five dot matrices. The three conditions are referred to as Filled Dots (connecting filled dots), Empty dots (connecting empty dots while filled dots function as distractors), and Switch (switching between connecting filled and empty dots).  The main cognitive domain is Executive functions, and the specific process are those listed above.  Scoring methods allows to determine if performance is in the normal or pathological range as well as severity. |
| **Scaling Metric**: Type of response format? (e.g., Likert, Binary, multiple-choice, continuum, reaction time, etc.) | Specify the scale of measurement:  Primary measures:  a) Total Correct Composite Score (Conditions 1 + 2 + 3)  b) Condition 1 Filled Dots Total Correct  c) Condition 2 Filled Dots Total Correct  d) Condition 3 Switching Total Correct  Optional scores:  **• Total Set-Loss Design Errors** - can signal mild problems (e.g., an occasional fifth line in the designs) to more  serious problems (e.g., scribbling).    **• Total Repeated Design Errors** - may signal perseverative tendencies, especially if the initial and  repeated designs occur in proximity, or memory problems, particularly if the initial and repeated designs occur further apart.    **• Total Attempted Designs** - poor problem-solving behaviors result in a low score on total attempted designs. May be related to neurostructural involvement (e.g., activation problems caused by subcortical or diffuse damage) or non-neurostructural factors (e.g., depression, anxiety, obsessiveness, or lack of effort)  **•Percent Design Accuracy** - a percentage of Total Attempted Designs divided by the Total Number  Correct. |
| **Respondent: Patient:** any specific requirements for the target study population (e.g. non-demented, motor, auditory, visual abilities, etc)? | Because of the visual and motor nature of the task, patients must have the ability to move hands, and enough visual accuracy as to see the stimuli |
| **Access:** How can the test be obtained (mail address or website)? | Pearson Clinical |
| Copyrighted or in public domain?  Conditions for use? Fee? | Copyrighted |
| **Translations:** Has the test been translated and validated in other languages besides English? | Yes, in several languages |

| **Test properties**  (Please provide references for all statements or note if this is your judgment) |  |
| --- | --- |
| Which cognitive domain (or component/specific aspect of a cognitive domain) does this test *primarily* measure? | Executive function  The test covers several processes of executive functions (planning, cognitive flexibility, and fluency in generation of visual patterns, above and beyond contributions from motor speed). |
| Which aspects of the domain you are assessing are not covered? | Other components of EEFF (inhibition, lexical retrieval, …) |

| **Use** |  |
| --- | --- |
| Was it *designed* to measure severity, screen or diagnosis of the domain? | Yes. |
| Is there a cut-off score? Are there measures of screening/diagnostic performance of the test? (Receiver characteristic curve analyses; sensitivity/specificity; Positive Predictive Value/Negative Predictive Value), True Positive Rate, False Positive Rate etc.) | Cut-off score in relation to the diagnostic unit (MCI/dementia)  No / NA |
| Is this appropriate for the PD population? If not, why (low sensitivity and/or specificity values)? | Absence of studies allowing us to consider the appropriateness of this test in PD. Absence of reliable data regarding sensitivity/specificity or performance in PD and subgroups |
| **Clinimetric/psychometric properties in the target population** |  |
| **Feasibility /Acceptability** |  |
| Length | NA |
| Ambiguities in instructions to patient | N |
| Ambiguities in rating anchors | N |
| Appropriateness of questions for population | Y/N, if Y, specify |
| Applicability across disease cognitive stages | MCI/Dementia 🡪 applicability in normal cognition and PD-MCI seems reasonable. Not for dementia (personal judgement) |
| Applicability across disease motor severity? | H&Y score: 1 (Unilateral involvement only) 🡪 YES  H&Y score: 2 or 3(bilateral involvement with or without postural instability) 🡪 Yes (Jaywant et al., 2014)  H&Y score: ≥4(several disabilities or confined to bed) 🡪 impossible due to motor compromise (personal judgement) |
| Clear instructions to raters | Y specifies, provided in the manual of the D-KEFS |
| Has the test been used by researcher other than developers?  By other groups  Not applicable? | Yes, D-KEFS is used worldwide. |
| Are there instructions for dealing with missing data?  Not applicable? | NA |
| Floor and ceiling effects, skewed score distributions? | Not reported |

| **Have the component of dimensionality, variability and dependency structures been analyzed?**  (Exploratory and confirmatory factor analysis (EFA and CFA)? | NA |
| --- | --- |
|  |  |
| **Reliability: assessed – good; not good. Not assessed**  Are alternative versions developed?  If so, were they validated with reliability measures? | Yes. There are alternate forms but not for Design Fluency. Normative data for  the alternate forms were derived through calibration procedures from a separate study involving 286 examinees between the ages of 16–89. Alternate-form reliabilities were provided for three of the D-KEFS tests, including the Verbal Fluency Test, Sorting Test, and Twenty Questions Test, but not for Design Fluency |
| Internal consistency (A rule of thumb)  0.9 ≤ Cronbach’s alpha (α) Excellent  0.8 ≤ α < 0.9 Good  0.7 ≤ α < 0.8 Acceptable  0.6 ≤ α < 0.7 Questionable  0.5 ≤ α < 0.6 Poor  α < 0.5 Unacceptable  (Streiner, 2003 Journal of Personality Assessment, 80:1, 99-103, DOI: 10.1207/S15327752JPA8001_18) | The 144-page technical manual provides evidence of reliability and validity. Internal consistency (split-half coefficients corrected by the Spearman-Brown formula and alternate  form reliability), test-retest reliability, standard errors of measurement, and confidence intervals were estimated for each D-KEFS test or test condition within each test. The manual is not on the hands of the present reviewer to further indicate the specific values. |
| “Corrected item-to-total correlation” | Y, but exact data is on the technical manual(4). |
| Test/retest | Y, but exact data is on the technical manual. |
| Inter-rater | Y, but exact data is on the technical manual. |
| Intra-rater | Y, but exact data is on the technical manual. |
| Other (e.g. Standard Error of Measurement) | Y/N, if Y, specify how large |
| **Validity: assessed –good; not good. Not assessed (reference)** |  |
| Face and Content validity (does test appears to be measuring what it is intended to measure and do test’s items comprehensively represent the domain of interest?) | Yes |
| Criterion validity (compared to gold-standard) or other criteria for  diagnosis) (e.g. other comparable tests or lesion or MRI research) | Several studies supporting the association between frontal lobe damage, executive disfunction and performance in the DF test.  Validity studies with the D-KEFS indicate that the various measures from the D-KEFS tests have reasonable sensitivity in distinguishing many different types of clinical groups (e.g., fetal alcohol exposure, schizophrenia, chronic alcoholism, Parkinson’s disease, focal  frontal lesions, etc.) from normal controls. No specific data for the DF test. |
| Construct validity (correlations with other convergent tests and divergent tests; known-groups comparisons) | Evidence for convergent and discriminant validity was provided. To provide evidence of convergent validity, test developers presented correlations between the D-KEFS California  Card Sorting Test (CCST) and the Wisconsin Card Sorting Test (WCST) from a small pilot study (n=23) and moderate correlations were found between the tests. Unfortunately, no data is provided for Design Fluency in isolation I |
| Reference group used to develop this test:   67) If available, please define the characteristic of the norm group adopted (demographic variables used for stratification: age, education, sex, etc). 68) What kind of standardized score generated (T score, Z score, derived IQ score, percentile ranks, etc)? 69) If translated/validated in other languages, are reference norms created? If so, please specify demographic variables used for stratification (age, education, gender etc). | Group characteristics: The D-KEFS was standardized on a nationally representative, stratified sample of 1750  non-clinical children, adolescents, and adults, ages 8–89 years old. The D-KEFS sample consisted of roughly equal proportions of men and women at each  age group, with the exception of the older age groups, which had more women than men. Age effects for each D-KEFS test were reported in the technical manual. The standard  deviations of the D-KEFS variables tended to be larger in the youngest and oldest age groups than in the adolescent, young-adult, and middle-aged groups.  Add the language of translation and group characteristics:  The D-KEFS is translated and validated in several (almost all) languages. Specific details are in the technical manual for each language just accessible through Pearson Clinical |

| **Responsiveness: assessed –good; not good. Not assessed (reference)** |  |
| --- | --- |
| Demonstrated to be sensitive to change (change over time or due to treatment) *  * [determined by the strength of the relationship between change in the test scores and the criterion or anchor scores] | I have not found enough data to allow me to confirm or deny this |
| Has the minimal clinically important change and minimal clinically relevant incremental difference been assessed?  (besides a statistically significant improvement) | NO |
| Has this test been assessed or used in patients with other condition than that of interest? | Yconditions. TBI, AD, HD, Schizophrenia, Alcoholism, ADHD, Depression, bipolar disorder. |
| Other |  |
| **Overall impression** |  |
| Strengths | It is a robust subtest of a widely accepted neuropsychological assessment battery (D-KEFS) that allows to take into account the motor component during task execution |
| Weaknesses | No specific validation studies in PD. Extremely expensive. |

| **Level of Recommendation** |  |
| --- | --- |
| “Recommended” – The test was applied to the target population, AND was studied clinimetrically and found to be valid, reliable and sensitive to change, AND was used in studies by researchers, others than the developers  “Recommended with caveats” – Test’s properties were generally found to be adequate, but some of the measurement properties were not evaluated or not evaluated specifically in a PD cohort."  ‘Suggested’ – The test was applied to the target population, but only one of the other criteria applies  ‘Listed” – The test was applied to the target population, but none of the other criteria applies | Suggested |
| Which type of study is this test suitable for and which type of study is it not suitable for (screening, prevalence, etiological (e.g. case-control or genetic), treatment trial, correlation with biological markers or other scales, e.g. of parkinsonism, clinical practice for diagnosis/ screening). List all. | Suitable for:  Screening  Prevalence  Case-control study  Treatment trial  Biomarker study  Other  Unsuitable for (please add just categories for which the measure is critically unsuitable):  Differential reasoning (etiological) |

**REFERENCES**

1.	Delis DC, Kaplan E, Kramer JHJA**.** Delis-Kaplan executive function system. 2001.

2.	Jones-Gotman M, Milner B**.** Design fluency: the invention of nonsense drawings after focal cortical lesions. Neuropsychologia. 1977;15(4-5):653-74.

3.	Ruff RM, Allen CC, Farrow CE, Niemann H, Wylie T**.** Figural fluency: differential impairment in patients with left versus right frontal lobe lesions. Arch Clin Neuropsychol. 1994;9(1):41-55.

4.	Homack S, Lee D, Riccio CA**.** Test review: Delis-Kaplan executive function system. J Clin Exp Neuropsychol. 2005;27(5):599-609.

1. In the WAIS-IV manual, qualitative descriptors are not provided for the subtest scores and it is not recommended to use the ‘composite’ descriptors, because their score ranges differed to substantially. There are some references, which do provide qualitative descriptors for subtests that are based on the standard deviations (<http://pearsonassessmentsupport.com/support/index.php?View=entry&EntryID=2494>). Similarly, the Italian validation and standardization of the WAIS-IV (7) allows three, five and seven categorizations of scaled scores. For instance, the 5-category system describes the *scaled scores* as follows: *scaled score from 1–3* as exceptional weakness, very poorly developed, or far below average with a corresponding percentile rank (PR) of 0.1*–*1; *scaled score from 4–6* as weakness, poorly developed, or below average with a corresponding PR of 2*–*9; *scaled score from 7–13* as average with a corresponding PR of 16*–*84; *scaled score from 14–16* as strength, well developed, or above average with a corresponding PR of 91*–*98, and *scaled score from 17–19* as exceptional strength, very well developed, or superior with a corresponding PR of 99*–*99.9.
2. <https://www.pearsonassessments.com/professional-assessments/featured-topics/pharma-research-services/how-to-choose-a-test.html>
3. In this and the following section (feasibility/acceptability and reliability), the reported statistic results are based largely on the WAIS-IV Manual (1, 2) or on the WAIS-IV Manual of the Italian validation of WAIS-IV (7). Unfortunately, the WAIS-IV manuals are not freely available. However, the Italian validation was based on a large sample similar to the American one in terms of sample size and demographic variables. In addition, the clinimetric/psychometric properties are comparable to those of the American validation (7).
4. In the WAIS-IV manual, qualitative descriptors are not provided for the subtest scores and it is not recommended to use the ‘composite’ descriptors, because their score ranges differed to substantially. There are some references, which do provide qualitative descriptors for subtests that are based on the standard deviations (<http://pearsonassessmentsupport.com/support/index.php?View=entry&EntryID=2494>). Similarly, the Italian validation and standardization of the WAIS-IV (16) allows three, five and seven categorizations of scaled scores. For instance, the 5-category system describes the *scaled scores* as follows: *scaled score from 1–3* as exceptional weakness, very poorly developed, or far below average with a corresponding percentile rank (PR) of 0.1*–*1; *scaled score from 4–6* as weakness, poorly developed, or below average with a corresponding PR of 2*–*9; *scaled score from 7–13* as average with a corresponding PR of 16*–*84; *scaled score from 14–16* as strength, well developed, or above average with a corresponding PR of 91*–*98, and *scaled score from 17–19* as exceptional strength, very well developed, or superior with a corresponding PR of 99*–*99.9.
5. <https://www.pearsonassessments.com/professional-assessments/featured-topics/pharma-research-services/how-to-choose-a-test.html>
6. In this and the following section (feasibility/acceptability and reliability), the reported statistic results are based largely on the WAIS-IV Manual (1, 2) or on the WAIS-IV Manual of the Italian validation of WAIS-IV (16). Unfortunately, the WAIS-IV manuals are not freely available. However, the Italian validation was based on a large sample similar to the American one in terms of sample size and demographic variables. In addition, the clinimetric/psychometric properties are comparable to those of the American validation (16).
7. In the WAIS-IV manual, qualitative descriptors are not provided for the subtest scores and it is not recommended to use the ‘composite’ descriptors, because their score ranges differed to substantially. There are some references, which do provide qualitative descriptors for subtests that are based on the standard deviations (<http://pearsonassessmentsupport.com/support/index.php?View=entry&EntryID=2494>). Similarly, the Italian validation and standardization of the WAIS-IV (4) allows three, five and seven categorizations of scaled scores. For instance, the 5-category system describes the *scaled scores* as follows: *scaled score from 1–3* as exceptional weakness, very poorly developed, or far below average with a corresponding percentile rank (PR) of 0.1*–*1; *scaled score from 4–6* as weakness, poorly developed, or below average with a corresponding PR of 2*–*9; *scaled score from 7–13* as average with a corresponding PR of 16*–*84; *scaled score from 14–16* as strength, well developed, or above average with a corresponding PR of 91*–*98, and *scaled score from 17–19* as exceptional strength, very well developed, or superior with a corresponding PR of 99*–*99.9.
8. <https://www.pearsonassessments.com/professional-assessments/featured-topics/pharma-research-services/how-to-choose-a-test.html>
9. In this and the following section, about feasibility/acceptability and reliability, the reported statistic results are based on the WAIS-IV Manual (1, 2) or on the WAIS-IV Manual of the Italian validation of WAIS-IV (4). Unfortunately, the WAIS-IV manuals are not freely available. However, the Italian validation was based on a large sample similar to the American one in terms of sample size and demographic variables. In addition, the clinimetric/psychometric properties are comparable to those of the American validation (4).
10. In the WAIS-IV manual, qualitative descriptors are not provided for the subtest scores and it is not recommended to use the ‘composite’ descriptors, because their score ranges differed to substantially. There are some references, which do provide qualitative descriptors for subtests that are based on the standard deviations (<http://pearsonassessmentsupport.com/support/index.php?View=entry&EntryID=2494>). Similarly, the Italian validation and standardization of the WAIS-IV (3) allows three, five and seven categorizations of scaled scores. For instance, the 5-category system describes the *scaled scores* as follows: *scaled score from 1–3* as exceptional weakness, very poorly developed, or far below average with a corresponding percentile rank (PR) of 0.1*–*1; *scaled score from 4–6* as weakness, poorly developed, or below average with a corresponding PR of 2*–*9; *scaled score from 7–13* as average with a corresponding PR of 16*–*84; *scaled score from 14–16* as strength, well developed, or above average with a corresponding PR of 91*–*98, and *scaled score from 17–19* as exceptional strength, very well developed, or superior with a corresponding PR of 99*–*99.9.
11. <https://www.pearsonassessments.com/professional-assessments/featured-topics/pharma-research-services/how-to-choose-a-test.html>
12. In this and the following section, about feasibility/acceptability and reliability, the reported statistic results are based on the WAIS-IV Manual (1, 2) or on the WAIS-IV Manual of the Italian validation of WAIS-IV (3). Unfortunately, the WAIS-IV manuals are not freely available. However, the Italian validation was based on a large sample similar to the American one in terms of sample size and demographic variables. In addition, the clinimetric/psychometric properties are comparable to those of the American validation (3).
13. Kent, P. L. (2017). Evolution of Wechsler’s Memory Scales: Content and structural analysis. *Applied Neuropsychology: Adult*, *24*(3), 232-251.
14. Parrao, T., Chana, P., Venegas, P., Behrens, M. I., & Aylwin, M. L. (2012). Olfactory deficits and cognitive dysfunction in Parkinson’s disease. *Neurodegenerative Diseases*, *10*(1-4), 179-182.
15. Drag, L. L., Bieliauskas, L. A., Kaszniak, A. W., Bohnen, N. I., & Glisky, E. L. (2009). Source memory and frontal functioning in Parkinson’s disease. *Journal of the International Neuropsychological Society*, *15*(3), 399-406.
16. Wechsler Memory Scale-Fourth Edition (WMS-IV). Technical and Interpretative Manual (D. Wechsler, 2008).
